# Mitochondrion to endoplasmic reticulum apposition length in zebrafish embryo spinal progenitors is unchanged in response to perturbations associated with Alzheimer’s disease

**DOI:** 10.1371/journal.pone.0179859

**Published:** 2017-06-21

**Authors:** Morgan Newman, Lena Halter, Anne Lim, Michael Lardelli

**Affiliations:** Alzheimer’s Disease Genetics Laboratory, Centre for Molecular Pathology, School of Biological Sciences, University of Adelaide, Adelaide, South Australia, Australia; Torrey Pines Institute for Molecular Studies, UNITED STATES

## Abstract

Mutations in the human genes *PRESENILIN1* (*PSEN1*), *PRESENILIN2* (*PSEN2*) and *AMYLOID BETA A4 PRECURSOR PROTEIN* (*APP)* have been identified in familial Alzheimer’s disease (AD). The length of mitochondrion-endoplasmic reticulum (M-ER) appositions is increased in *Psen1*^*-/-*^*/Psen2*^*-/-*^ double knockout murine embryonic fibroblasts and in fibroblasts from AD-affected individuals. Development of an easily accessible, genetically manipulable, *in vivo* system for studying M-ER appositions would be valuable so we attempted to manipulate M-ER apposition length in zebrafish (*Danio rerio*) embryos. We injected fertilized zebrafish eggs with antisense morpholino oligonucleotides (MOs) that inhibit expression of zebrafish familial AD gene orthologues *psen1* and *psen2*. Furthermore, we treated zebrafish embryos with DAPT (a highly specific γ-secretase inhibitor) or with sodium azide (to mimic partially hypoxic conditions). We then analyzed M-ER apposition in an identified, presumably proliferative neural cell type using electron microscopy. Our analysis showed no significant differences in M-ER apposition lengths at 48 hours post fertilization (hpf) between *psen1* & *psen2* MO co-injected embryos, embryos treated with DAPT, or sodium azide, and control embryos. Instead, the distribution of M-ER apposition lengths into different length classes was close to identical. However, this indicates that it is feasible to reproducibly measure M-ER size distributions in zebrafish embryos. While our observations differ from those of murine and human studies, this may be due to differences in cellular differentiation and metabolic state, cell age, or species-specific responses. In particular, by focusing on a presumably proliferative embryonic cell type, we may have selected a cell heavily already reliant on anaerobic glycolysis and less responsive to factors affecting M-ER apposition. Future examination of more differentiated, more secretory cell types may reveal measurable responses of M-ER apposition to environmental and genetic manipulation.

## Introduction

Alzheimer’s disease (AD) is a neurodegenerative disorder characterized by the occurrence of memory loss in its initial stages, with other effects such as the impairment of speech and motor ability, depression, hallucinations, behaviour disturbances and, ultimately death in more advanced stages of the disease [reviewed in [1]]. The major neuropathological hallmarks of the disease in the brain are extracellular deposits of Amyloid beta (Aβ) peptide in “plaques”, and intracellular neurofibrillary tangles, which are composed of hyperphosphorylated forms of the tau protein. The exact mechanism of the disease remains unclear. There have been numerous hypotheses suggested with the most widely accepted being the amyloid cascade hypothesis [2]. This posits that the accumulation of Aβ, either via overproduction or lack of clearance, leads to its oligomerization and deposition in the brain and, ultimately, to neuronal dysfunction, degeneration and death.

The majority of AD cases are sporadic (sAD), with a small number of cases that are familial (fAD). Familial AD characteristically has an early age of onset (<65 years). Although only accounting for a small percentage of AD cases, most of our understanding of the molecular events underlying the development of AD comes from fAD, since genetic analysis can be used to identify the genes and proteins involved.

Mutations in the *PRESENILIN* genes (*PSEN1* and *PSEN2*) [3, 4] and the *AMYLOID BETA A4 PRECURSOR PROTEIN* (*APP*) [5] gene have been identified in fAD. The PRESENILIN proteins are the catalytic components of γ-secretase enzyme complexes which cleave type I transmembrane proteins. APP, Notch, E-cadherin, Jagged and a great number of other proteins are substrates of γ-secretase [6]. The cleavage of APP by γ-secretase forms the Aβ peptides found in the amyloid plaques of AD brains [7]. It has been shown that AD associated *PRESENILIN* mutations commonly cause partial or severe loss of function of γ-secretase [8]. Accordingly, γ-secretase-specific inhibitors such as DAPT have been suggested as an attractive tool for study of AD pathogenesis and several γ-secretase inhibitors have entered trials to inhibit Aβ production as a potential AD therapy [reviewed in [9]. (This approach was recently criticized by Kelleher and Shen who suggested instead that drugs be found to restore the lost γ-secretase activity [10].) The therapeutic failure of γ-secretase inhibitor trials has been ascribed, in part, to unintended inhibition of cleavage of other γ-secretase substrates such as Notch [11, 12]. However, PRESENILIN proteins also possess functions independent of their role in γ-secretase. For example, before endoproteolysis to activate its γ-secretase activity, the PRESENILIN1 holoprotein plays a role in the acidification of lysosomes through facilitation of glycosylation of the V0a1 subunit of v-ATPase [13]. PRESENILINs also regulate β-catenin stability via an Axin-independent pathway for phosphorylation of β-catenin [14] and form Ca^2+^ leak channels in the endoplasmic reticulum that allow the release of Ca^2+^ to the cytoplasm [15].

The subcellular localizations of the components of γ-secretase and its substrate APP have been of great interest since their elucidation would provide further insight into the pathogenesis of AD. Various studies have found PRESENILINs located in almost all membranous compartments of the cell [16–22]. A recent discovery by Area-Gomez and colleagues identified a previously unrecognized compartment of *PSEN1* and *PSEN2* enrichment. When examining mammalian cell membrane fractions they found the PRESENILINs to be located predominantly in the endoplasmic reticulum (ER) and specifically in a sub-compartment of the ER known as the mitochondria-associated ER membrane (MAM) [23]. The MAM is a lipid raft-like compartment [24] that contains various enzymes involved in critical cellular functions including the synthesis and transfer of phospholipids [25], oxidative protein folding [reviewed in [26]], cholesterol metabolism [27] and calcium homeostasis [28]. Interestingly, MAM is also the site of formation of autophagosomes [29] and is involved in the UPR [30]. The MAM is physically linked to the outer mitochondrial membrane by protein tethers that are sufficiently stable for MAM to be co-isolated with mitochondria by subcellular fractionation (reviewed in [31]). Further work by Area-Gomez et al. [32] looked at the degree of M-ER apposition in double knockout murine embryonic fibroblasts (MEFs) and AD patient cells by electron microscopy. Interestingly, they observed that the lengths of M-ER appositions are increased in *Psen1*^*-/-*^*/Psen2*^*-/-*^ double knockout MEFs and well as in fibroblasts from both fAD and sAD individuals compared to control fibroblasts. Increased levels of phospholipid and cholesteryl ester synthesis in MEFs and in fibroblasts from AD individuals were also seen along with the increase in M-ER physical association. Additionally, it has been shown that activity of the APOE ε4 isoform (the main genetic risk factor for sporadic AD) upregulates the physical extent and phospholipid synthesis activity of MAM [33]. This suggests that increased M-ER communication due to upstream events may be a common factor in fAD and sAD. Perturbations to MAM function might possibly result in particular features of AD that the amyloid hypothesis fails to explain.

The zebrafish, *Danio rerio*, is advantageous as a model organism for the study of human disease. Zebrafish embryos are robust and can undergo experimental manipulations such as the injection of MOs into embryos to modify simultaneously the expression of multiple genes. Embryos are produced in large numbers and develop rapidly [34]. Zebrafish possess genes orthologous to human *APP*, *PSEN1* and *PSEN2* along with the other components of the γ-secretase complex [35–37]. To date, the zebrafish animal model has not been used to study M-ER apposition. Our previous work has revealed aspects of PRESENILIN protein function not seen in cell culture systems [38–40]. We were, therefore, curious to see whether zebrafish might be an advantageous model in which to study the effects of Alzheimer´s disease-like conditions on MAM formation. Here, we determine the effects of inhibition of *psen1* and *psen2* activity, DAPT and sodium azide treatment on M-ER appositions in midline spinal cord cells in the trunk region of zebrafish embryos. The treatment with DAPT, a potent γ-secretase inhibitor, is of high interest to determine whether γ-secretase activity plays a direct role in affecting MAM formation apposition. Chemical agents like sodium azide are commonly used to mimic hypoxic conditions [41]. Sodium azide inhibits cytochrome-c oxidase (complex IV) of the mitochondrial electron transport chain thereby causing mitochondrial dysfunction [41]. A reduced activity of complex IV has been demonstrated several times in AD [42–44]. Mitochondrial dysfunction is associated with increased levels of reactive oxygen species (ROS) [45, 46] and there is increasing evidence suggesting the involvement of oxidative stress in AD [47–50]. There is also a large body of evidence suggesting that hypoxia plays a role in AD pathogenesis via its effect on cerebrovasculature. Correct functioning of cerebrovasculature is crucial for sufficient supply of oxygen and glucose to the brain. Reduced cerebral blood flow and changes in cerebrovasculature have been observed in AD [51–53]. Hence, sodium azide is a suitable compound to study the role of hypoxia in AD pathogenesis.

## Methods

### Ethics statement

All zebrafish work was performed under the auspices of The Animal Ethics Committee of The University of Adelaide.

### Animal husbandry

Wild-type zebrafish were maintained in a recirculated water system with a 14 hour light/10 hour dark cycle. Fertilized embryos were grown at 28˚C in aqueous support embryo medium (E3)[54].

### Morpholino microinjection of zebrafish embryos

The morpholinos used in this work have been extensively valided in our previous research [55, 56]. Morpholinos were synthesized by Gene Tools LLC (Corvallis, OR, USA) and are listed in Table 1. Fertilized zebrafish embryos were rinsed in E3 medium and injected at the one cell stage. To ensure consistency of MO injections, zebrafish eggs were always injected with solutions at 1 mM total concentration. (i.e. 0.5 mM MoPS1Tln + 0.5 mM MoPS2Tln, or 1.0 mM MoCont on its own).

**Table 1:** Morpholino sequences.

Morpholino	Morpholino Sequences (5’– 3’)
**MoCont**	CCTCTTACCTCAGTTACAATTTATA
**MoPS1Tln**	ACTAAATCAGCCATCGGAACTGTGA
**MoPS2Tln**	GTGACTGAATTTACATGAAGGATGA

### γ-Secretase inhibitor IX treatment

γ-Secretase Inhibitor IX (DAPT, N-[N-(3,5-Difluorophenacetyl)-L-alanyl]-S-phenylglycine t-butyl ester) in DMSO was purchased from Calbiochem (San Diego, CA, USA). This was added to the aqueous support E3 medium of 4 hpf embryos until they developed to 48 hpf so that the final concentration was 100 μM with 1% DMSO. We [57] and others [12] have previously shown this concentration to be effective in inhibiting γ-secretase activity in zebrafish embryos.

### Sodium azide treatment

Exposure of larvae to sodium azide (NaN_3_, Sigma-Aldrich CHEMIE GmbH, Steinheim, Germany) was performed at 100μM that we have previously demonstrated is an effective concentration for mimicry of hypoxia [58, 59]. NaN_3_ was added to the aqueous support medium of 36 hpf embryos until they developed to 48 hpf.

### Transmission electron microscopy

24 and 48 hpf zebrafish larvae were fixed in 4% paraformaldehyde, 1.25% glutaraldehyde in PBS with 4% sucrose, pH 7.2 overnight at 4˚C, and rinsed in washing buffer (PBS with 4% sucrose). Embryos were post-fixed in 2% osmium tetroxide, followed by dehydration through an ethanol series, and rinsed with propylene oxide followed by resin infiltration. Embedded embryos were sectioned laterally past the embryonic yolk ball, at the beginning of the yolk extension using an ultramicrotome to obtain several 85nm thick sections of the spinal cord. The ultrathin sections were stained with uranyl acetate and lead citrate and imaged on an Olympus-SIS Veleta CCD camera in a FEI Tecnai G2 Spirit TEM. Images were obtained from 3 to 6 cells at the midline area of the spinal cord; this was performed for 3 embryos of each treatment. The mitochondria-ER apposition lengths were measured using Image J software and statistically analysed using Fisher’s exact test [60].

## Results

A previous study by Area-Gomez et al. [32] found that MEFs as well as fibroblasts from patients with fAD and sAD show increased lengths of M-ER apposition compared to wild type (WT) cells. They found more appositions in the 50-200nm (long) and >200nm (very long) ranges compared to WT MEFs which show more appositions in a shorter length range <50nm. Interestingly, they found essentially no effect on the degree of M-ER apposition in HeLa cells after treatment with DAPT. Zebrafish have proven to be a highly manipulable model for analysis of *PRESENILIN* gene function and might prove to be a useful tool for analysis of the role of these genes in communication between the ER and mitochondria. Therefore, we sought to determine the effect of inhibition of *psen1* and *psen2* activity on M-ER apposition in the zebrafish animal model. Since our broader research focus is Alzheimer’s disease we wished to analyse M-ER apposition in a neural cell type. However, unlike analysis of M-ER apposition in cultured cells, the central nervous system of developing zebrafish embryos contains many cell types with varying energy production and protein secretion characteristics that might affect the extent of M-ER apposition within cells and confound the statistical analysis required to reveal changes in M-ER apposition length caused by experimental treatments. Additionally, it can be difficult to identify particular neural cell types by morphological criteria in sections observed by transmission electron microscopy (TEM). Therefore we chose cells near the midline of the developing spinal cord in the mid-trunk region of the embryo as relatively easily easy to identify reproducibly (by their position) and most likely representing a proliferative progenitor cell type that had not adopted a final differentiated state.

### Analysis in embryos at 24 hpf

**Fig 1:**
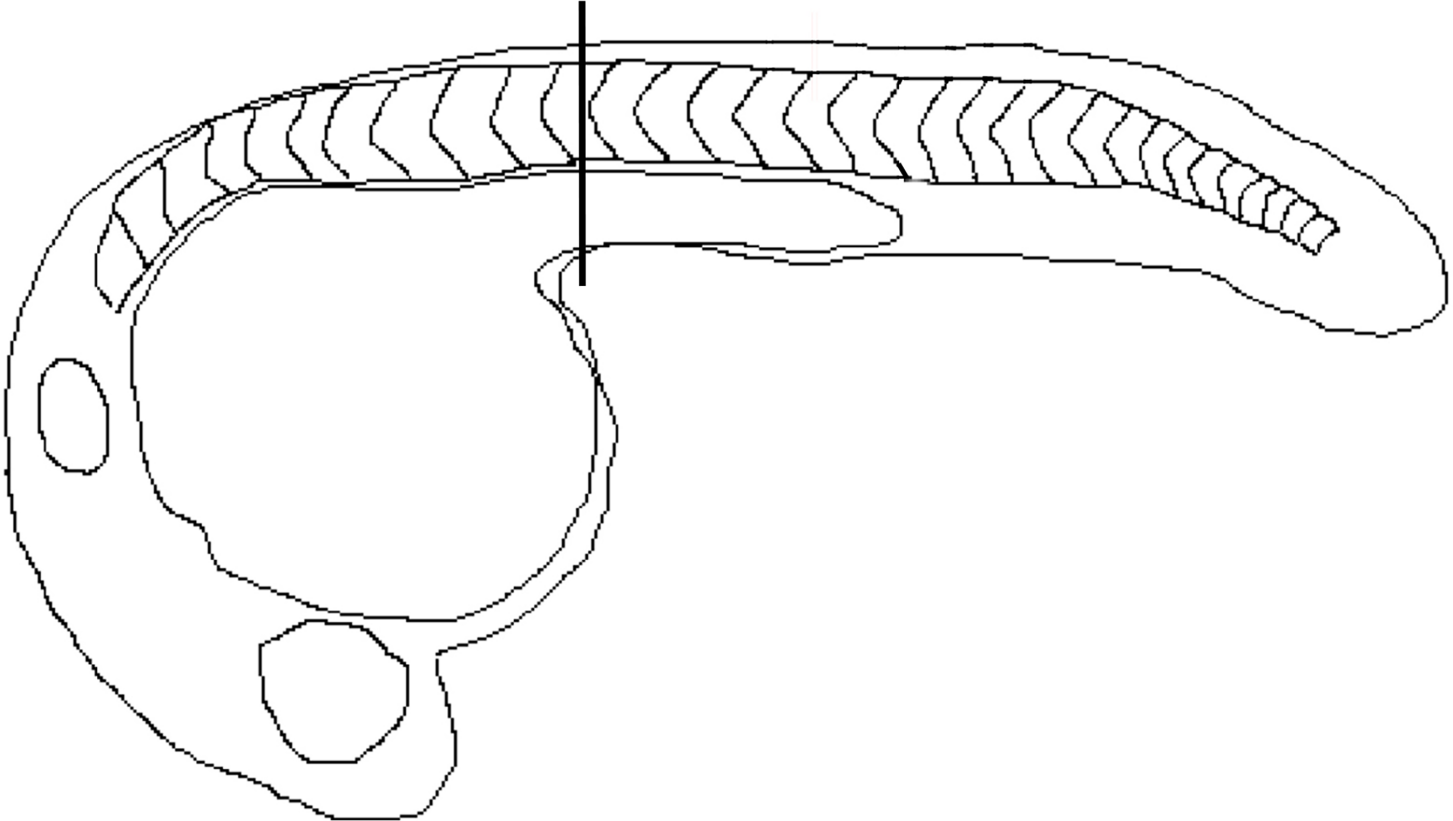
Zebrafish embryo. Several sections of 85 nm thickness were obtained from the area posterior to the yolk ball, within the yolk extension as indicated by the vertical line.

**Fig 2:**
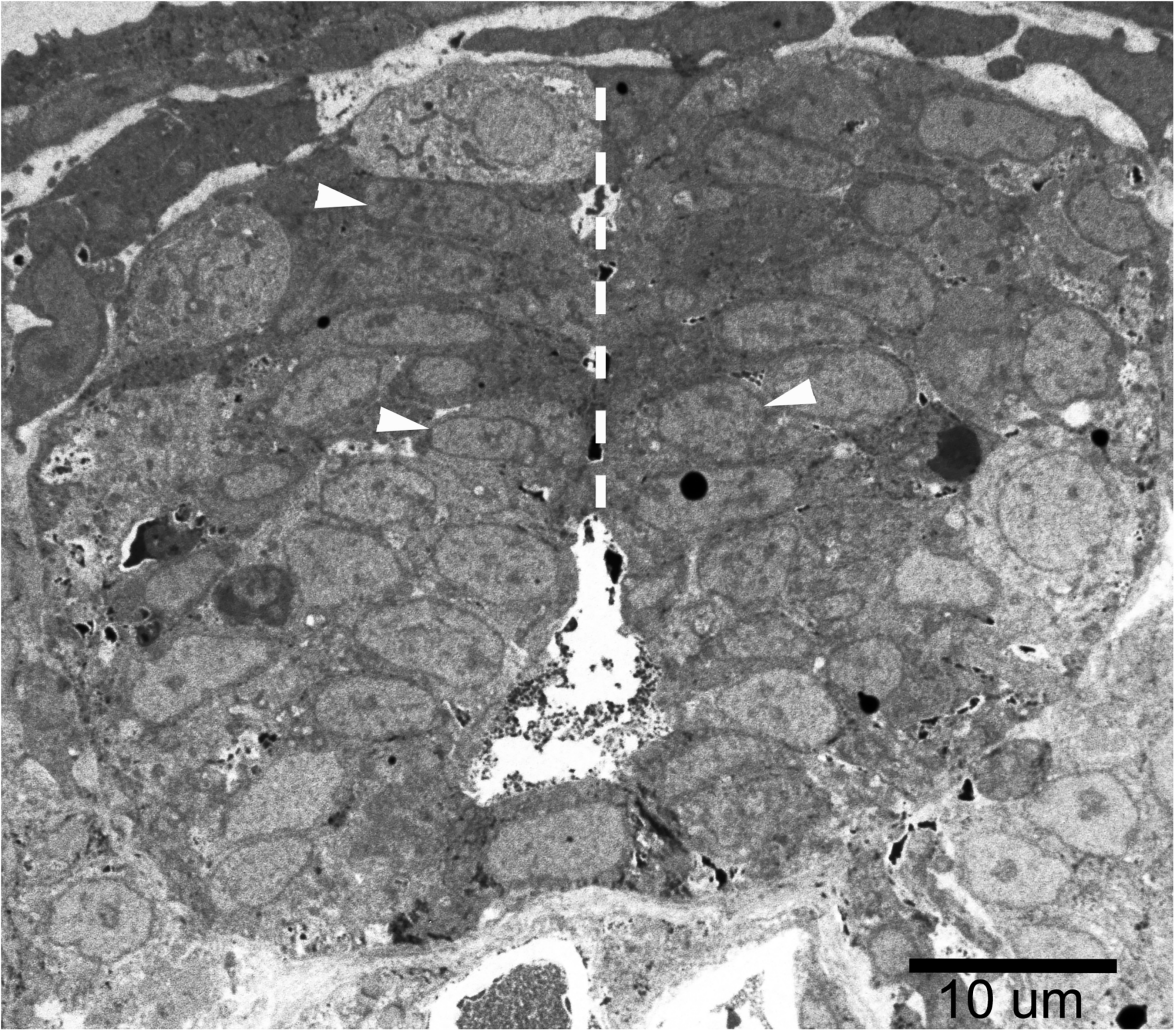
The midline of the spinal cord region. M-ER apposition lengths were measured in 3–6 cells at the midline area of the spinal cord at each embryo (such as those indicated by white arrowheads). Three embryos were examined for each treatment. The white, dashed line indicates the midline of the spinal cord.

**Fig 3:**
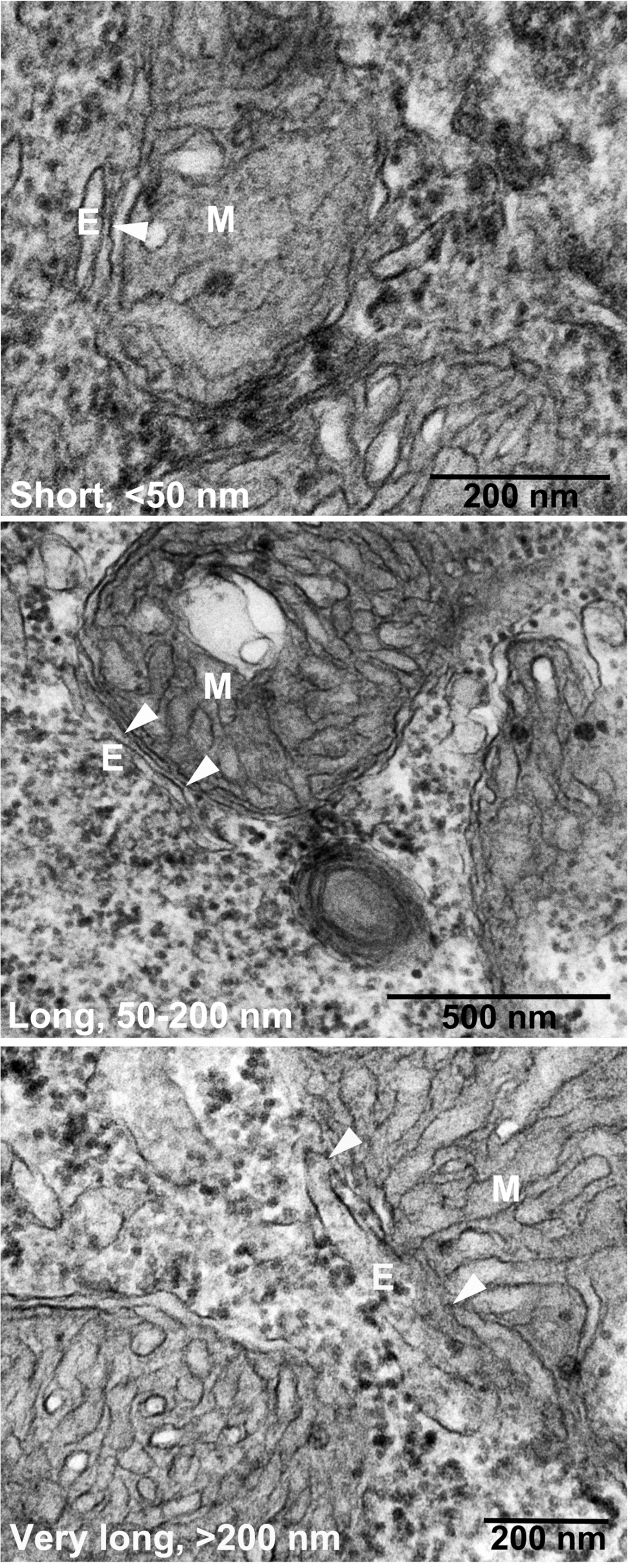
Electron microscopy of zebrafish neural cells. Cells were treated with morpholinos binding the start codon of *psen1* and *psen2* mRNA (MoPS1Tln and MoPS2Tln respectively). Arrowheads indicate the region of apposition between mitochondria (M) and endoplasmic reticulum (E) i.e. MAM.

**Fig 4:**
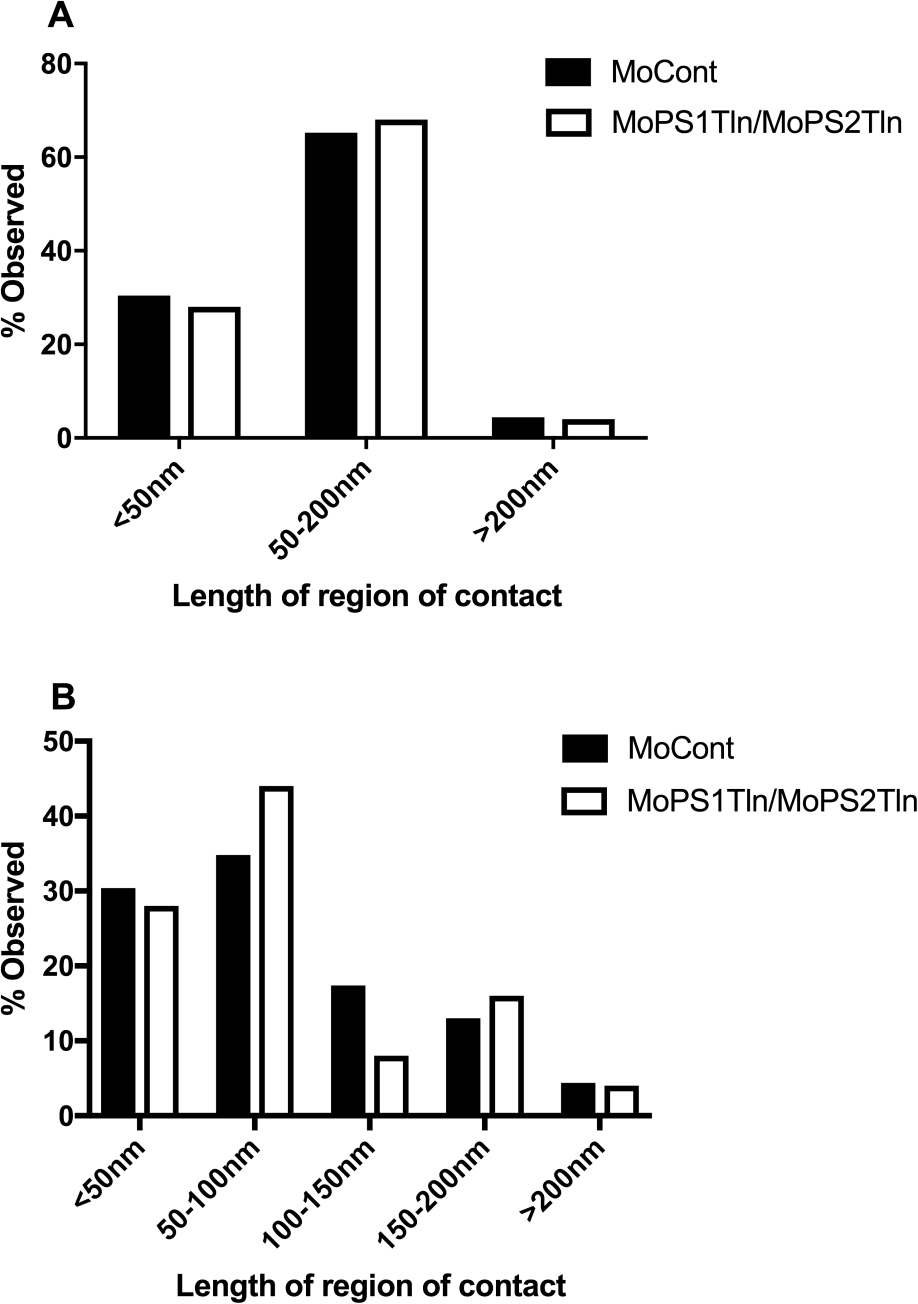
Quantification of 24 hpf zebrafish embryo M-ER apposition lengths. Percentage observed for each length class for 3 classes A) and 5 classes B). Total apposition events analysed were 23 in MoCont and 25 in MoPS1Tln/MoPS2Tln-injected embryos.

In an initial experiment we examined zebrafish embryos at 24 hpf in which we had simultaneously blocked translation of both the zebrafish Psen1 and Psen2 proteins by injection of embryos at the 1-cell stage with MOs binding over the start codons of the *psen1* and *psen2* mRNAs (MoPS1Tln plus MoPS2Tln respectively–we have previously used these successfully in numerous experiments [38–40]. We injected an inactive MO (MoCont) as a negative control. To standardise the neural cell type examined we sectioned transversely through the rostocaudal axis of embryos in that area of the trunk region of the embryo featuring the yolk extension (Fig 1). This region has an anatomical structure that does not vary greatly along the rostro-caudal axis. We then sought to identify cells in the spinal cord adjacent to the midline (Fig 2). Three embryos from each treatment were analysed. M-ER apposition lengths were then examined in three cells from each embryo and all the data were combined (Figs 3 and 4). A total of 23 appositions in MoCont-injected embryos and 25 appositions in MoPS1Tln and MoPS2Tln co-injected embryos were observed.

The individual M-ER apposition length measurements from our analyses at 24 hpf are shown in S1 Data. All EM images used for our analyses are available in S1–S6 Files.

We analysed our results in a manner similar to published work using cultured cells [32, 61]. First, the M-ER appositions were classed into three ranges of length; <50 nm, 50–200 nM, and >200 nm (Fig 4A and Table 2A). We were surprised by the similarity in overall M-EF length distribution between the control and treated embryos. We also examined the data when divided among five classes of length range; <50 nm, 50–100 nM, 100–150 nM, 150–200 nM, and >200 nm (Fig 4B and Table 2B). Analysis by Fisher’s exact tests [60] showed no significant difference in apposition length distribution between control embryos and those lacking zebrafish Presenilin activity. Total apposition events analysed were 23 in embryos injected with MoCont and 25 in embryos injected with both MoPS1Tln and MoPS2Tln. p = 1 for three classes and p = 0.844 for five classes.

**Table 2:** A) Percentage observed per length class from 24hpf, 3 classes. B) Percentage observed per length class from 24 hpf embryos, 5 classes.

**A**							
**Treatment**	**Length of region of apposition, mitochondrial perimeter (nm)**						
	**<50nm**		**50-200nm**			**>200nm**	
**MoCont injected (n = 22)**	31.8%(7)		63.6%(14)			4.5%(1)	
**MoPS1Tln/MoPS2Tln injected (n = 25)**	28%(7)		68%(17)			4%(1)	
**B**							
**Treatment**	**Length of region of apposition, mitochondrial perimeter (nm)**						
	**<50nm**	**50-100nm**		**100-150nm**	**150-200nm**		**>200nm**
**MoCont injected (n = 23)**	31.8%(7)	31.8%(7)		18.2%(4)	13.6%(3)		4.5%(1)
**MoPS1Tln/MoPS2Tln injected (n = 25)**	28%(7)	44%(11)		8%(2)	16%(4)		4%(1)

The failure to observe changes in M-ER apposition length was unexpected, especially considering that mouse Psen2 activity was recently shown to affect mitochondrial to ER tethering via inhibition of Mitofusin2 activity in MEFs [62]. However, we reasoned that, possibly, zebrafish embryos retained sufficient Presenilin protein activity from before MO injection to allow normal MAM formation. Since we had not controlled for Presenilin activity loss in our experiment we decided to modify the experiment with additional treatments and controls. MO-injected embryos were allowed to develop for 48 hours by which time the pigmentation-loss and hydrocephalus phenotypes of Presenilin loss are obvious (see Fig 2 in [55]). This allowed selection of embryos for which we were confident that MO-injection had been effective. We also expanded the treatments to include inhibition of γ-secretase activity with the compound DAPT [12], and inhibition of mitochondrial respiration (mimicking hypoxia) using sodium azide [58].

### Analysis in embryos at 48 hpf

Five treatment classes were examined; Untreated, γ-secretase inhibition (with 100 μM DAPT from 4 hpf), hypoxia mimicry (with 100 μM sodium azide from 36 hpf), Presenilin activity loss (injected with MoPS1Tln plus MoPS2Tln) and MO-injection negative control (injected with MoCont). Since exposure to hypoxia slows embryo development, the treatment group exposed to sodium azide was allowed to develop until these embryos reached a stage equivalent to that of 48 hpf embryos under normoxia (at 28.5°C–they reached this developmental stage after a delay of 6–8 hours). The effectiveness of the above treatments was confirmed by observation for developmental delay (under mimicry of hypoxia), the loss of melanotic cells (melanophores) in the trunk region (under γ-secretase inhibition), and for loss of melanophores and the occurrence of hydrocephalus (caused by Psen1 and Psen2 protein loss). Only embryos showing the appropriate phenotypes were selected for analysis.

Once again, three embryos were examined per treatment and transverse sections were taken through from the yolk extension region of the trunk. In this study, up to 6 cells were examined from each embryo to reduce the possible confounding effects of measuring M-ER apposition lengths in cells that might have different differentiation states.

The individual M-ER apposition length measurements from our analyses at 48 hpf are shown in S2 Data. All EM images used for our analyses are available in S7–S11 Files.

**Fig 5:**
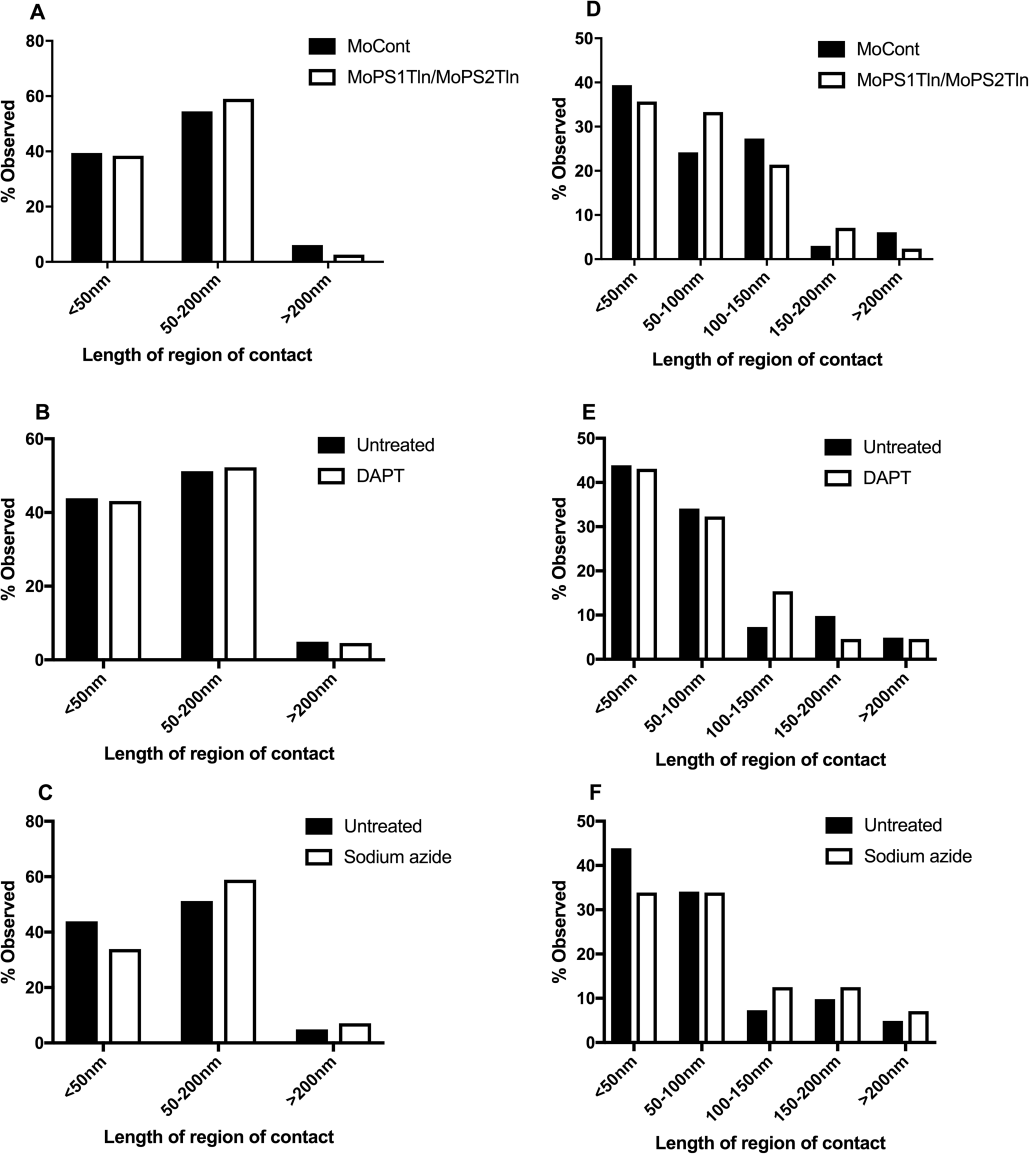
Quantification of 48 hpf zebrafish embryo M-ER apposition lengths. Percentage observed for each length class for 3 classes A-C) and 5 classes D-F). Total apposition events analysed were 33 in MoCont, 42 in MoPS1Tln/MoPS2Tln, 56 in sodium azide and 65 in DAPT -injected embryos.

As previously, we examined the M-ER appositions lengths when grouped into three and five classes of range length (Fig 5 and Table 3A and 3B). Fisher’s exact tests [60] revealed no significant differences in the distribution of M-ER apposition lengths for the various treatments compared to their controls. Total apposition events analysed in untreated embryos were 41. For 3 classes, p = 0.779 for MoPS1Tln/MoPS2Tln compared to MoCont, p = 1 for DAPT compared to untreated and p = 0.63 for sodium azide compared to untreated. For 5 classes, p = 0.74 for MoPS1Tln/MoPS2Tln compared to MoCont, p = 0.667 for DAPT compared to untreated and p = 0.844 for sodium azide compared to untreated.

**Table 3:** A) Percentage observed per length class from 48 hpf embryos, 3 classes. B) Percentage observed per length class from 48 hpf embryos, 5 classes.

**A**						
**Treatment**	**Length of region of apposition, mitochondrial perimeter (nm)**					
	**<50nm**		**50-200nm**		**>200nm**	
**MoCont injected (n = 33)**	39.4%(13)		54.5%(18)		6.1%(2)	
**MoPS1Tln/MoPS2Tln injected (n = 39)**	38.4%(15)		59%(23)		2.6%(1)	
**Untreated (n = 41)**	43.9%(18)		51.2%(21)		4.9%(2)	
**DAPT (n = 65)**	43.1%(28)		52.3%(34)		4.6%(3)	
**Sodium azide (n = 56)**	33.9%(19)		58.9%(33)		7.1%(4)	
**B**						
**Treatment**	**Length of region of apposition, mitochondrial perimeter (nm)**					
	**<50nm**	**50-100nm**		**100-150nm**	**150-200nm**	**>200nm**
**MoCont injected (n = 33)**	39.4%(13)	24.2%(8)		27.3%(9)	3.0%(1)	6.1%(2)
**MoPS1Tln/MoPS2Tln injected (n = 42)**	35.7%(15)	33.3%(14)		21.4%(9)	7.1%(3)	2.4%(1)
**Untreated (n = 41)**	43.9%(18)	34.1%(14)		7.3%(3)	9.8%(4)	4.9%(2)
**DAPT (n = 65)**	43.1%(28)	32.3%(21)		15.4%(10)	4.6%(3)	4.6%(3)
**Sodium azide (n = 56)**	33.9%(19)	33.9%(19)		12.5%(7)	12.5%(7)	7.1%(4)

## Discussion

The 2012 study by Area-Gomez et al. [32], observed an increase M-ER apposition length in MEFs lacking both *Psen1* and *Psen2* activity. Also, mimicry of hypoxia might be expected to increase M-ER apposition length as cells attempt to increase mitochondrial activity. However, Area-Gomez et al. [32] did not observe an increase in M-ER apposition when γ-secretase activity was inhibited using DAPT. If the neural cells we examined in zebrafish embryos had shown similar responses to the MEFs examined by Area-Gomez and colleagues we might have expected to see increased M-ER apposition in the embryos injected with MOs blocking Psen1 and Psen2 translation and, possibly, in the sodium azide-treated embryos but not in the DAPT-treated embryos. Unexpectedly we saw no significant differences in any treatment group.

During the development of the zebrafish spinal cord, cells divide near the apical “surface” (the midline) and terminally differentiating neurons migrate outwards (e.g. see [63]). Thus, cells near the midline are likely to be proliferative and not to have taken on a particular differentiation state. A subjective observation made during collection of the M-ER apposition data reported in this paper is that these cells typically had large nuclei relative to the cytosolic volume. While M-ER appositions occur mainly in perinuclear areas these were not very numerous in the cell type we observed. This limited the statistical power of our analysis. Unfortunately, the methods available for quantitative assessment of M-ER apposition/MAM formation are limited. While sectioning and analysis using EM allowed us, apparently, to focus on one cell type, (and this would be essential for a quantitative, *in vivo* analysis) this method is very labour intensive and expensive and limits the scale of research studies that can be performed.

In mammalian cells, the majority of apposition lengths observed in wild type cells were punctate (< 50 nm) [32] while in our study, the majority of apposition lengths were in the 50–200 nm range. The cell type examined may also be a cause of this difference. Area-Gomez et al. [32] used fibroblast cells from mice and AD patients while we examined putative neuronal progenitor cells that probably have quite different energy and substrate requirements relative to fibroblasts (see below). These differences may affect the interaction between mitochondria and the ER.

Cells can control the activity of mitochondria via release of Ca^2+^ ions from the ER [64]. Mitochondria are attracted to the Ca^2+^ ion source to form M-ER appositions [65] and they use the Ca^2+^ to support the activity of a number of enzyme systems (reviewed by [66]). However, gene expression evidence supports a relatively hypoxic nature of embryogenesis in zebrafish [58, 67] and proliferative/stem cells can be heavily reliant on glycolysis (reviewed by [68, 69]). Our research focusses on neurodegeneration, and since brain is enriched in MAM we focussed in this study on a neural cell type that we could, with reasonable confidence, identify reproducibly due to its position. However, it may be that this proliferative cell type, if already relatively reliant on anaerobic glycolysis, is insensitive to treatments that would cause significant differences in M-ER apposition in terminally differentiated cells largely dependent on oxidative phosphorylation such as neurons. It is possible that the genetic malleability of the zebrafish embryo can yet be exploited to investigate the control of M-ER apposition/MAM formation by observing a reproducibly identifiable non-neural, differentiated cell type with a high secretory load (since MAM is responsible for oxidative protein folding). Cells of the zebrafish embryo’s hatching gland are an obvious candidate but have no mammalian equivalent and so have less than optimal translational relevance.

## Conclusions

We observed no significant difference in the distribution of M-ER apposition lengths in apically located cells of the developing spinal cord in the trunk of zebrafish embryos between individuals injected with negative control morpholinos and those injected with morpholinos for simultaneous blockage of *Psen1* and *Psen2* translation at 24 and 48 hours post fertilization. Furthermore, we observed no significant difference in the distribution of M-ER apposition lengths in such cells between embryos treated with DAPT or sodium azide and in untreated embryos at 48 hours post fertilization. The inconsistency between our observations and those performed in mammalian systems may be due to differences in cell type, developmental stage and/or differences in vertebrate species. Future studies should examine other zebrafish cell types and ages.

## Supporting information

S1 Data Individual M-ER apposition length measurements for 24 hpf embryos. (XLSX)

S2 Data Individual M-ER apposition length measurements for 48 hpf embryos. (XLSX)

S1 File EM images of M-ER apposition length for MoCont injected 24 hpf embryo 1. (ZIP)

S2 File EM images of M-ER apposition length for MoCont injected 24 hpf embryo 2. (ZIP)

S3 File EM images of M-ER apposition length for MoCont injected 24 hpf embryo 3. (ZIP)

S4 File EM images of M-ER apposition length for MoPS1Tln/PS2Tln injected for 24 hpf embryo 1. (ZIP)

S5 File EM images of M-ER apposition length for MoPS1Tln/PS2Tln injected for 24 hpf embryo 2. (ZIP)

S6 File EM images of M-ER apposition length for MoPS1Tln/PS2Tln injected for 24 hpf embryo 3. (ZIP)

S7 File EM images of M-ER apposition length for MoCont injected 48 hpf embryos. (ZIP)

S8 File EM images of M-ER apposition length for MoPS1Tln/PS2Tln injected 48 hpf embryos. (ZIP)

S9 File EM images of M-ER apposition length for untreated 48 hpf embryos. (ZIP)

S10 File EM images of M-ER apposition length for DAPT treatment of 48 hpf embryos. (ZIP)

S11 File EM images of M-ER apposition length for sodium azide treatment of 48 hpf embryos. (ZIP)
