# Supplementary figures and images for: Mitochondrion to endoplasmic reticulum apposition length in zebrafish embryo spinal progenitors is unchanged in response to perturbations associated with Alzheimer’s disease (part 1 of 2)
Source: PLoS One. 2017 Jun 21;12(6):e0179859. doi: 10.1371/journal.pone.0179859 (PMC5479591; doi:10.1371/journal.pone.0179859)

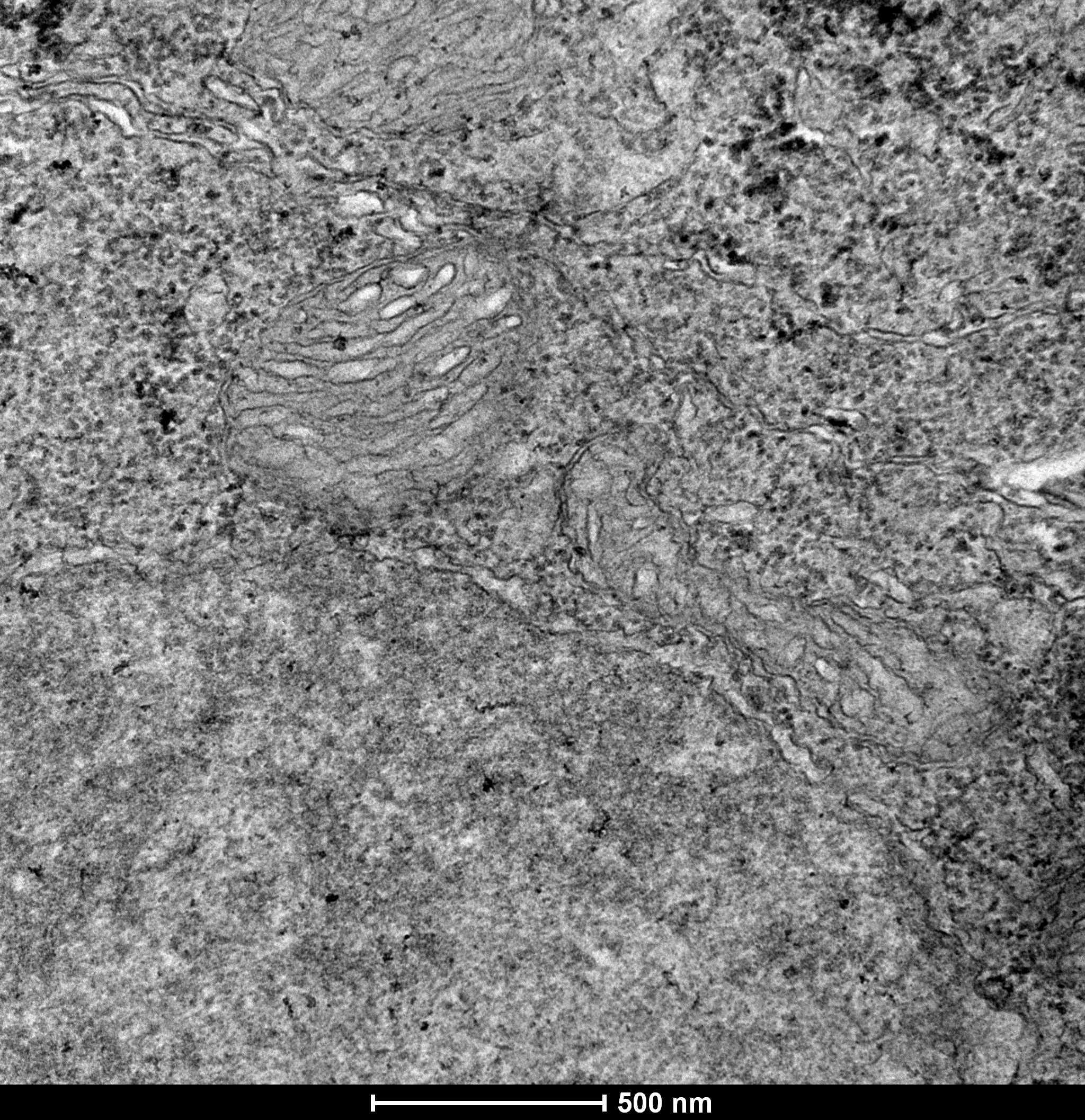

Supplement: S1 File — (ZIP) [file pone.0179859.s003.zip › Supplementary Images 2A1/section 1 embryo 1 cell 1 image 1 .tif]

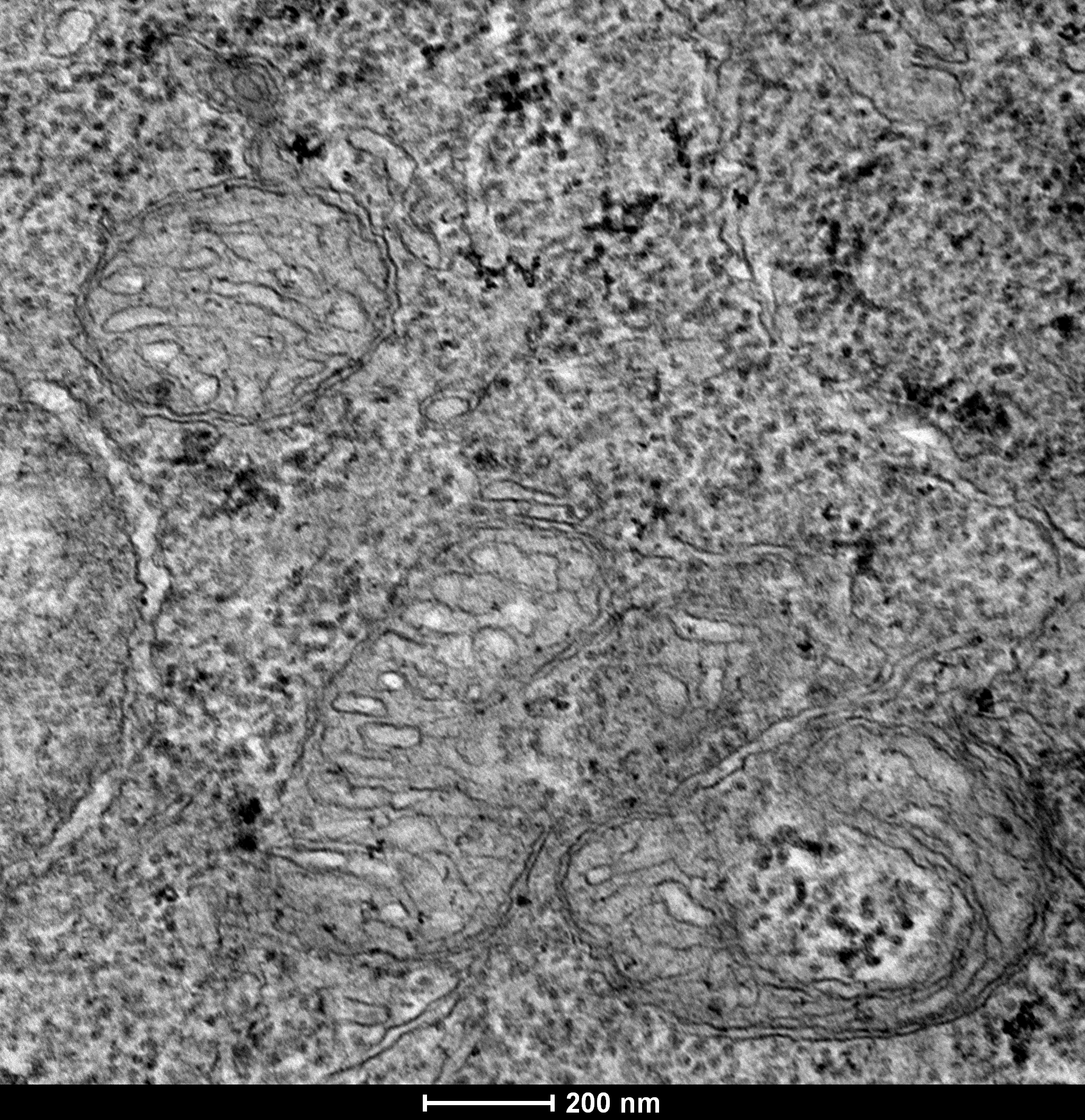

Supplement: S1 File — (ZIP) [file pone.0179859.s003.zip › Supplementary Images 2A1/section 1 embryo 1 cell 3 image 1.1 .tif]

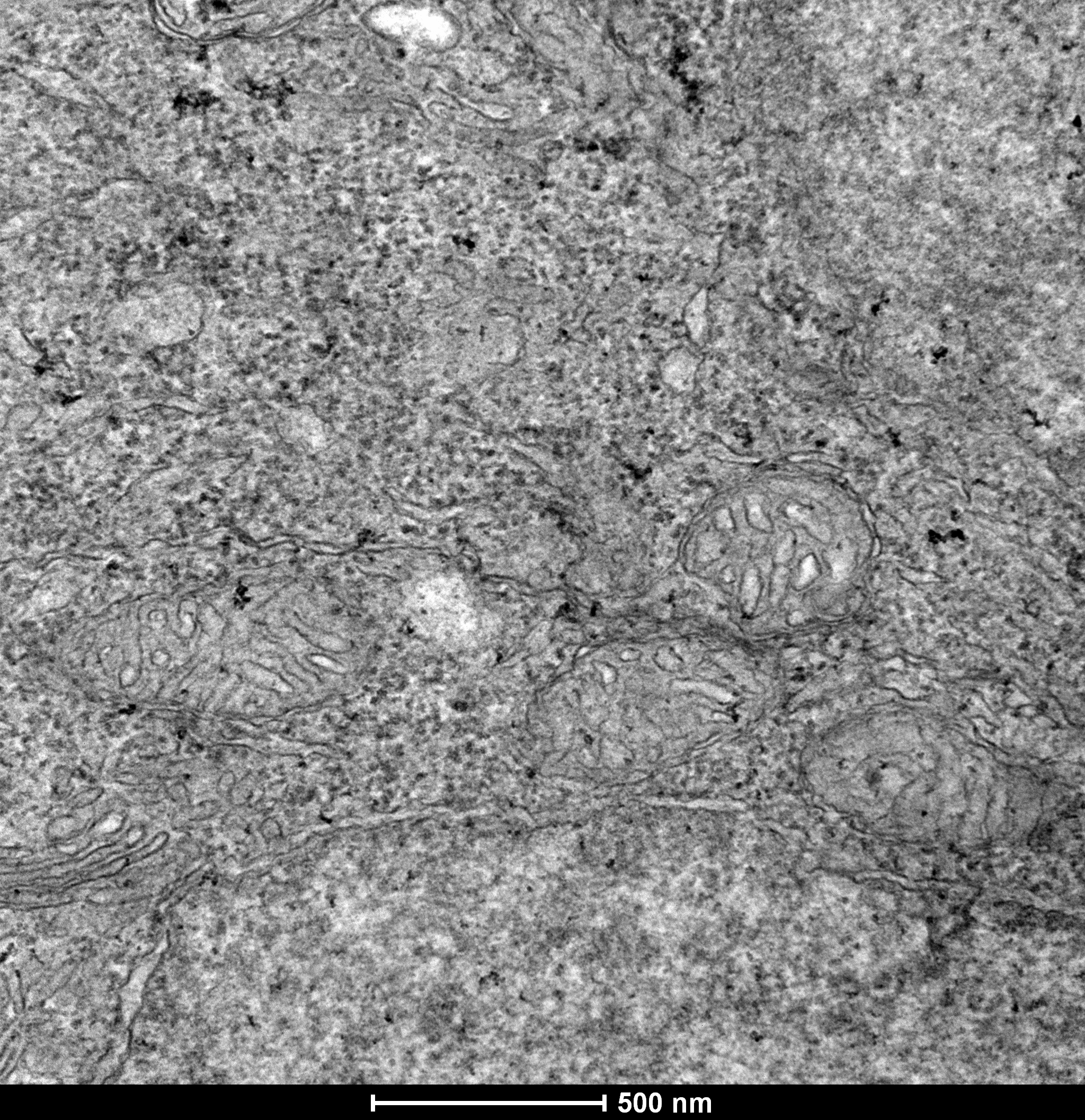

Supplement: S1 File — (ZIP) [file pone.0179859.s003.zip › Supplementary Images 2A1/section 1 embryo 1 cell 3 image 2 43000x .tif]

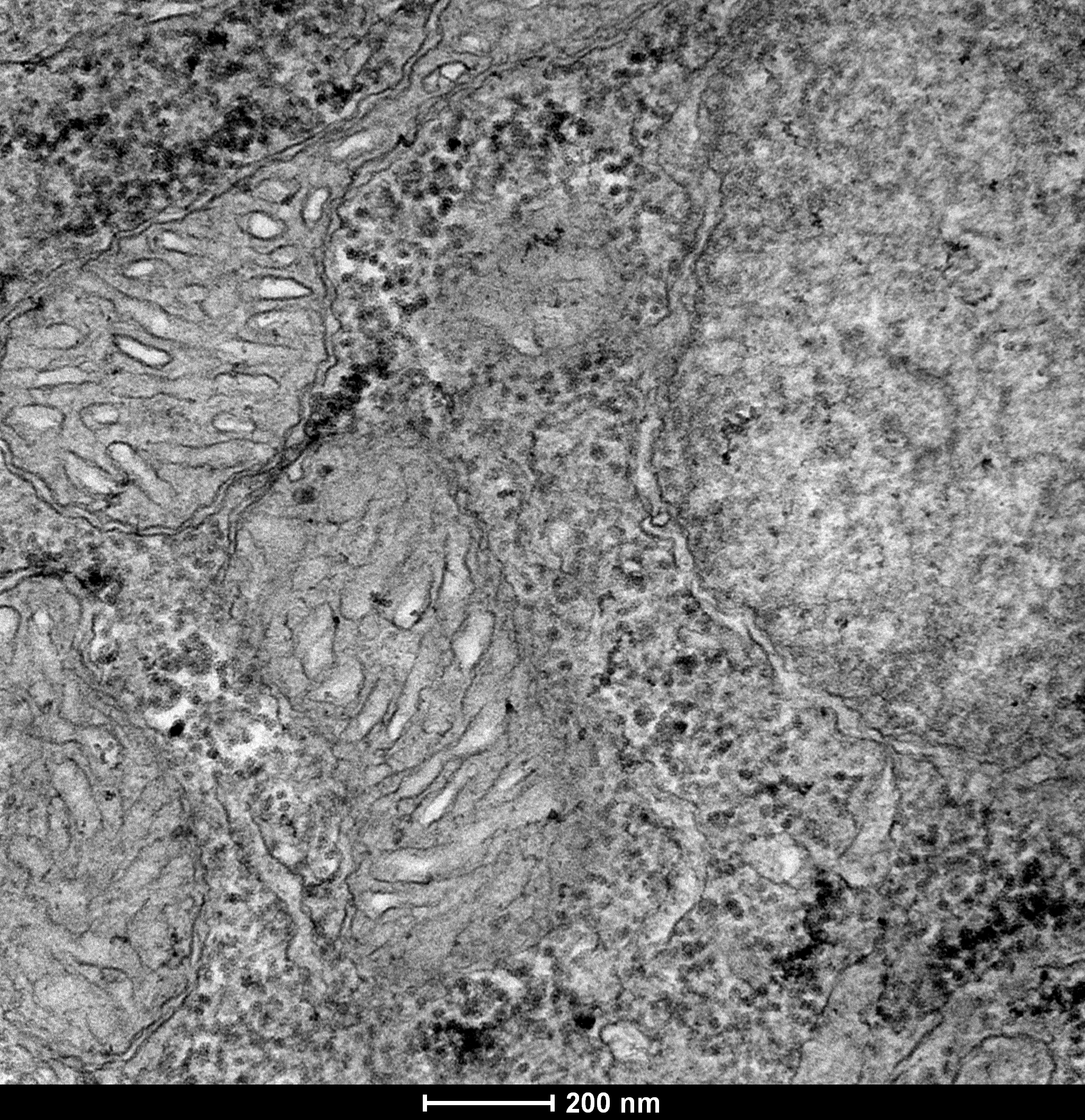

Supplement: S1 File — (ZIP) [file pone.0179859.s003.zip › Supplementary Images 2A1/section 2 embryo 1 cell 2 image 1.1 60000x.tif]

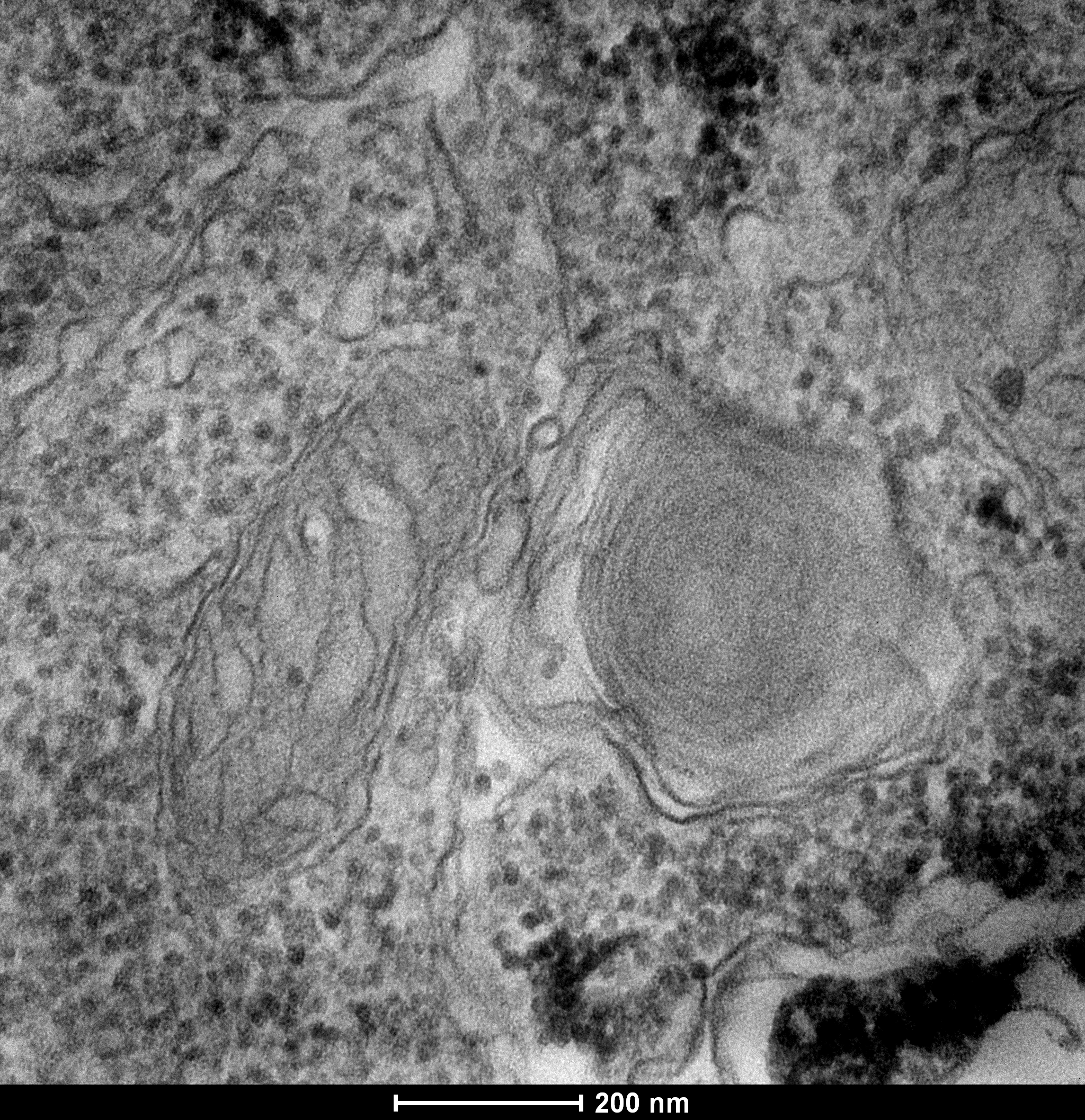

Supplement: S2 File — (ZIP) [file pone.0179859.s004.zip › Supplementary Images 2A2/embryo 2 cell 1 image 1.0 87000x .tif]

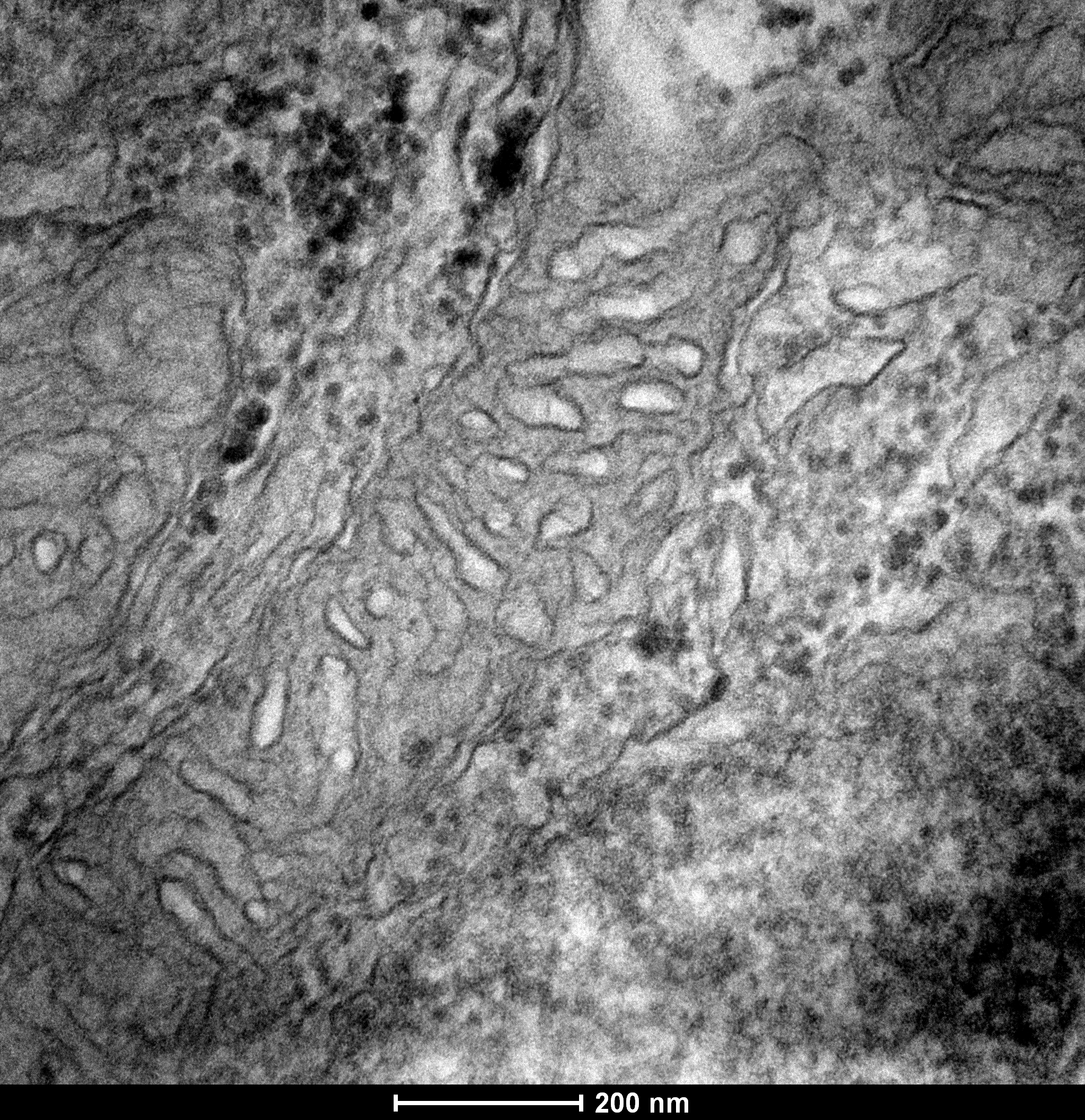

Supplement: S2 File — (ZIP) [file pone.0179859.s004.zip › Supplementary Images 2A2/embryo 2 cell 2 image 1.1 87000x right most mito .tif]

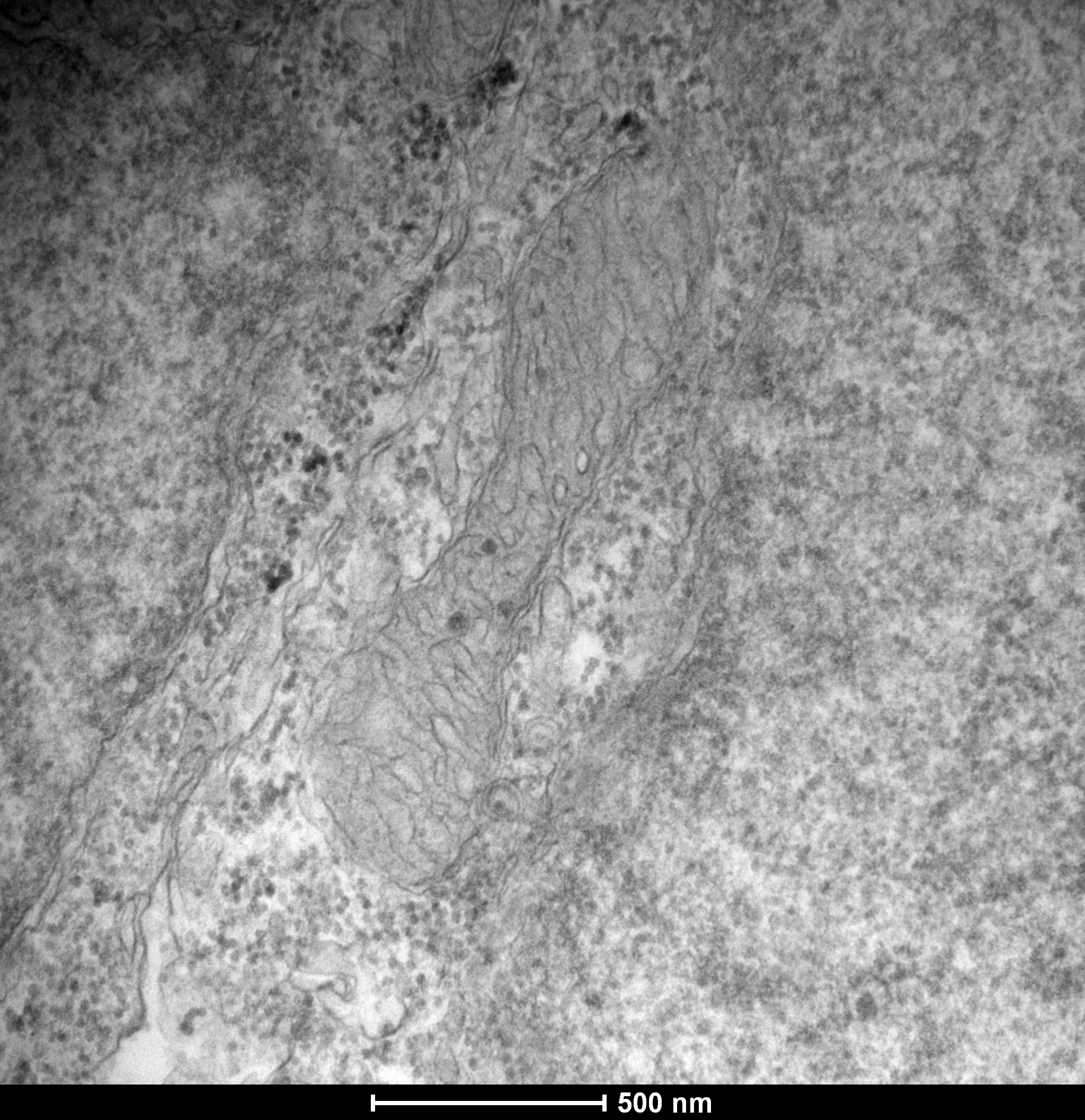

Supplement: S2 File — (ZIP) [file pone.0179859.s004.zip › Supplementary Images 2A2/embryo 2 cell 4 image 1.0 43000x .tif]

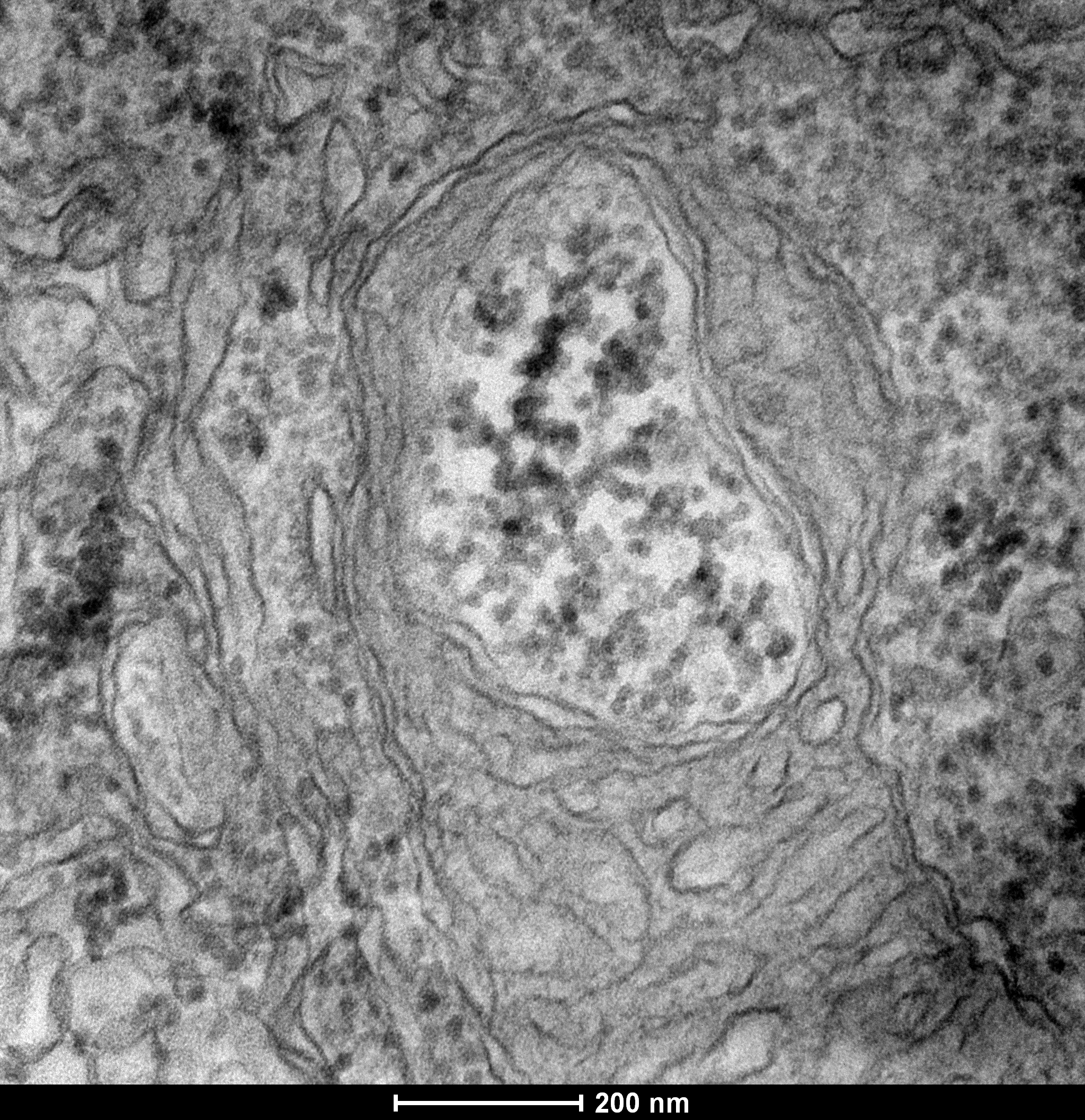

Supplement: S2 File — (ZIP) [file pone.0179859.s004.zip › Supplementary Images 2A2/embryo 2 cell 5 image 2.1 87000x .tif]

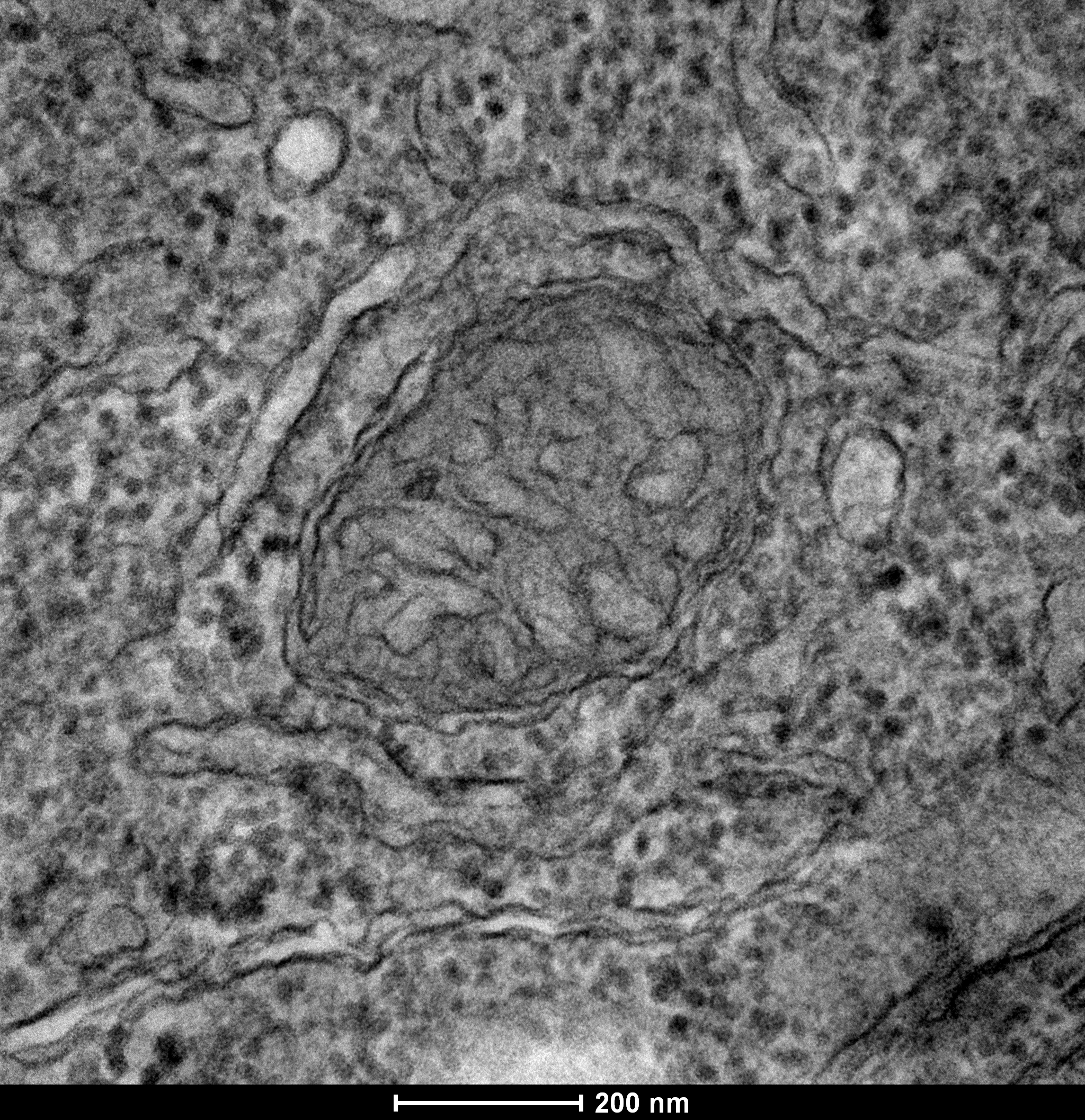

Supplement: S3 File — (ZIP) [file pone.0179859.s005.zip › Supplementary Images 2A3/embryo 3 cell 3 image 1.2 87000x .tif]

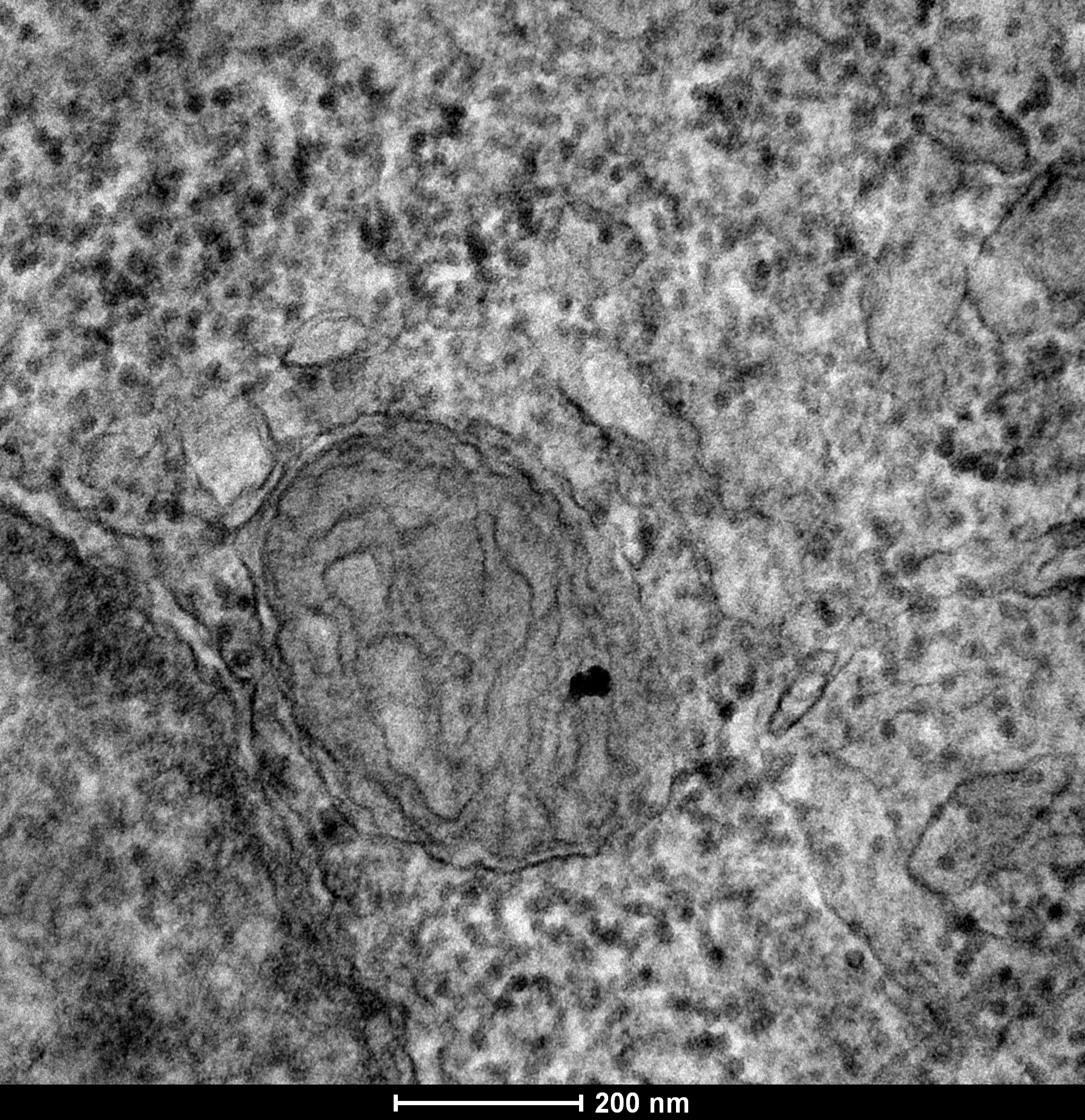

Supplement: S3 File — (ZIP) [file pone.0179859.s005.zip › Supplementary Images 2A3/embryo 3 cell 3 image 2 87000x .tif]

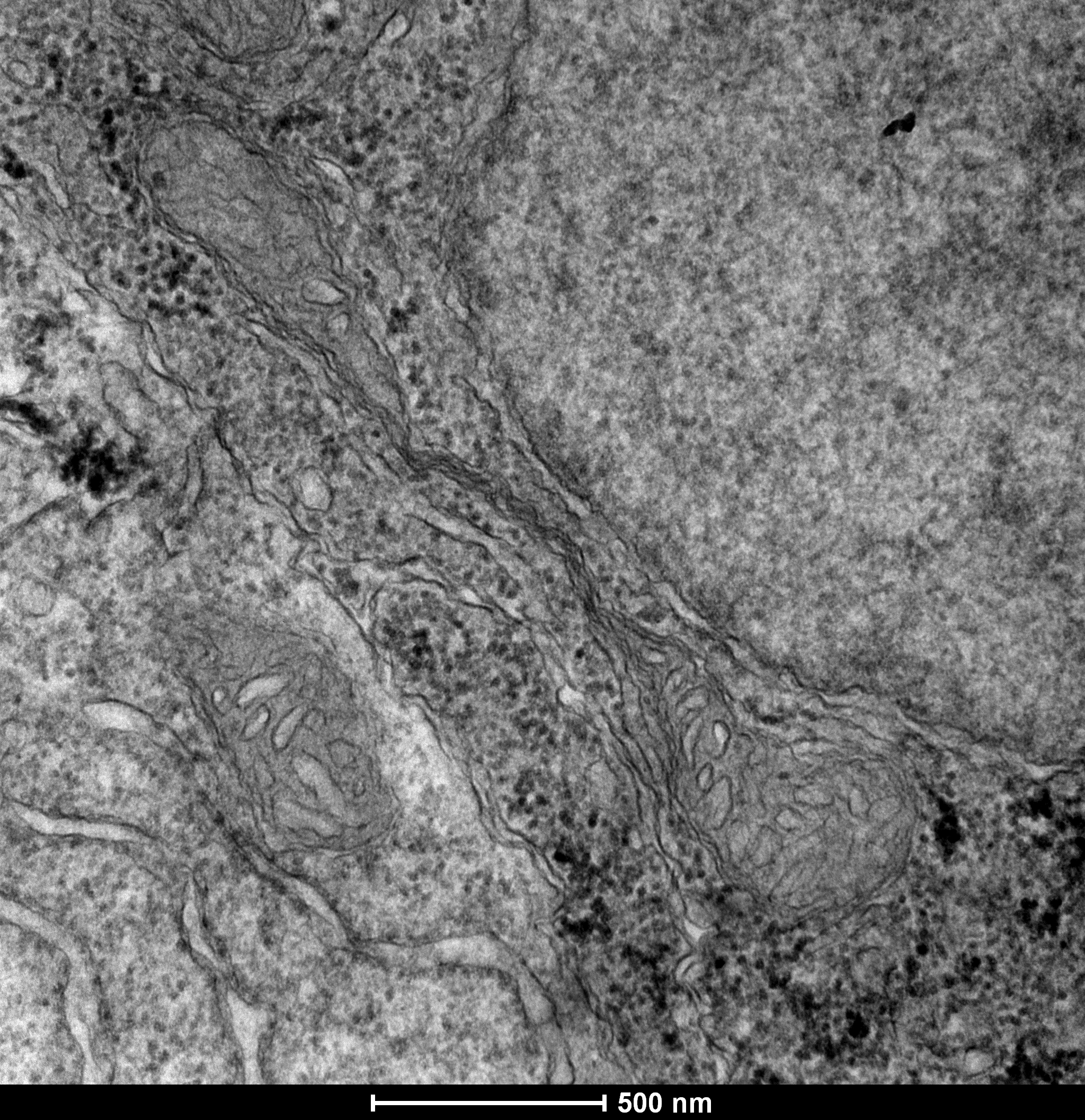

Supplement: S3 File — (ZIP) [file pone.0179859.s005.zip › Supplementary Images 2A3/embryo 3 cell 4 image 1.0 43000x .tif]

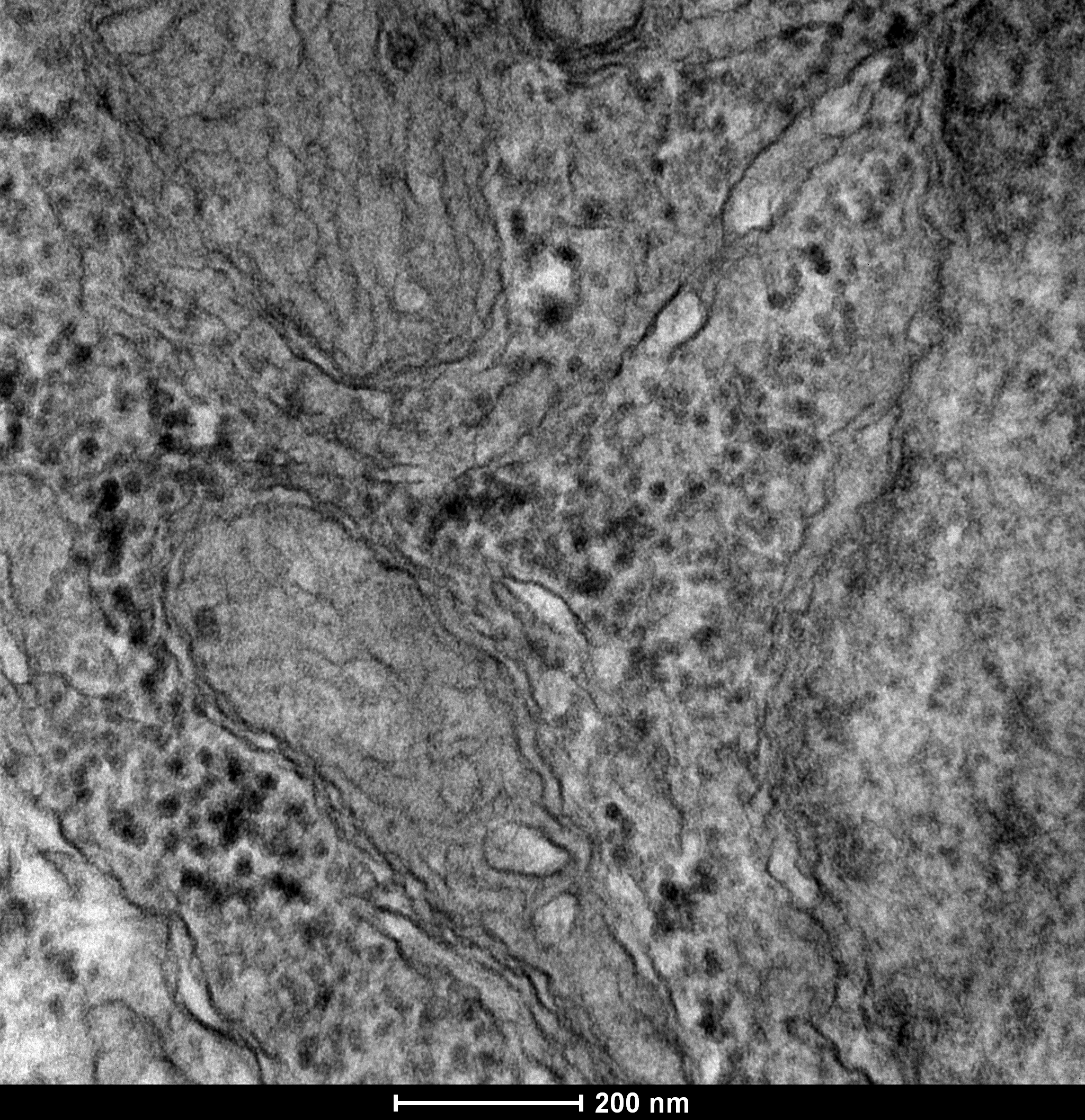

Supplement: S3 File — (ZIP) [file pone.0179859.s005.zip › Supplementary Images 2A3/embryo 3 cell 4 image 1.1 87000x .tif]

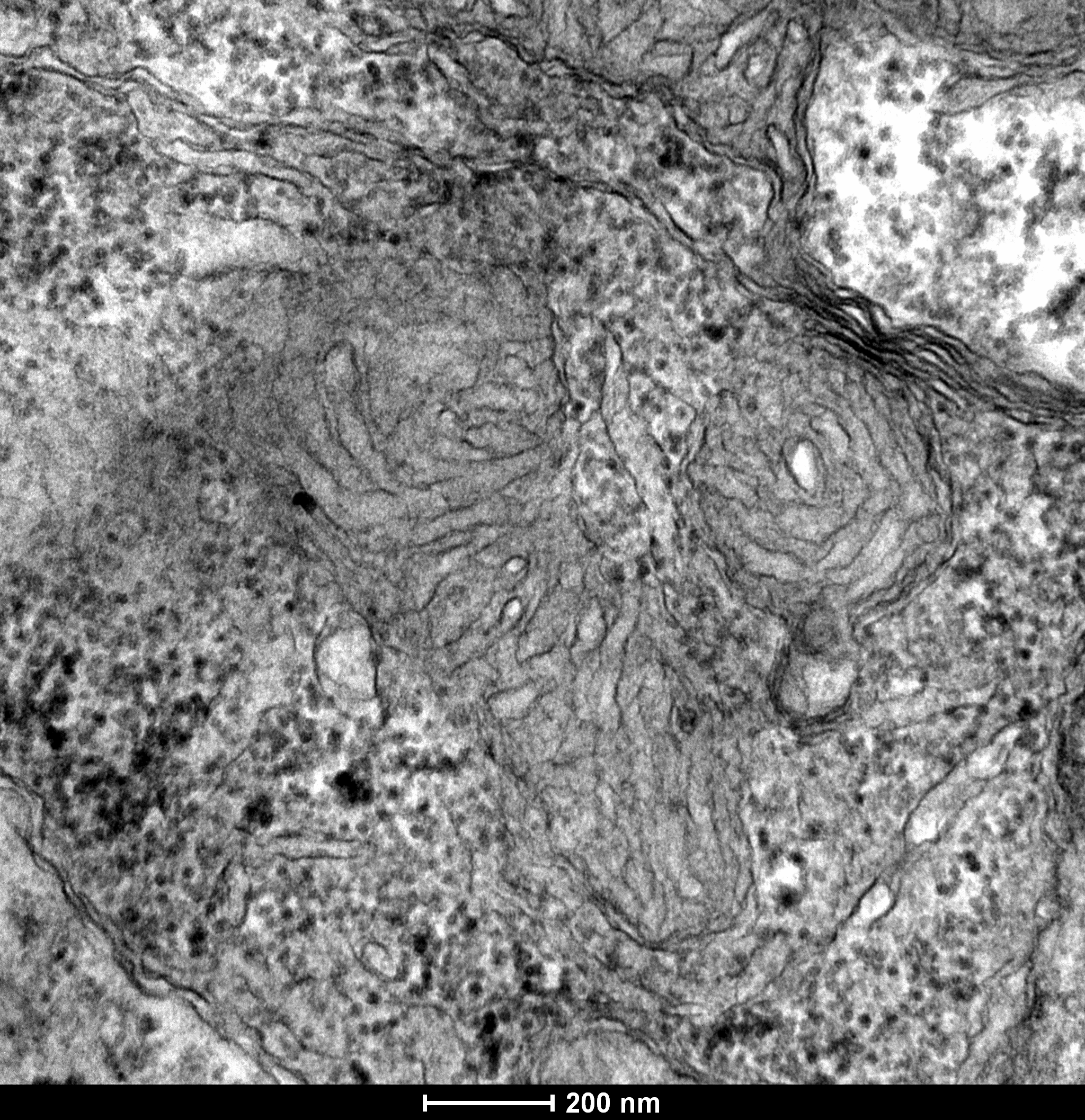

Supplement: S3 File — (ZIP) [file pone.0179859.s005.zip › Supplementary Images 2A3/embryo 3 cell 4 image 2 60000x .tif]

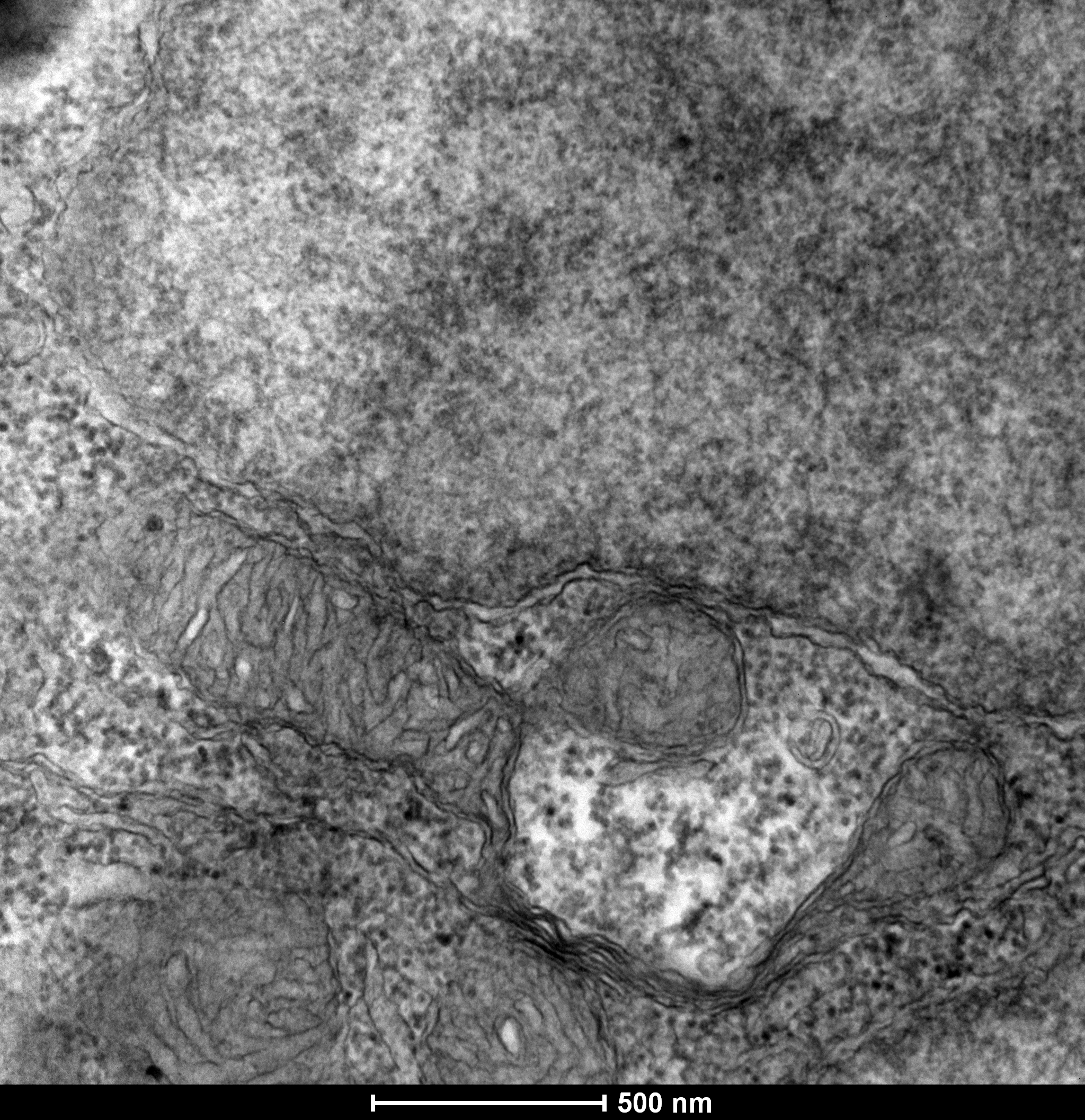

Supplement: S3 File — (ZIP) [file pone.0179859.s005.zip › Supplementary Images 2A3/embryo 3 cell 5 image 1.0 43000x .tif]

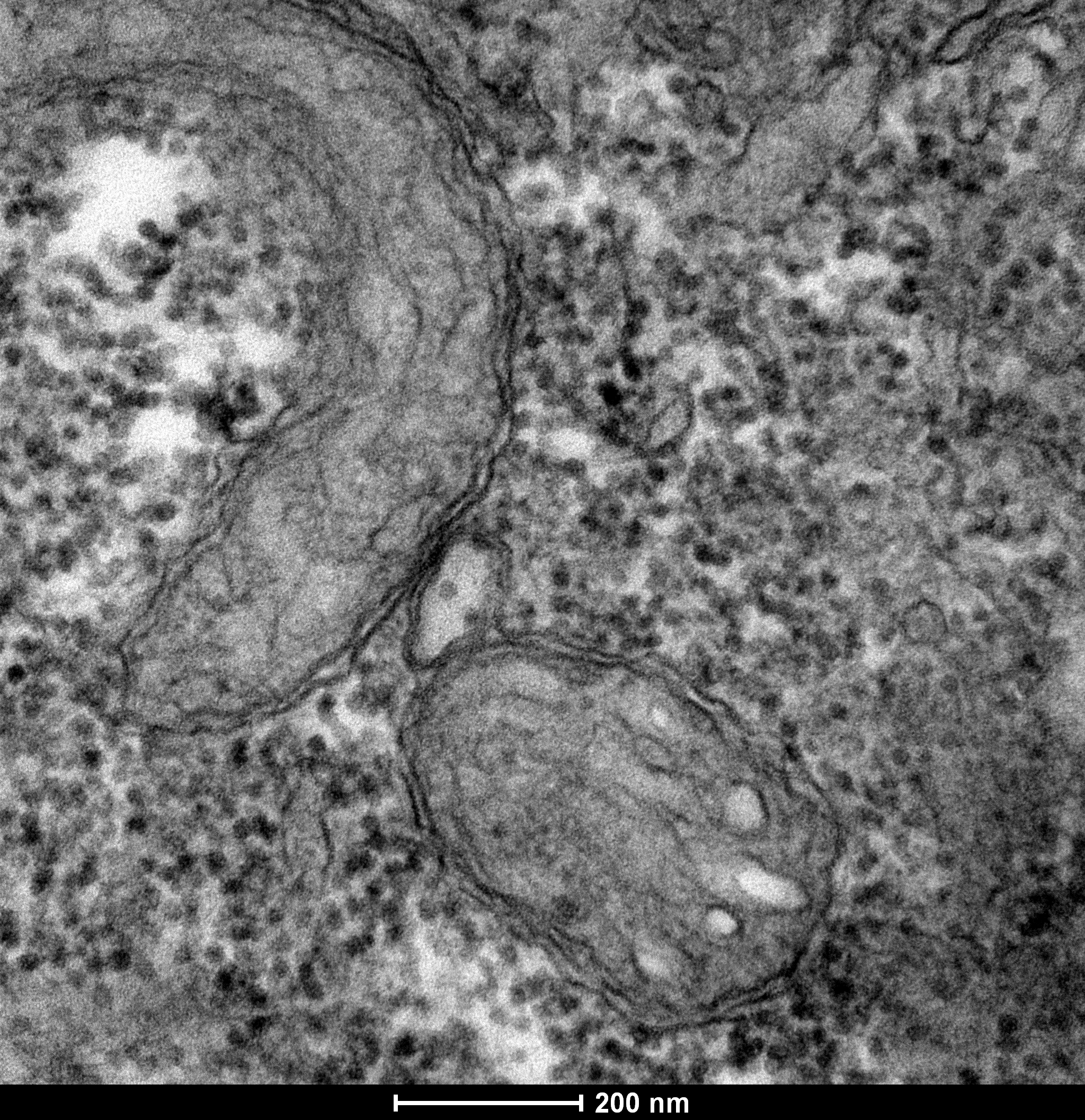

Supplement: S4 File — (ZIP) [file pone.0179859.s006.zip › Supplementary Images 2B1/section 1 embryo B1 cell 1 image 1.1 87000x .tif]

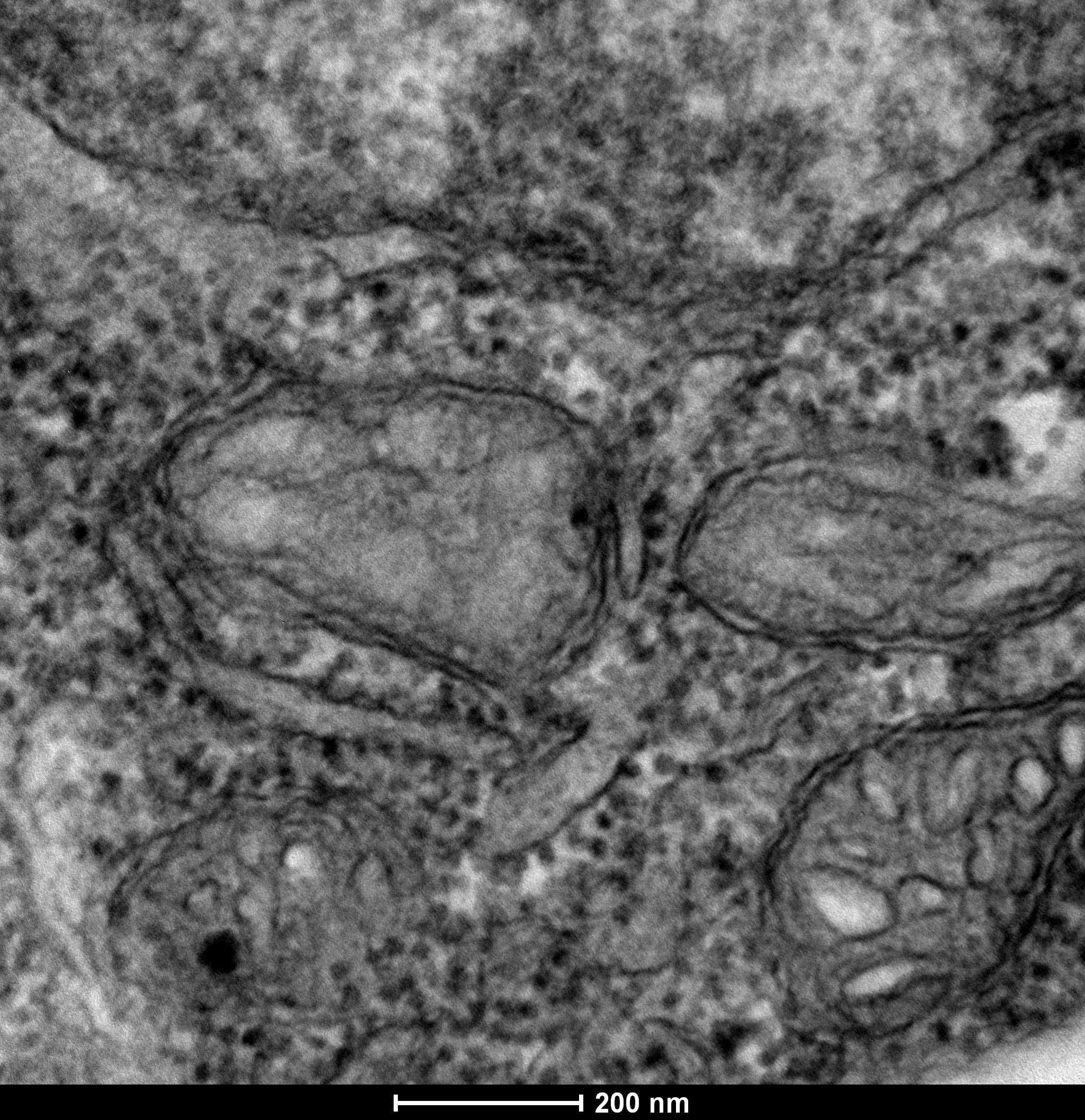

Supplement: S4 File — (ZIP) [file pone.0179859.s006.zip › Supplementary Images 2B1/section 1 embryo B1 cell 2 image 1 87000x .tif]

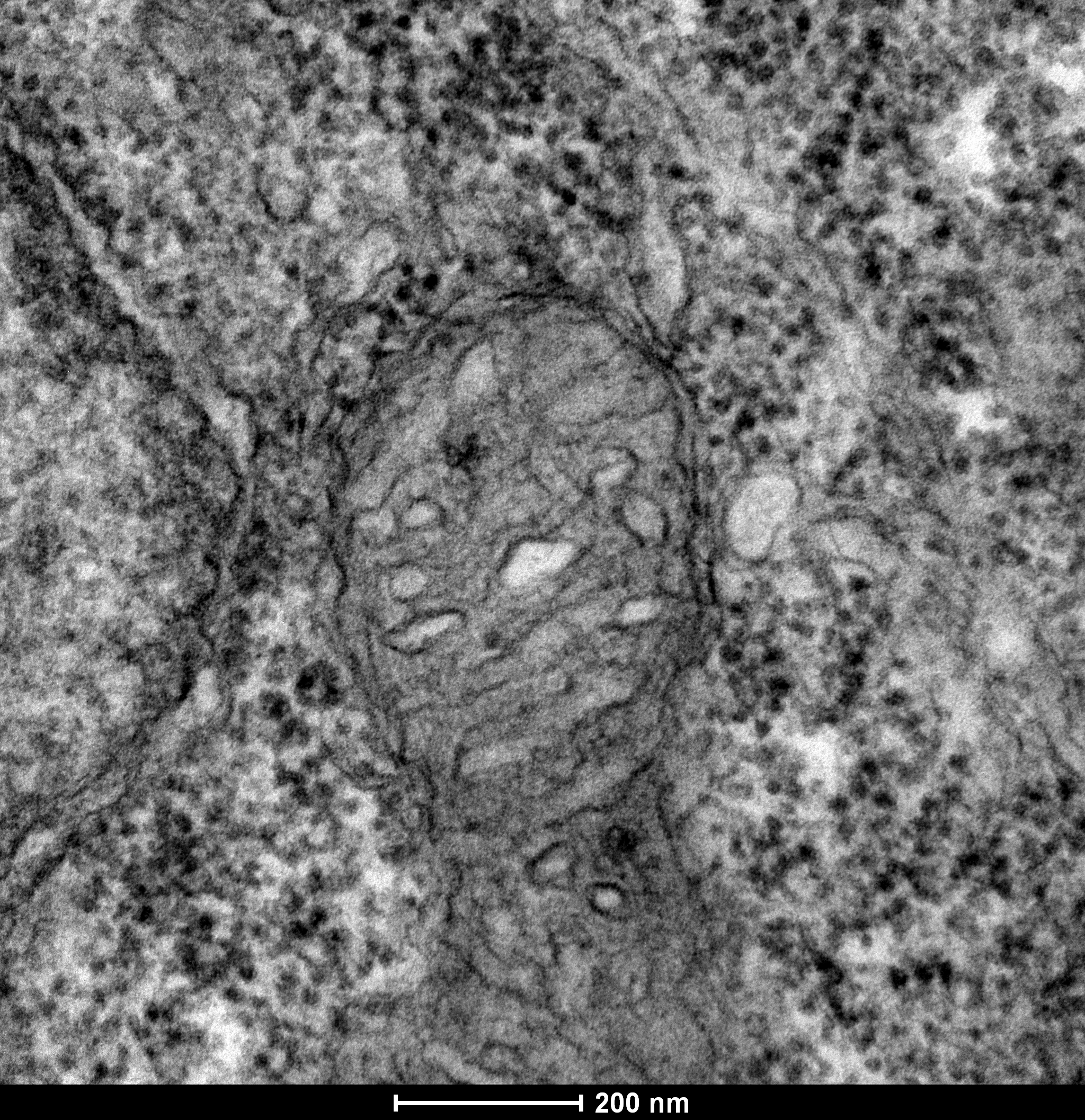

Supplement: S4 File — (ZIP) [file pone.0179859.s006.zip › Supplementary Images 2B1/section 1 embryo B1 cell 4 image 1 87000x .tif]

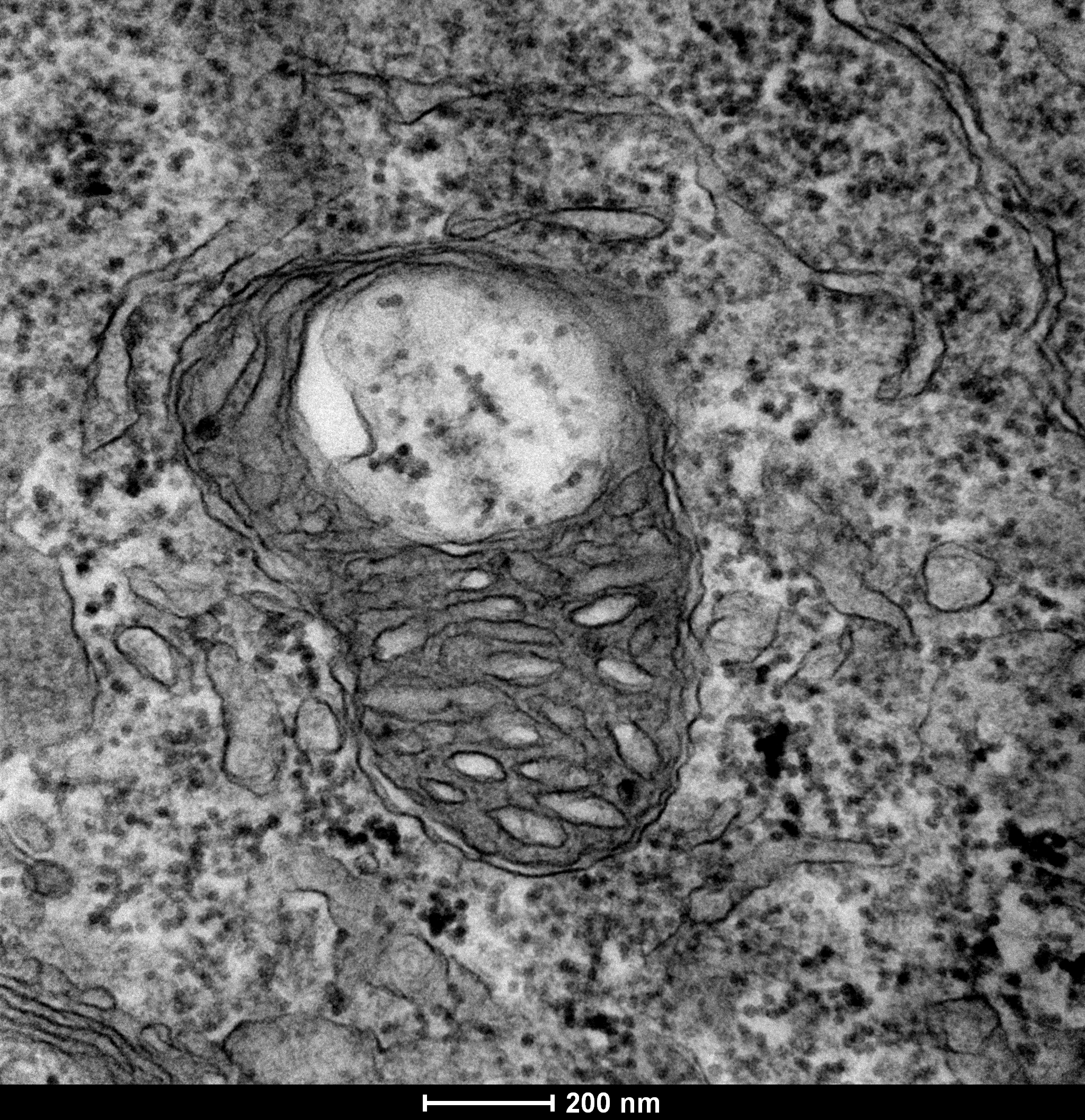

Supplement: S5 File — (ZIP) [file pone.0179859.s007.zip › Supplementary Images 2B2/embryo 2 cell 1 image 1.1 60000x .tif]

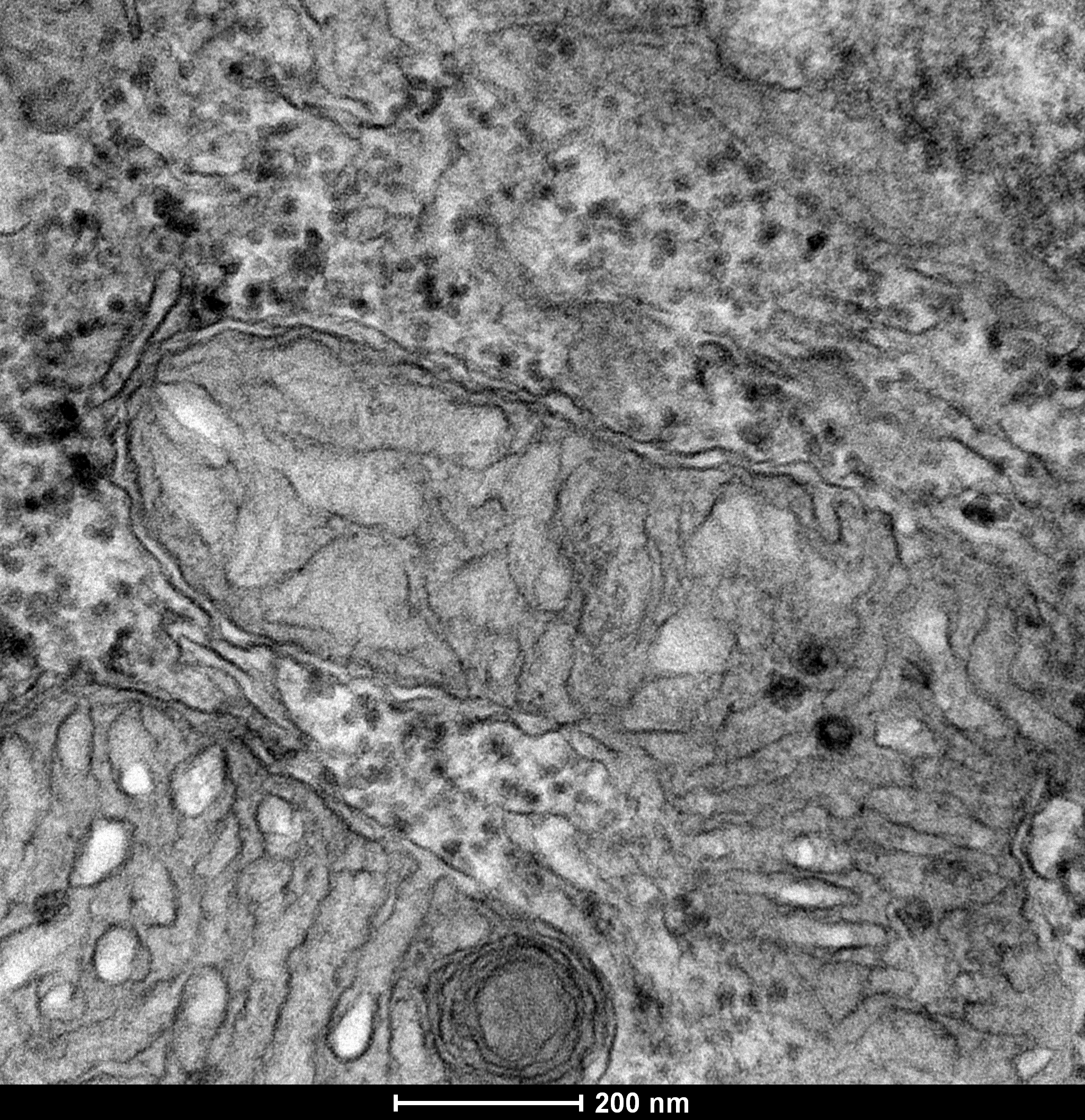

Supplement: S5 File — (ZIP) [file pone.0179859.s007.zip › Supplementary Images 2B2/embryo 2 cell 3 image 1.1 87000x .tif]

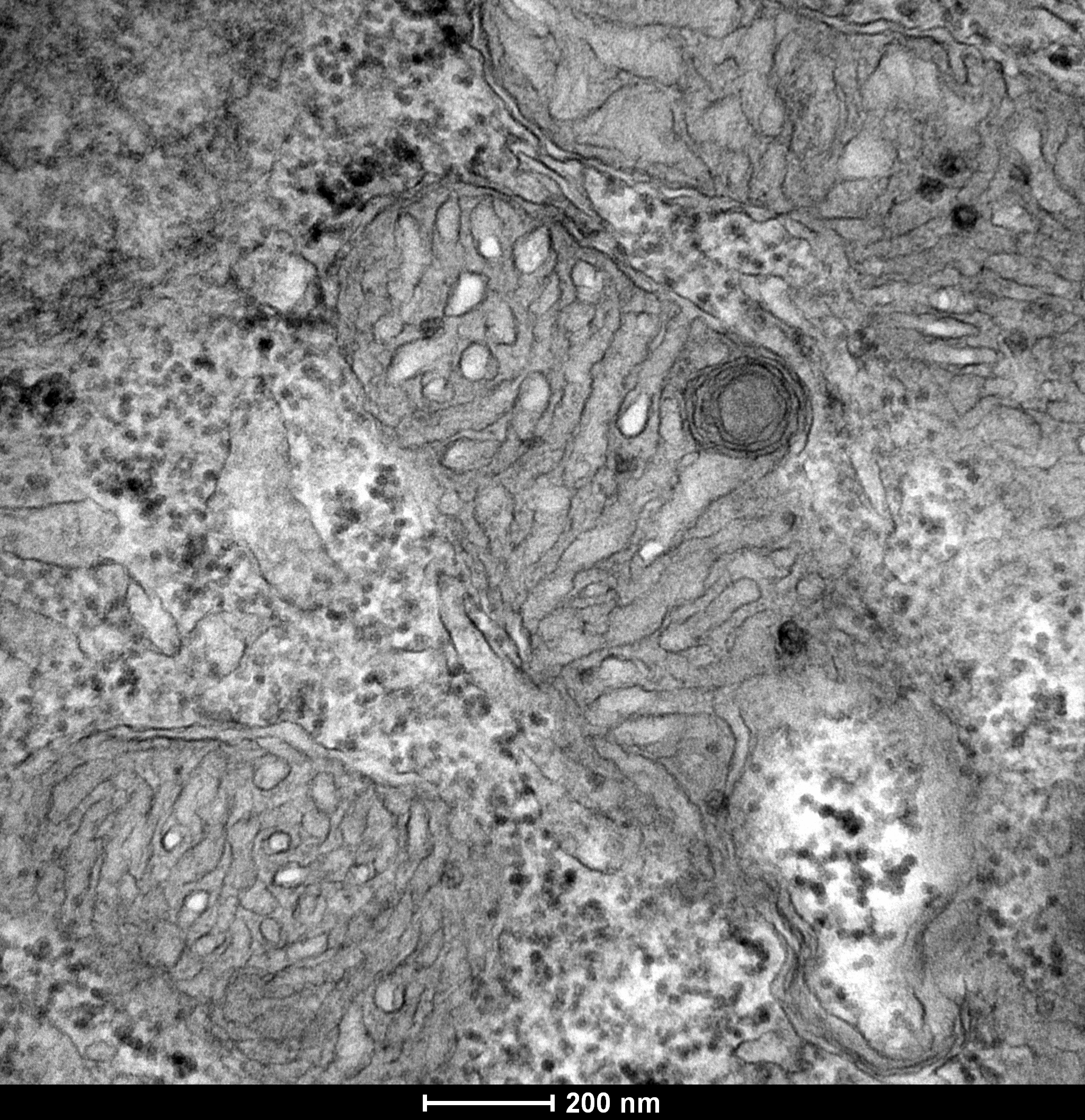

Supplement: S5 File — (ZIP) [file pone.0179859.s007.zip › Supplementary Images 2B2/embryo 2 cell 3 image 1.2 60000x .tif]

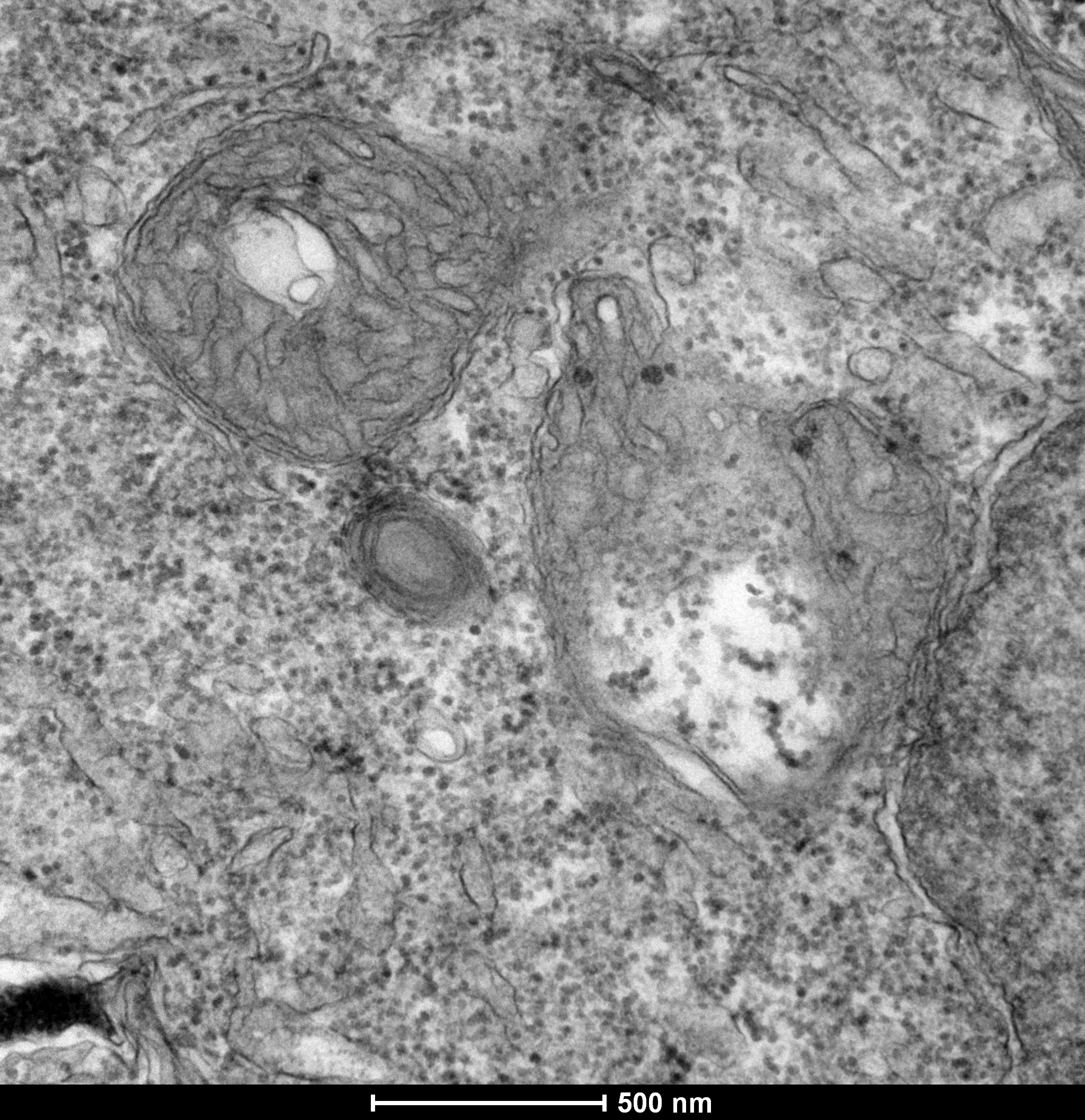

Supplement: S5 File — (ZIP) [file pone.0179859.s007.zip › Supplementary Images 2B2/embryo 2 cell 4 image 1.0 43000x .tif]

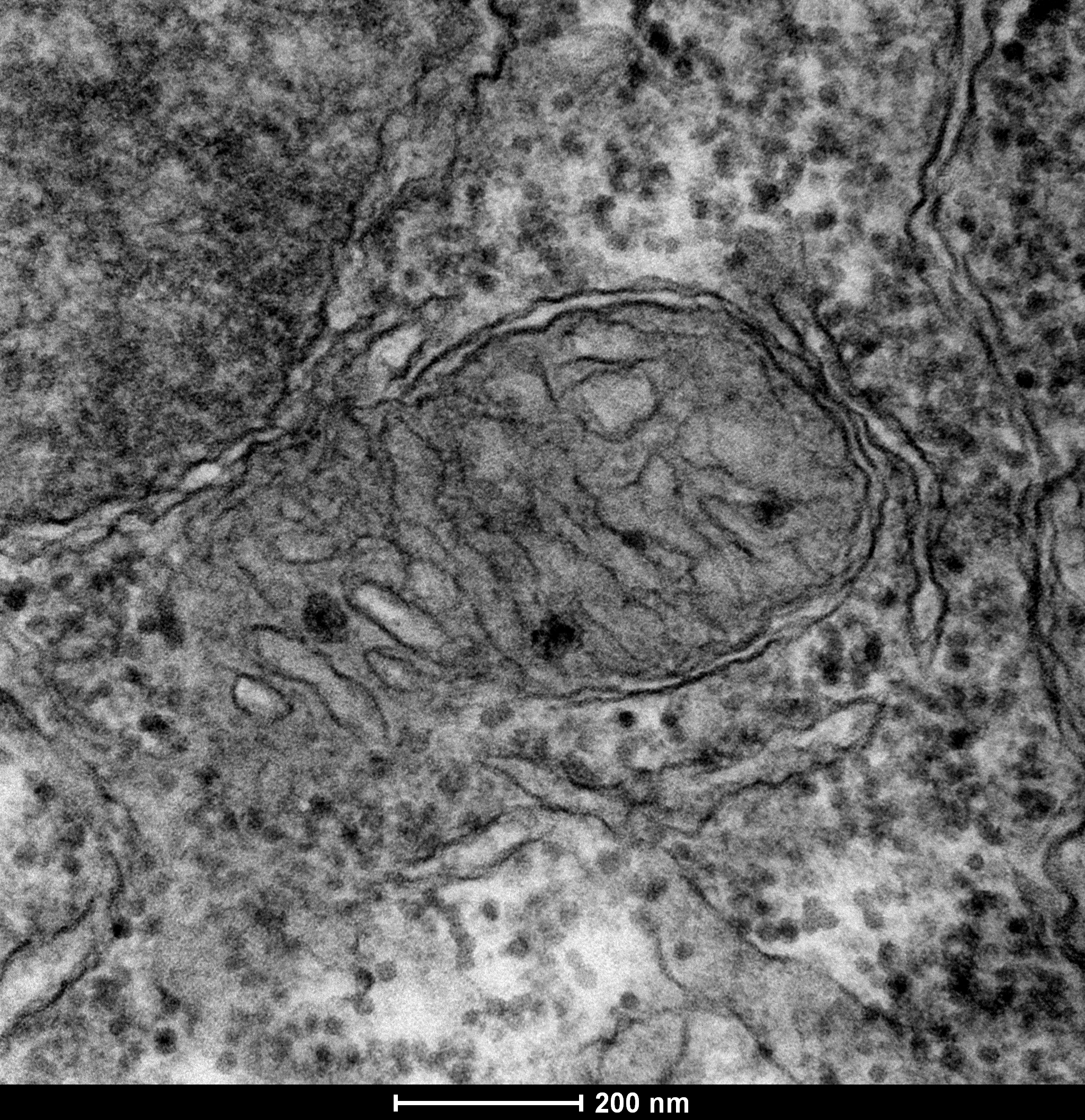

Supplement: S5 File — (ZIP) [file pone.0179859.s007.zip › Supplementary Images 2B2/embryo 2 cell 4 image 2 87000x .tif]

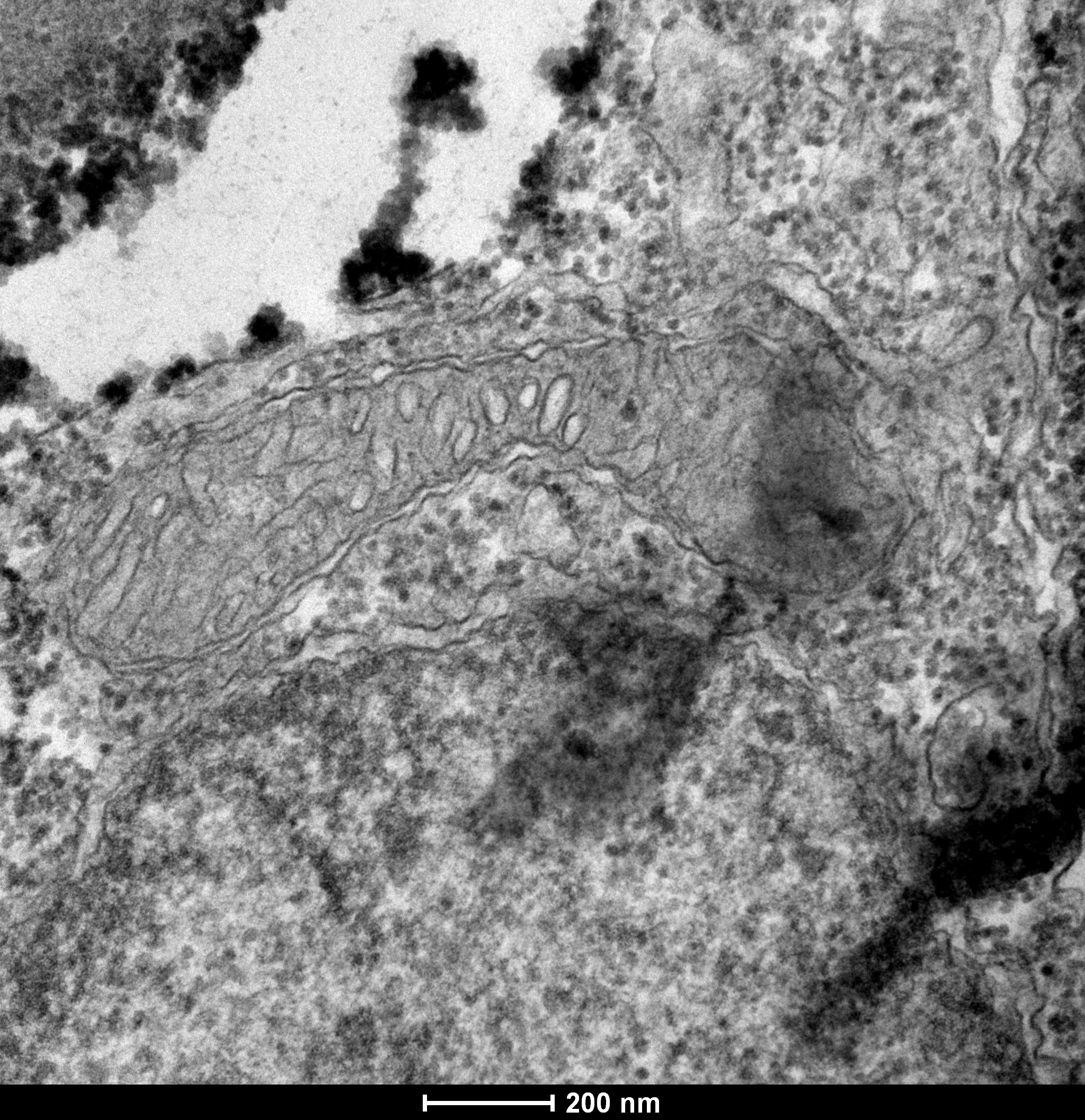

Supplement: S6 File — (ZIP) [file pone.0179859.s008.zip › Supplementary Images 2B3/embryo 3 cell 1 image 1.0 60000x .tif]

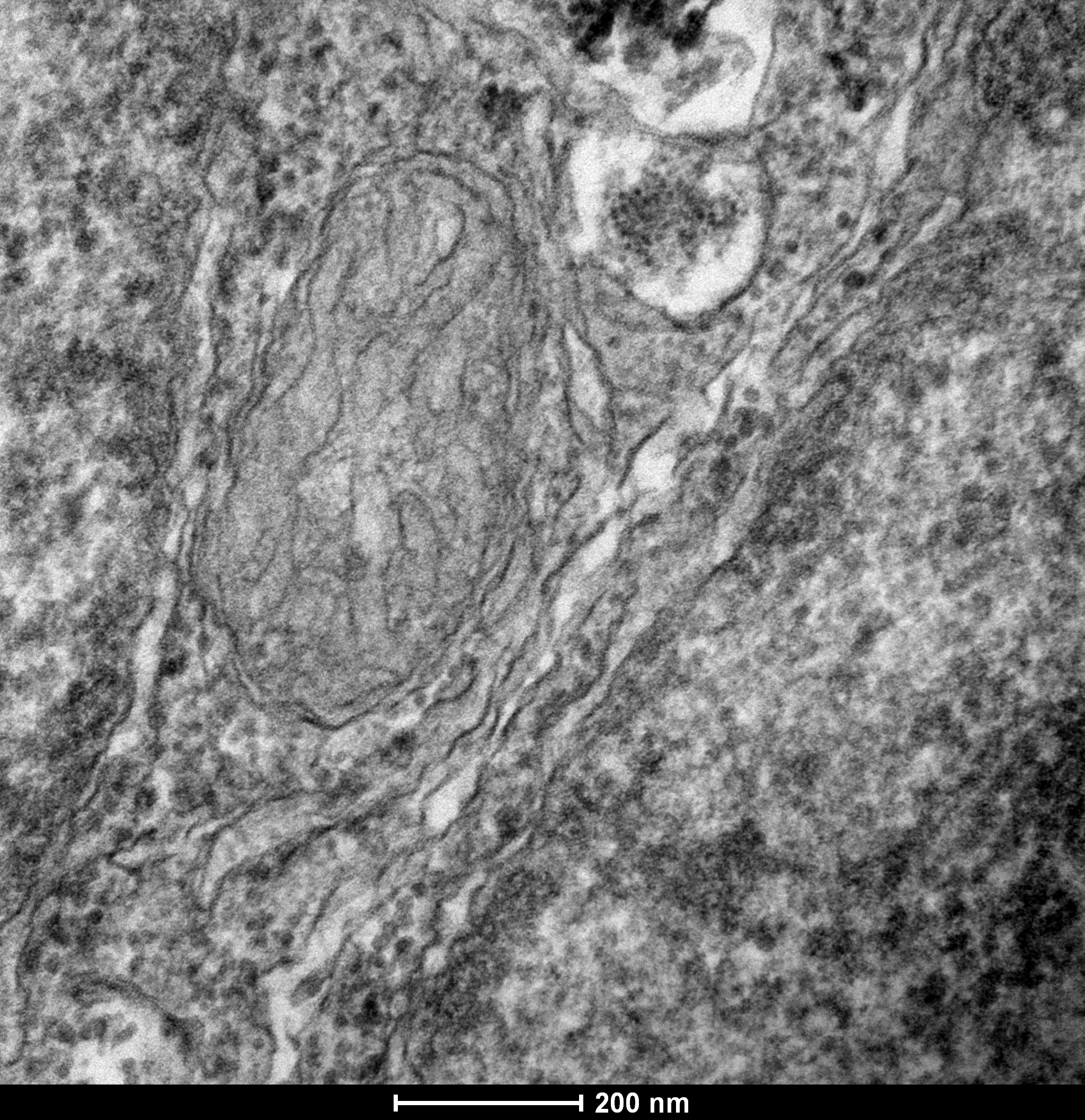

Supplement: S6 File — (ZIP) [file pone.0179859.s008.zip › Supplementary Images 2B3/embryo 3 cell 4 image 1.0 87000x .tif]

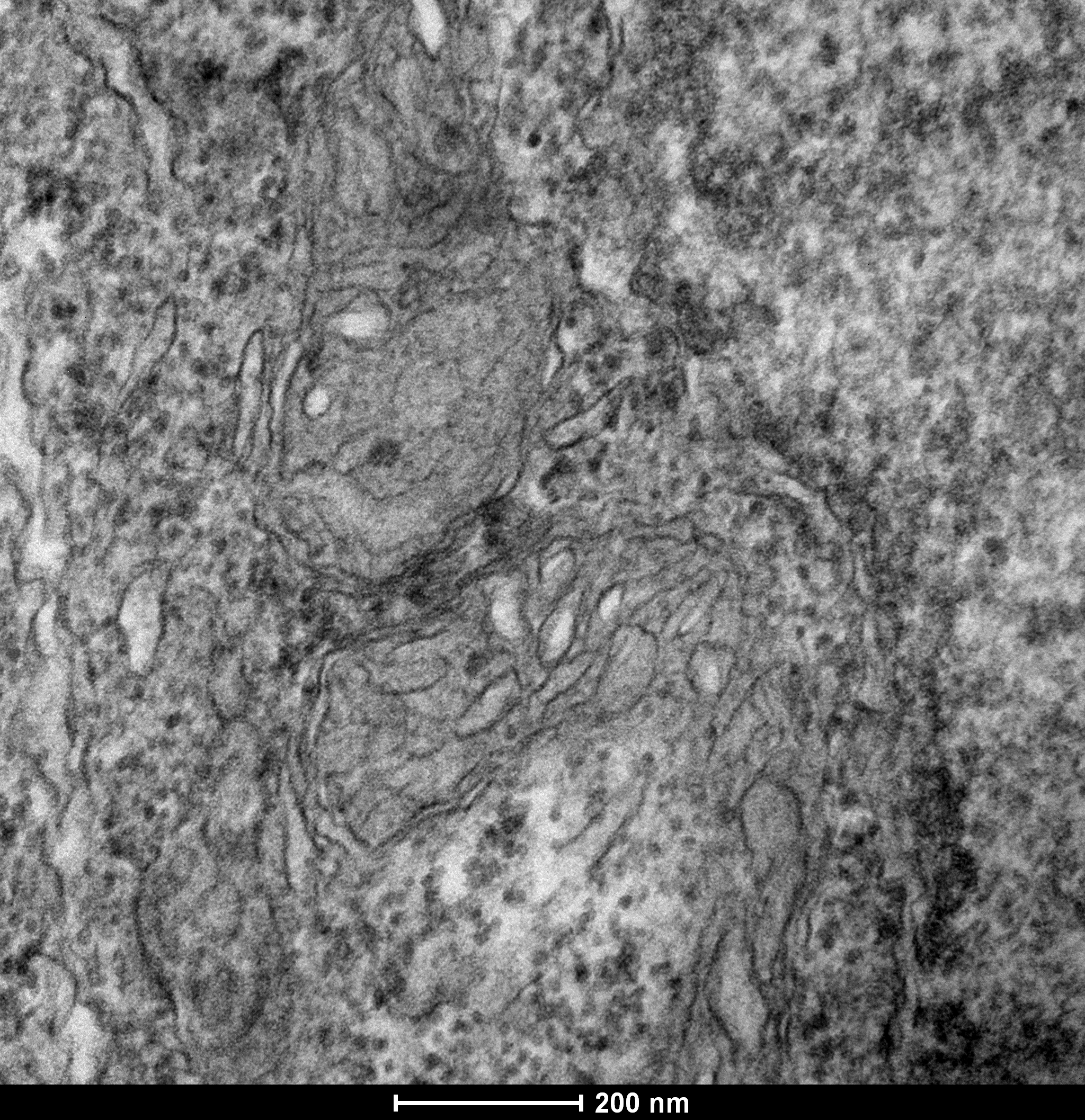

Supplement: S6 File — (ZIP) [file pone.0179859.s008.zip › Supplementary Images 2B3/embryo 3 cell 5 image 1.0 87000x .tif]

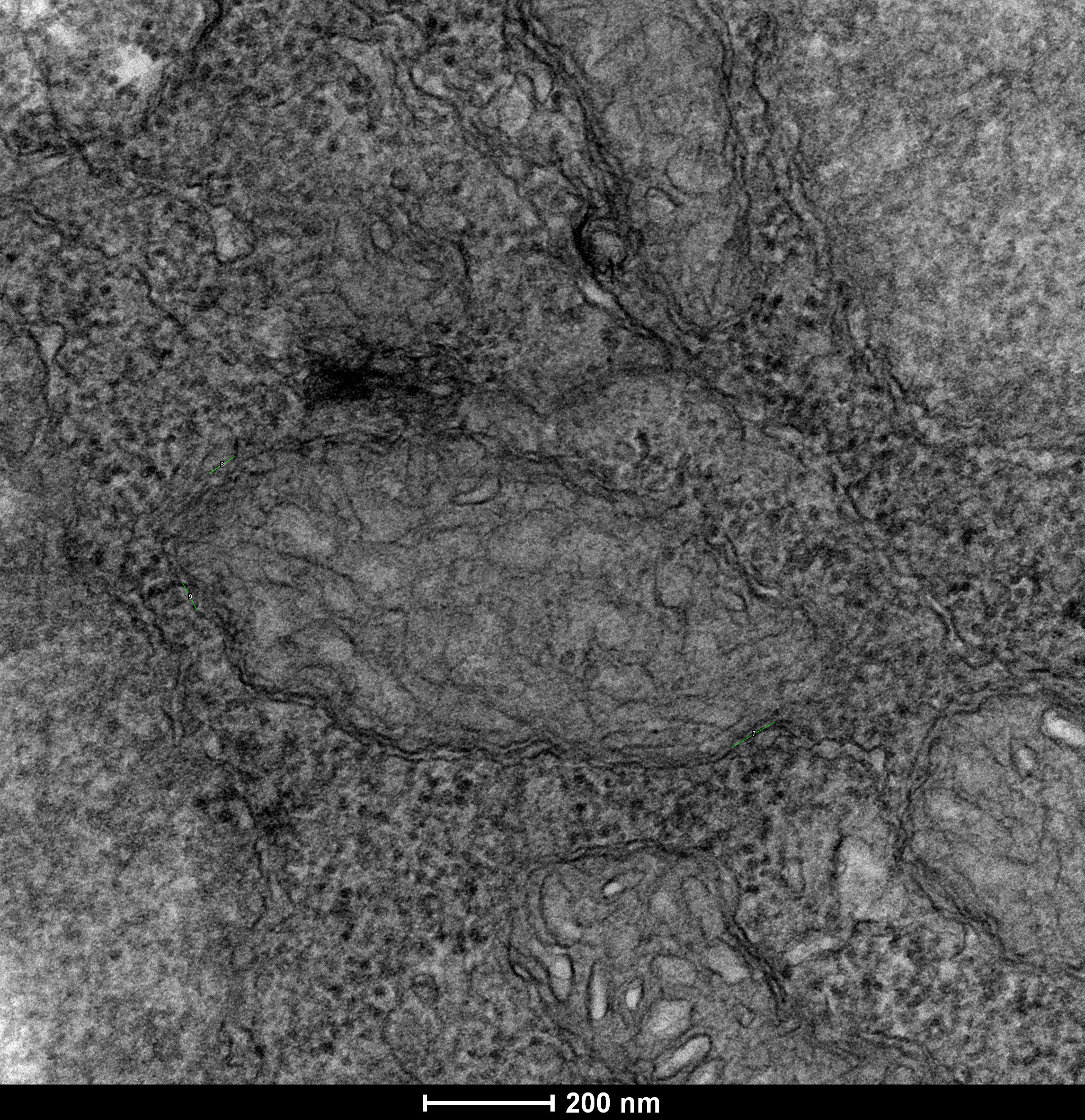

Supplement: S7 File — (ZIP) [file pone.0179859.s009.zip › Supplementary Images 4B/2a_L1_60000x_c1_m1 .jpg]

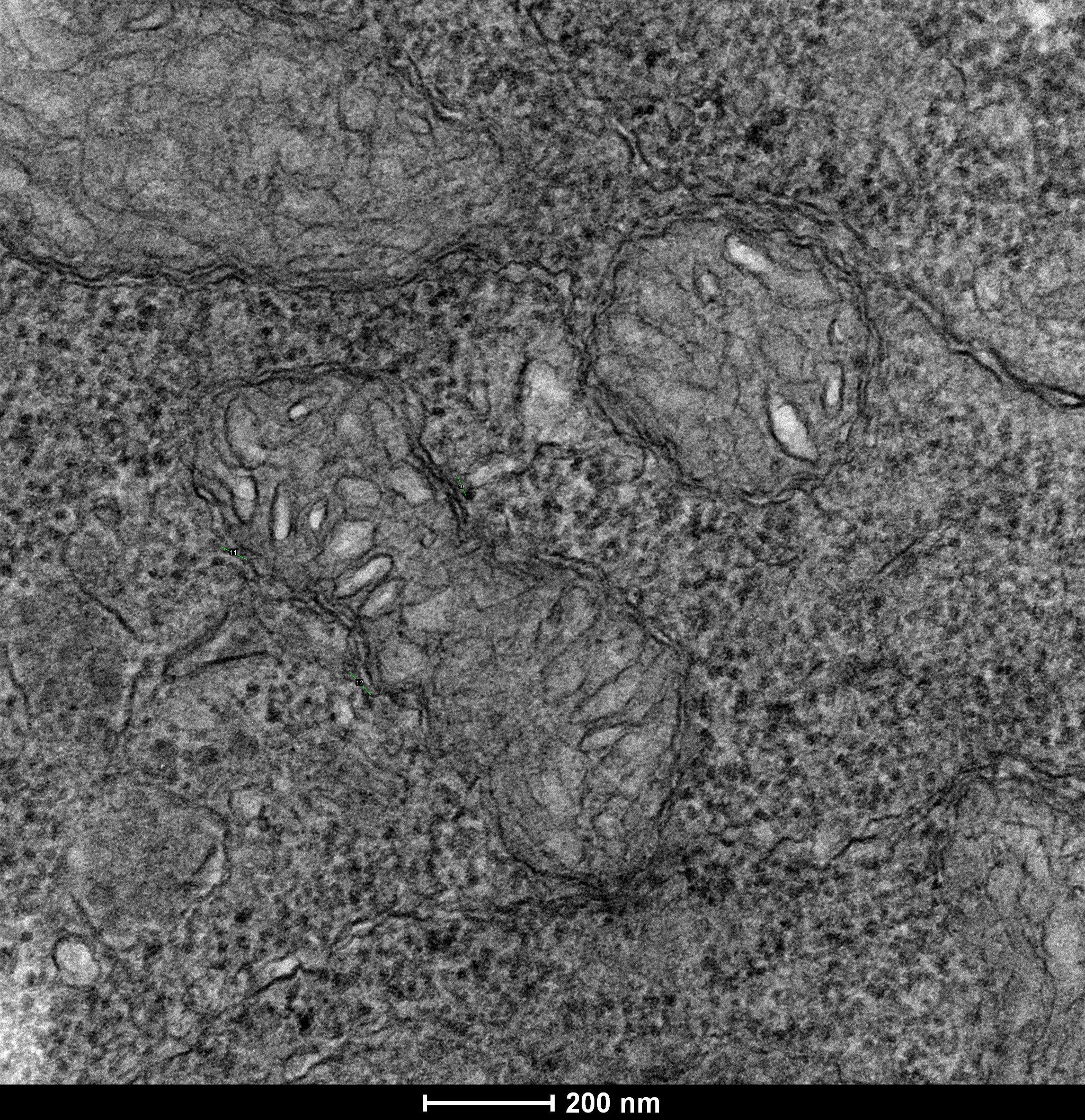

Supplement: S7 File — (ZIP) [file pone.0179859.s009.zip › Supplementary Images 4B/2a_L1_60000x_c1_m2.jpg]

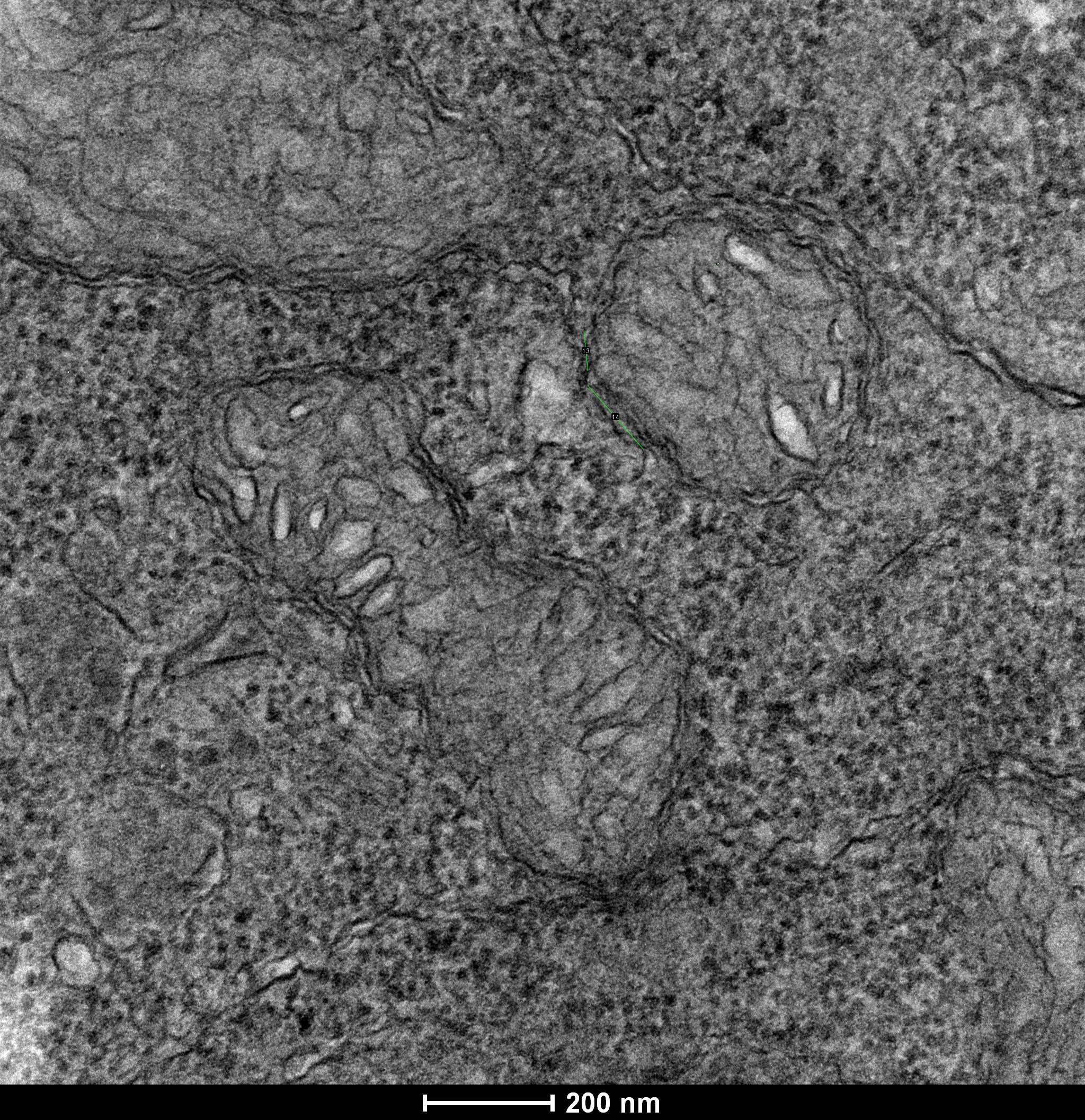

Supplement: S7 File — (ZIP) [file pone.0179859.s009.zip › Supplementary Images 4B/2a_L1_60000x_c1_m3.jpg]

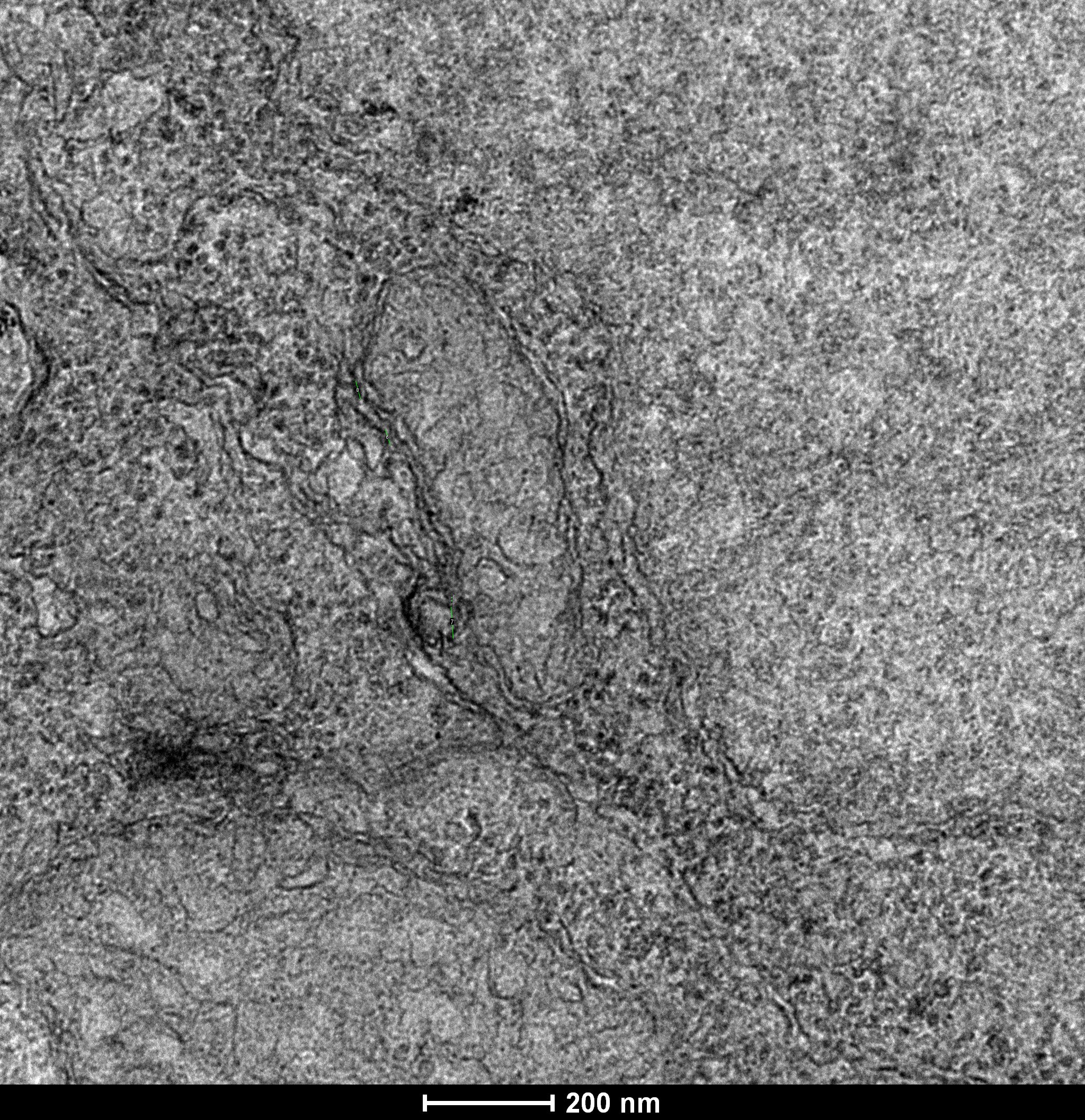

Supplement: S7 File — (ZIP) [file pone.0179859.s009.zip › Supplementary Images 4B/2a_L1_60000x_c4_m1 .jpg]

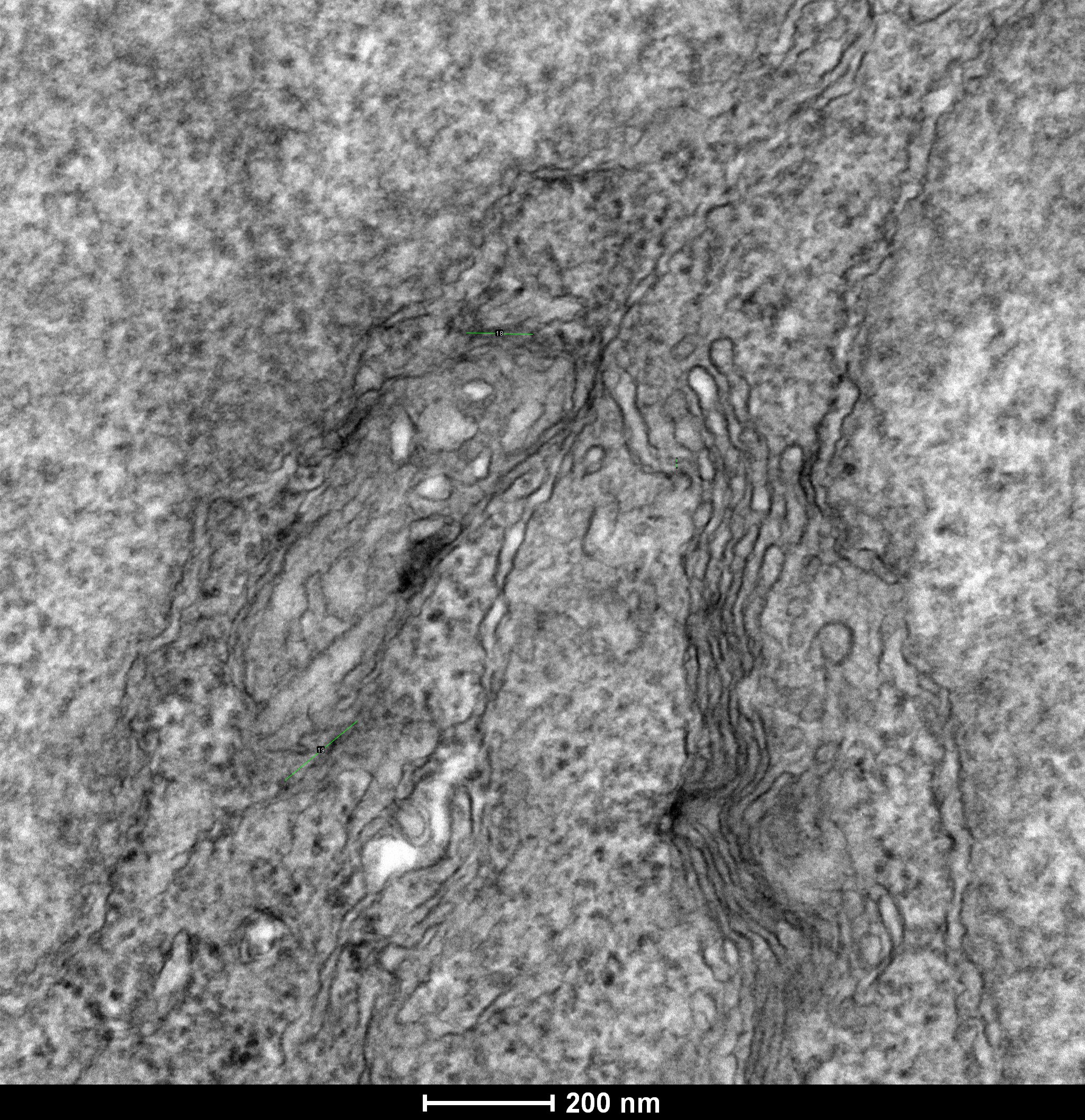

Supplement: S7 File — (ZIP) [file pone.0179859.s009.zip › Supplementary Images 4B/2a_L1_60000x_c5_m1 .jpg]

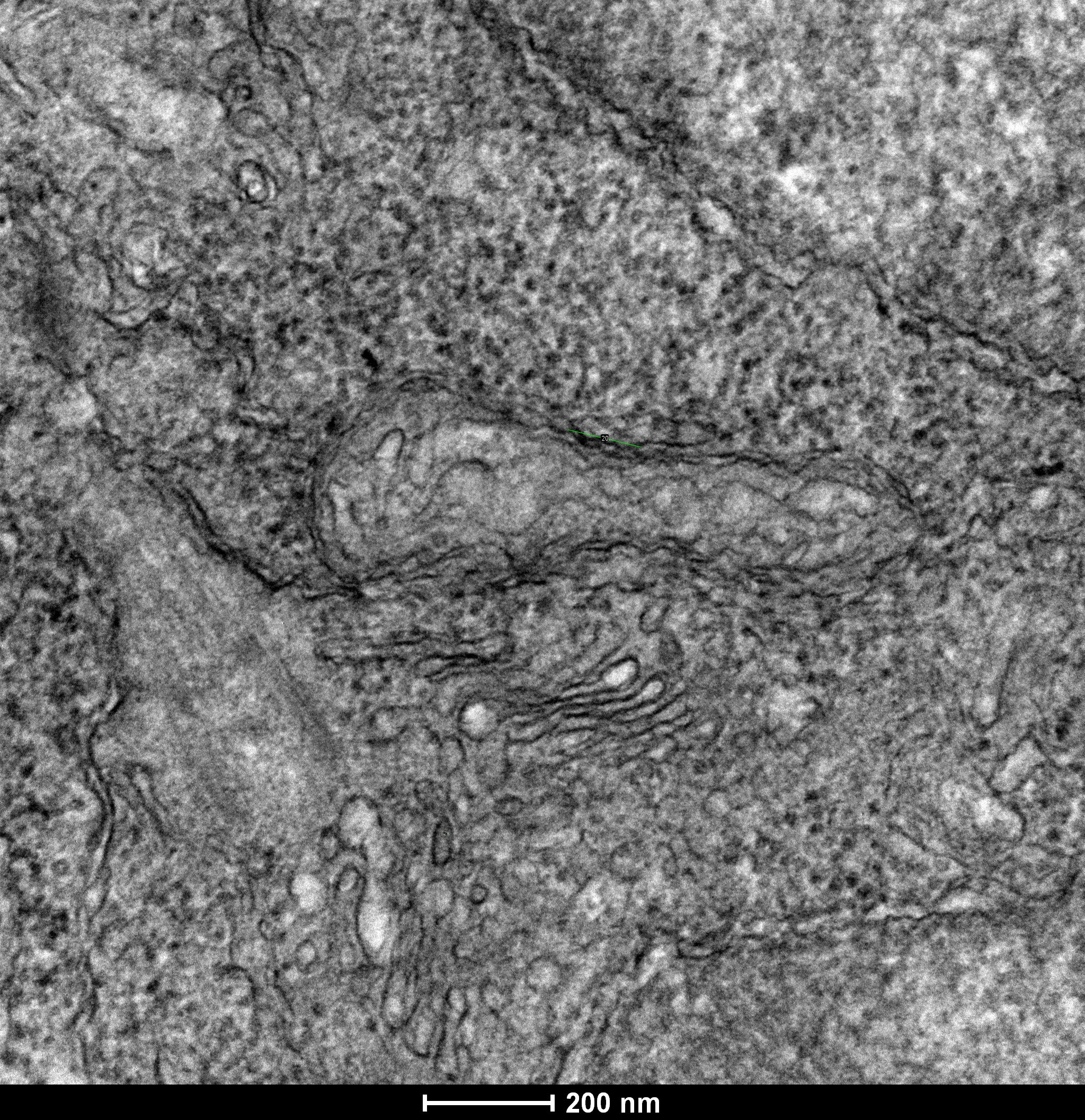

Supplement: S7 File — (ZIP) [file pone.0179859.s009.zip › Supplementary Images 4B/2a_L1_60000x_c6_m1 .jpg]

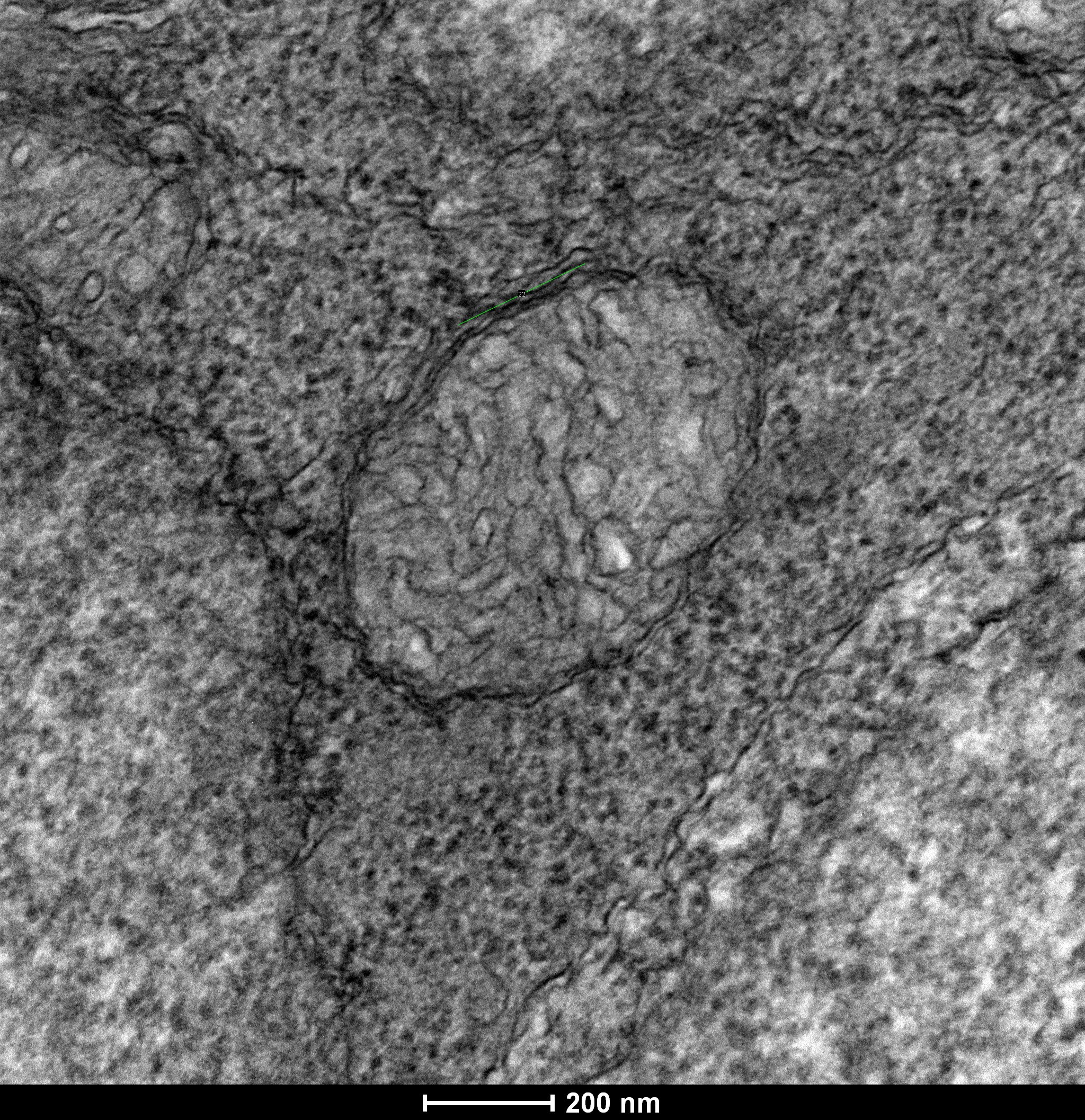

Supplement: S7 File — (ZIP) [file pone.0179859.s009.zip › Supplementary Images 4B/2a_L1_60000x_c6_m3.jpg]

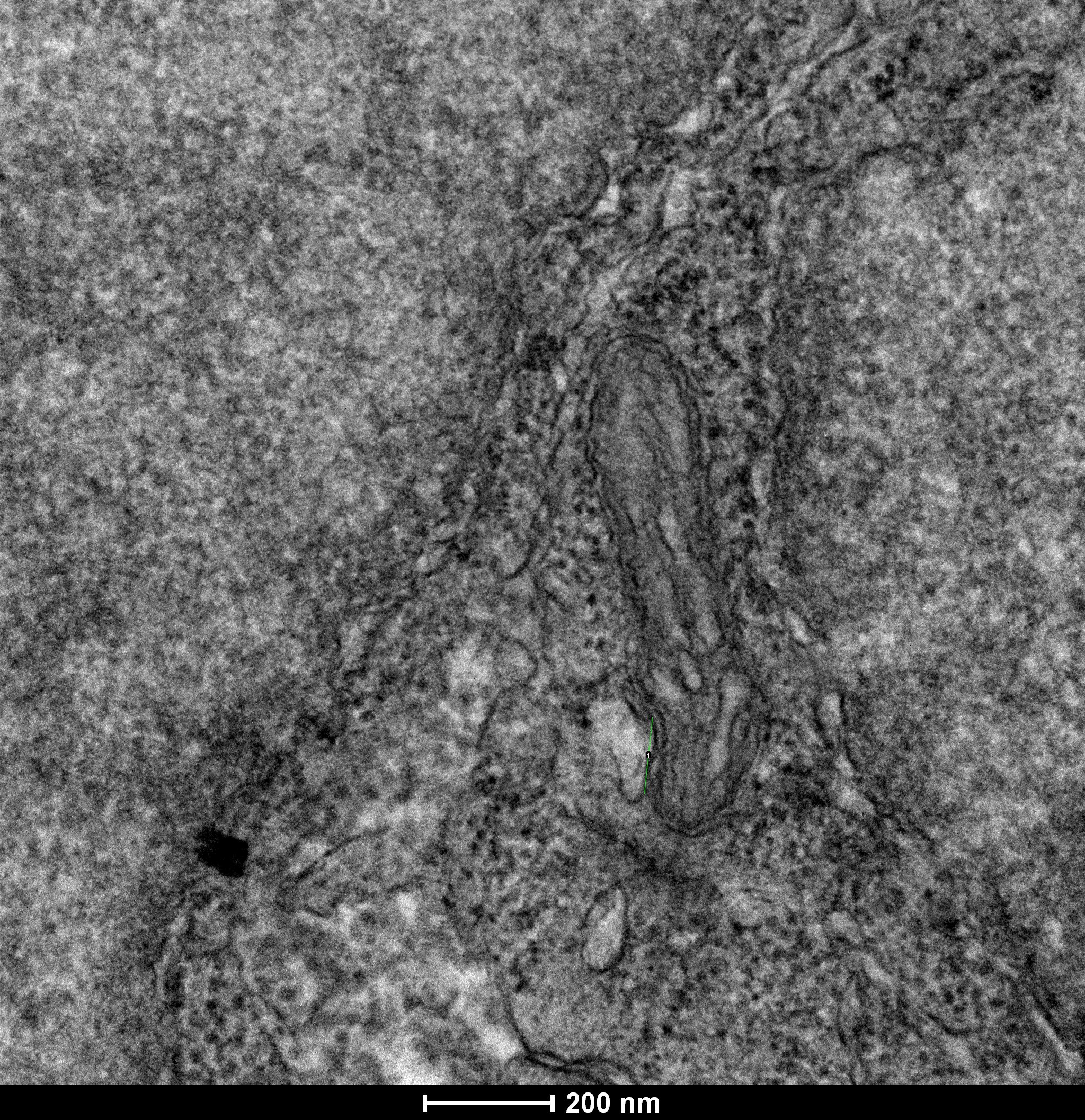

Supplement: S7 File — (ZIP) [file pone.0179859.s009.zip › Supplementary Images 4B/2c_L2_60000x_c2_m1.jpg]

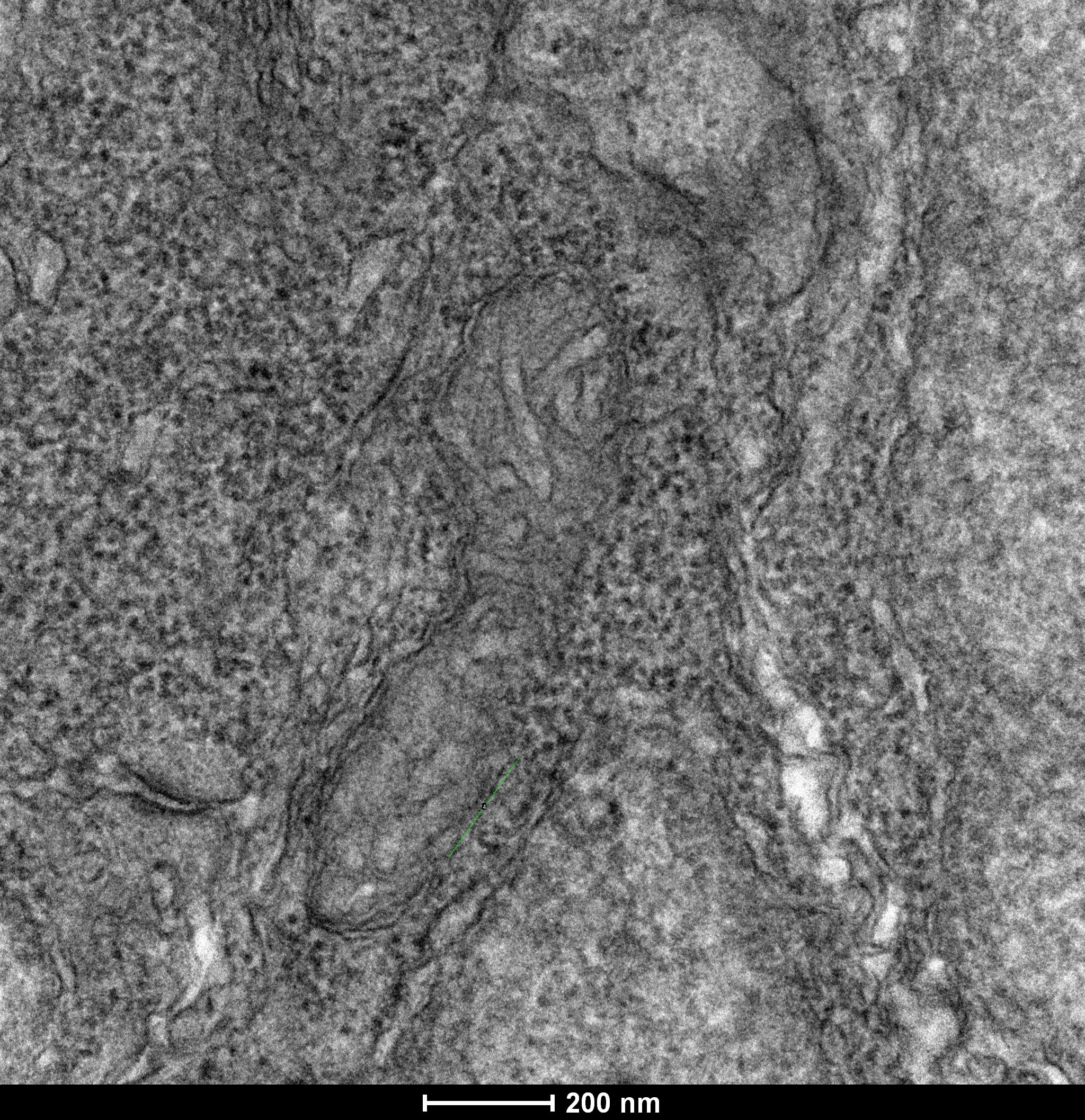

Supplement: S7 File — (ZIP) [file pone.0179859.s009.zip › Supplementary Images 4B/2c_L2_60000x_c2_m2.jpg]

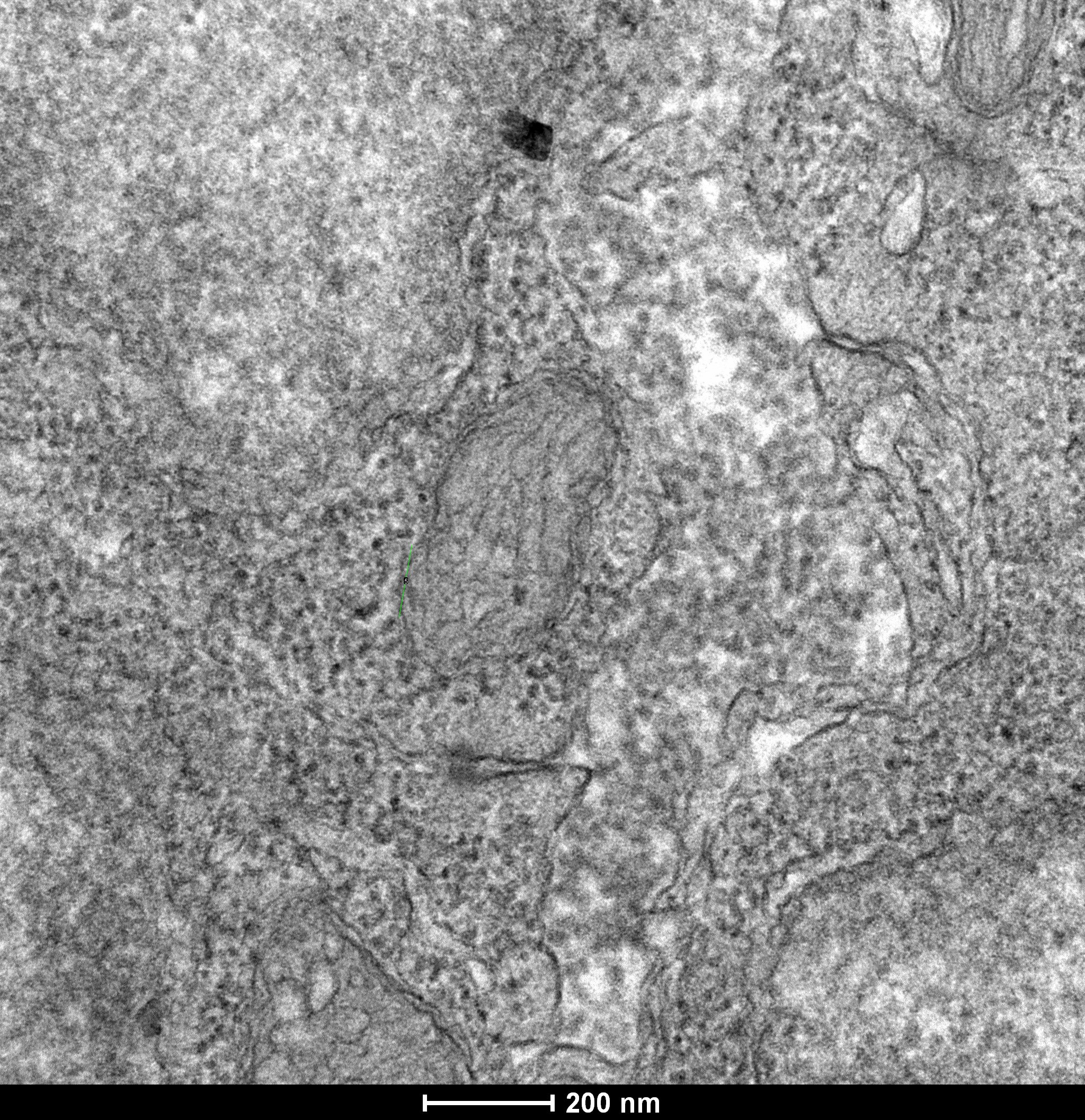

Supplement: S7 File — (ZIP) [file pone.0179859.s009.zip › Supplementary Images 4B/2c_L2_60000x_c3_m1.jpg]

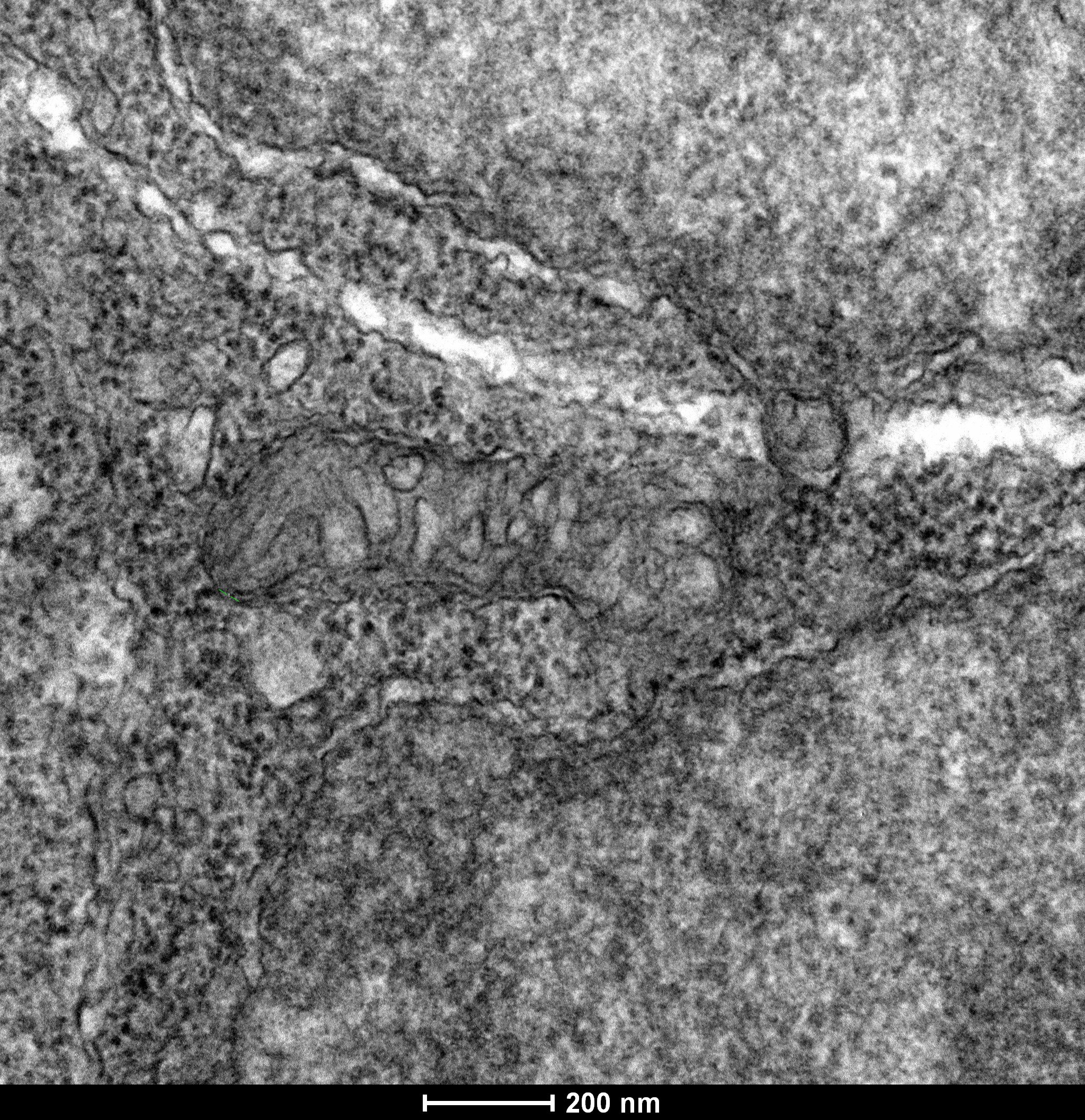

Supplement: S7 File — (ZIP) [file pone.0179859.s009.zip › Supplementary Images 4B/2c_L2_60000x_c5_m1.jpg]

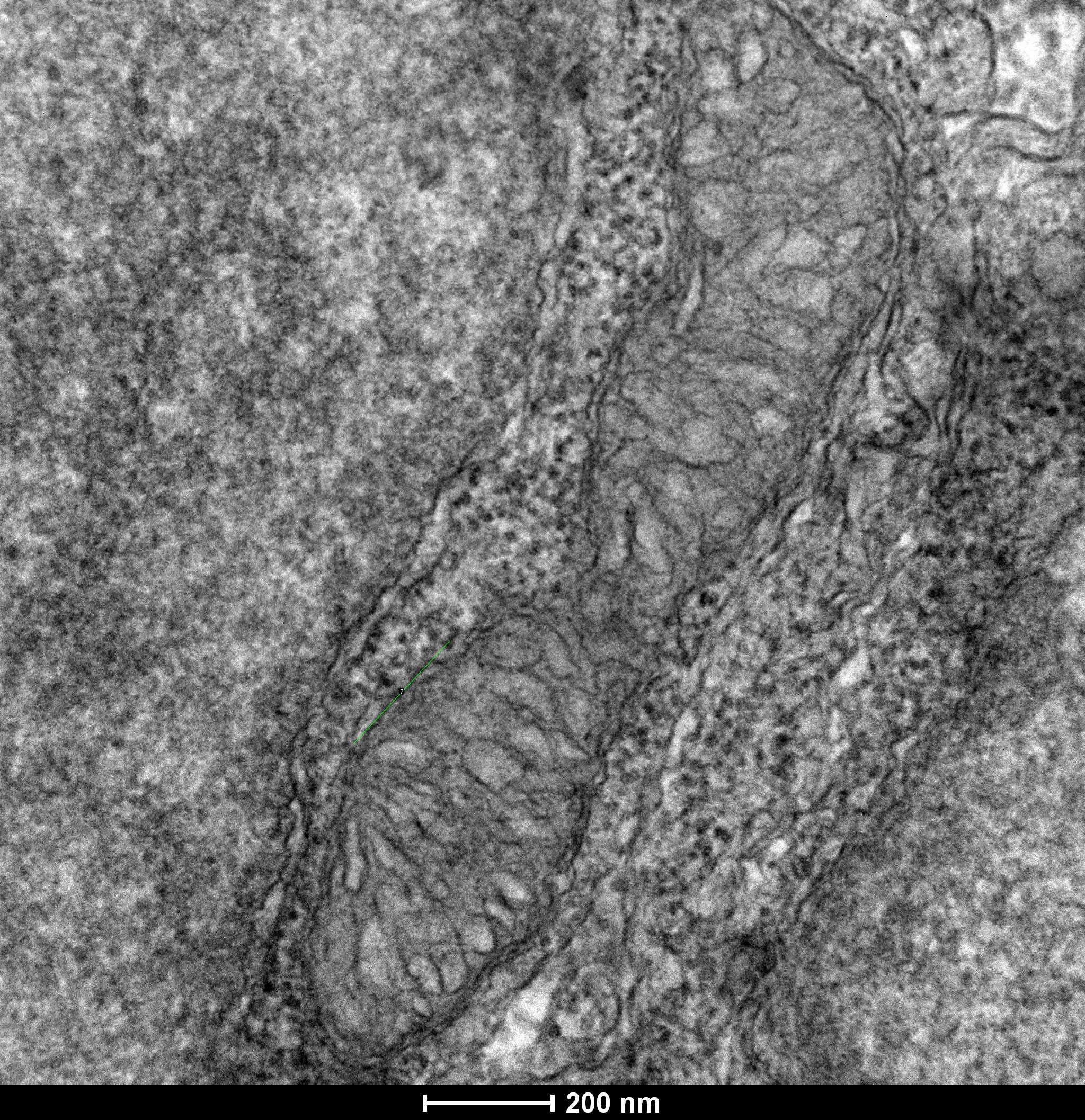

Supplement: S7 File — (ZIP) [file pone.0179859.s009.zip › Supplementary Images 4B/2c_L2_60000x_c5_m2.jpg]

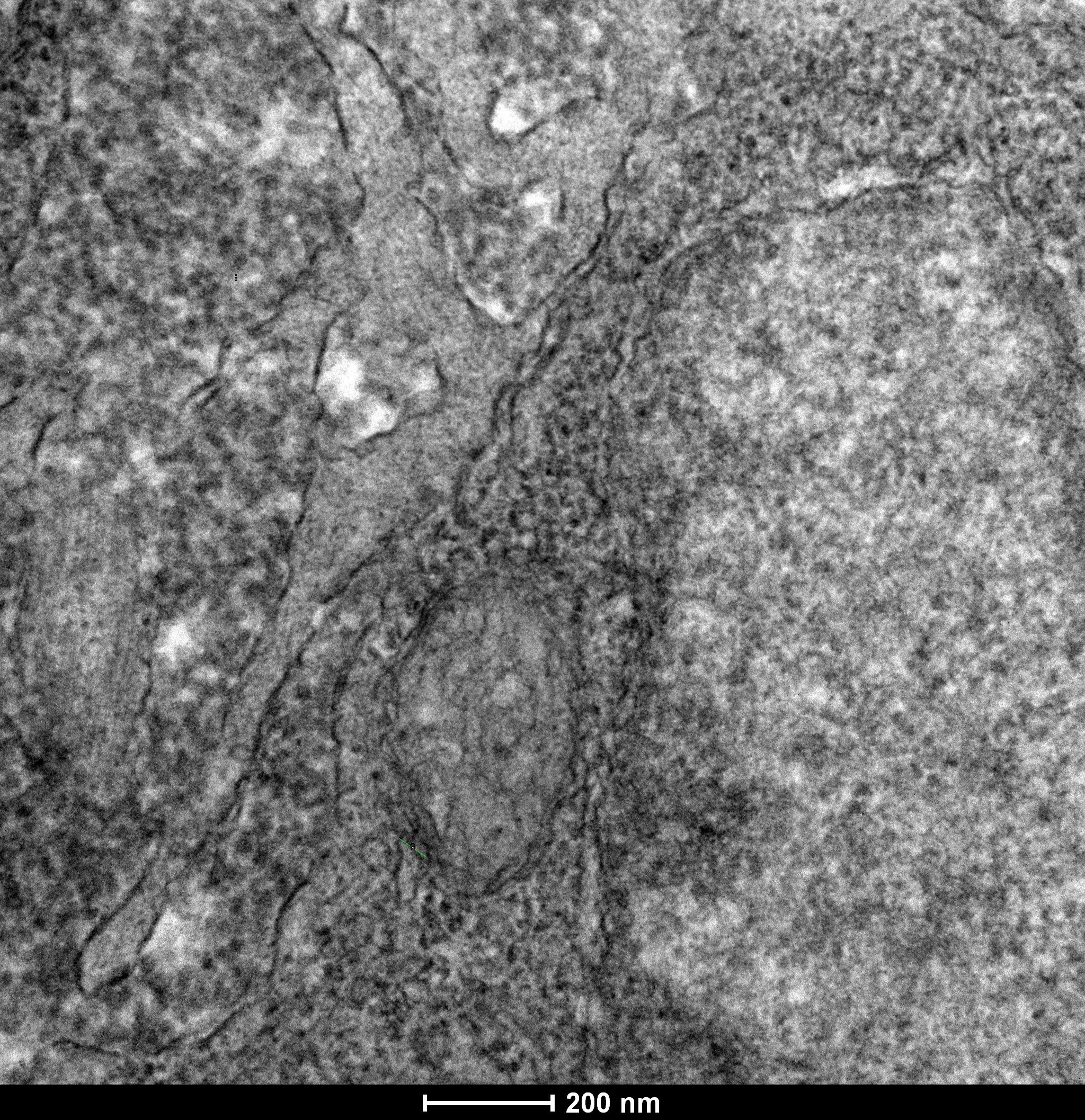

Supplement: S7 File — (ZIP) [file pone.0179859.s009.zip › Supplementary Images 4B/2c_L2_60000x_c9_m1.1.jpg]

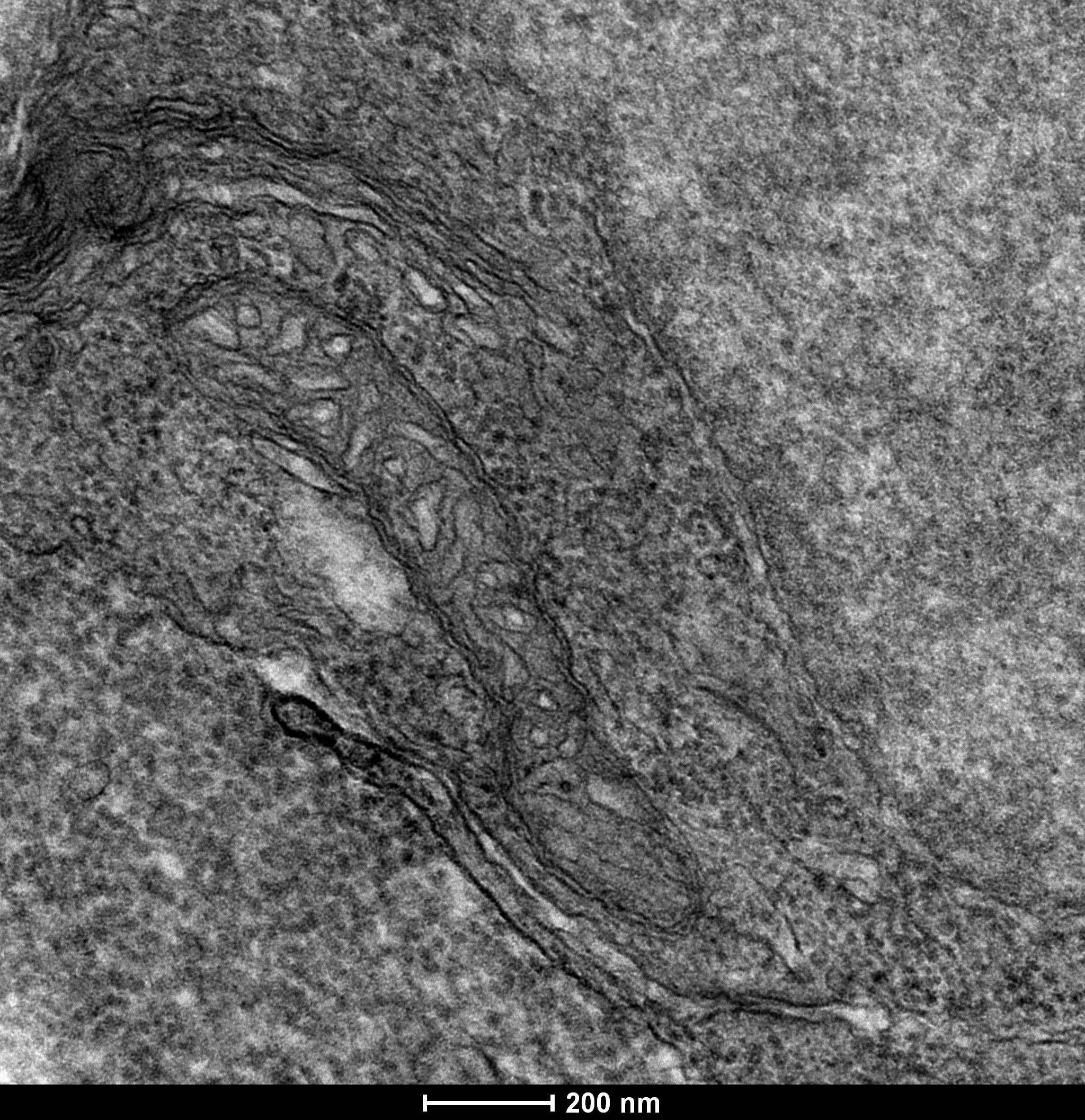

Supplement: S7 File — (ZIP) [file pone.0179859.s009.zip › Supplementary Images 4B/2b_L2_60000x_c1_M1.jpg]

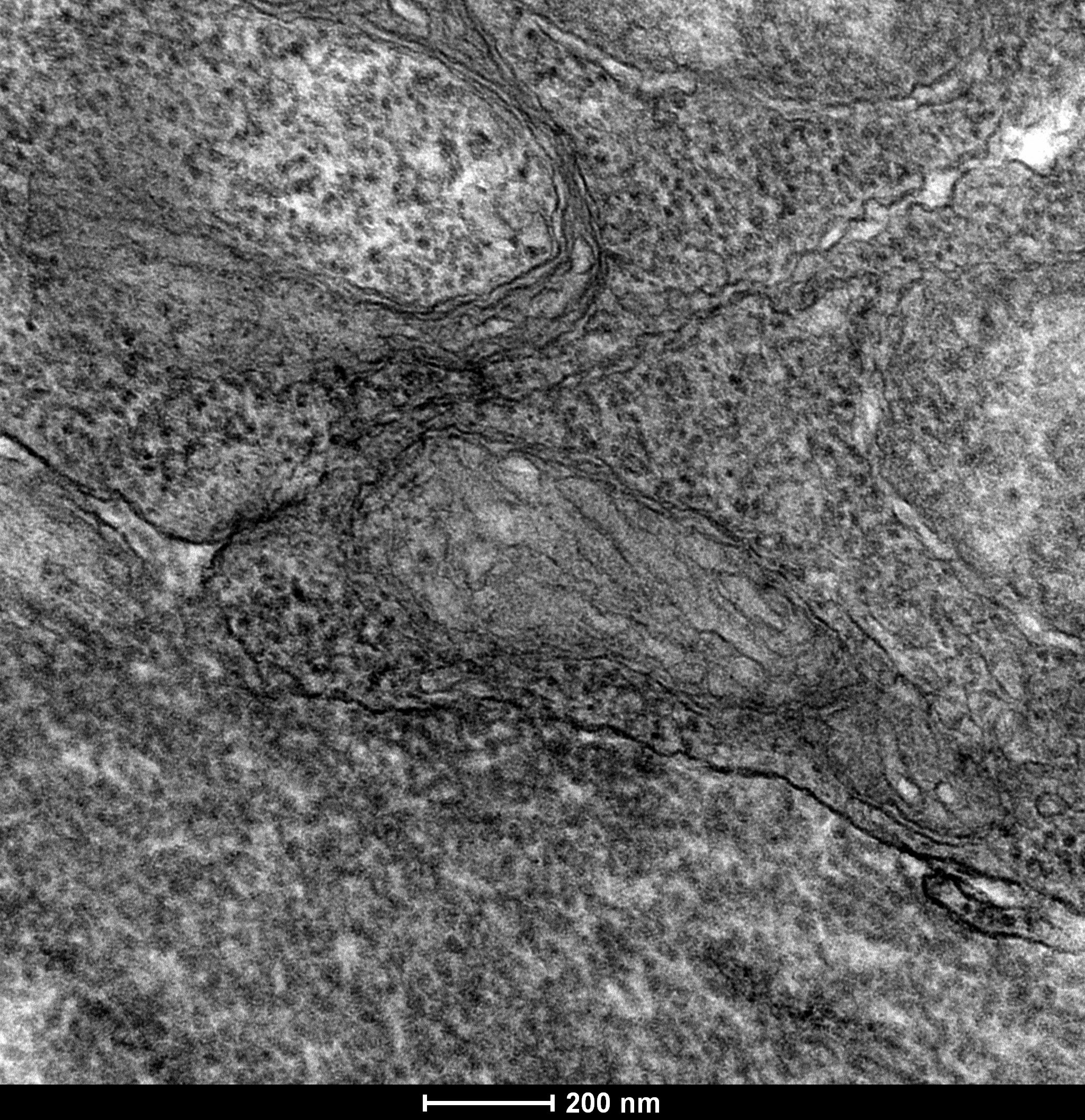

Supplement: S7 File — (ZIP) [file pone.0179859.s009.zip › Supplementary Images 4B/2b_L2_60000x_c1_M2.jpg]

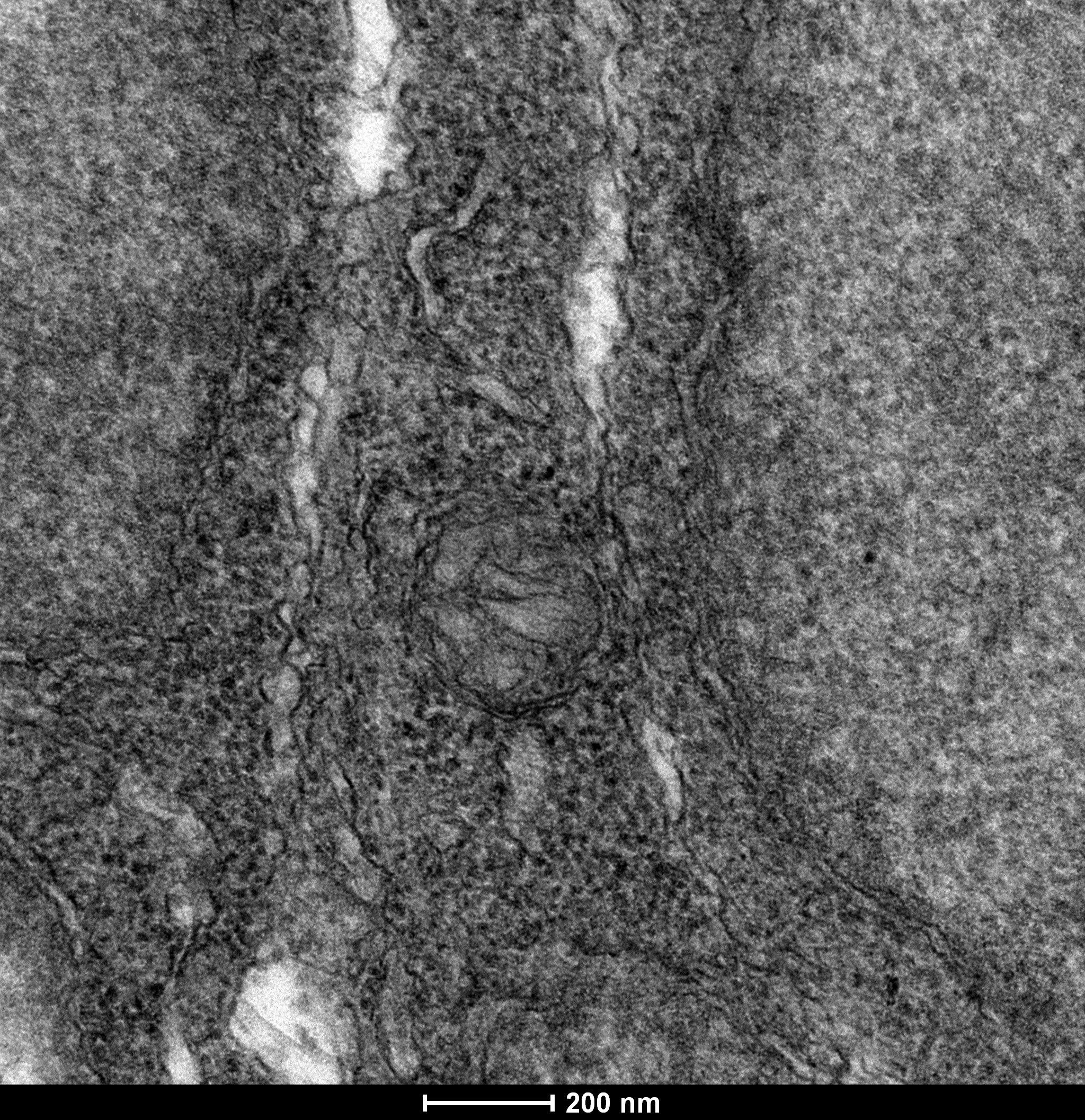

Supplement: S7 File — (ZIP) [file pone.0179859.s009.zip › Supplementary Images 4B/2b_L2_60000x_c3_M2.jpg]

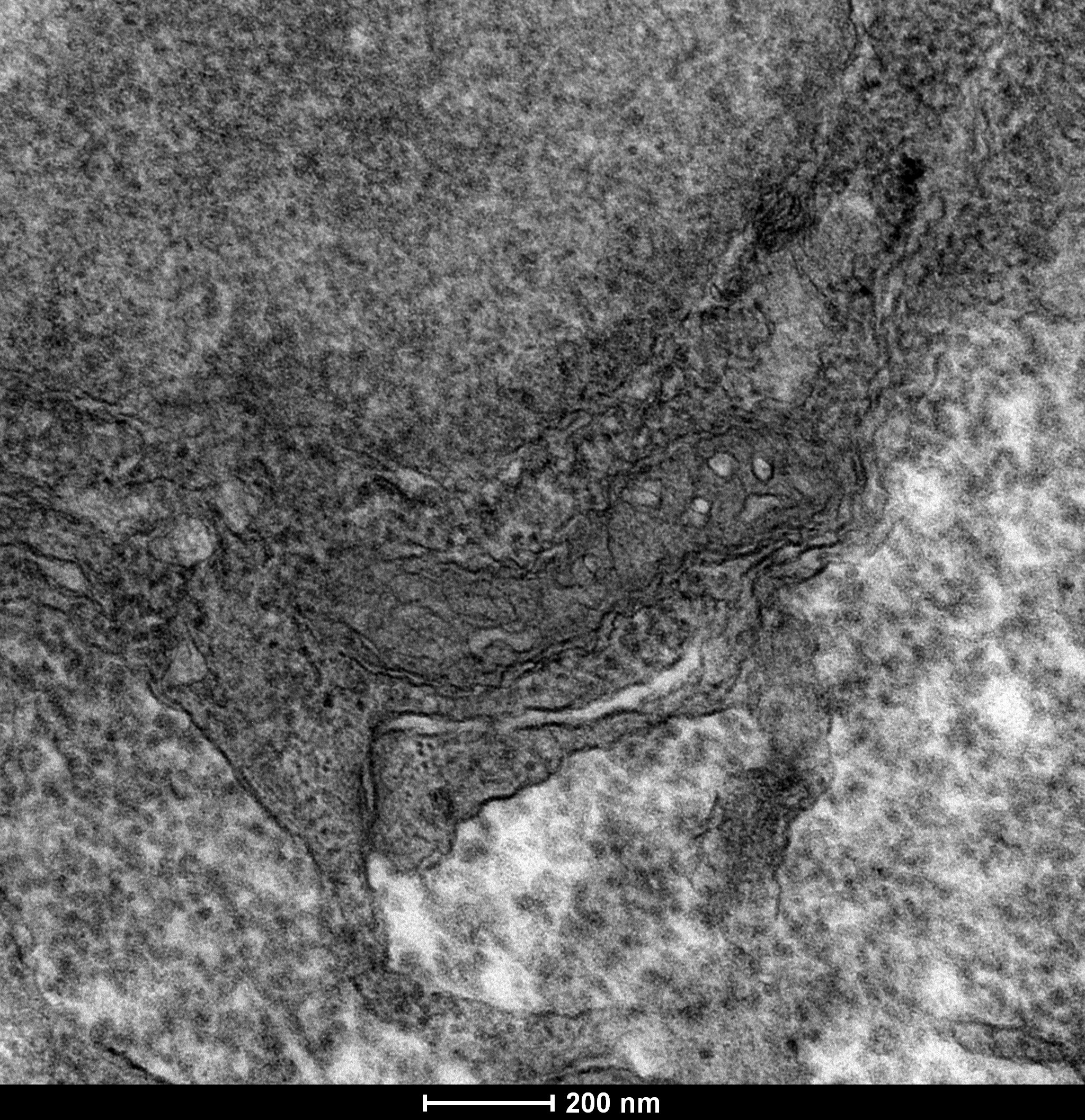

Supplement: S7 File — (ZIP) [file pone.0179859.s009.zip › Supplementary Images 4B/2b_L2_60000x_c4_M2.jpg]

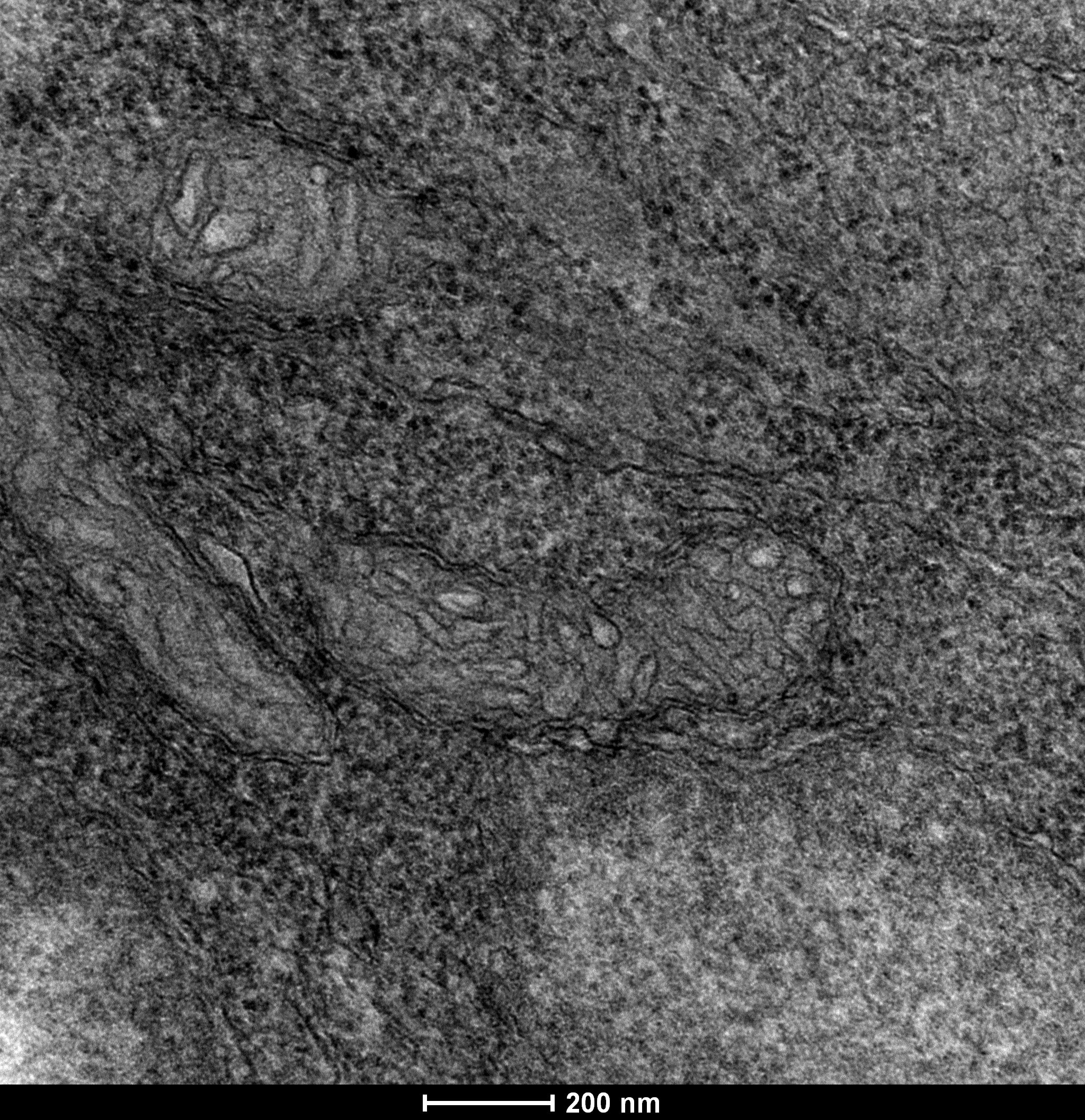

Supplement: S7 File — (ZIP) [file pone.0179859.s009.zip › Supplementary Images 4B/2b_L2_60000x_c6_M1 .jpg]

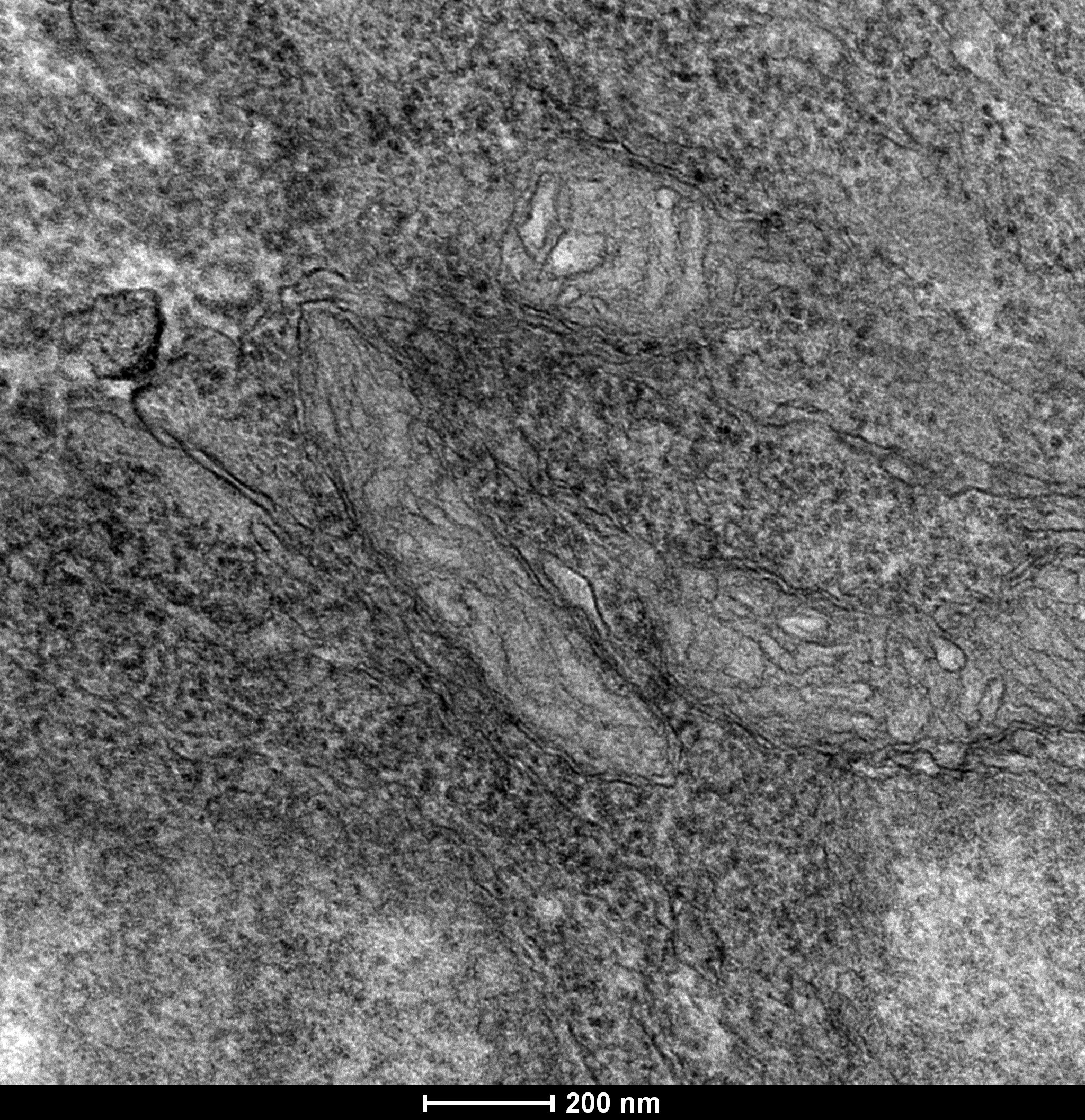

Supplement: S7 File — (ZIP) [file pone.0179859.s009.zip › Supplementary Images 4B/2b_L2_60000x_c6_M2 .jpg]

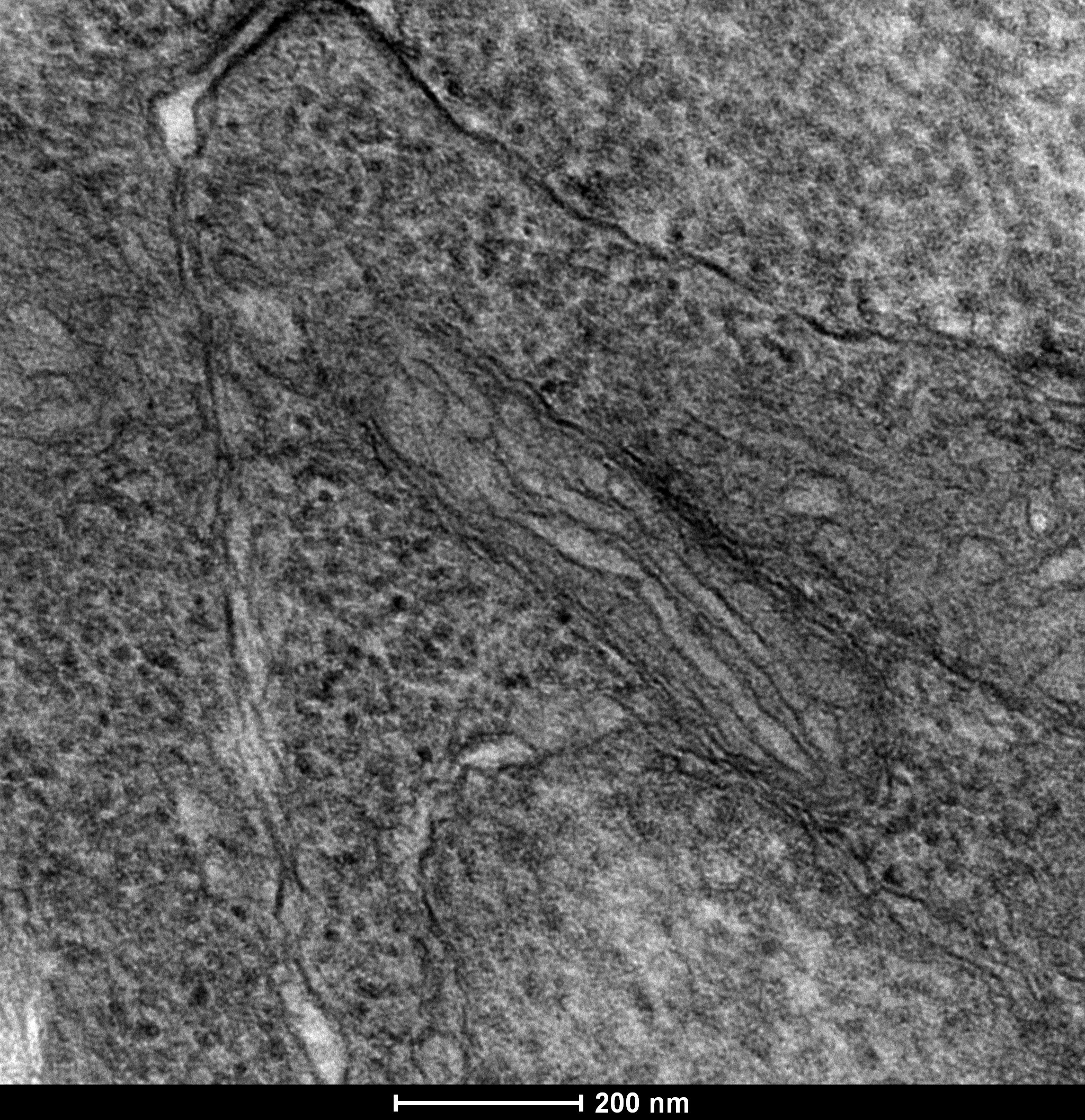

Supplement: S7 File — (ZIP) [file pone.0179859.s009.zip › Supplementary Images 4B/2b_L2_87000x_c5_M2 .jpg]

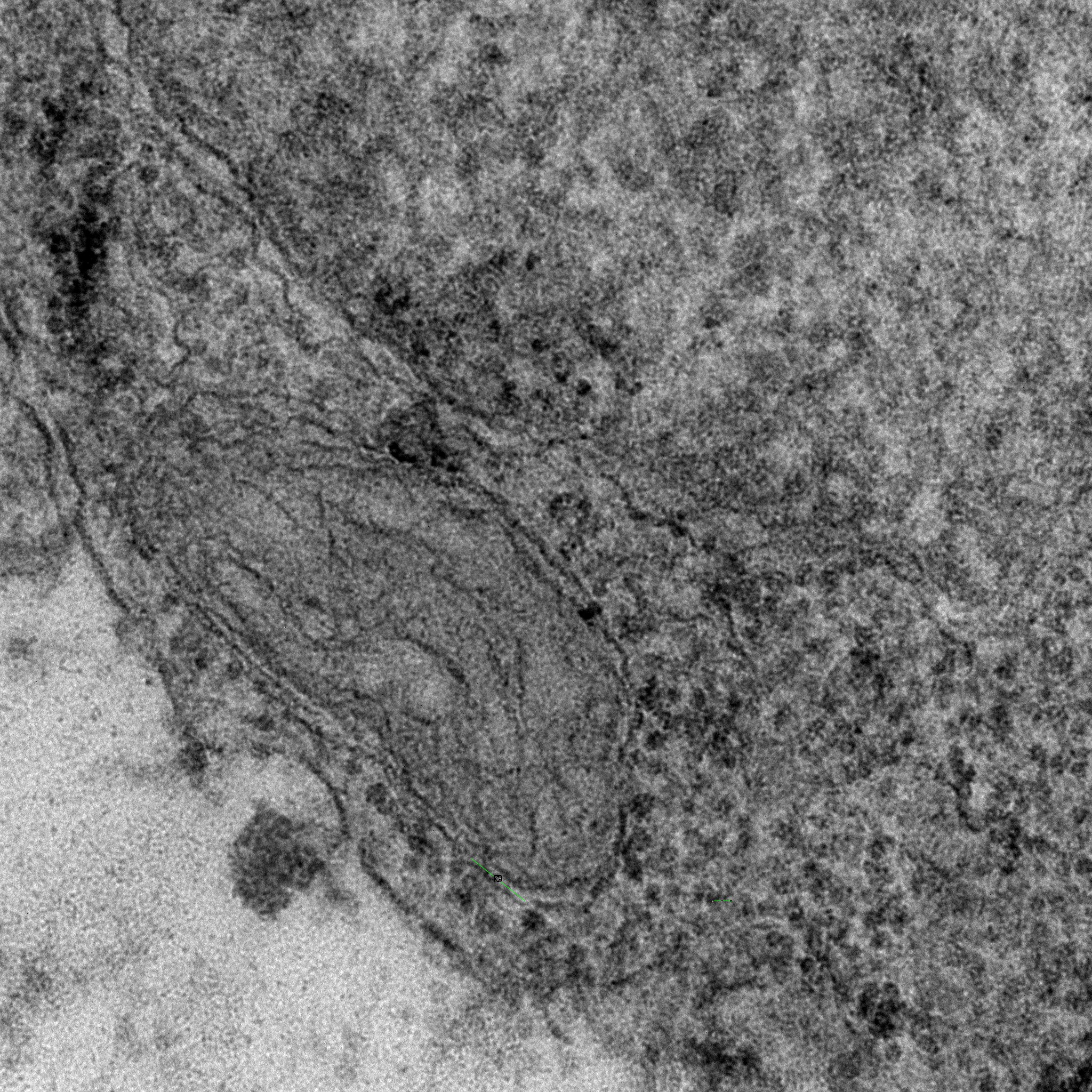

Supplement: S8 File — (ZIP) [file pone.0179859.s010.zip › Supplementary Images 4C/3a_L1_87000x_c4_m1.jpg]

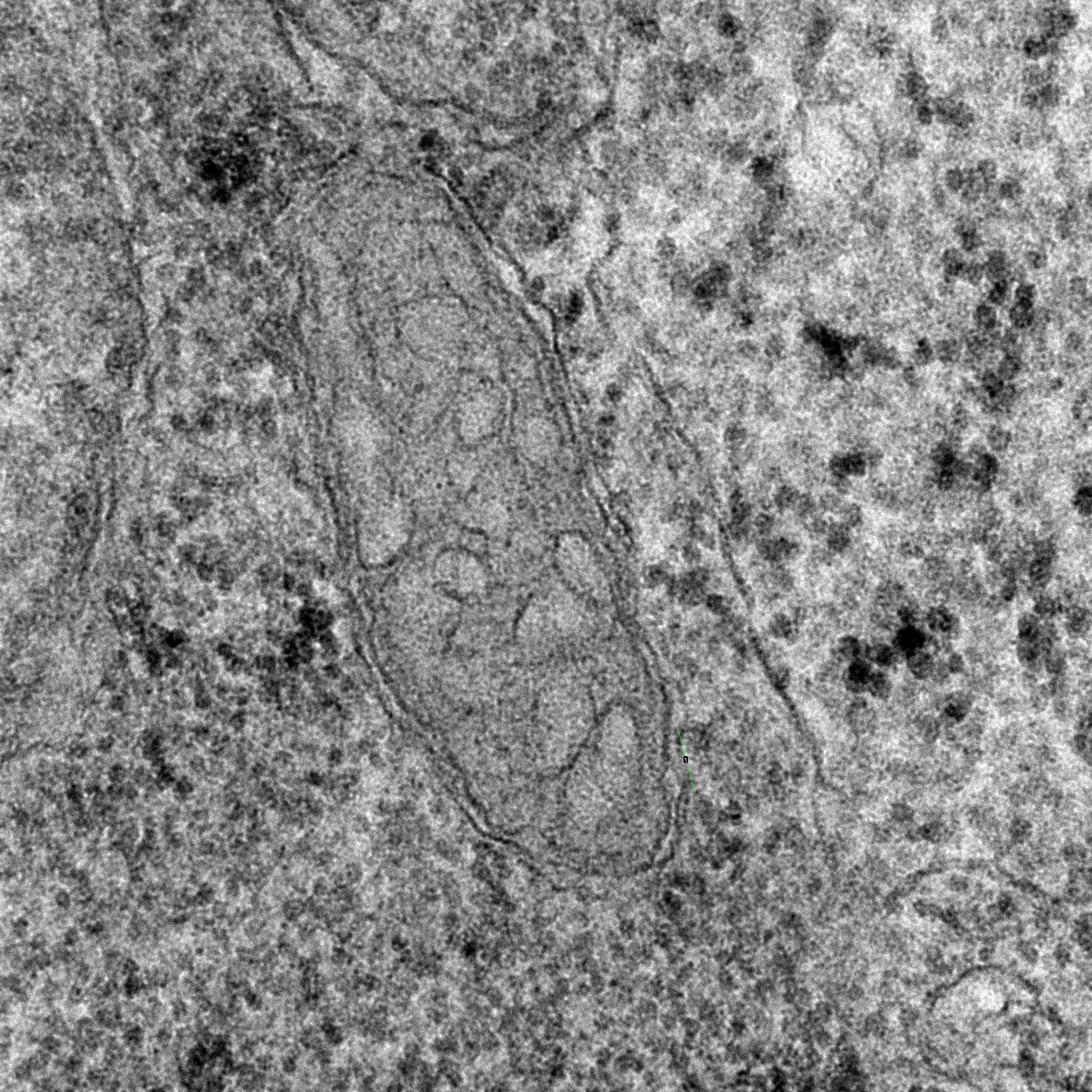

Supplement: S8 File — (ZIP) [file pone.0179859.s010.zip › Supplementary Images 4C/3a_L1_87000x_c4_m2.jpg]

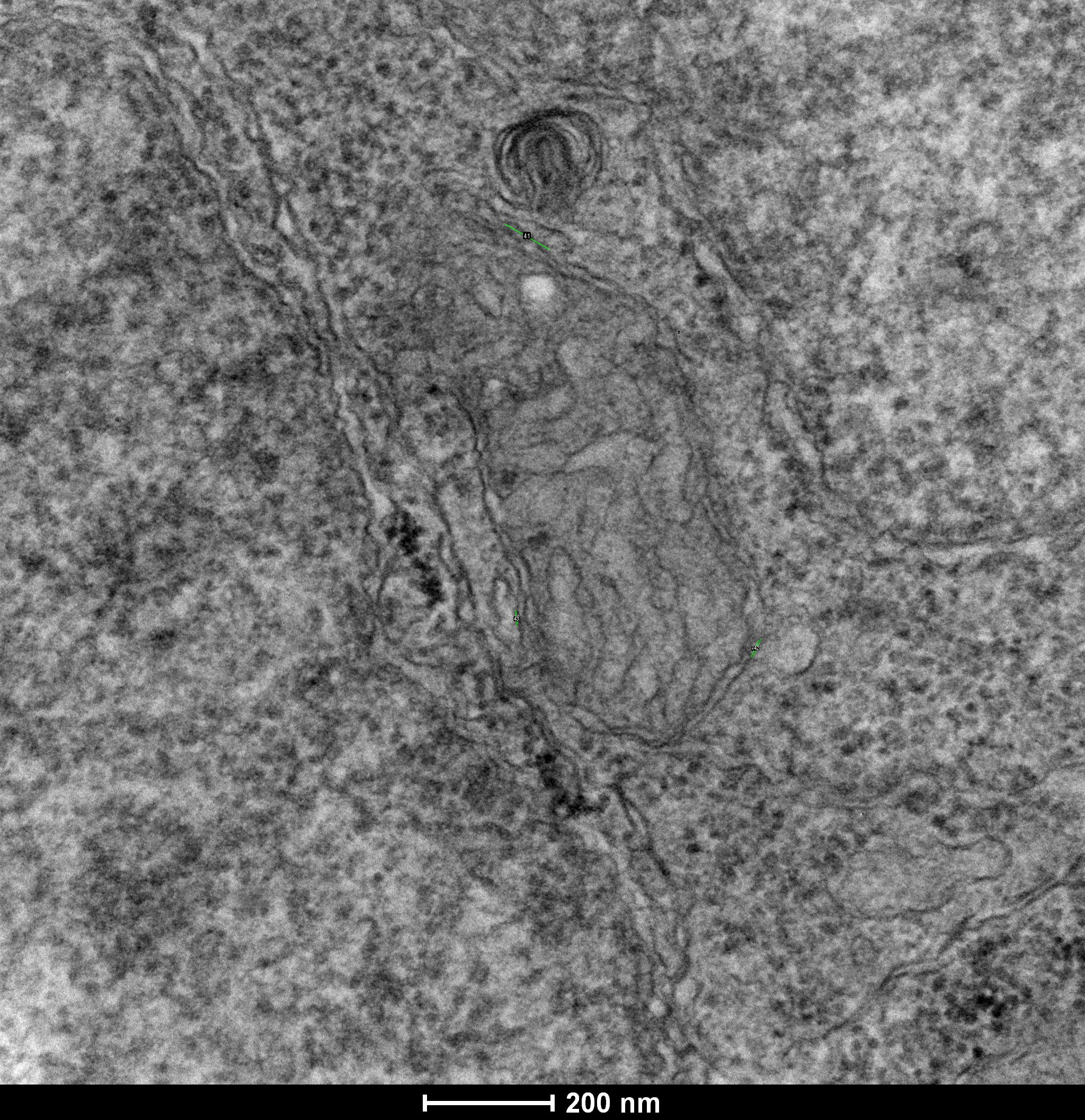

Supplement: S8 File — (ZIP) [file pone.0179859.s010.zip › Supplementary Images 4C/3a_L2_60000x_c1_m1.jpg]

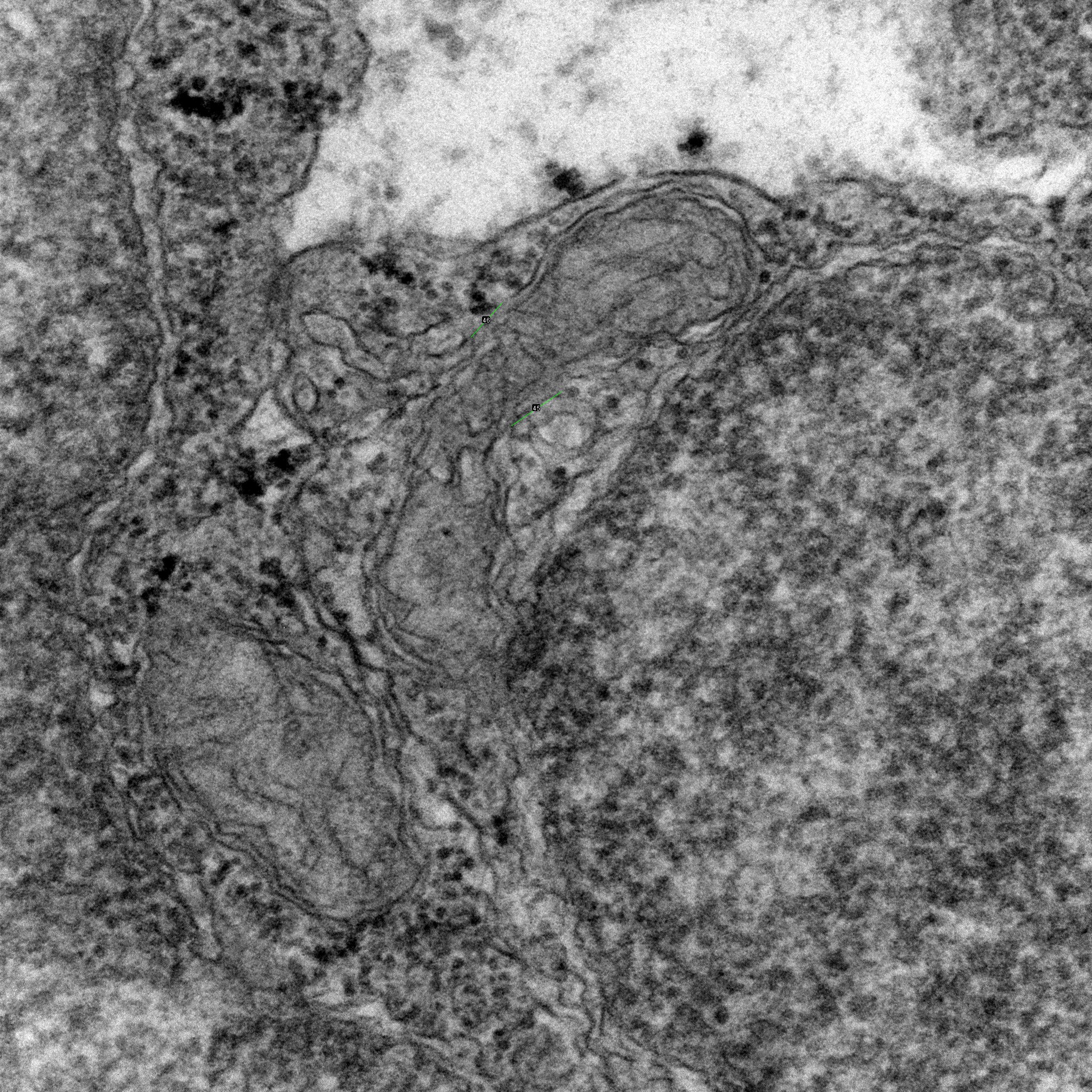

Supplement: S8 File — (ZIP) [file pone.0179859.s010.zip › Supplementary Images 4C/3a_L2_60000x_c5_m1.jpg]

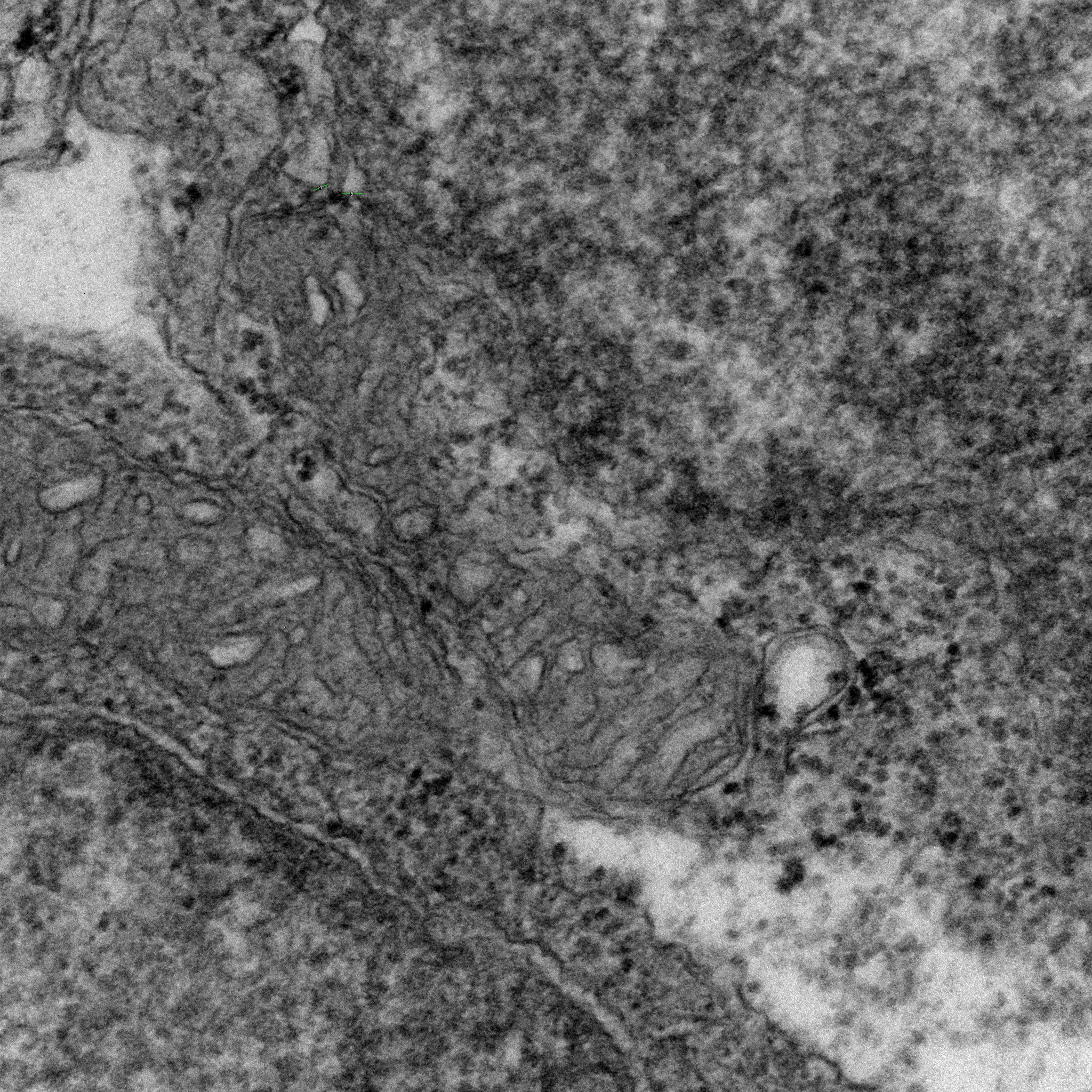

Supplement: S8 File — (ZIP) [file pone.0179859.s010.zip › Supplementary Images 4C/3a_L2_60000x_c5_m2.jpg]

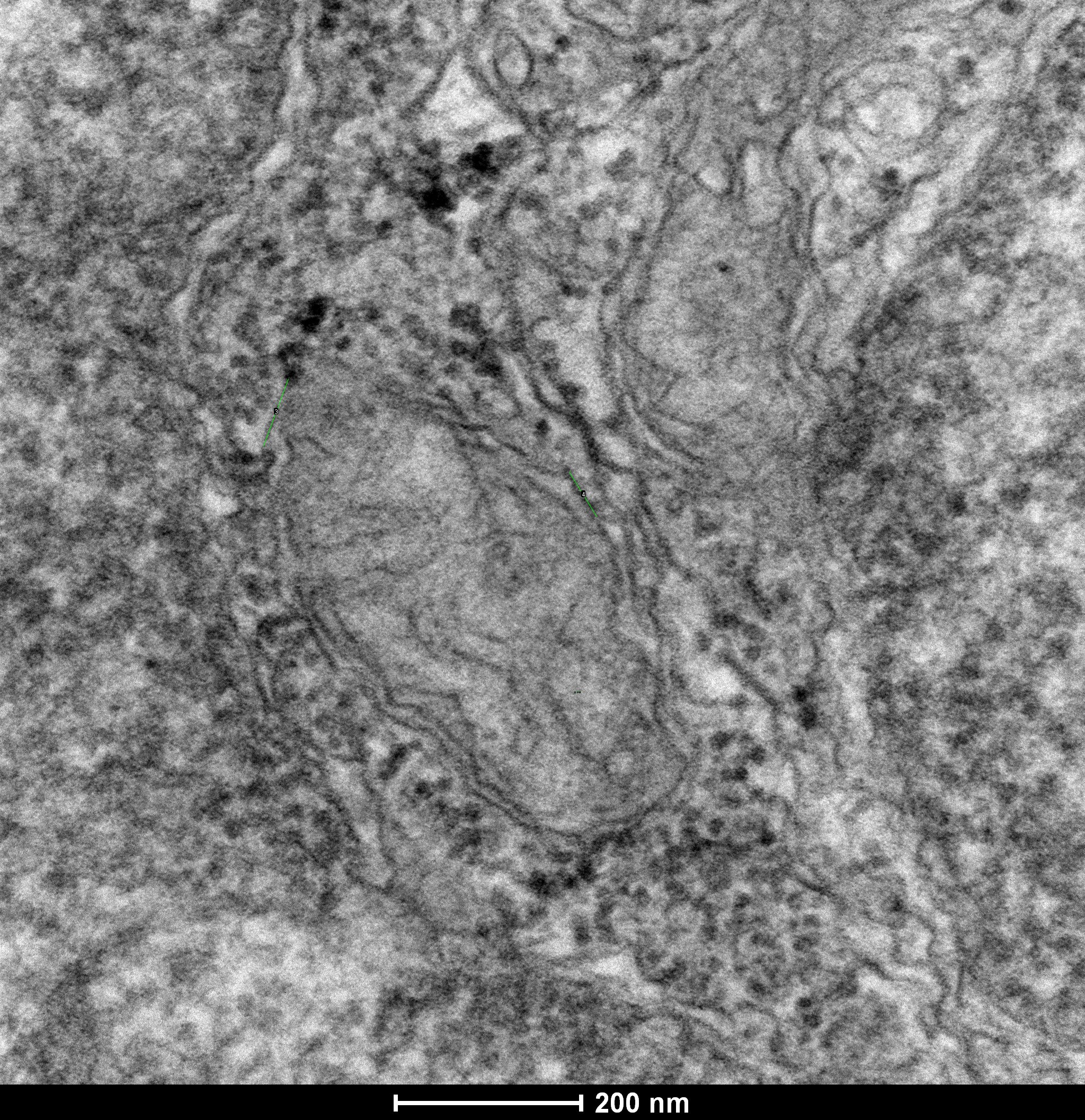

Supplement: S8 File — (ZIP) [file pone.0179859.s010.zip › Supplementary Images 4C/3a_L2_87000x_c3_m1.jpg]

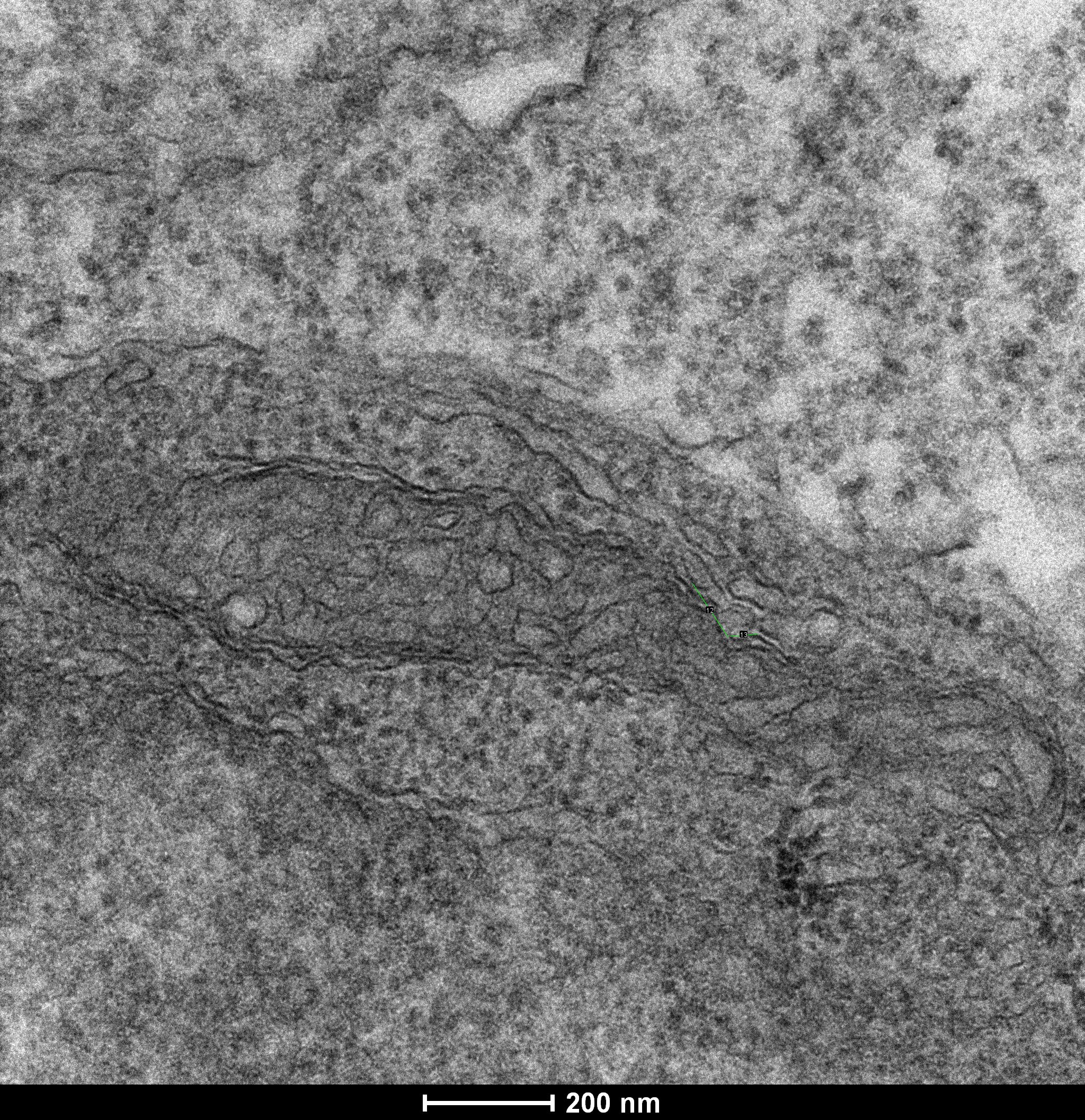

Supplement: S8 File — (ZIP) [file pone.0179859.s010.zip › Supplementary Images 4C/3b_L1_60000x_c1_m1.jpg]

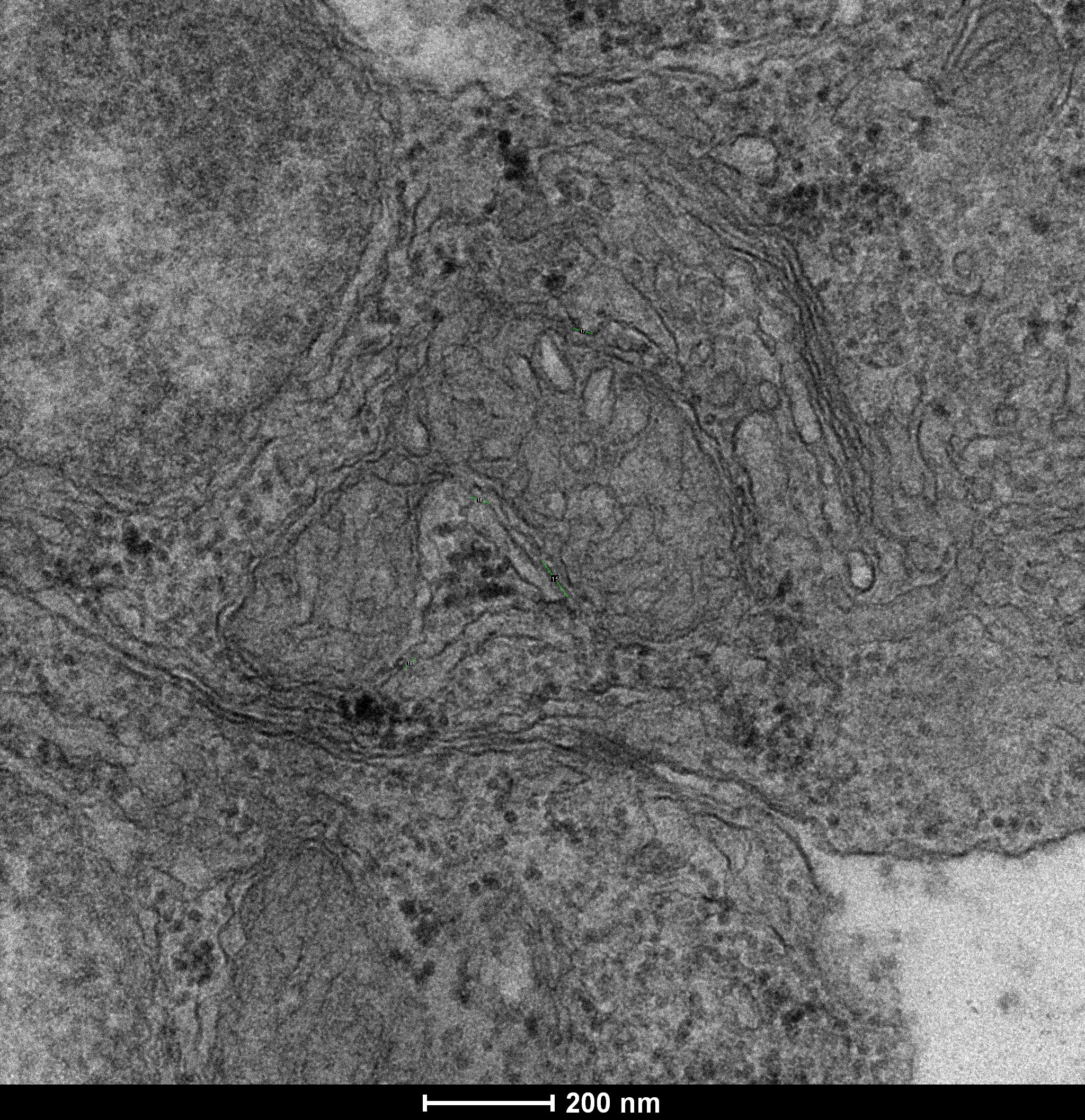

Supplement: S8 File — (ZIP) [file pone.0179859.s010.zip › Supplementary Images 4C/3b_L1_60000x_c3_m1_m2.jpg]

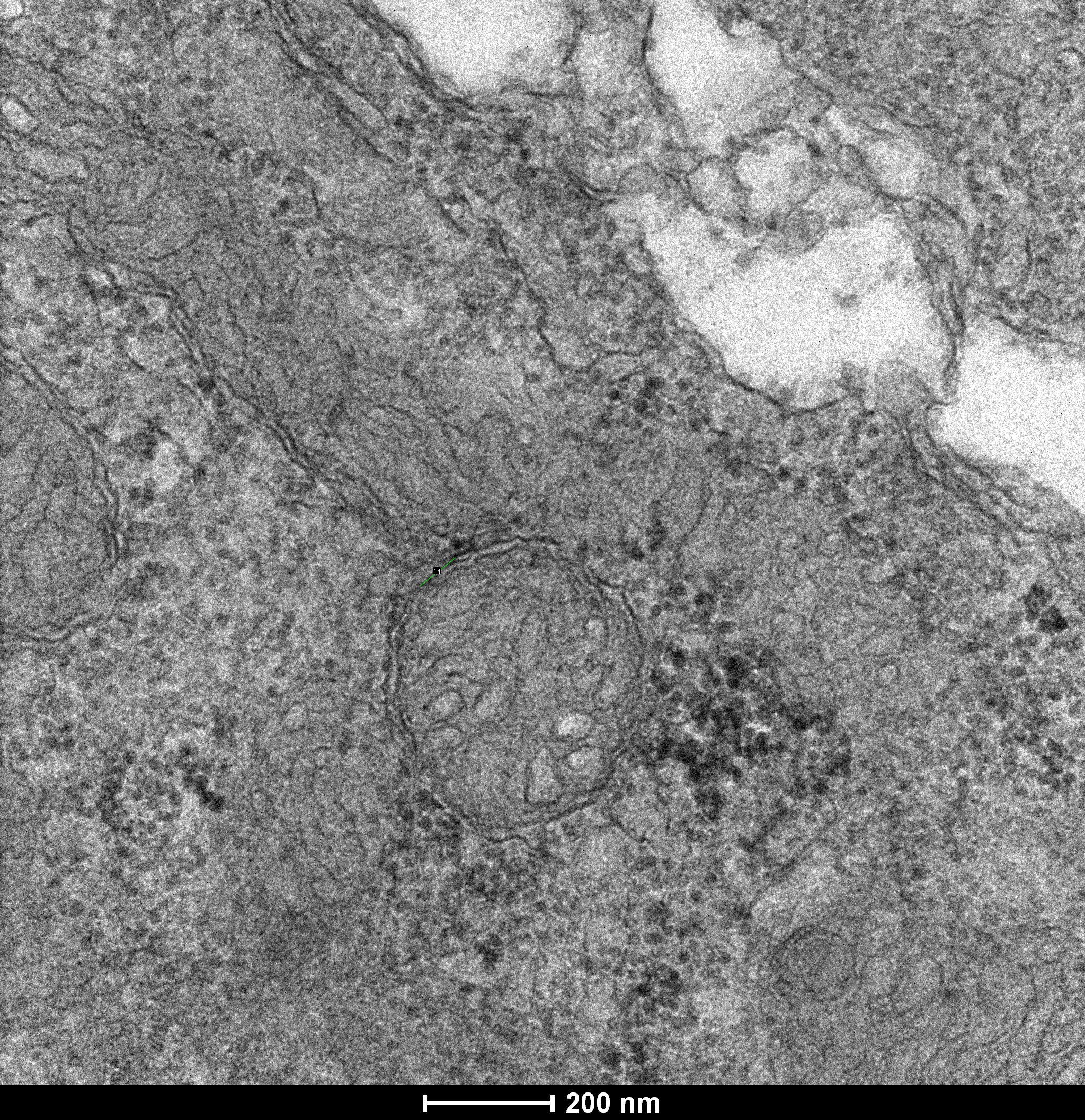

Supplement: S8 File — (ZIP) [file pone.0179859.s010.zip › Supplementary Images 4C/3b_L1_60000x_c3_m3.jpg]

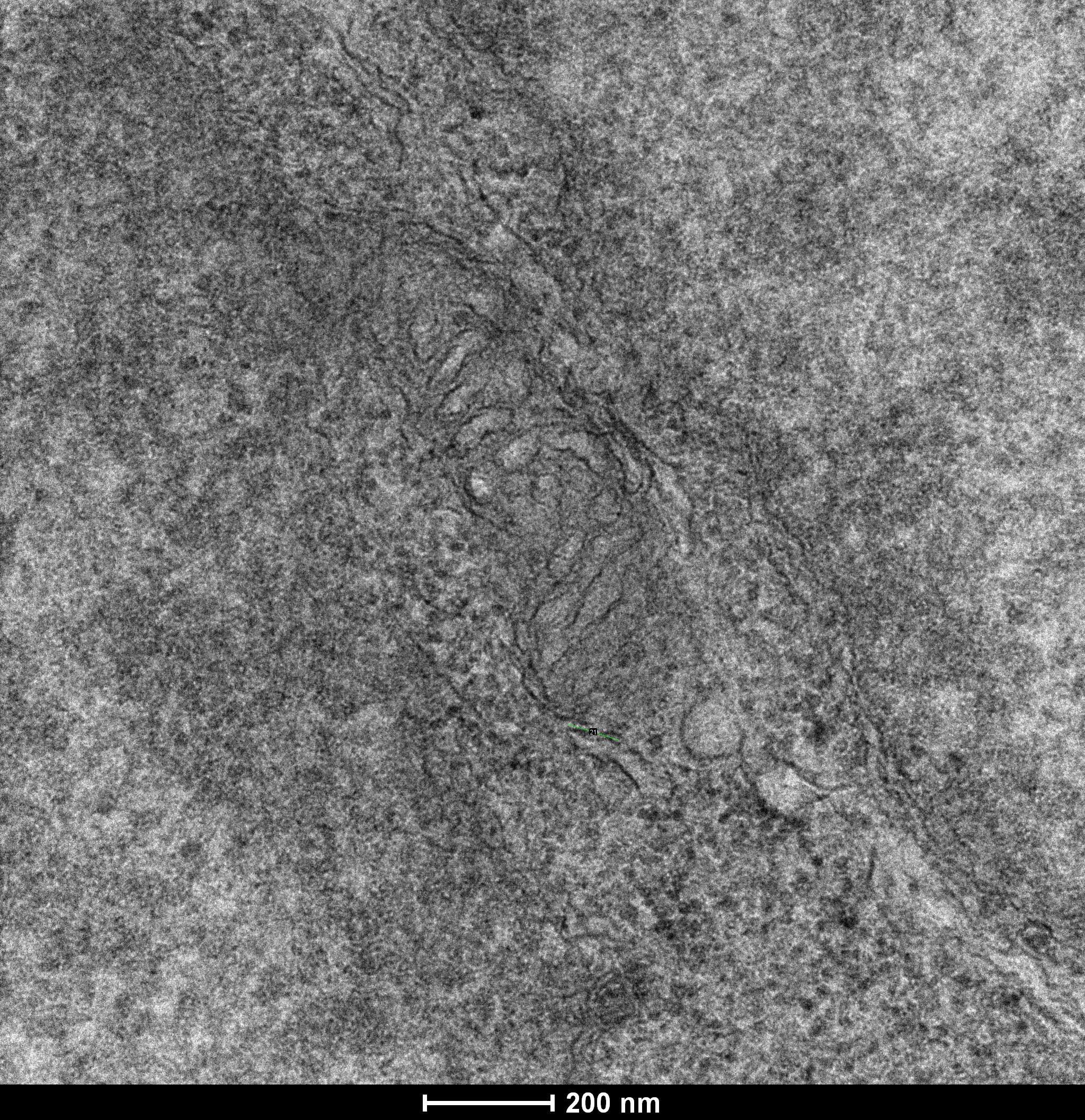

Supplement: S8 File — (ZIP) [file pone.0179859.s010.zip › Supplementary Images 4C/3b_L1_60000x_c4_m1.jpg]

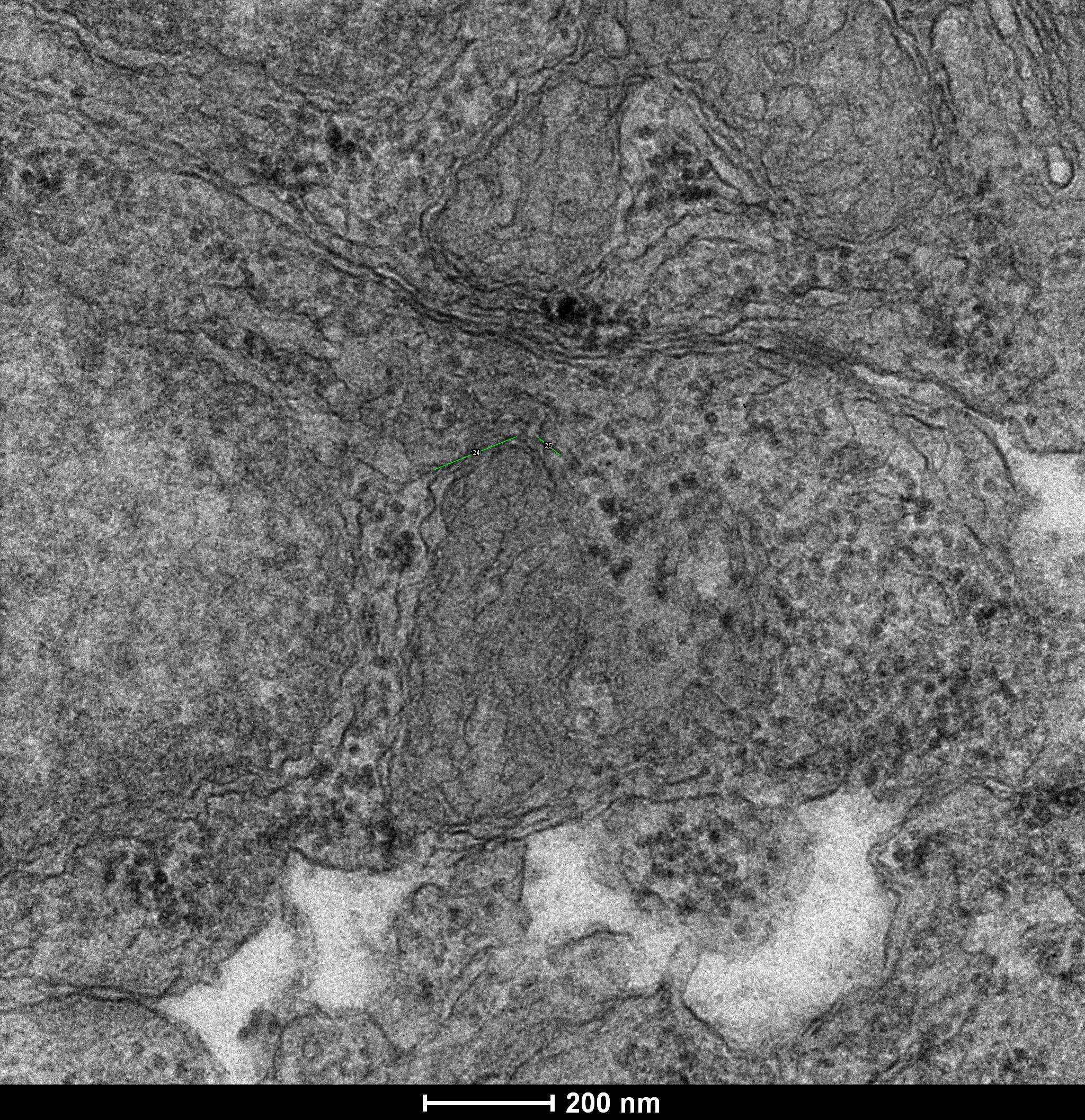

Supplement: S8 File — (ZIP) [file pone.0179859.s010.zip › Supplementary Images 4C/3b_L1_60000x_c4_m2.jpg]

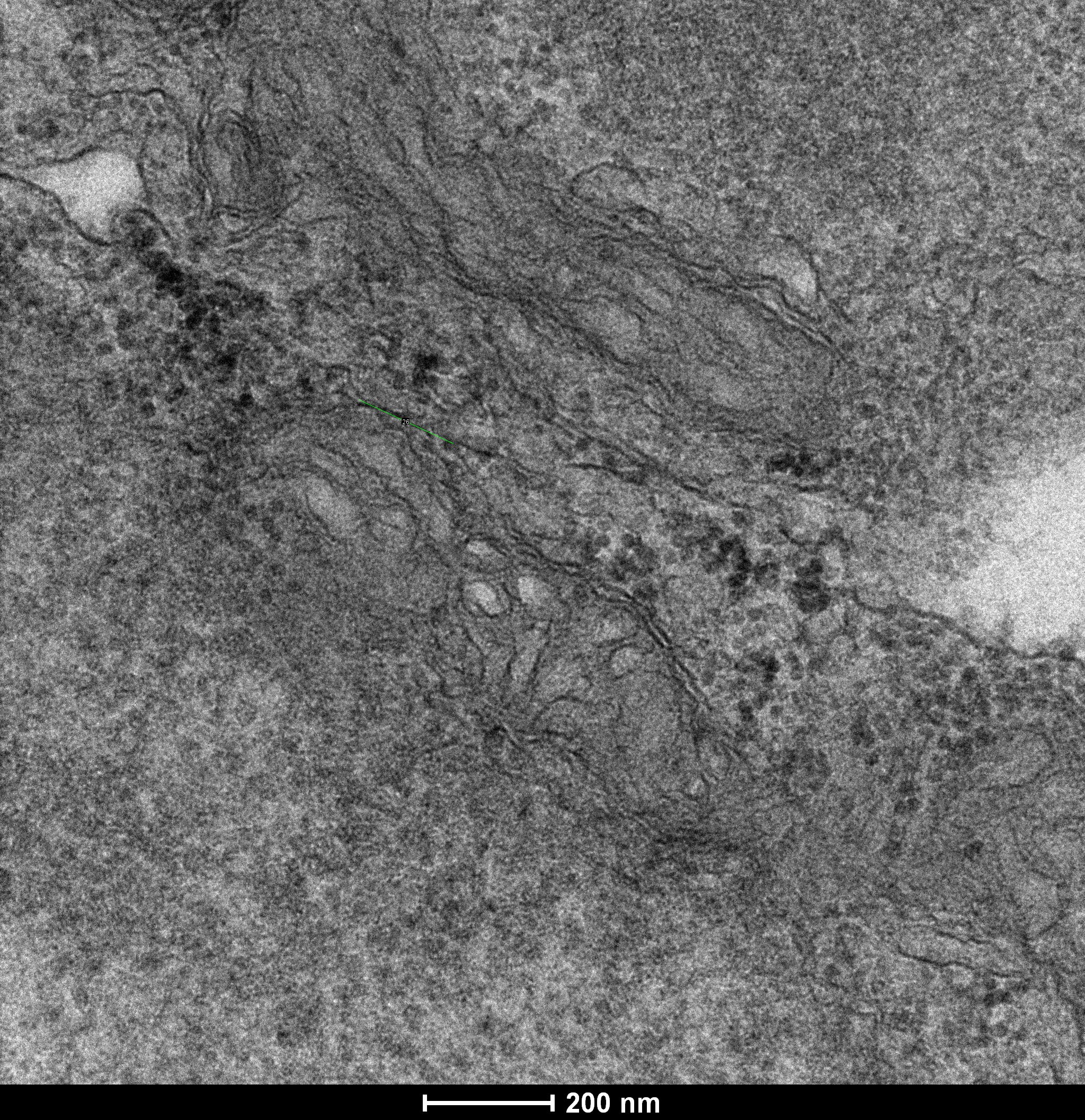

Supplement: S8 File — (ZIP) [file pone.0179859.s010.zip › Supplementary Images 4C/3b_L1_60000x_c5_m1.jpg]

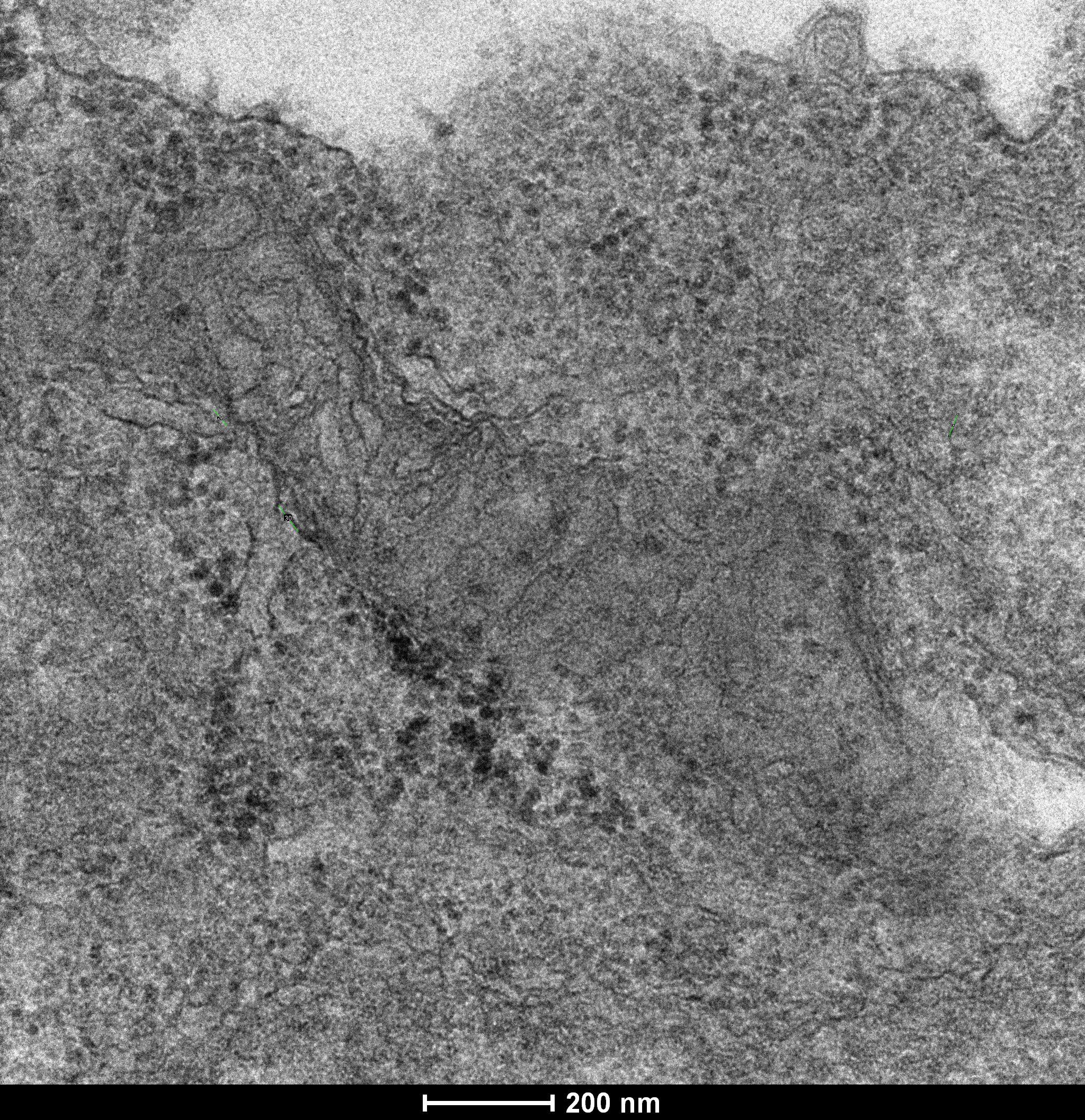

Supplement: S8 File — (ZIP) [file pone.0179859.s010.zip › Supplementary Images 4C/3b_L1_60000x_c5_m2.jpg]

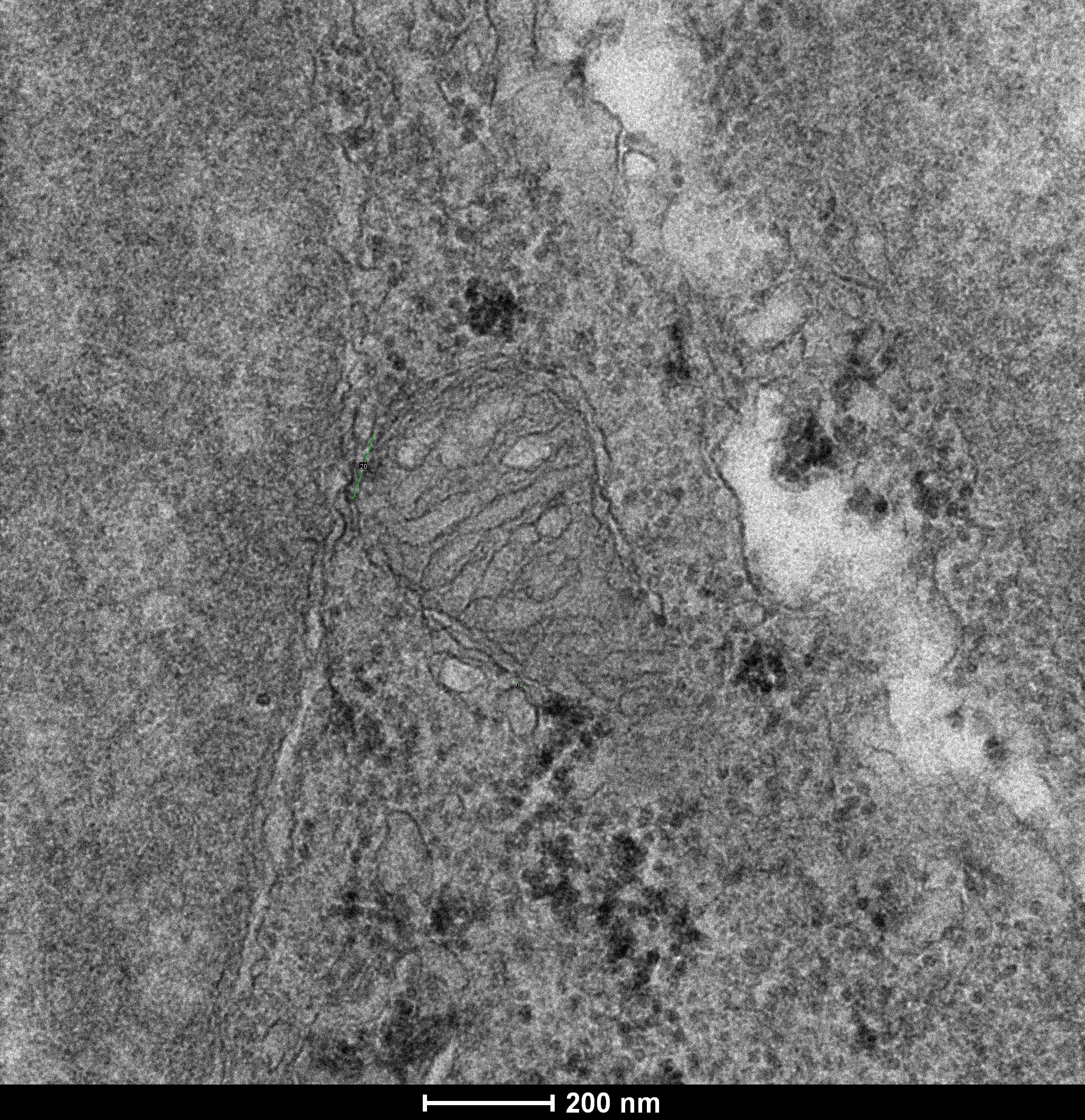

Supplement: S8 File — (ZIP) [file pone.0179859.s010.zip › Supplementary Images 4C/3b_L1_60000x_c8_m1.jpg]

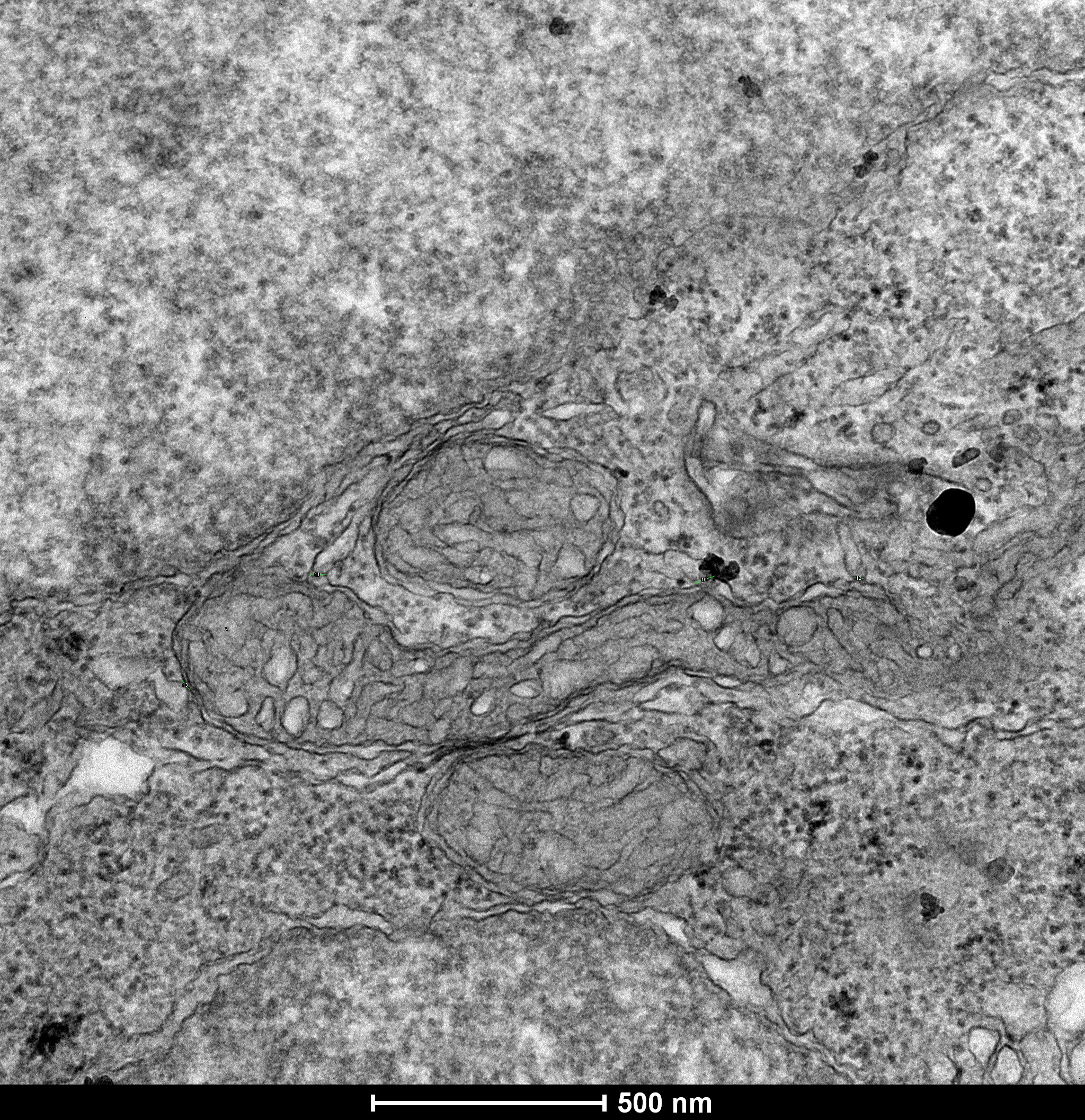

Supplement: S8 File — (ZIP) [file pone.0179859.s010.zip › Supplementary Images 4C/3c_L1_43000x_c2_m1_m2.jpg]

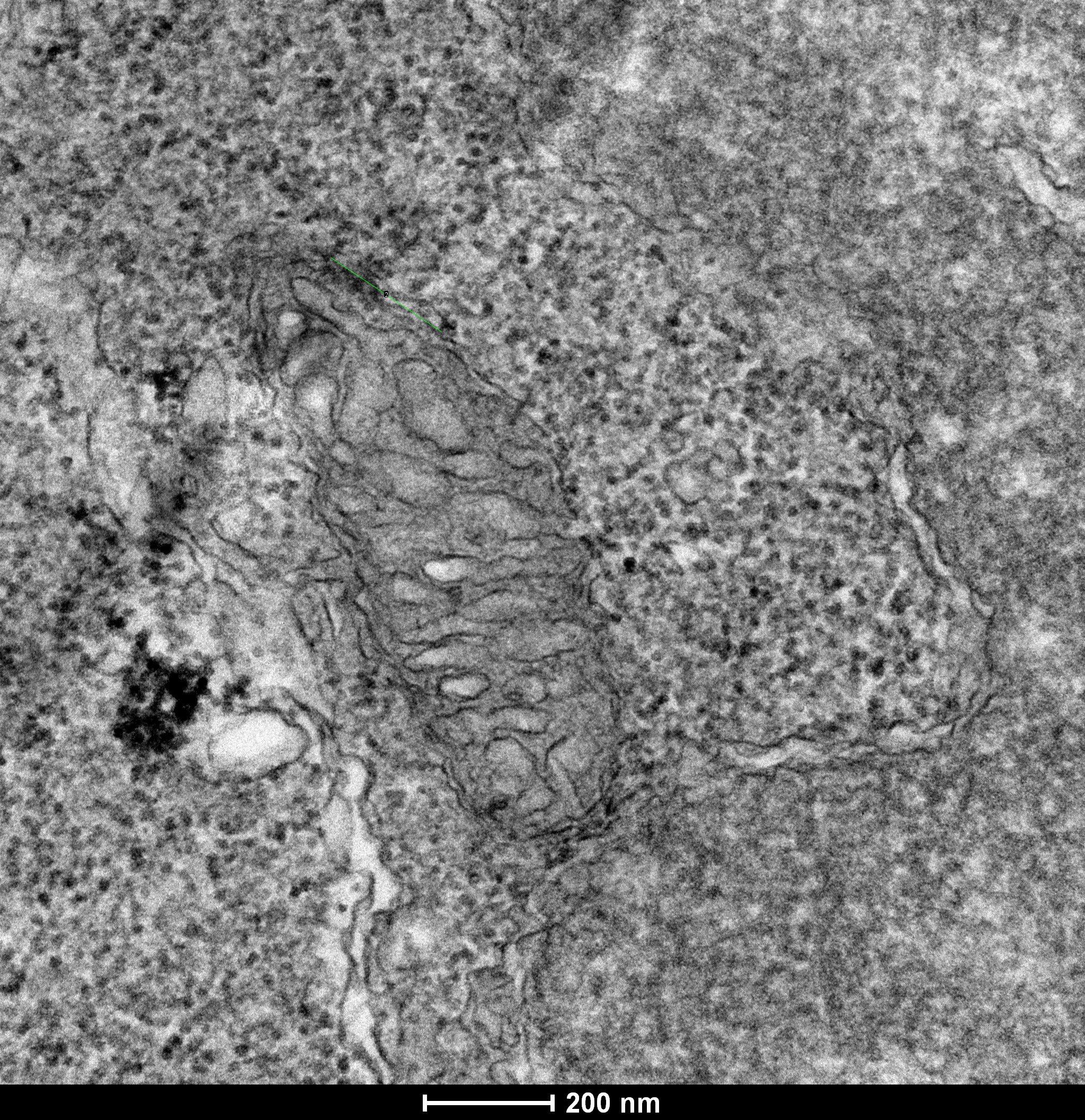

Supplement: S8 File — (ZIP) [file pone.0179859.s010.zip › Supplementary Images 4C/3c_L1_60000x_c1_m1.jpg]

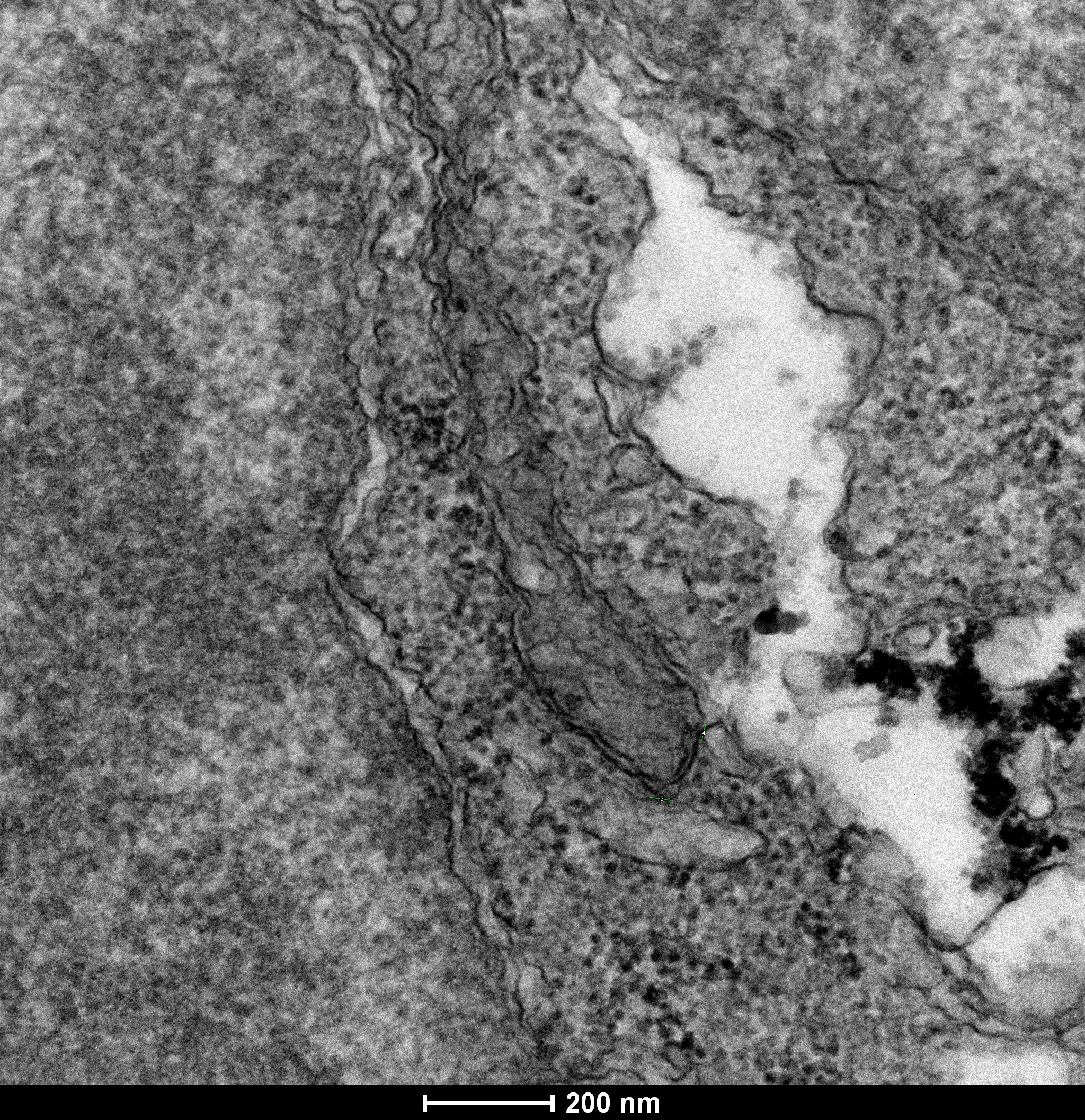

Supplement: S8 File — (ZIP) [file pone.0179859.s010.zip › Supplementary Images 4C/3c_L1_60000x_c1_m2.jpg]

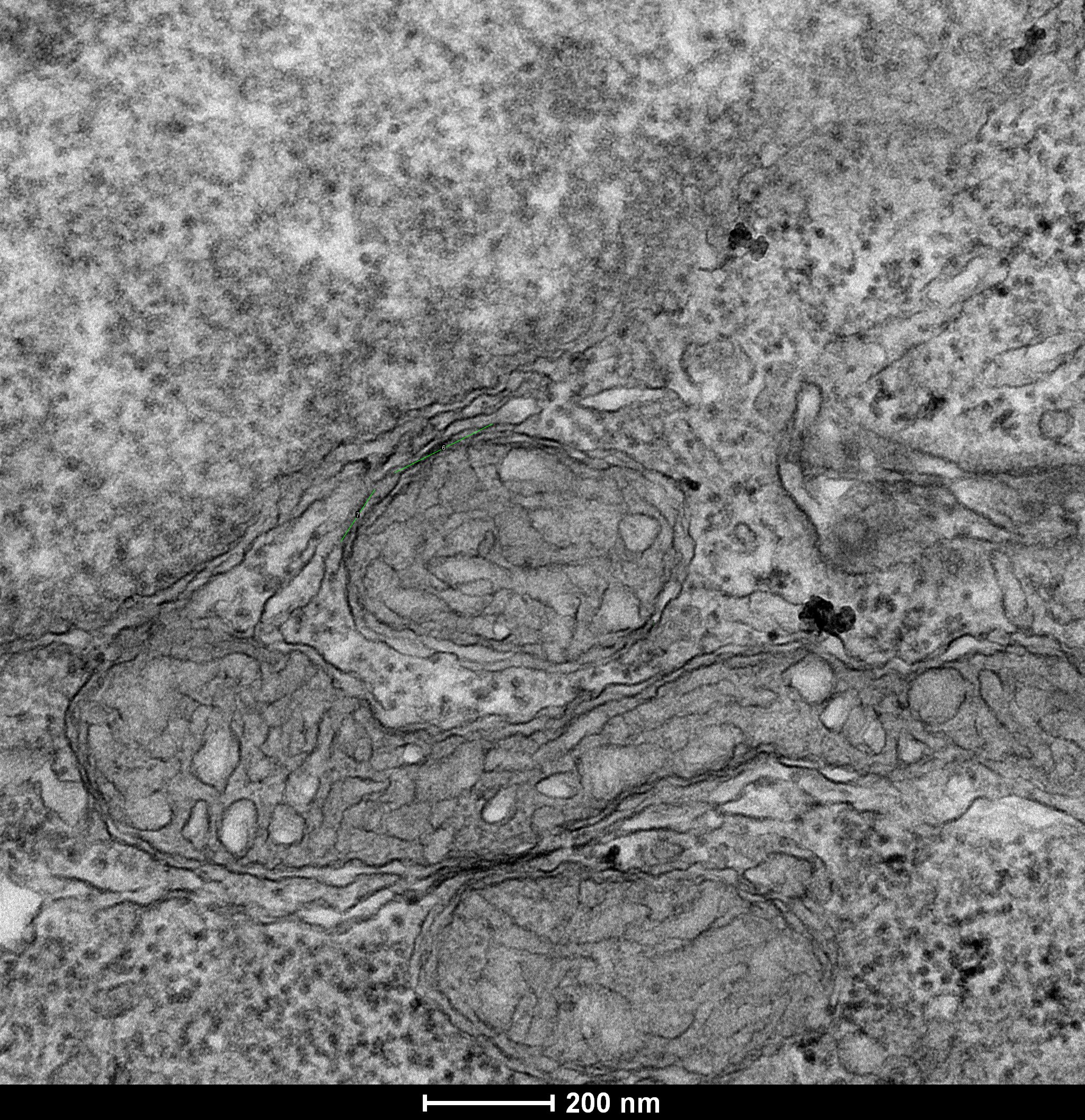

Supplement: S8 File — (ZIP) [file pone.0179859.s010.zip › Supplementary Images 4C/3c_L1_60000x_c2_m1.jpg]

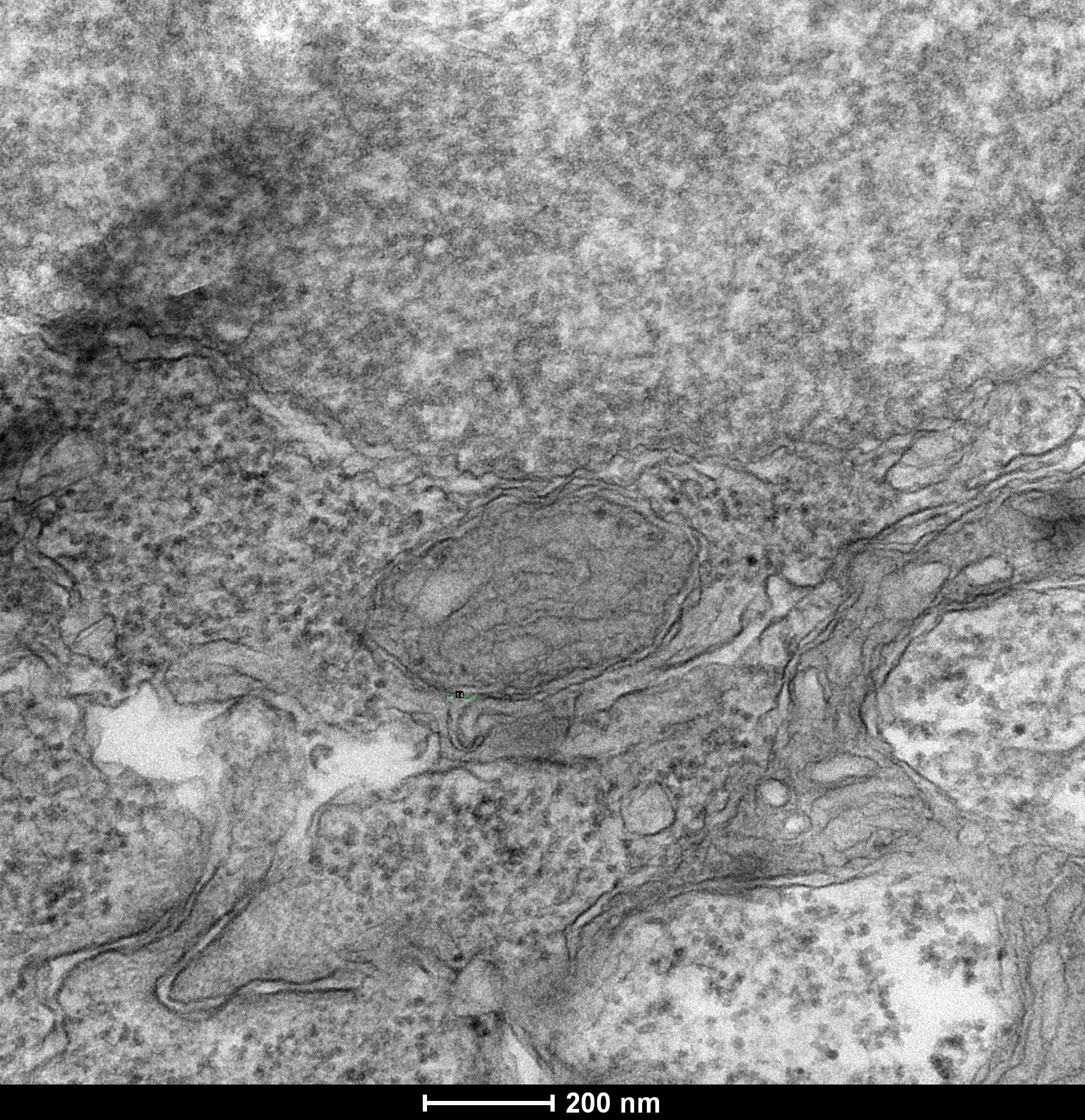

Supplement: S8 File — (ZIP) [file pone.0179859.s010.zip › Supplementary Images 4C/3c_L1_60000x_c3_m1.jpg]

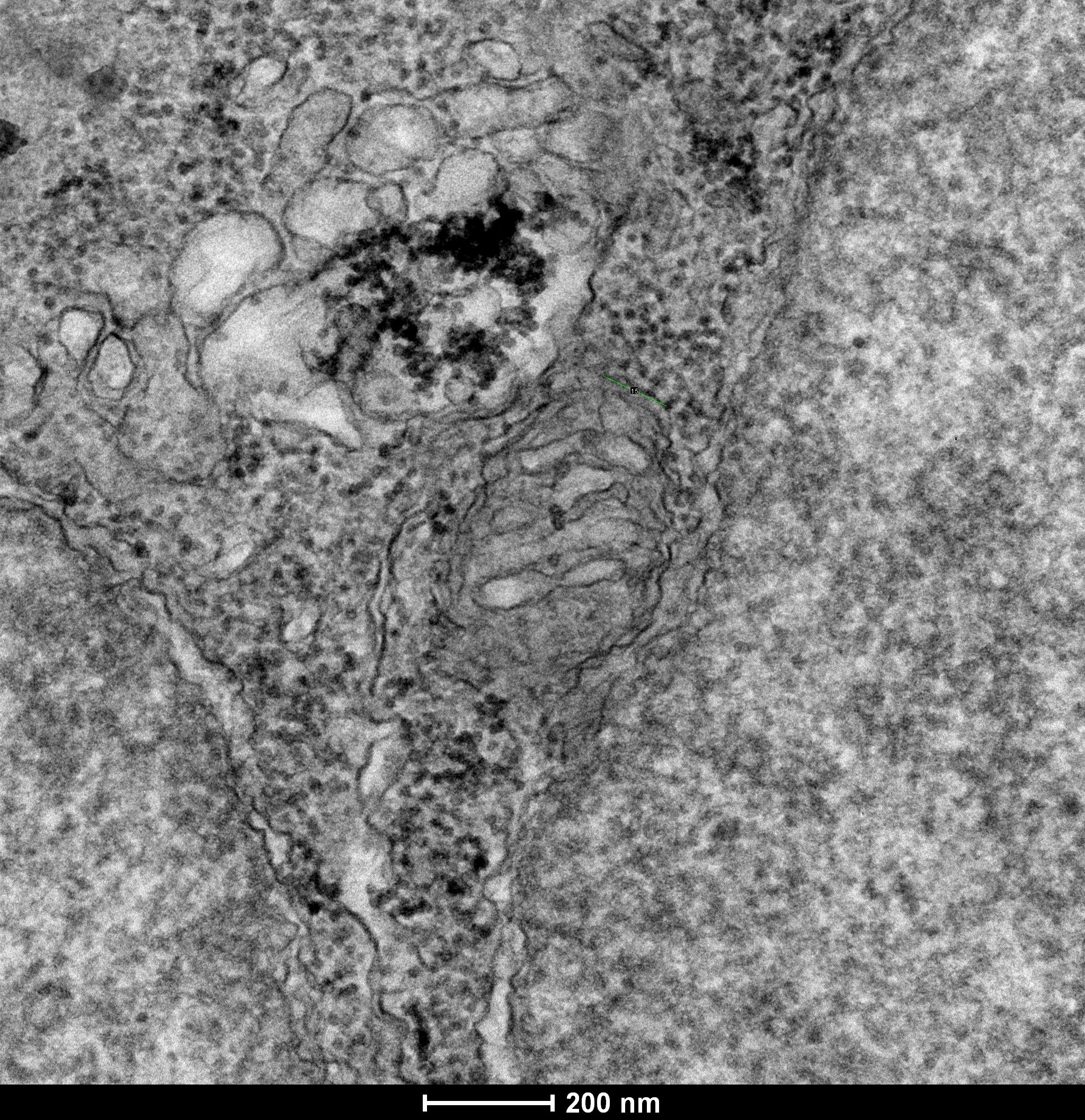

Supplement: S8 File — (ZIP) [file pone.0179859.s010.zip › Supplementary Images 4C/3c_L1_60000x_c4_m1.jpg]

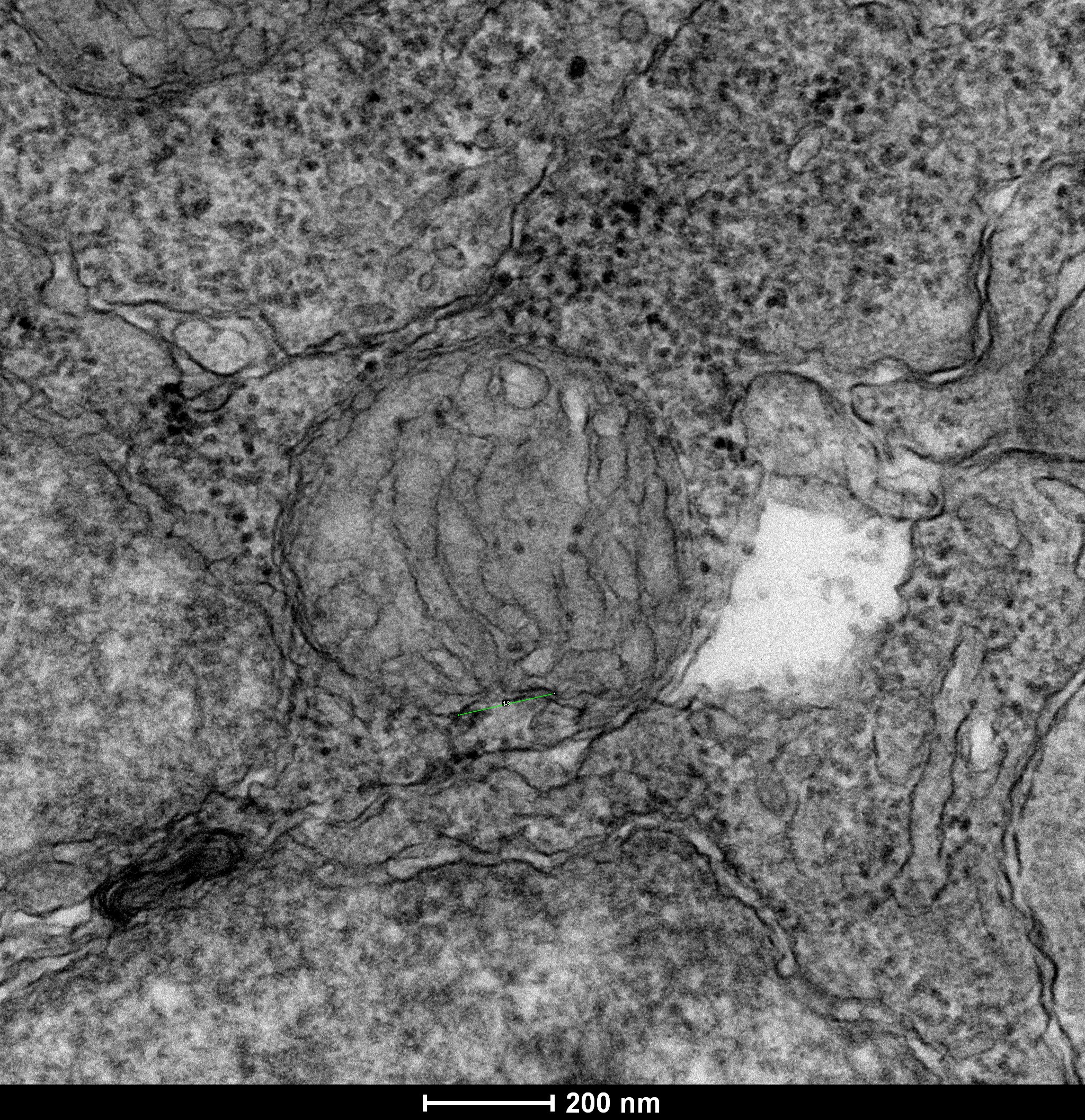

Supplement: S8 File — (ZIP) [file pone.0179859.s010.zip › Supplementary Images 4C/3c_L1_60000x_c4_m3.jpg]

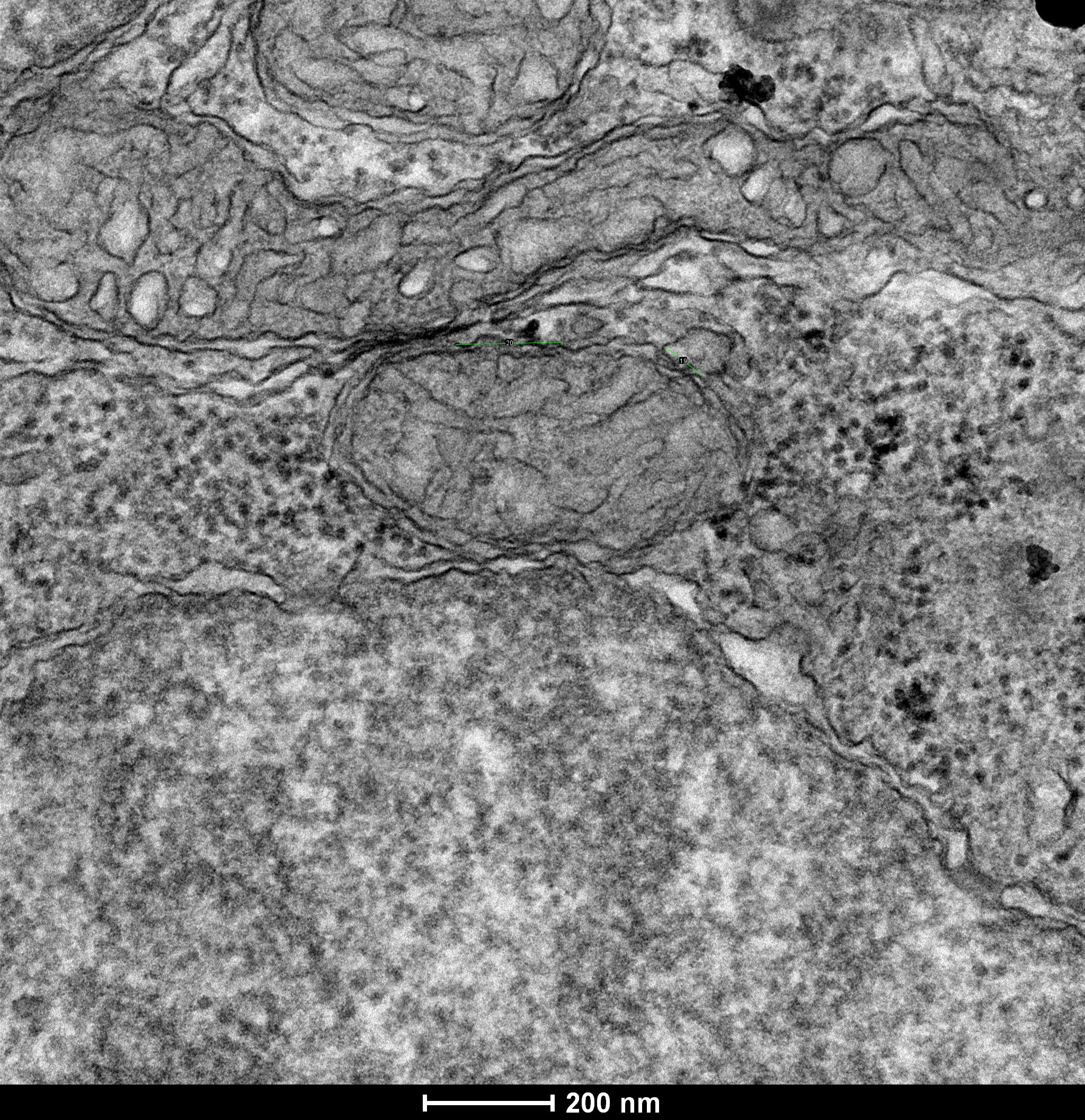

Supplement: S8 File — (ZIP) [file pone.0179859.s010.zip › Supplementary Images 4C/3c_L1_60000x_c5_m1.jpg]

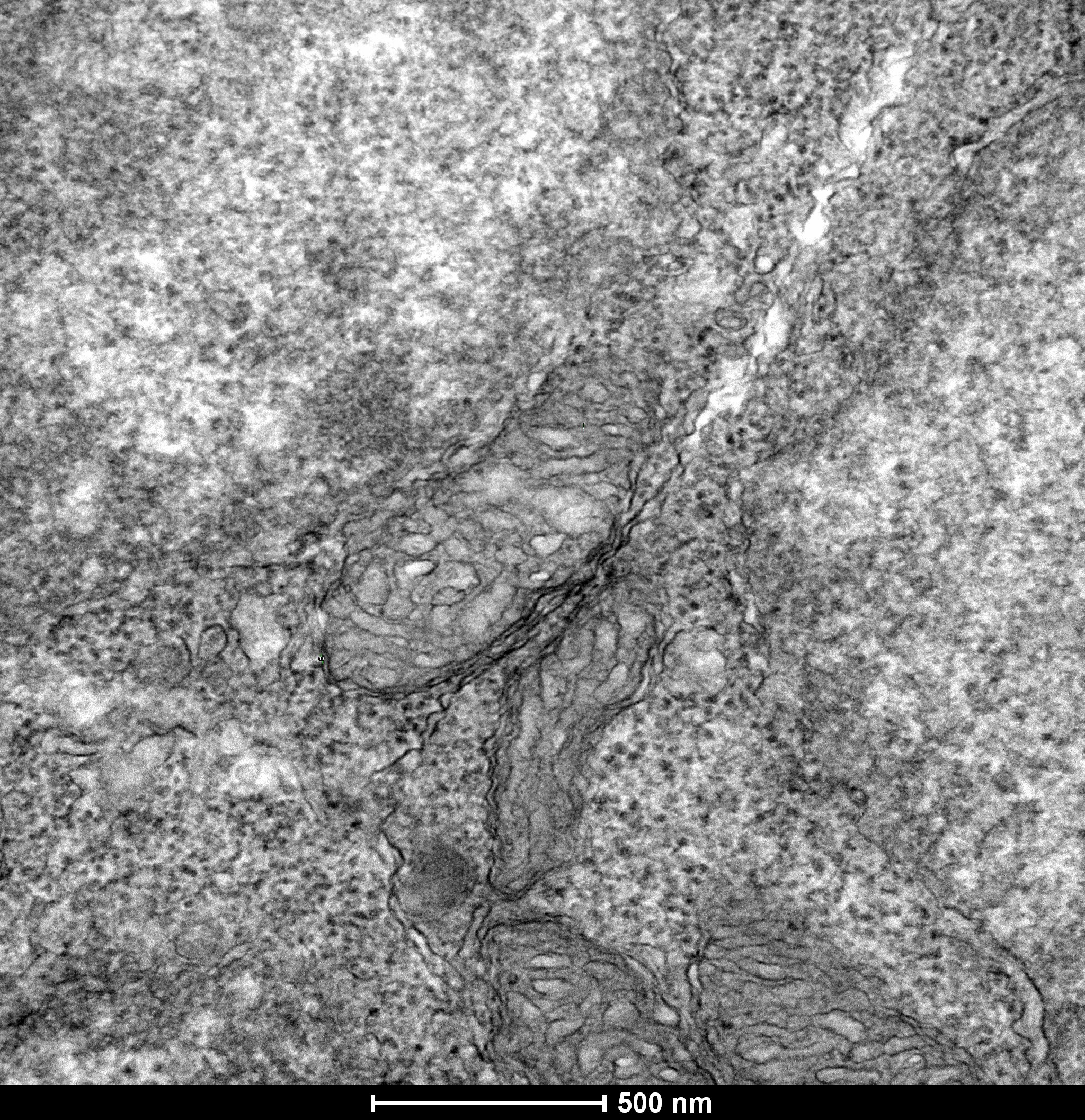

Supplement: S9 File — (ZIP) [file pone.0179859.s011.zip › Supplementary Images 4D/4c_L1_43000x_c6_m1.jpg]

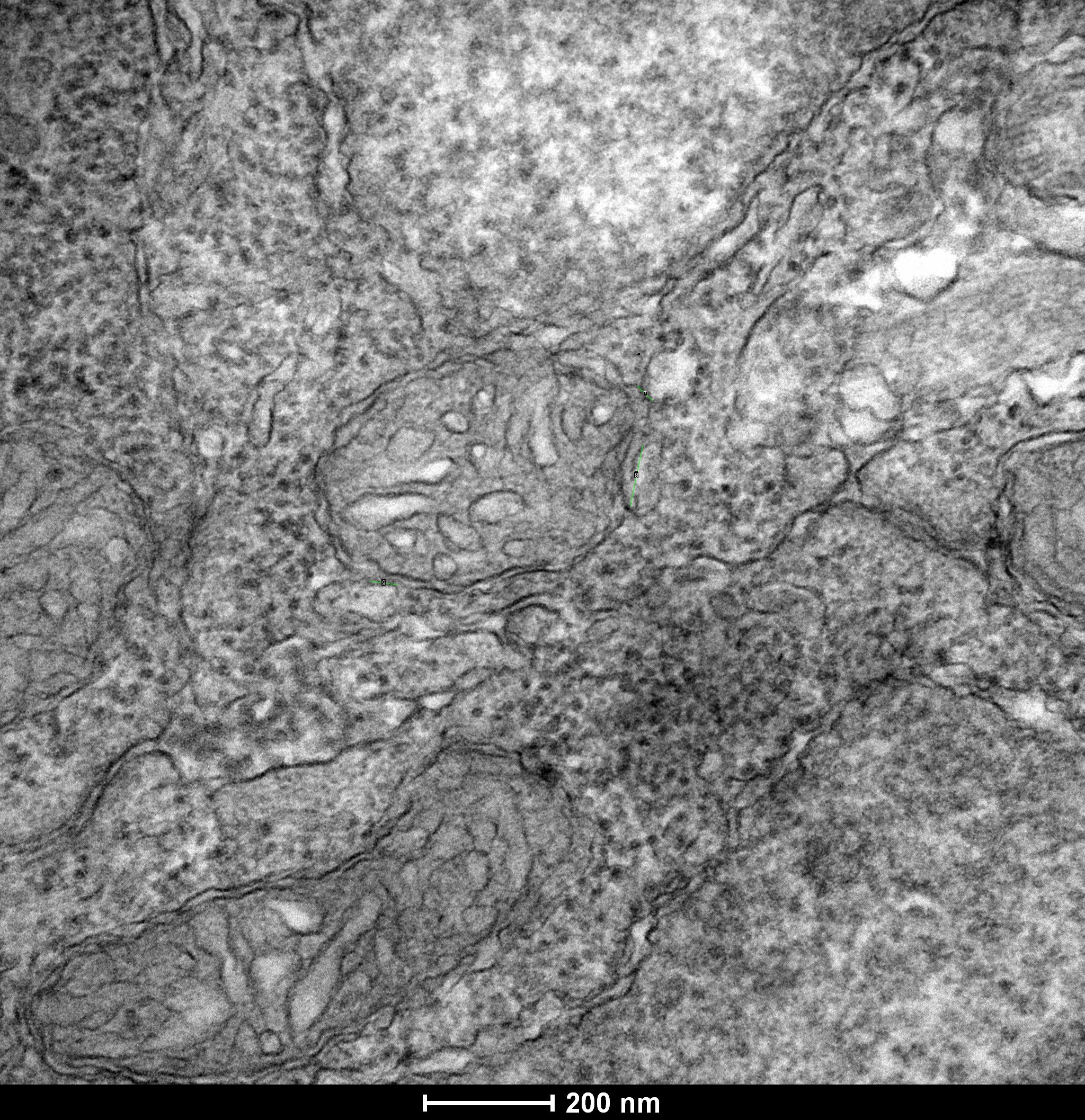

Supplement: S9 File — (ZIP) [file pone.0179859.s011.zip › Supplementary Images 4D/4c_L1_60000x_c1_m1-1.jpg]

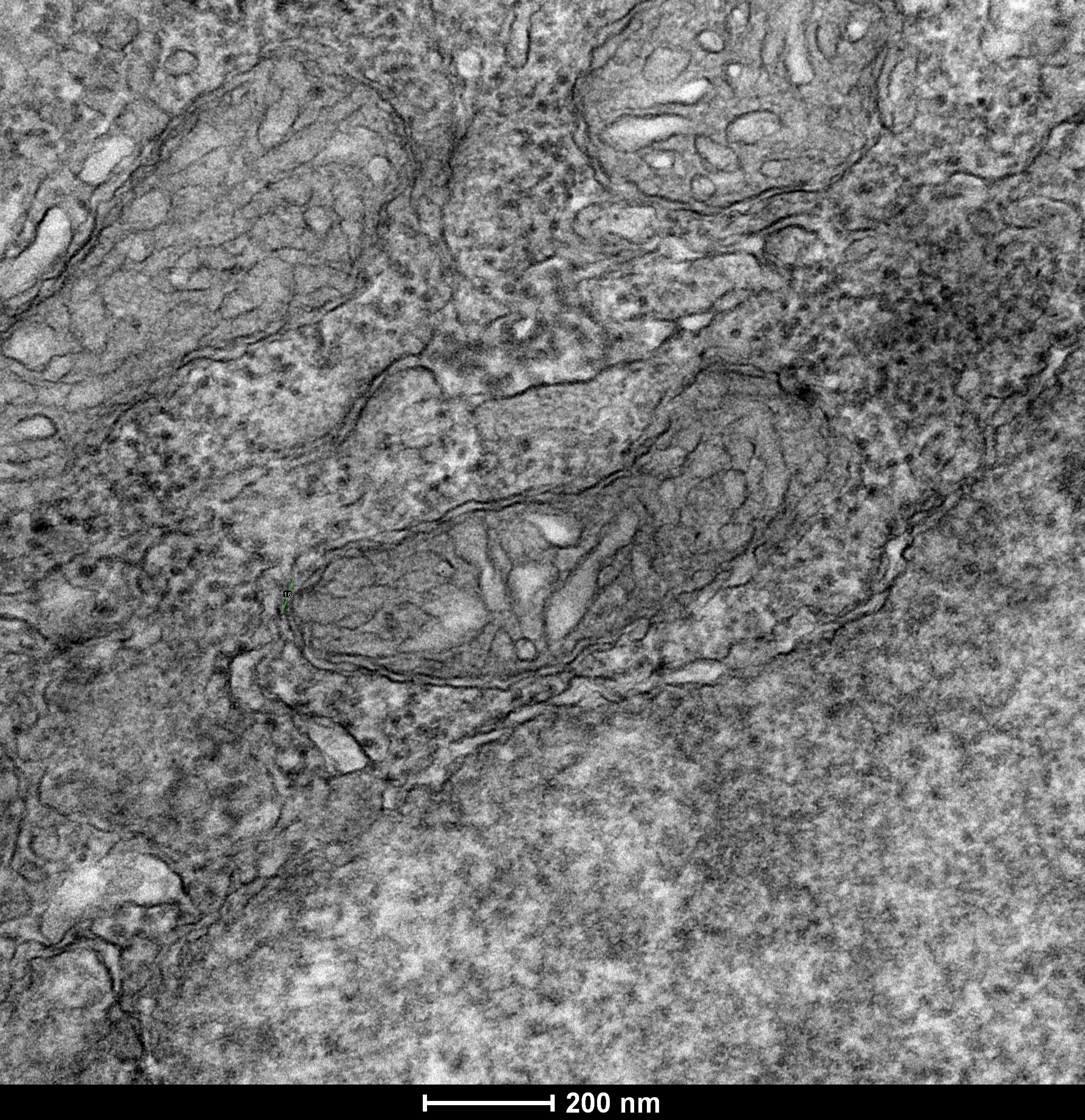

Supplement: S9 File — (ZIP) [file pone.0179859.s011.zip › Supplementary Images 4D/4c_L1_60000x_c2_m1.jpg]

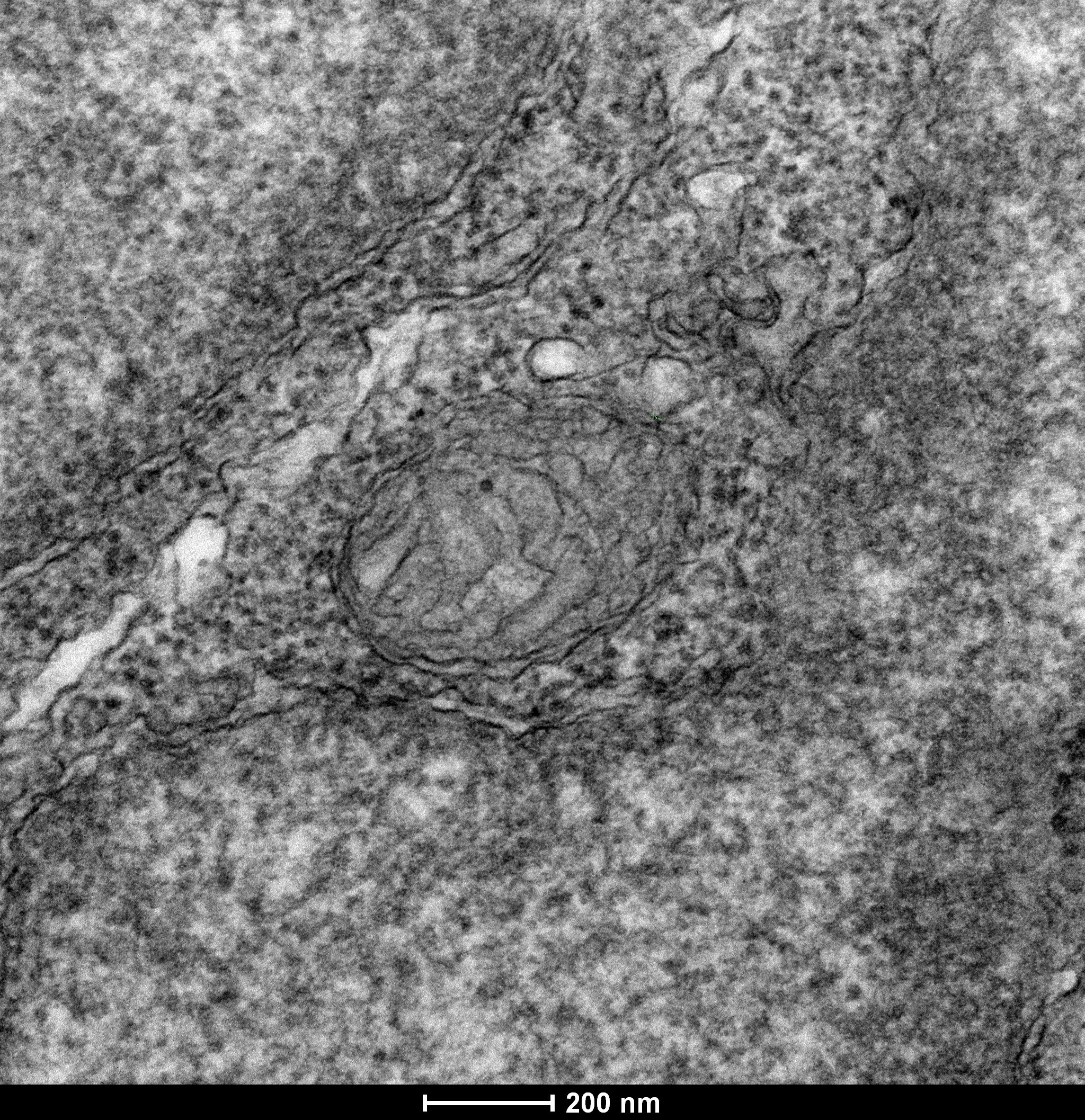

Supplement: S9 File — (ZIP) [file pone.0179859.s011.zip › Supplementary Images 4D/4c_L1_60000x_c4_m1.jpg]

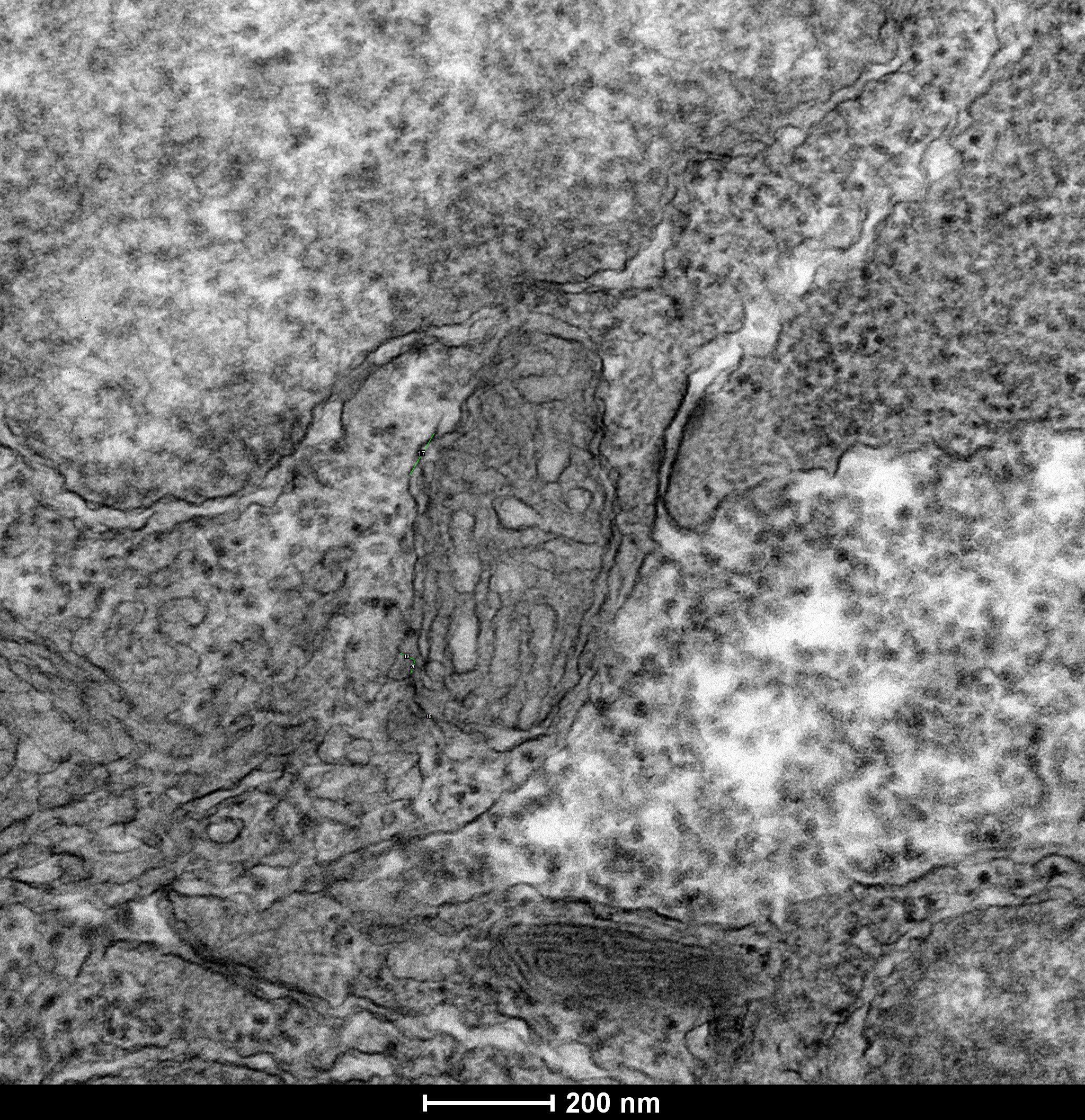

Supplement: S9 File — (ZIP) [file pone.0179859.s011.zip › Supplementary Images 4D/4c_L1_60000x_c4_m2.jpg]

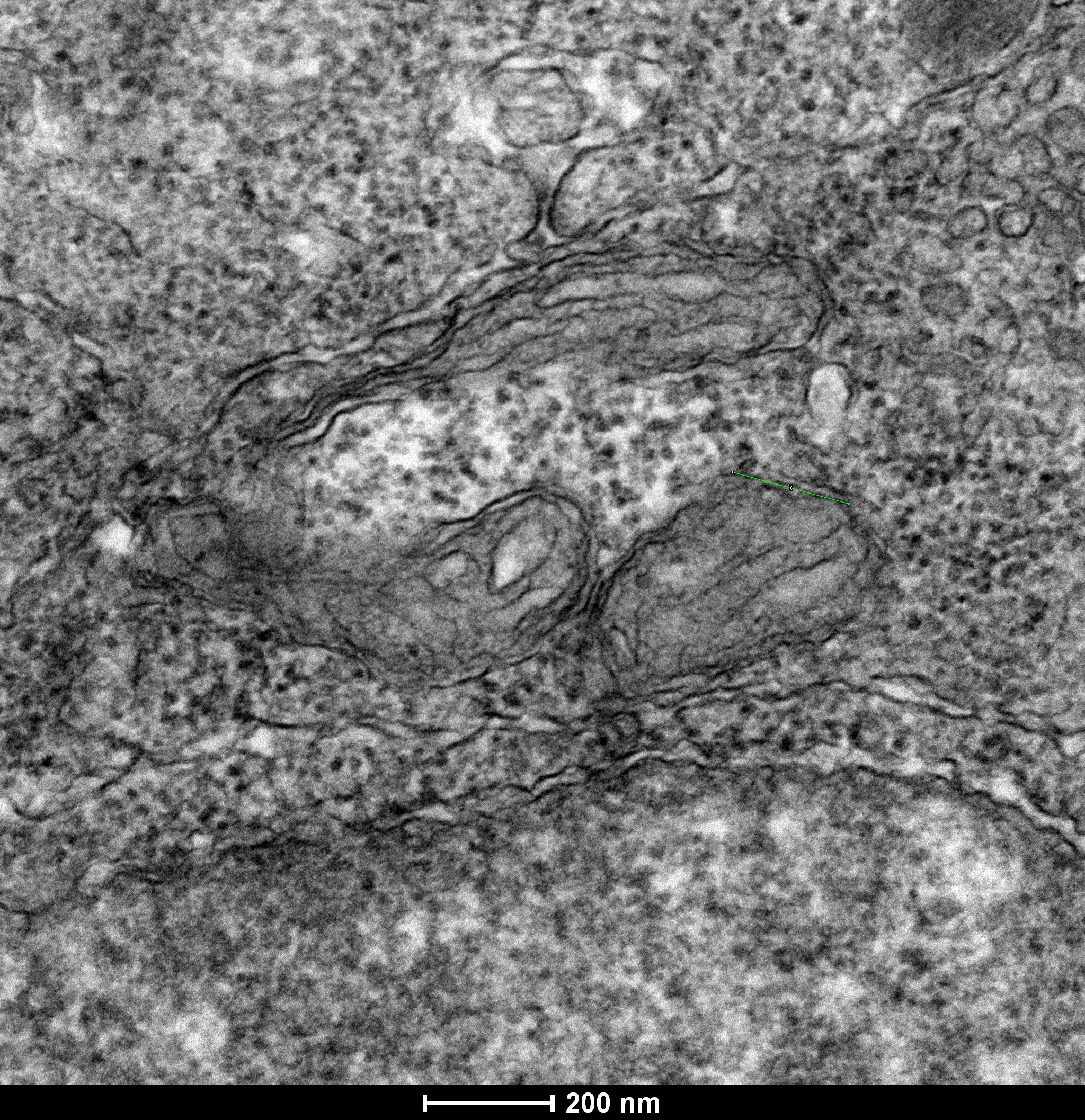

Supplement: S9 File — (ZIP) [file pone.0179859.s011.zip › Supplementary Images 4D/4c_L1_60000x_c5_m1_m2_m3.jpg]

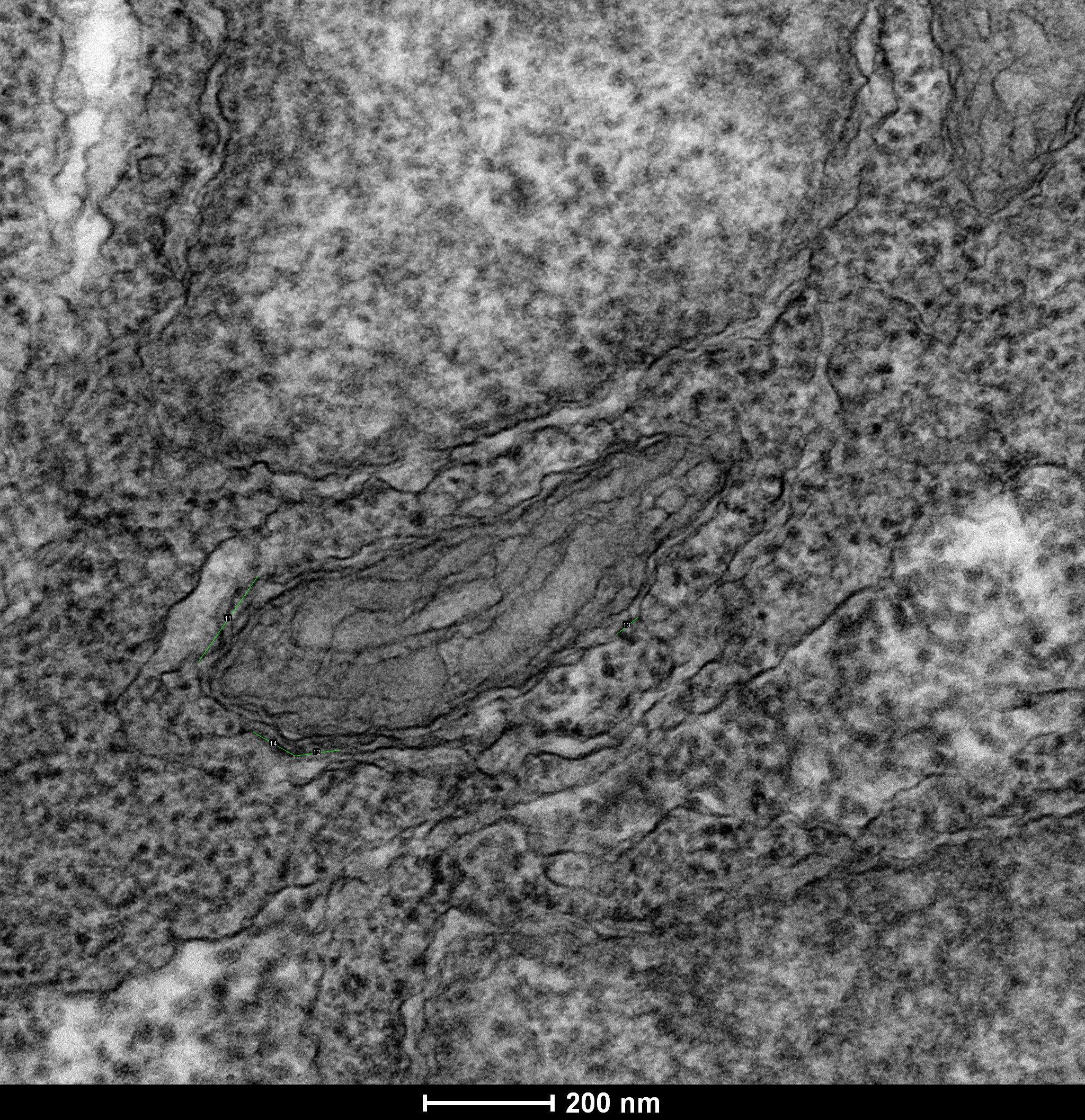

Supplement: S9 File — (ZIP) [file pone.0179859.s011.zip › Supplementary Images 4D/4c_L1_60000x_c7_m1.jpg]

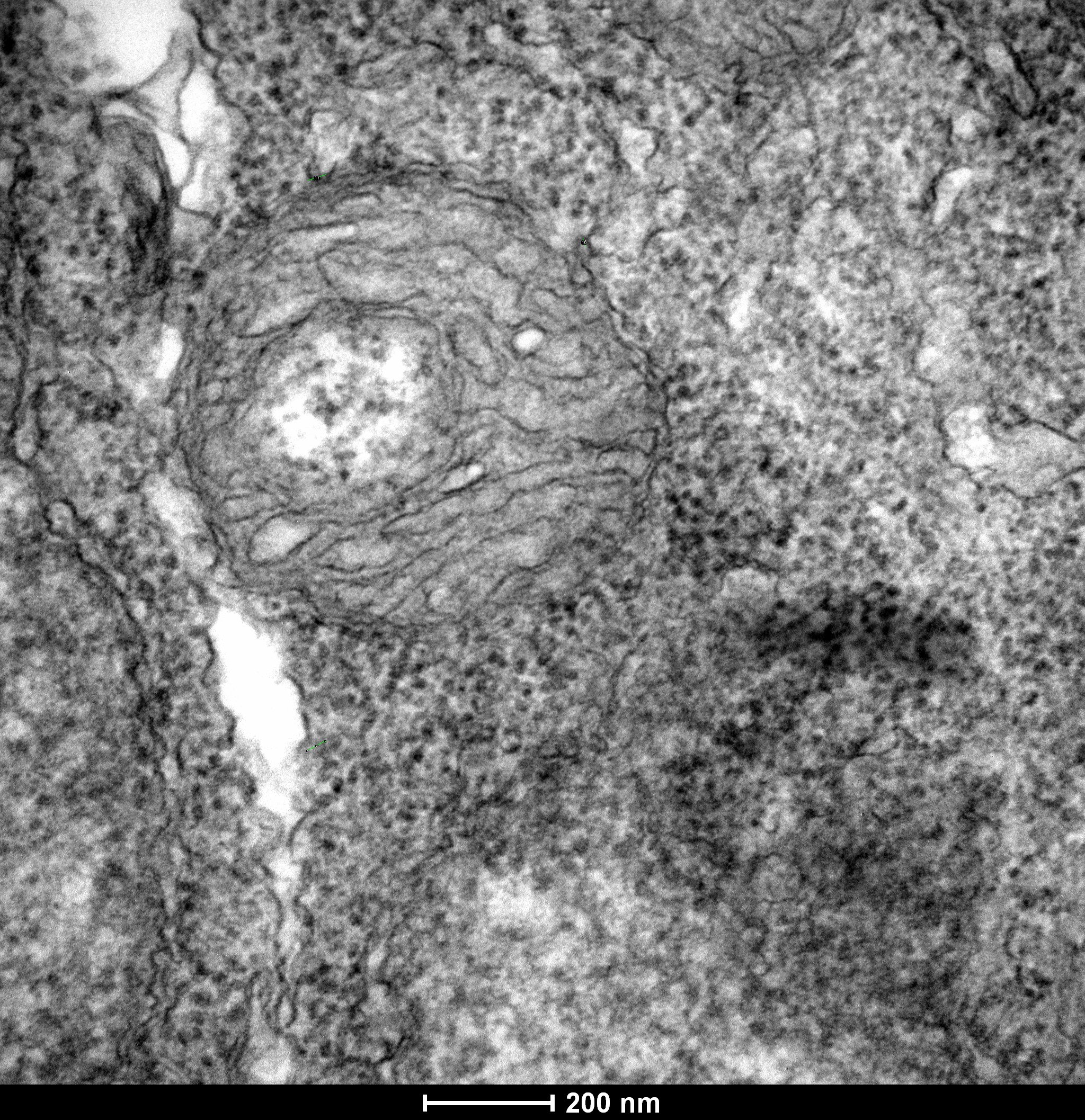

Supplement: S9 File — (ZIP) [file pone.0179859.s011.zip › Supplementary Images 4D/4c_L1_60000x_c7_m3.jpg]

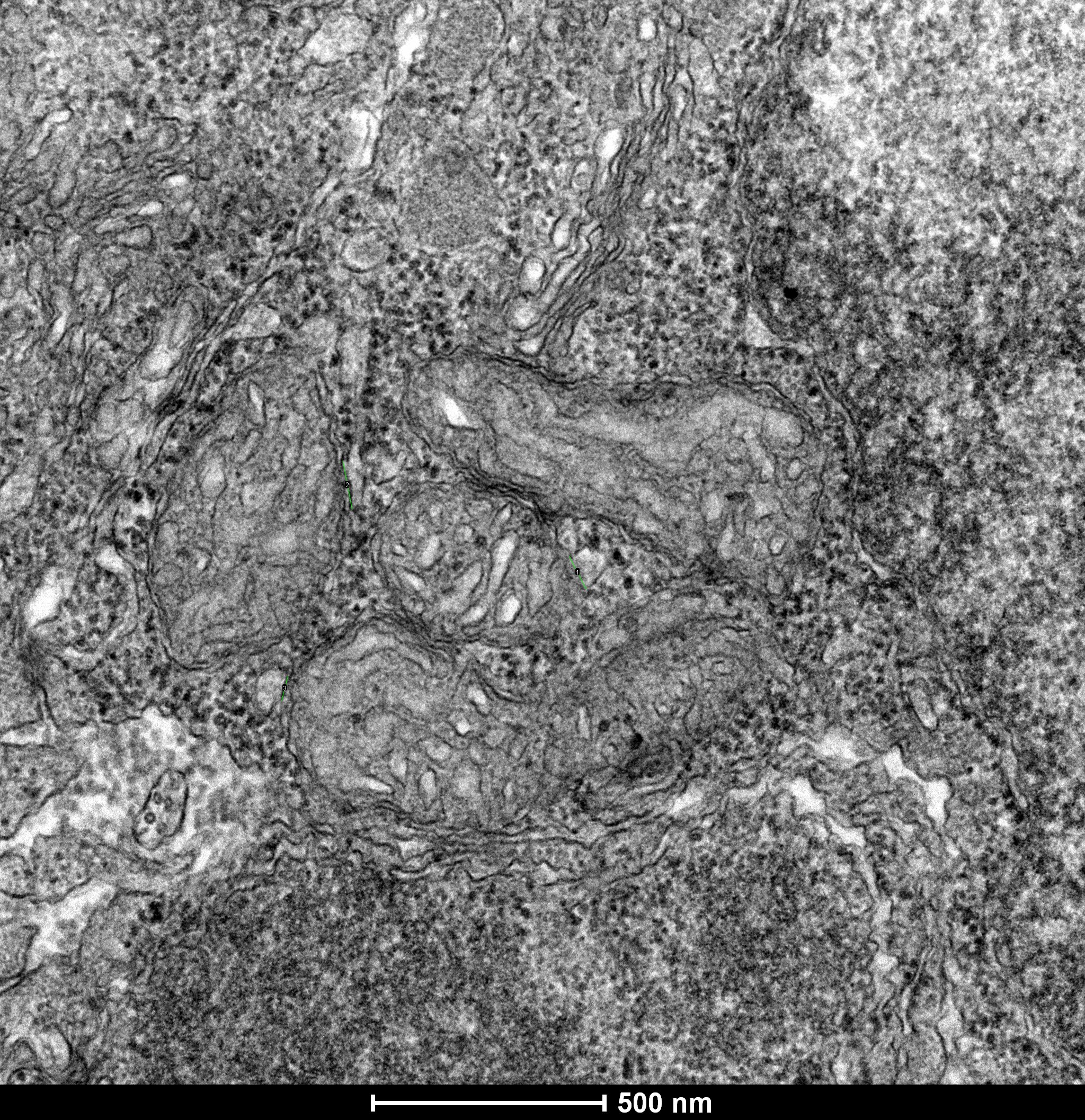

Supplement: S9 File — (ZIP) [file pone.0179859.s011.zip › Supplementary Images 4D/4d_L1_43000x_c3_m1_m2_m3_m4.jpg]

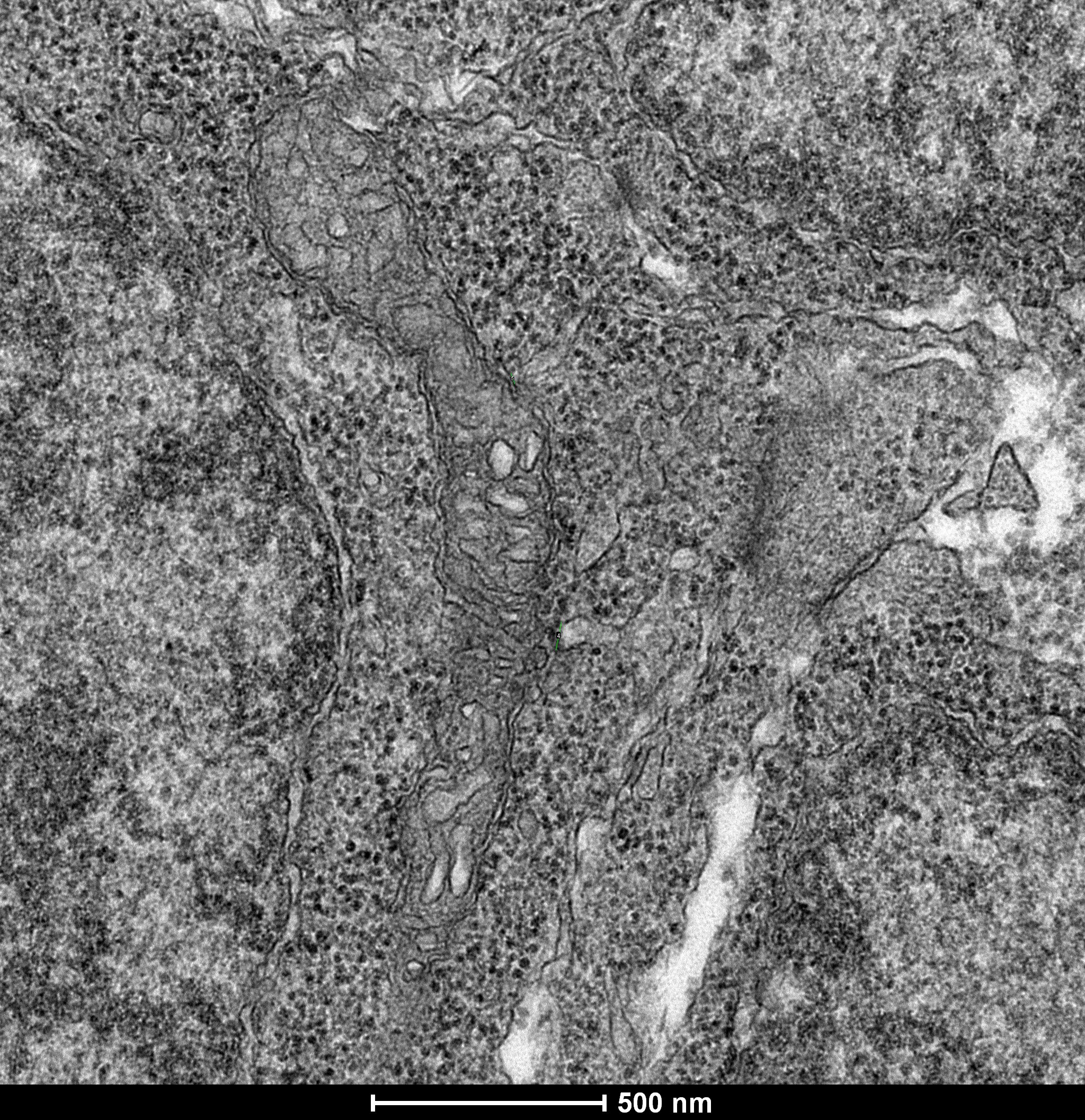

Supplement: S9 File — (ZIP) [file pone.0179859.s011.zip › Supplementary Images 4D/4d_L1_43000x_c6_m1.jpg]

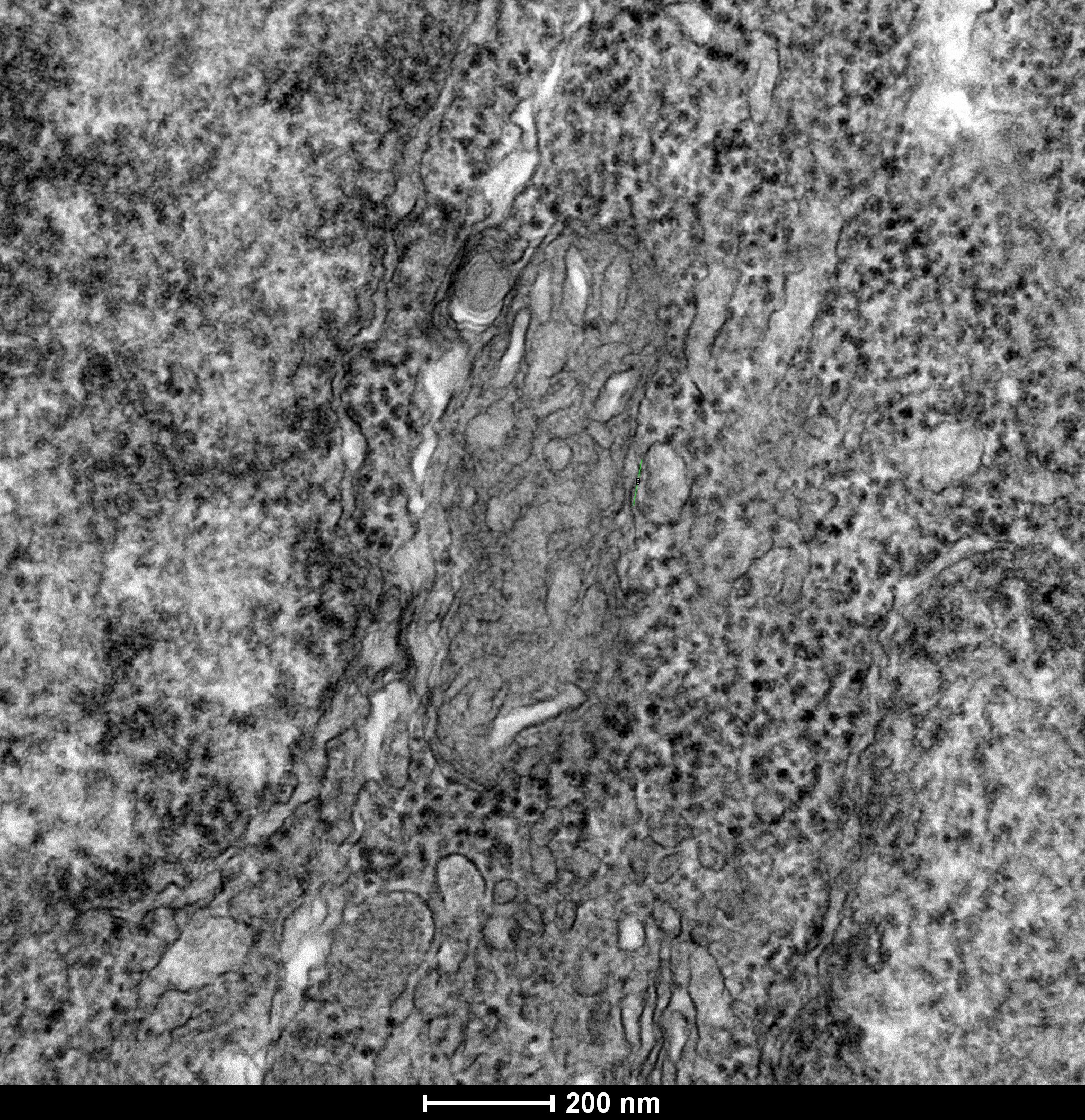

Supplement: S9 File — (ZIP) [file pone.0179859.s011.zip › Supplementary Images 4D/4d_L1_60000x_c3_m5.jpg]

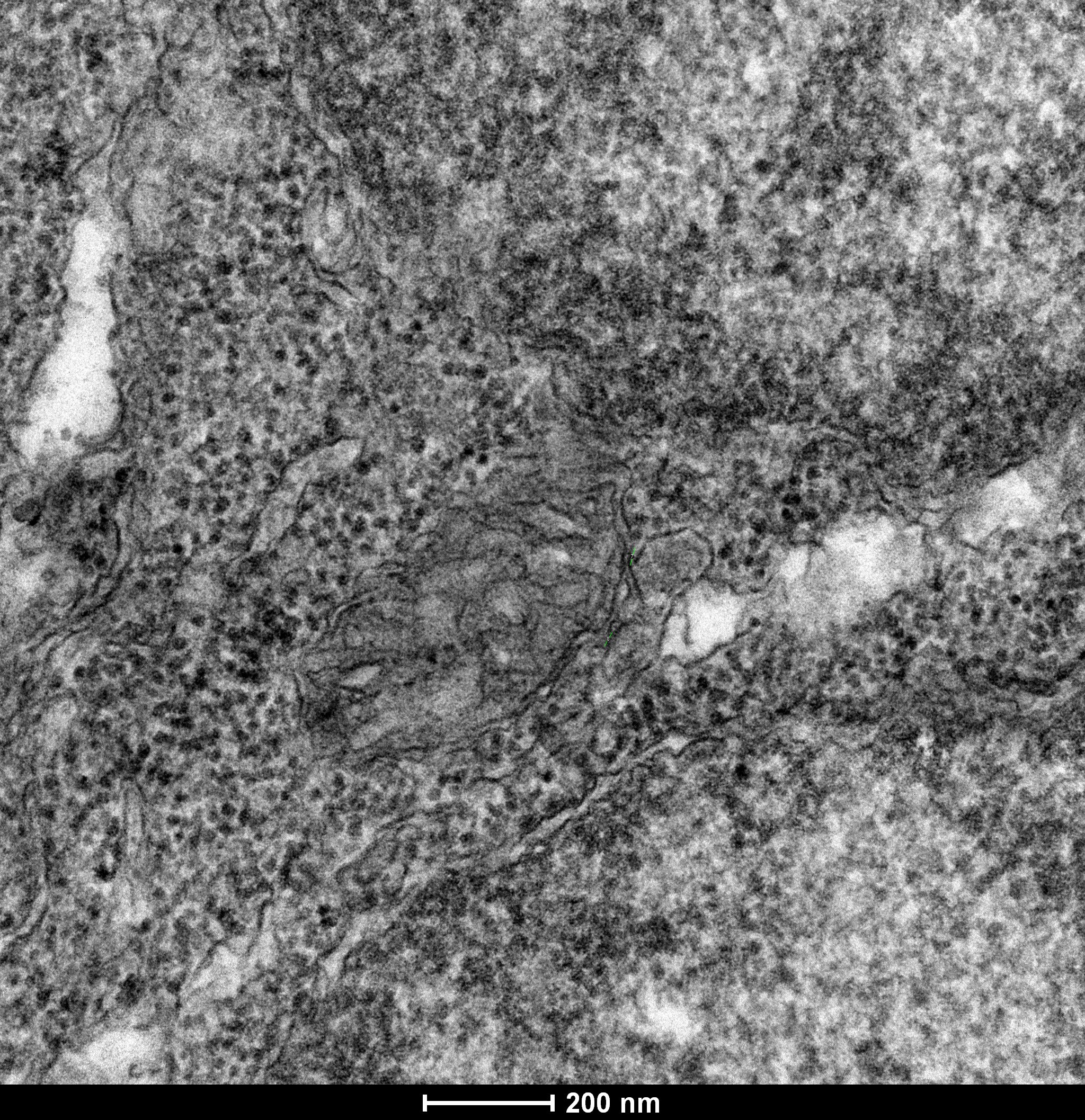

Supplement: S9 File — (ZIP) [file pone.0179859.s011.zip › Supplementary Images 4D/4d_L1_60000x_c5_m1.jpg]

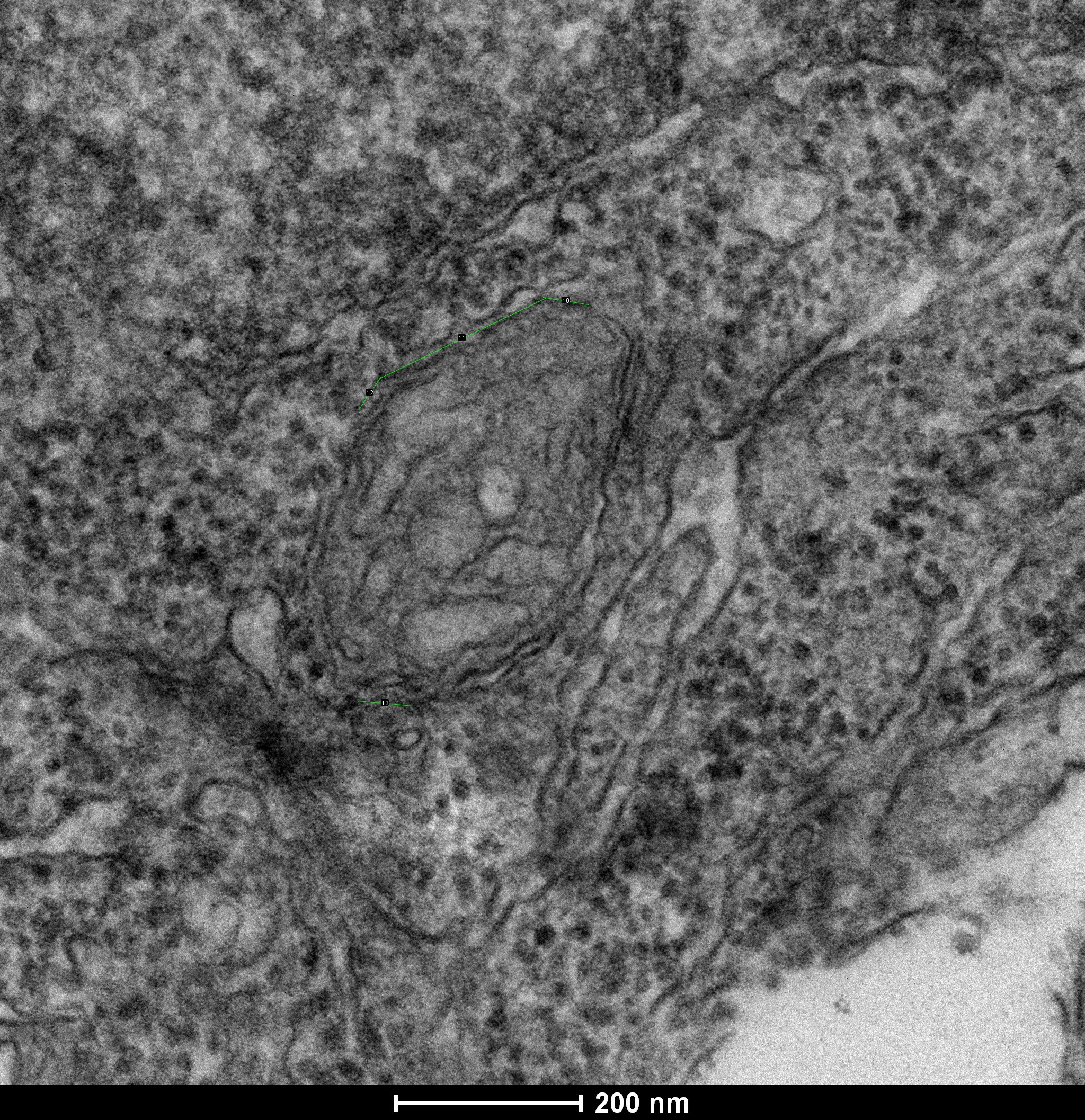

Supplement: S9 File — (ZIP) [file pone.0179859.s011.zip › Supplementary Images 4D/4d_L1_87000x_c4_m1.jpg]

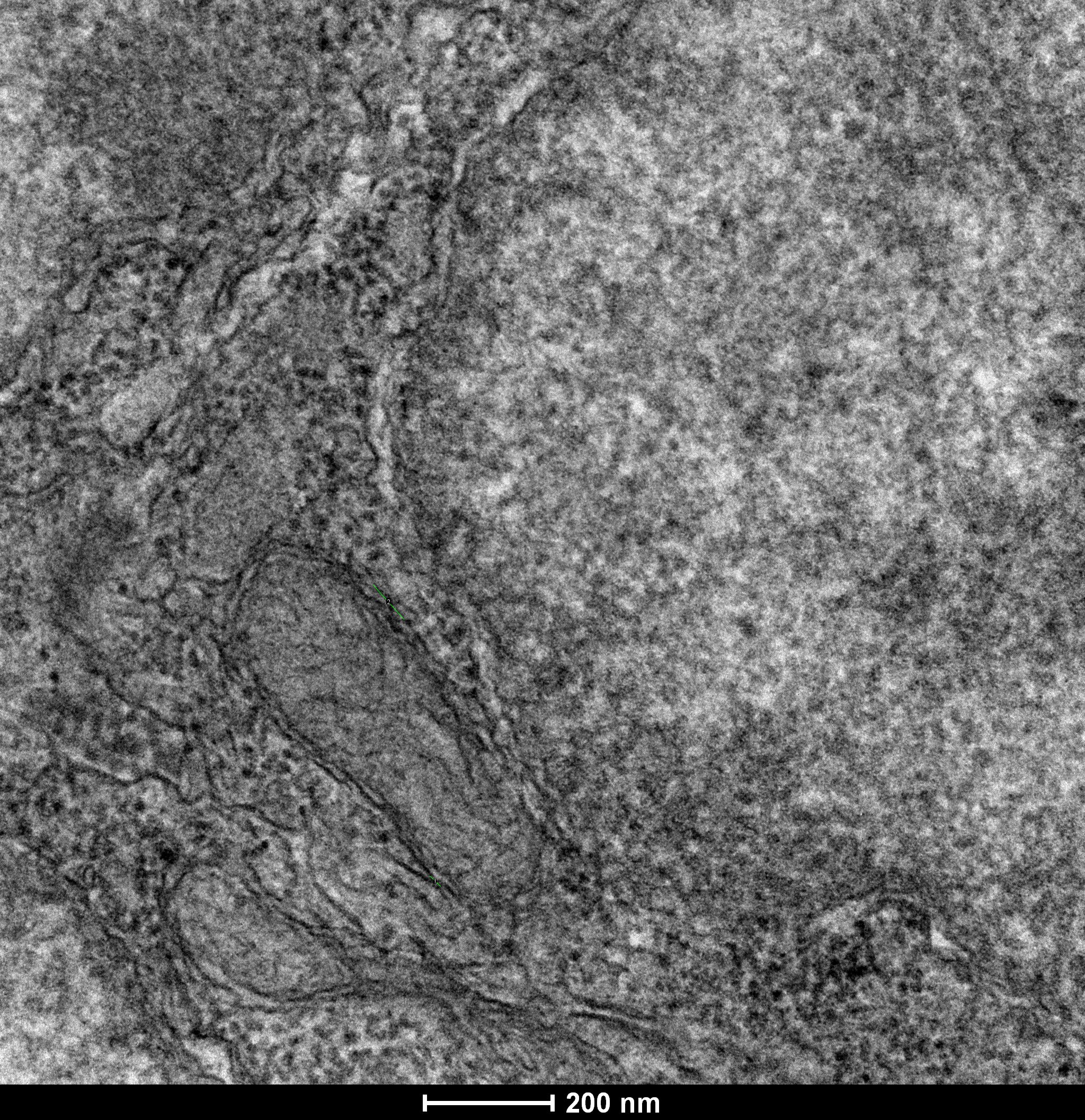

Supplement: S9 File — (ZIP) [file pone.0179859.s011.zip › Supplementary Images 4D/4b_L1_60000x_c1_m1.jpg]

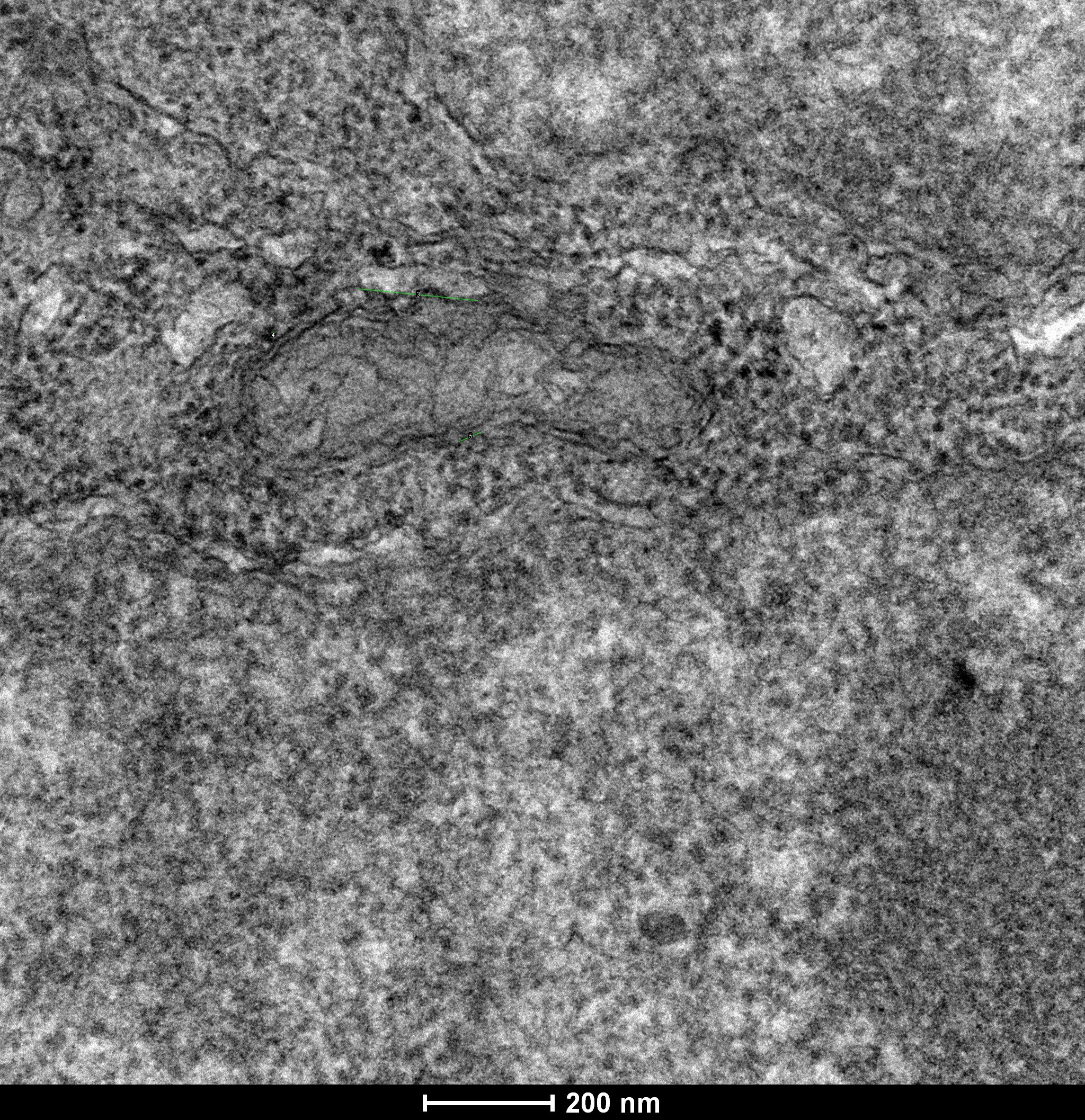

Supplement: S9 File — (ZIP) [file pone.0179859.s011.zip › Supplementary Images 4D/4b_L1_60000x_c1_m2.jpg]

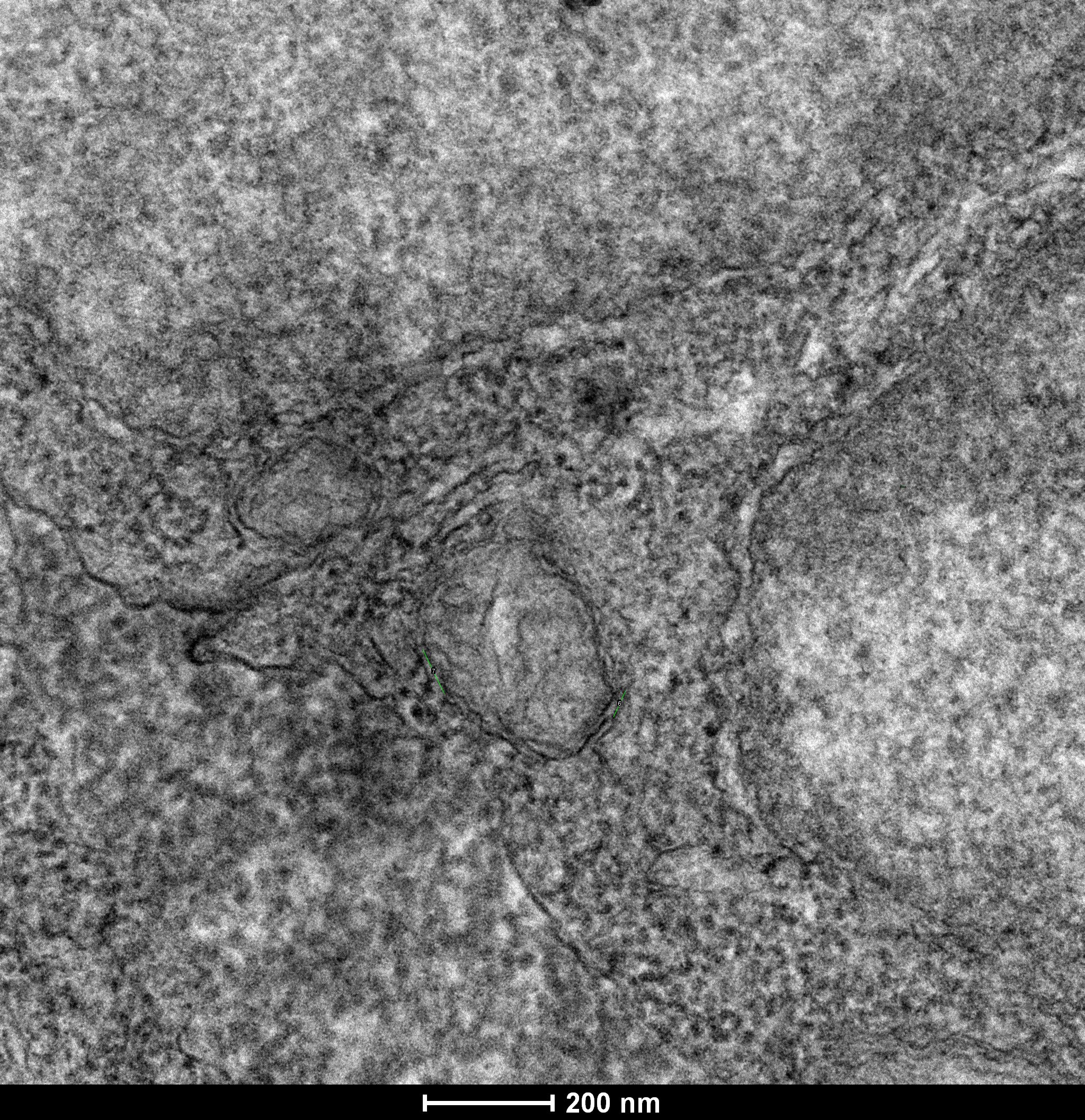

Supplement: S9 File — (ZIP) [file pone.0179859.s011.zip › Supplementary Images 4D/4b_L1_60000x_c3_m1.jpg]

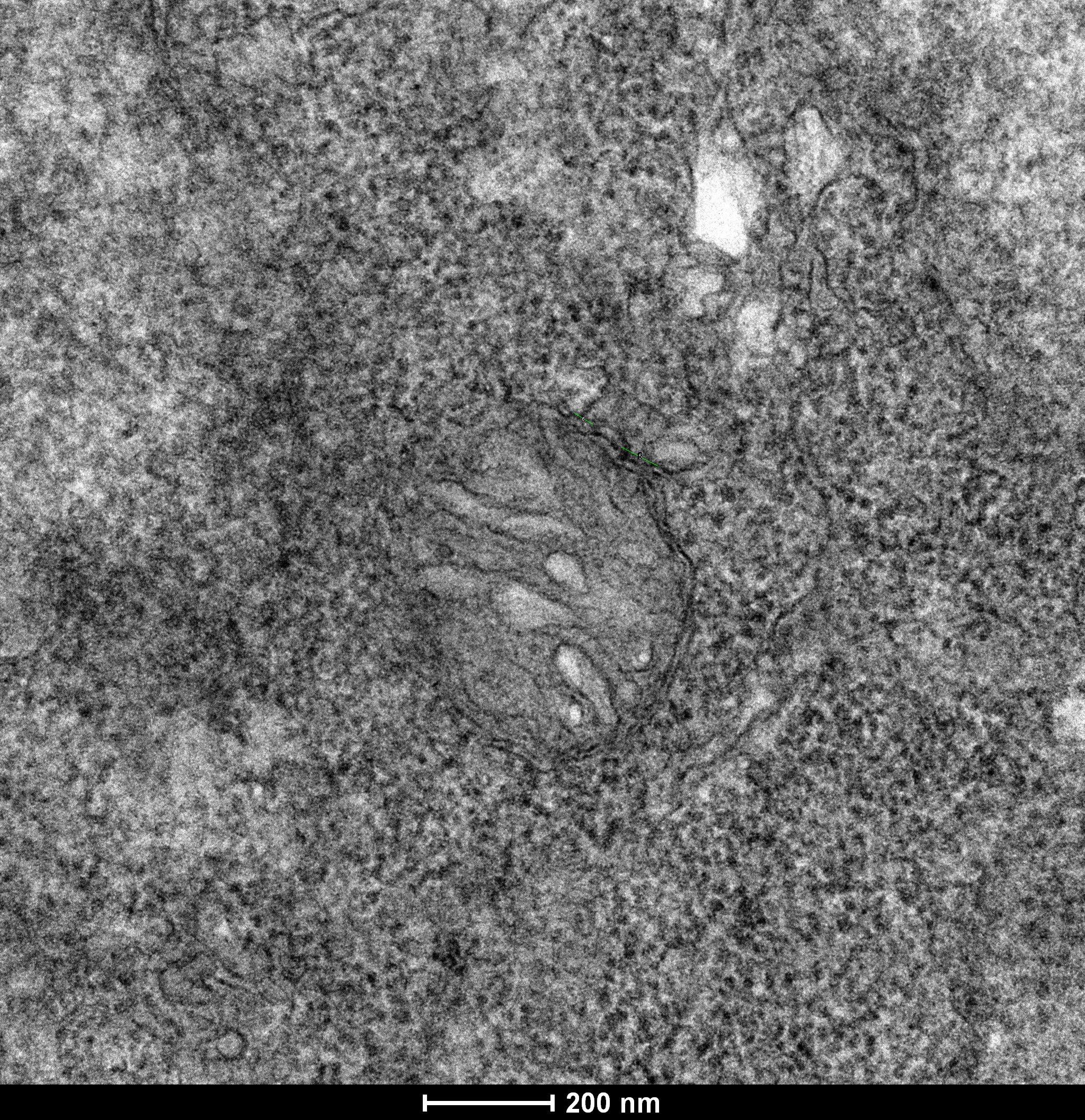

Supplement: S9 File — (ZIP) [file pone.0179859.s011.zip › Supplementary Images 4D/4b_L1_60000x_c5_m1.jpg]

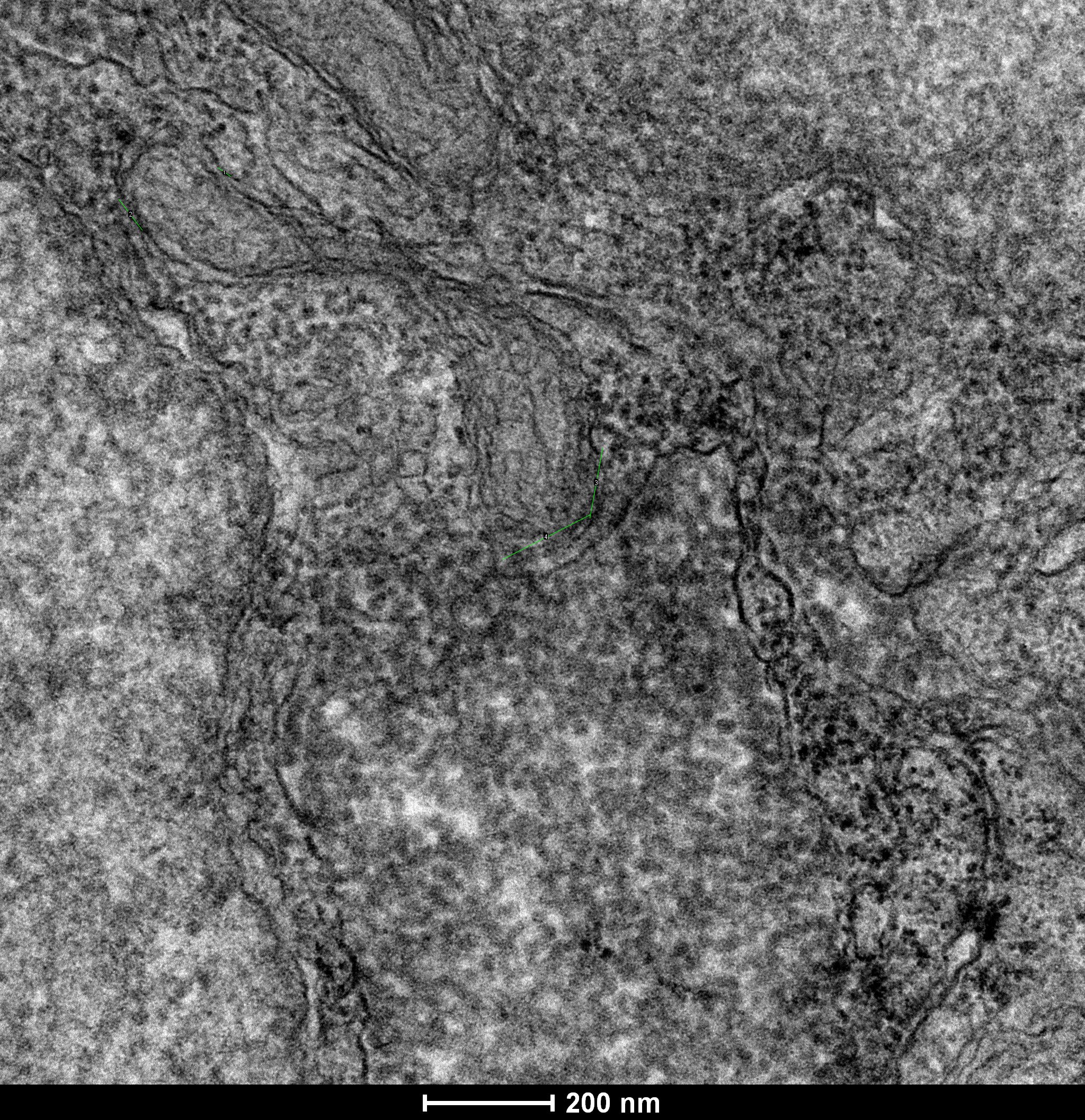

Supplement: S9 File — (ZIP) [file pone.0179859.s011.zip › Supplementary Images 4D/4b_L1_60000x_c7_m1.jpg]

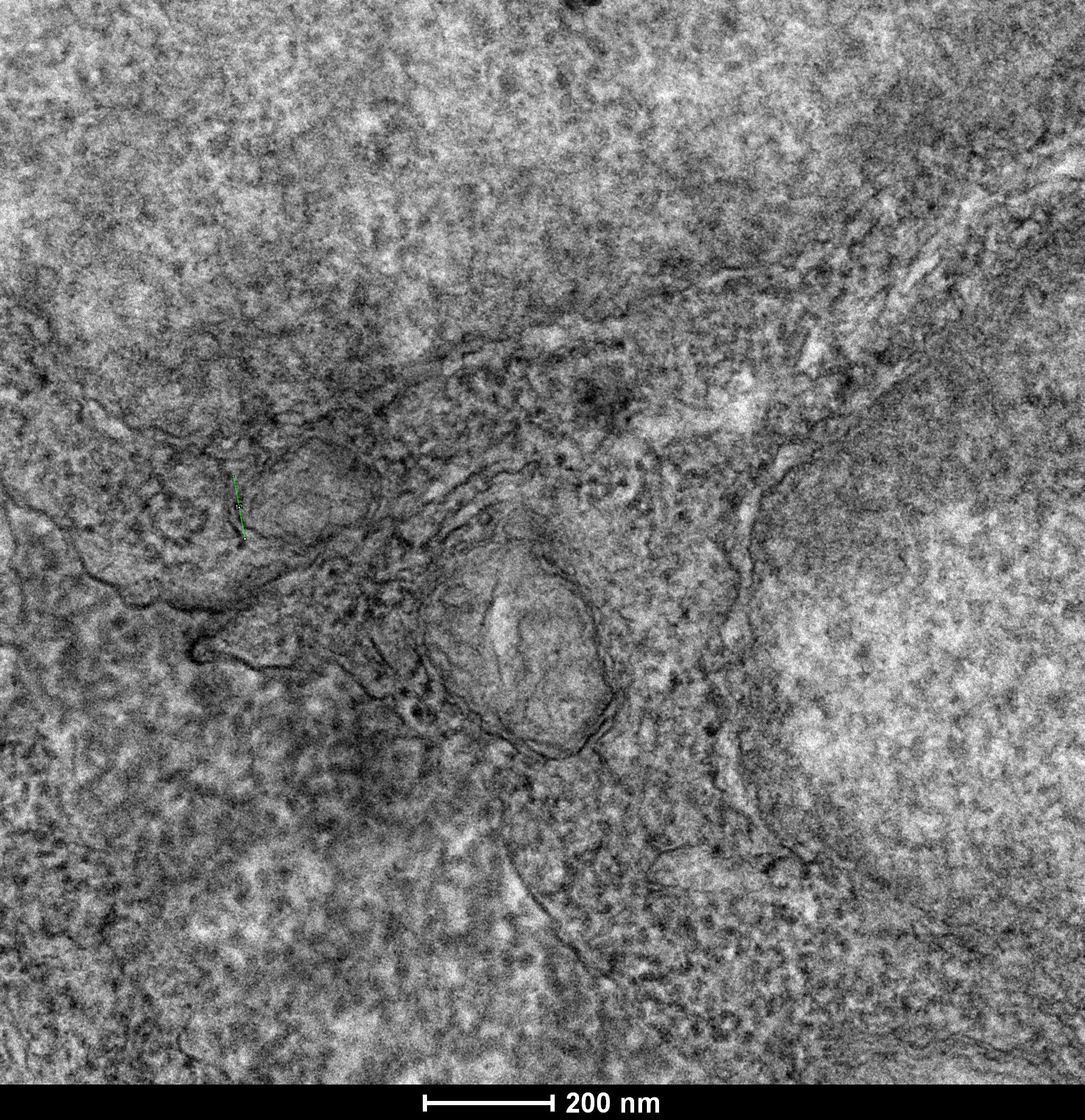

Supplement: S9 File — (ZIP) [file pone.0179859.s011.zip › Supplementary Images 4D/4b_L1_60000x_c8_m1.jpg]

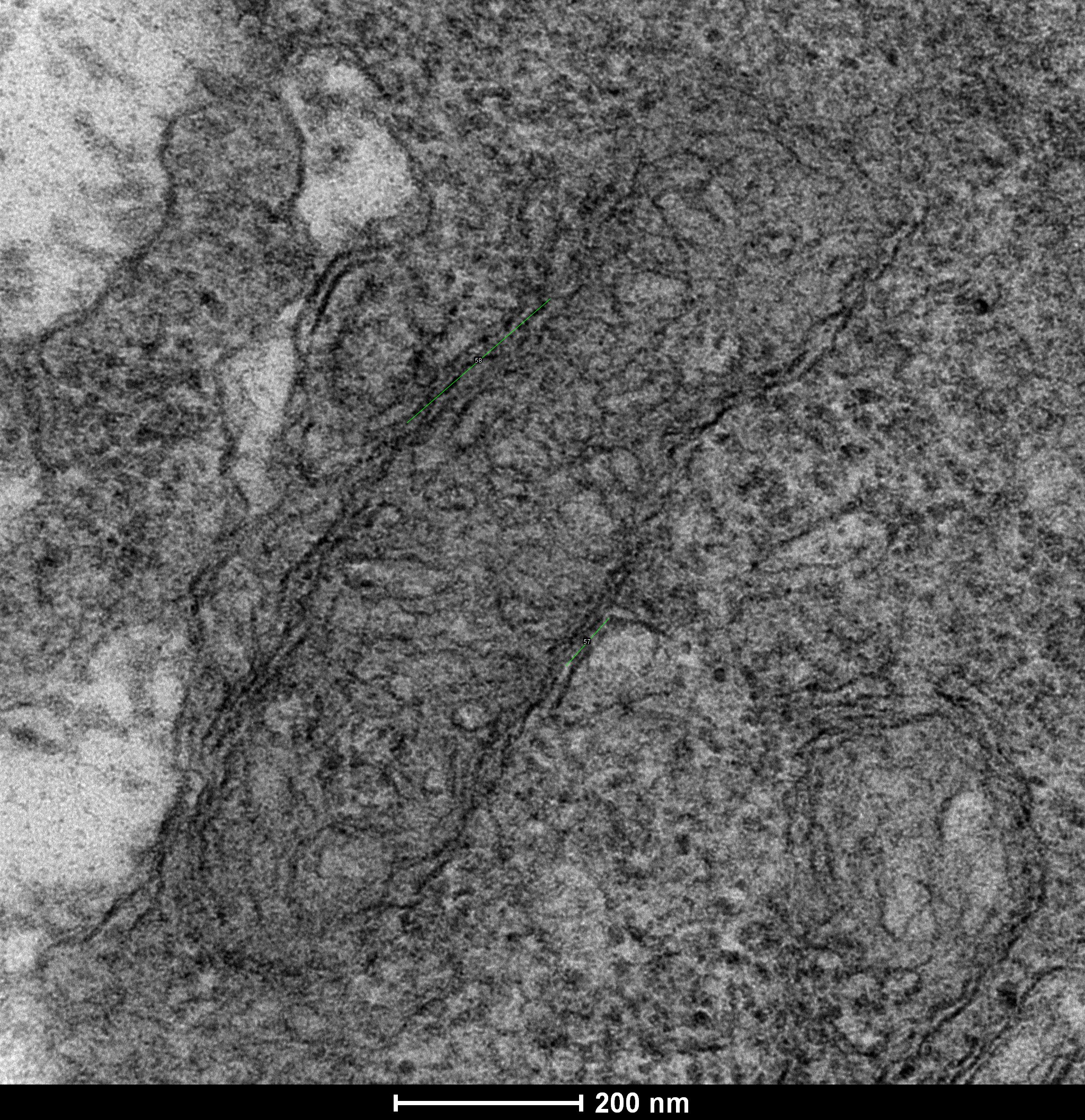

Supplement: S10 File — (ZIP) [file pone.0179859.s012.zip › Supplementary Images 4A/1b_L1_87000x_c1_M3.jpg]

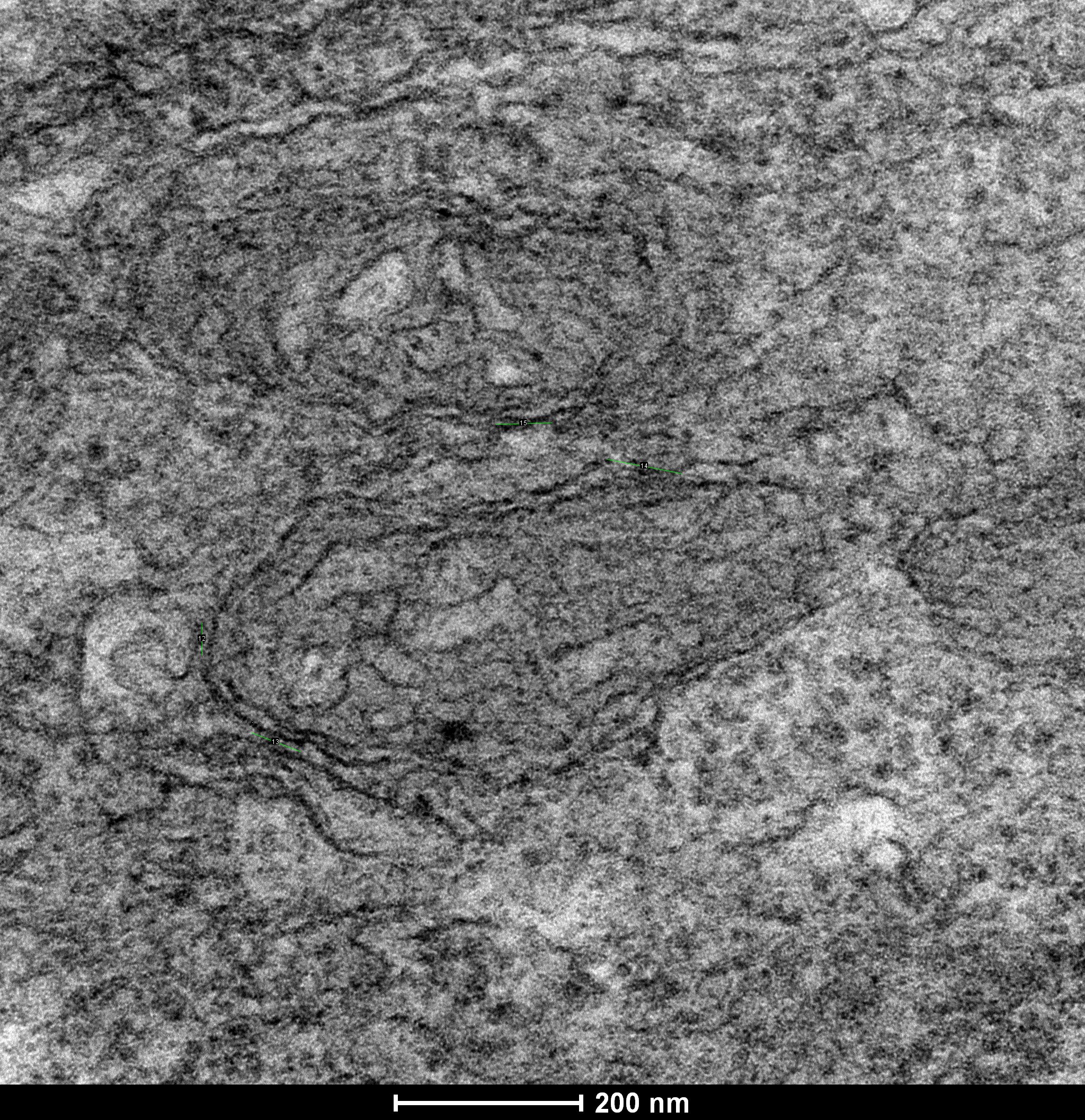

Supplement: S10 File — (ZIP) [file pone.0179859.s012.zip › Supplementary Images 4A/1b_L1_87000x_c7_M1_M2.jpg]

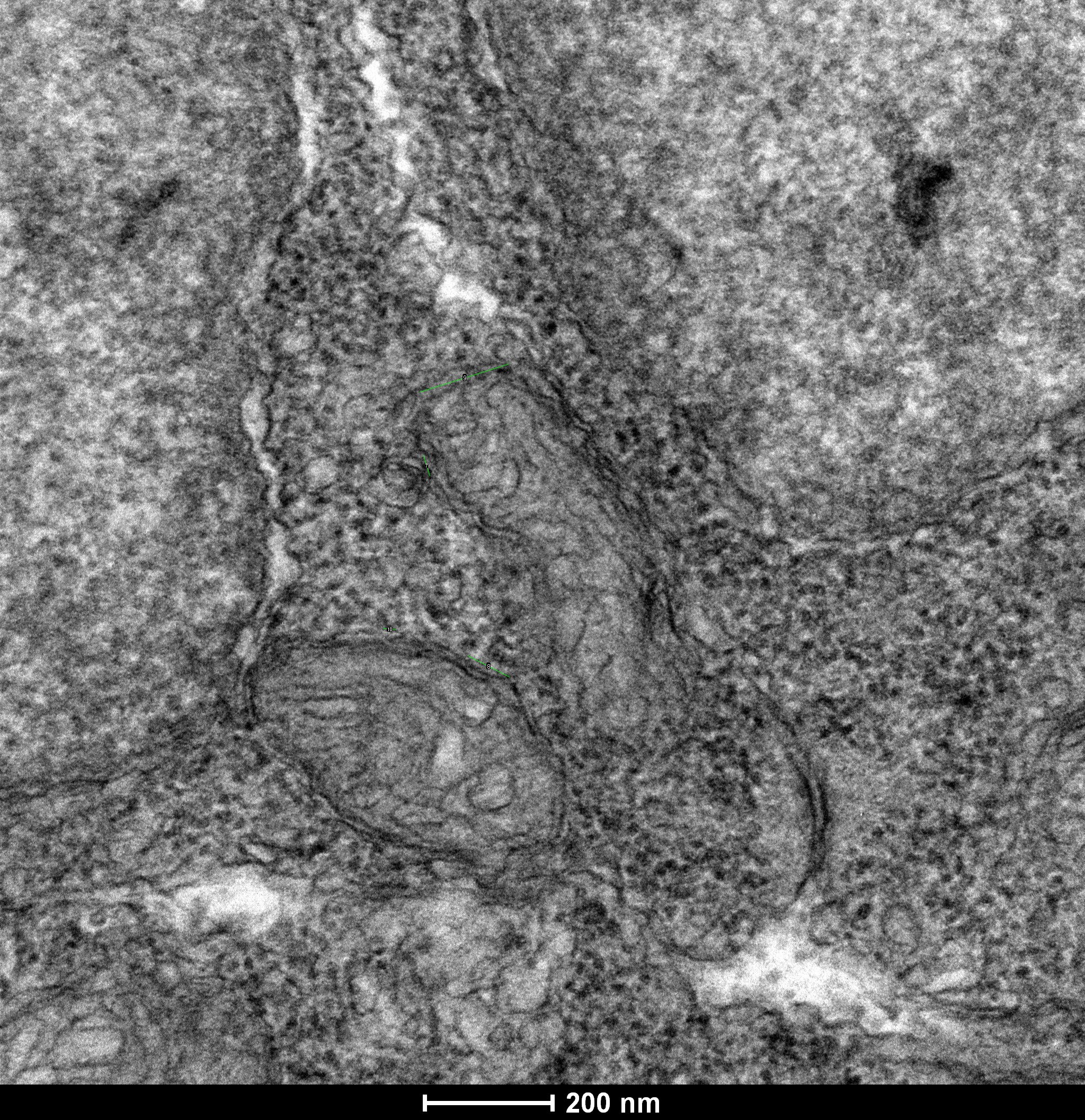

Supplement: S10 File — (ZIP) [file pone.0179859.s012.zip › Supplementary Images 4A/1b_L2_60000x_c2_m1_m2.jpg]

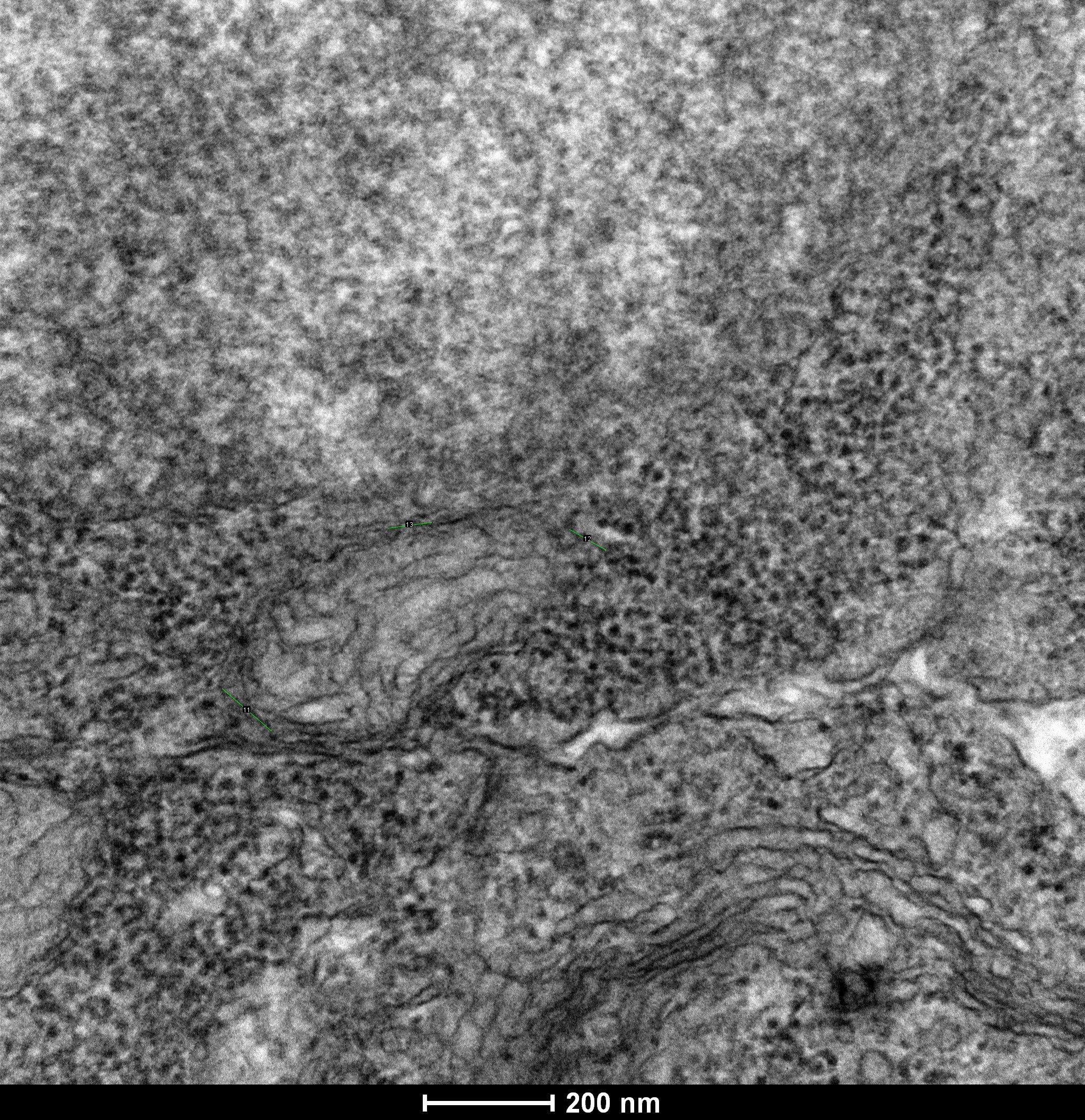

Supplement: S10 File — (ZIP) [file pone.0179859.s012.zip › Supplementary Images 4A/1b_L2_60000x_c3_m1.jpg]

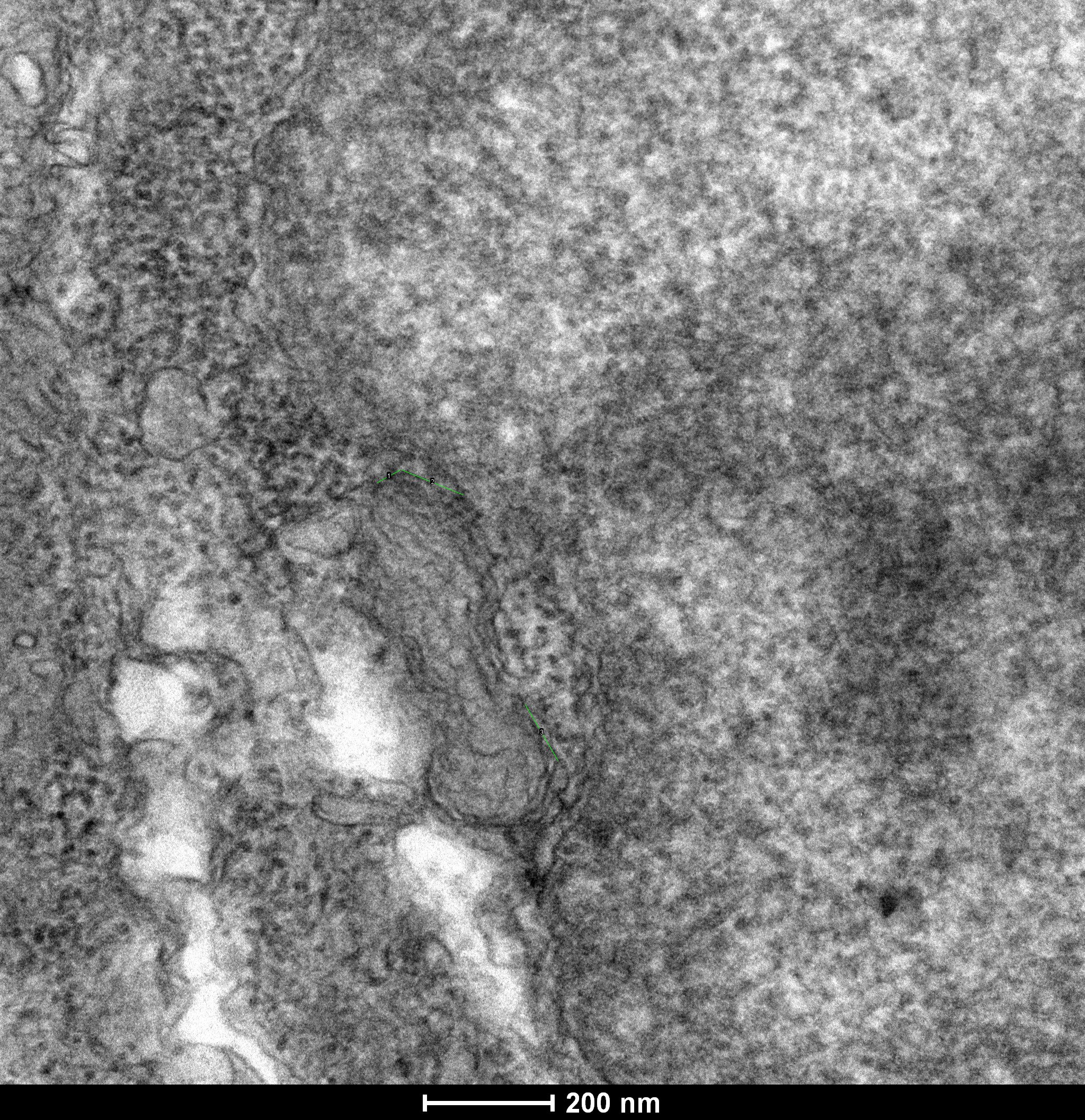

Supplement: S10 File — (ZIP) [file pone.0179859.s012.zip › Supplementary Images 4A/1b_L2_60000x_c4_m1.jpg]

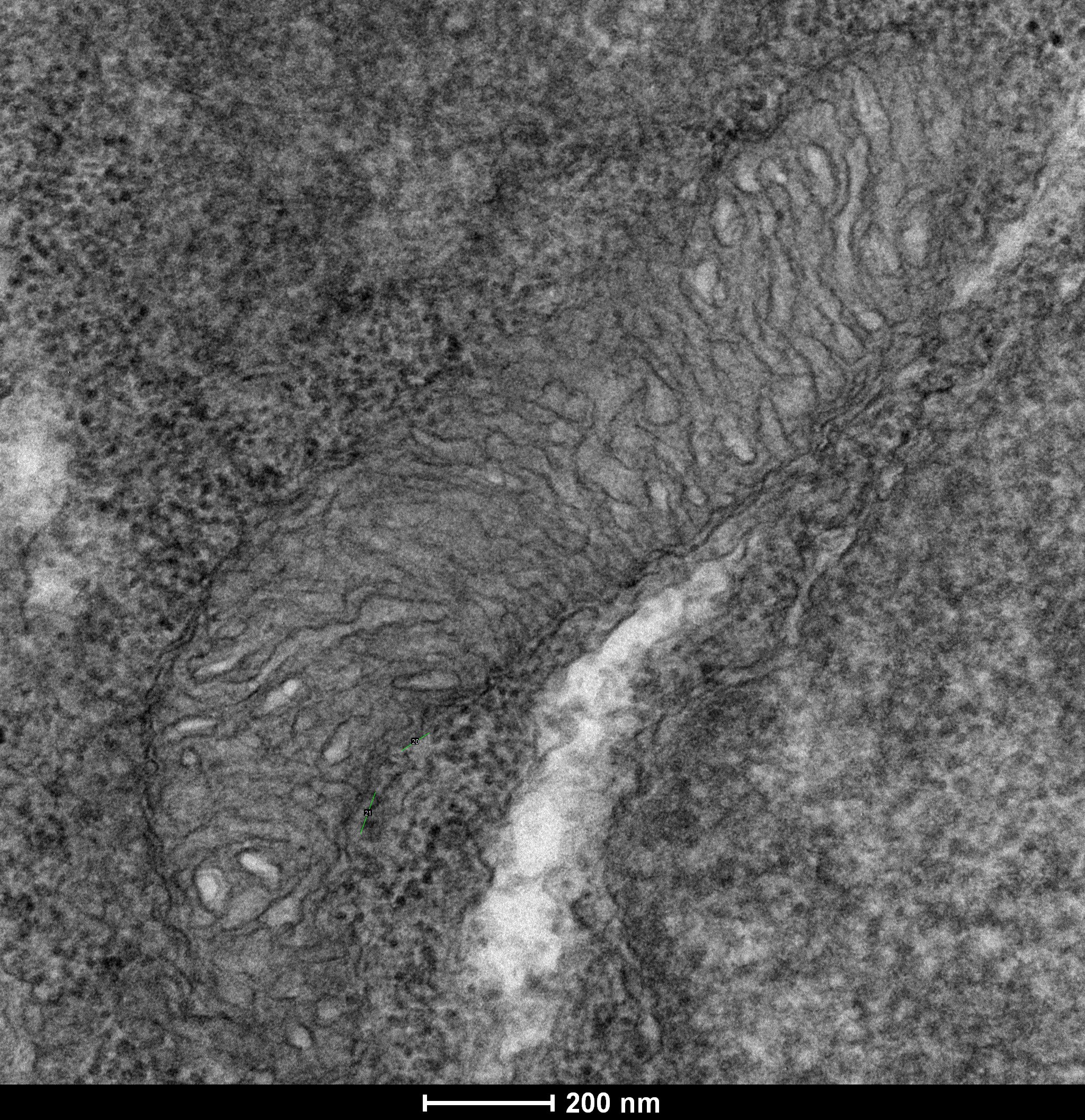

Supplement: S10 File — (ZIP) [file pone.0179859.s012.zip › Supplementary Images 4A/1b_L2_60000x_c5_m4.jpg]

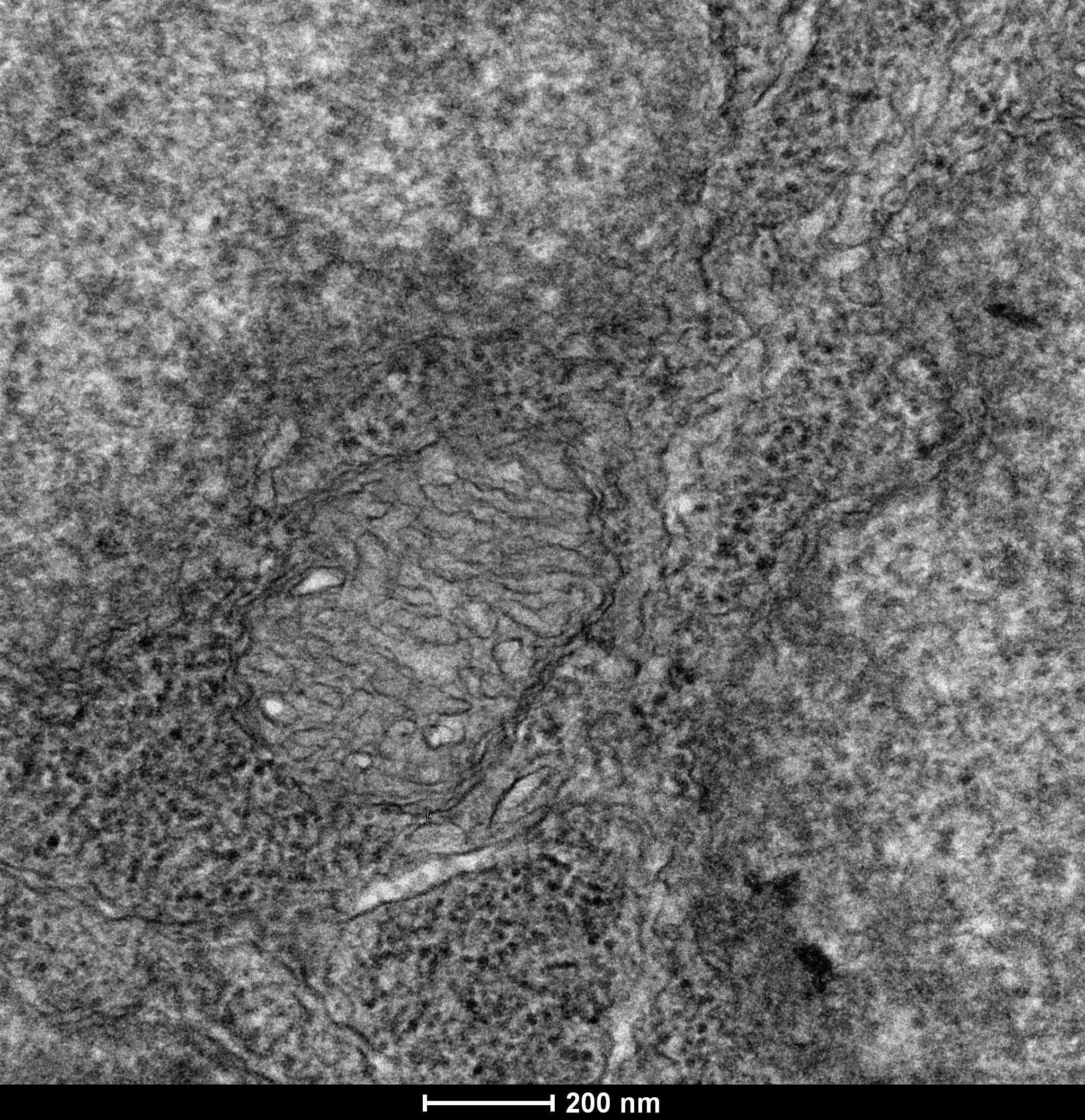

Supplement: S10 File — (ZIP) [file pone.0179859.s012.zip › Supplementary Images 4A/1b_L2_60000x_c7_m1.jpg]

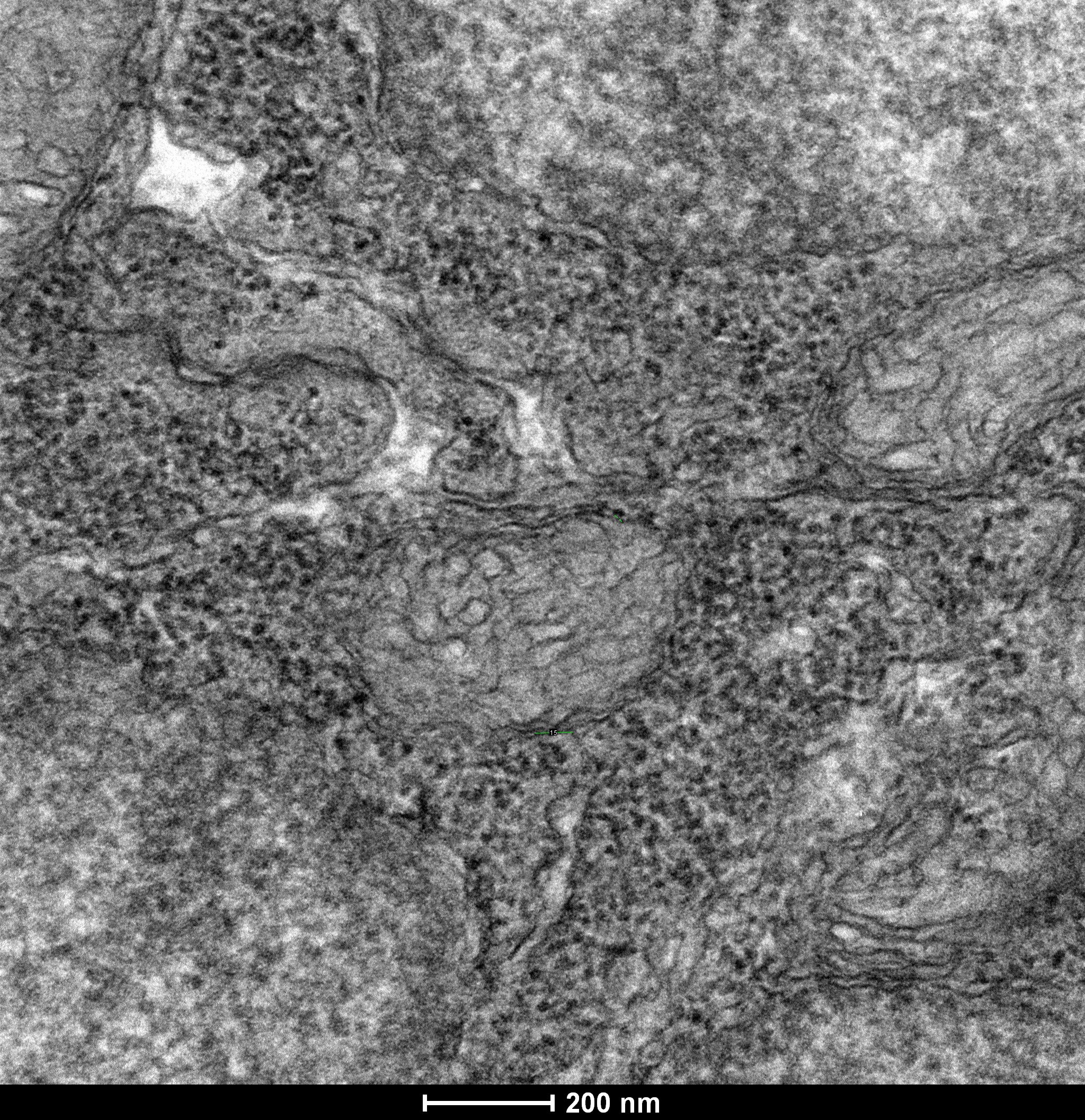

Supplement: S10 File — (ZIP) [file pone.0179859.s012.zip › Supplementary Images 4A/1b_L2_60000x_c7_m2.jpg]

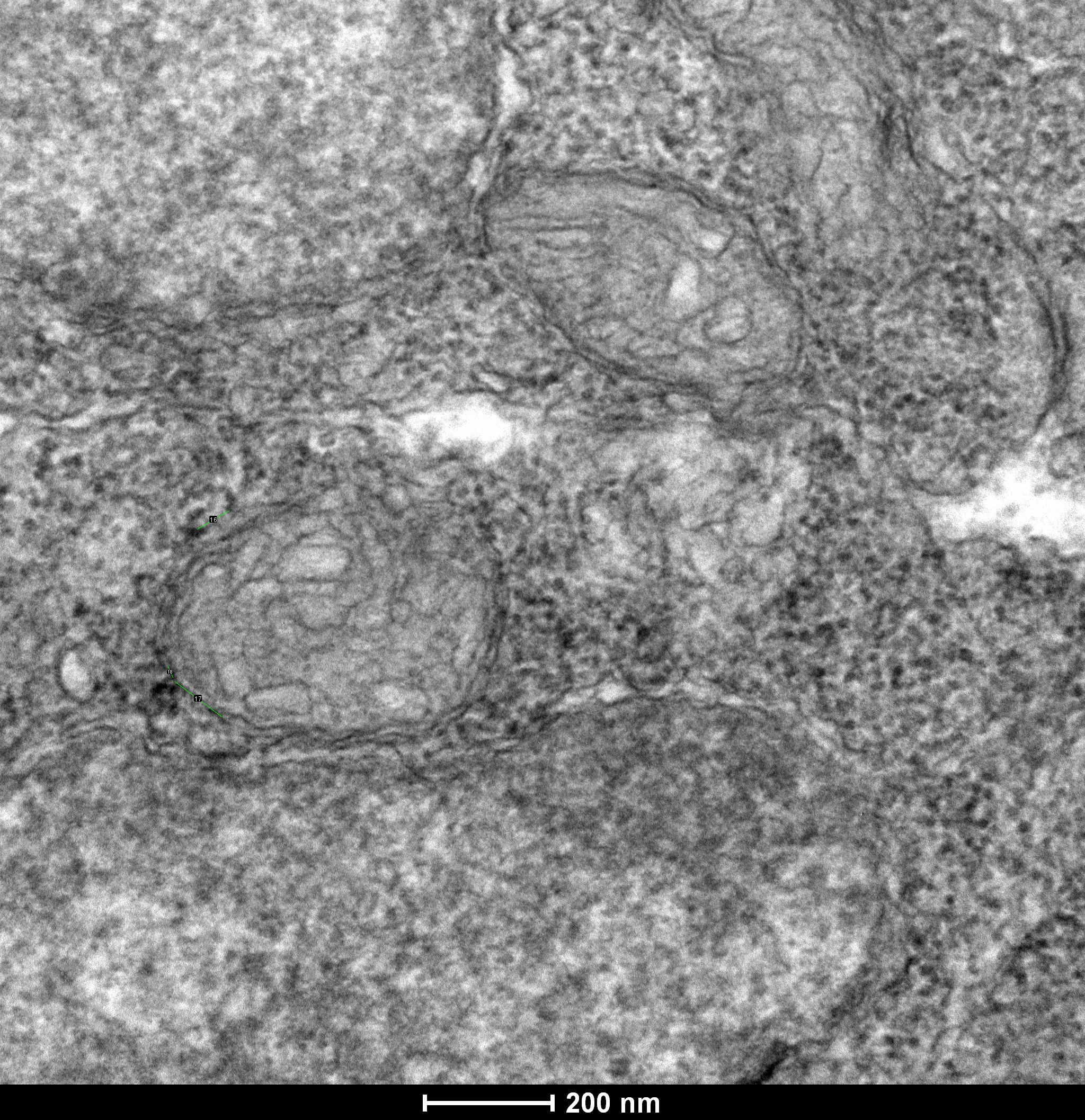

Supplement: S10 File — (ZIP) [file pone.0179859.s012.zip › Supplementary Images 4A/1b_L2_60000x_c8_m1.jpg]

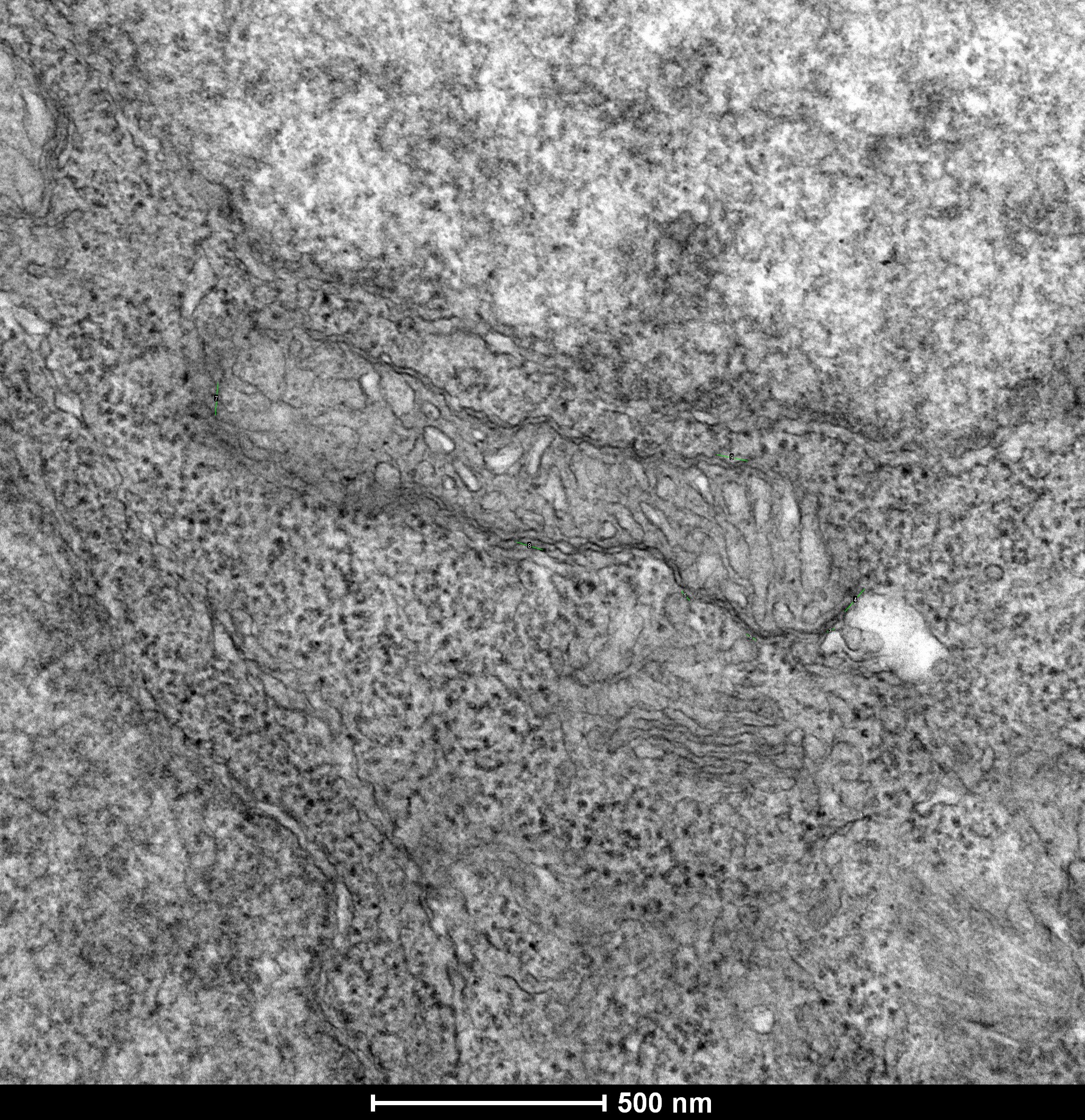

Supplement: S10 File — (ZIP) [file pone.0179859.s012.zip › Supplementary Images 4A/1e_L1_43000x_c1_m2.jpg]

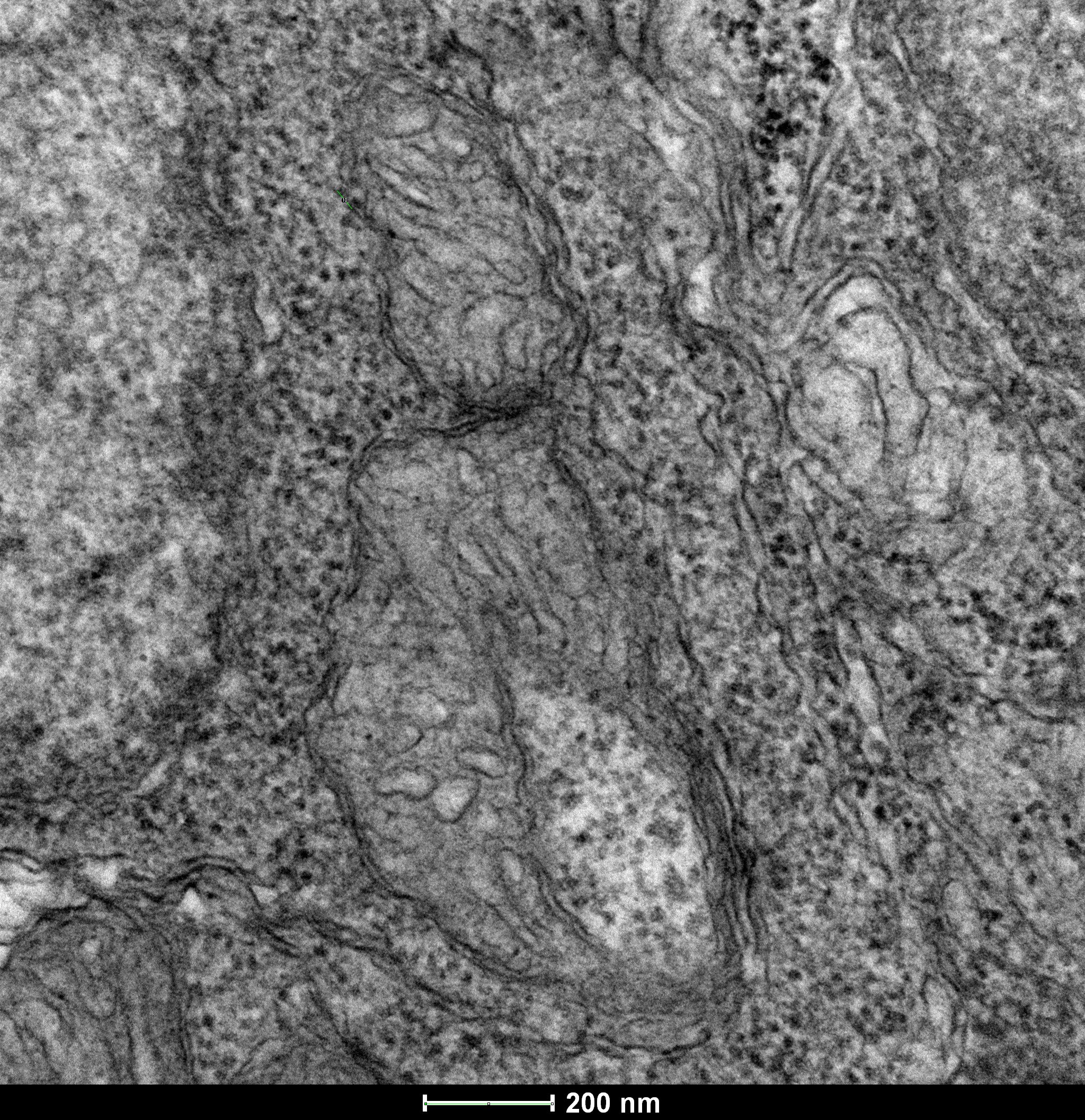

Supplement: S10 File — (ZIP) [file pone.0179859.s012.zip › Supplementary Images 4A/1e_L1_60000x_c1_m3_m4.jpg]

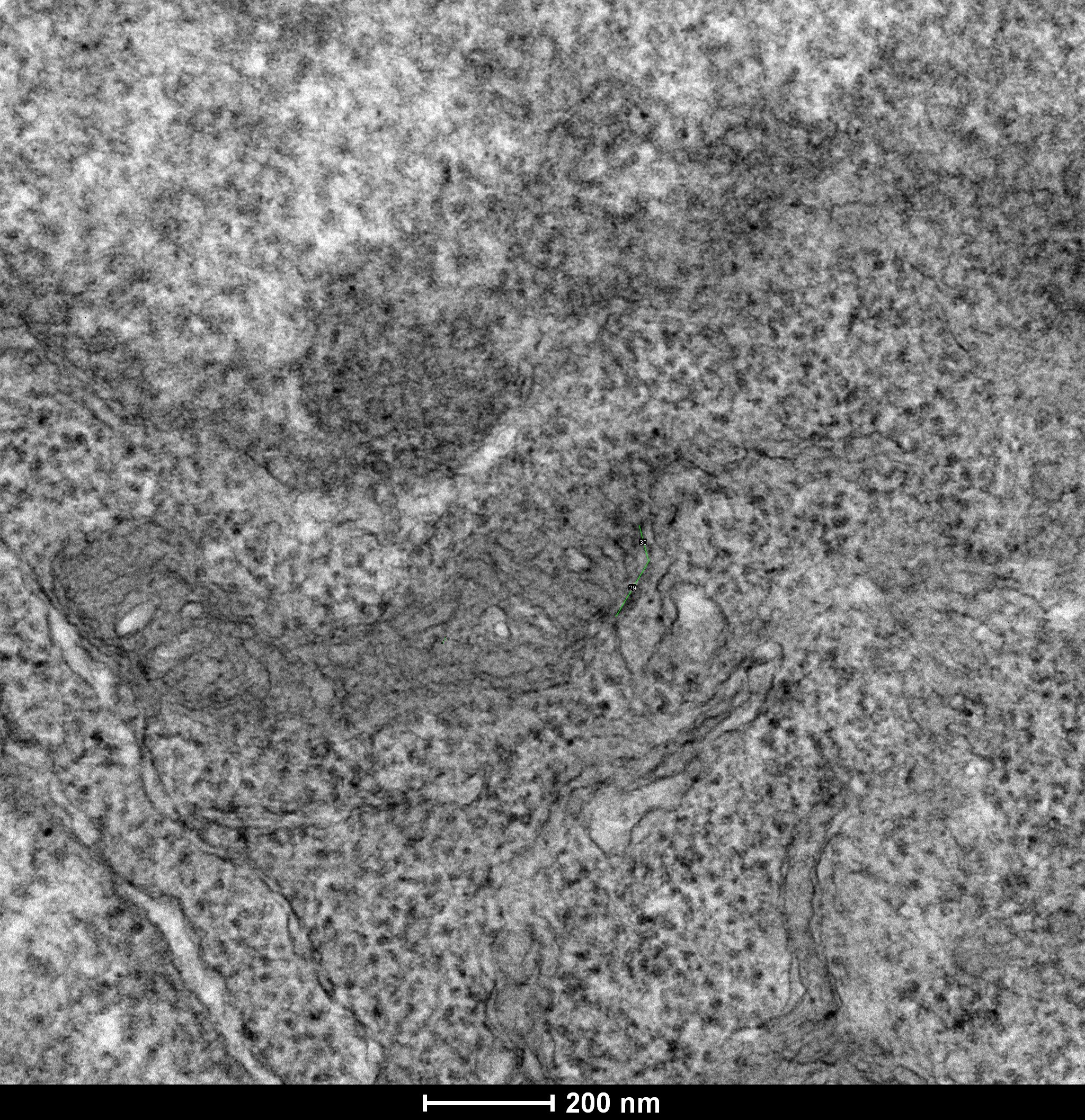

Supplement: S10 File — (ZIP) [file pone.0179859.s012.zip › Supplementary Images 4A/1e_L1_60000x_c4_m1.jpg]

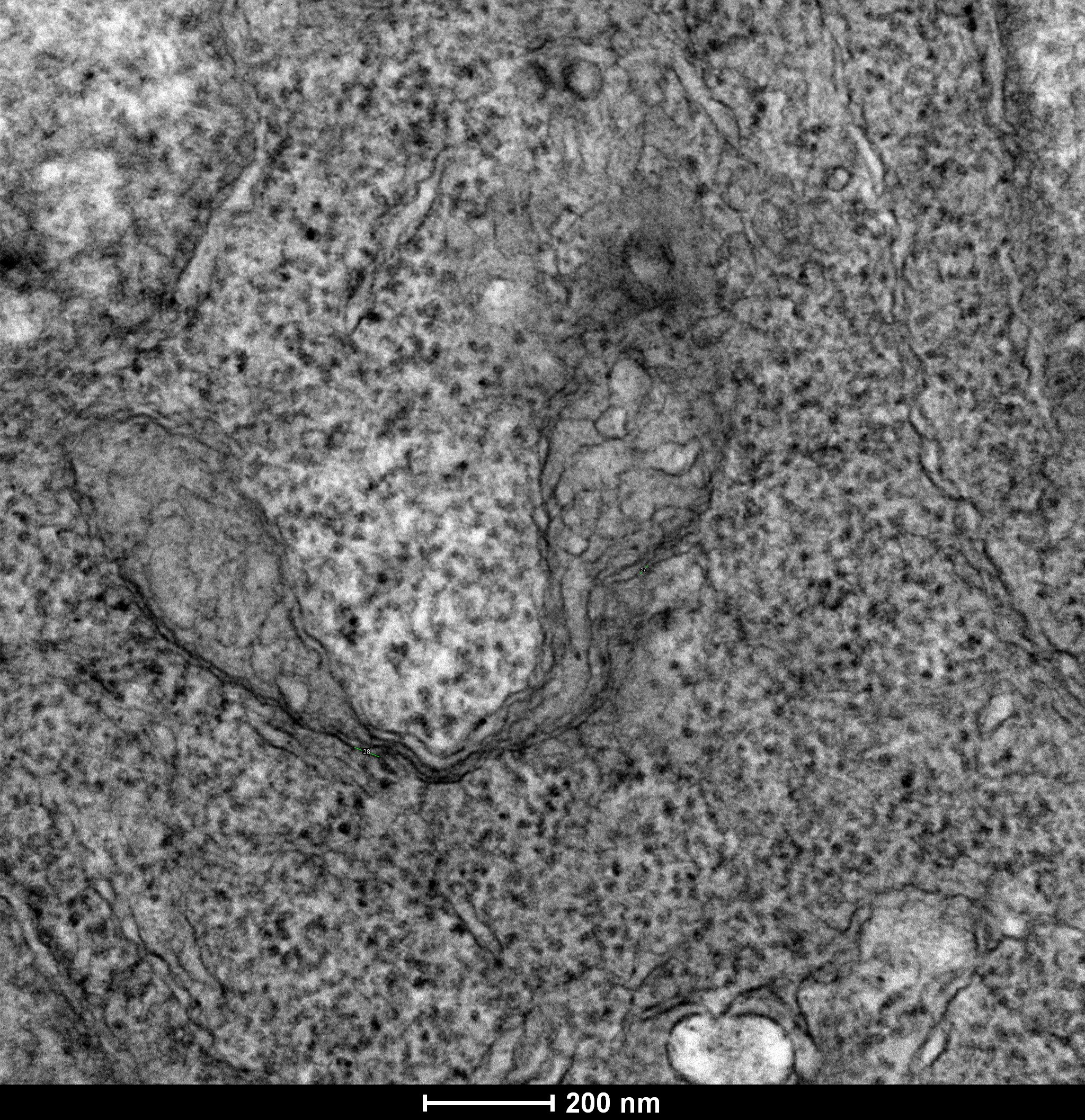

Supplement: S10 File — (ZIP) [file pone.0179859.s012.zip › Supplementary Images 4A/1e_L1_60000x_c5_m1.jpg]

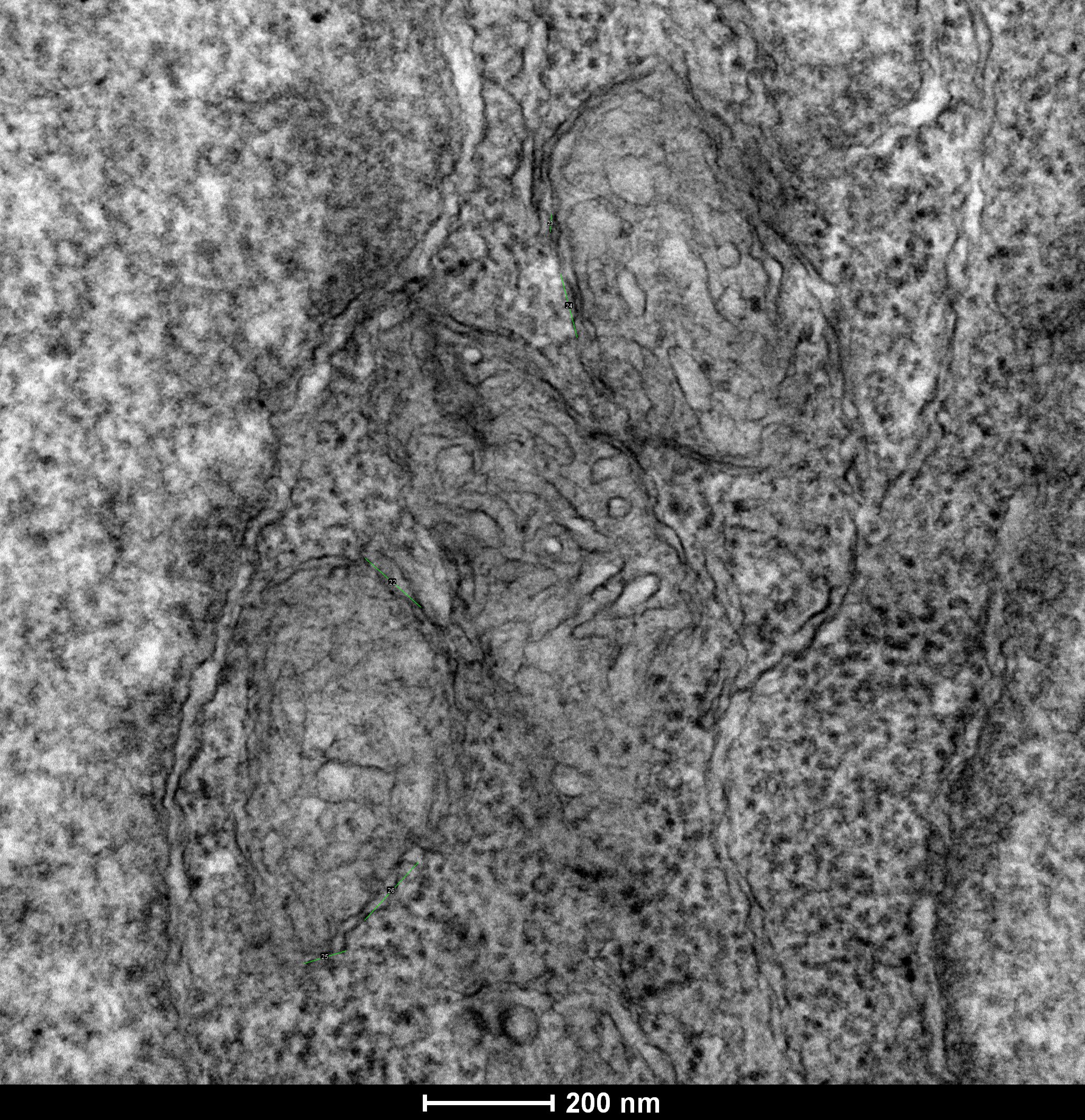

Supplement: S10 File — (ZIP) [file pone.0179859.s012.zip › Supplementary Images 4A/1e_L1_60000x_c5_m2_m3_m4.jpg]
