# Supplementary figures and images for: Mitochondrion to endoplasmic reticulum apposition length in zebrafish embryo spinal progenitors is unchanged in response to perturbations associated with Alzheimer’s disease (part 2 of 2)
Source: PLoS One. 2017 Jun 21;12(6):e0179859. doi: 10.1371/journal.pone.0179859 (PMC5479591; doi:10.1371/journal.pone.0179859)

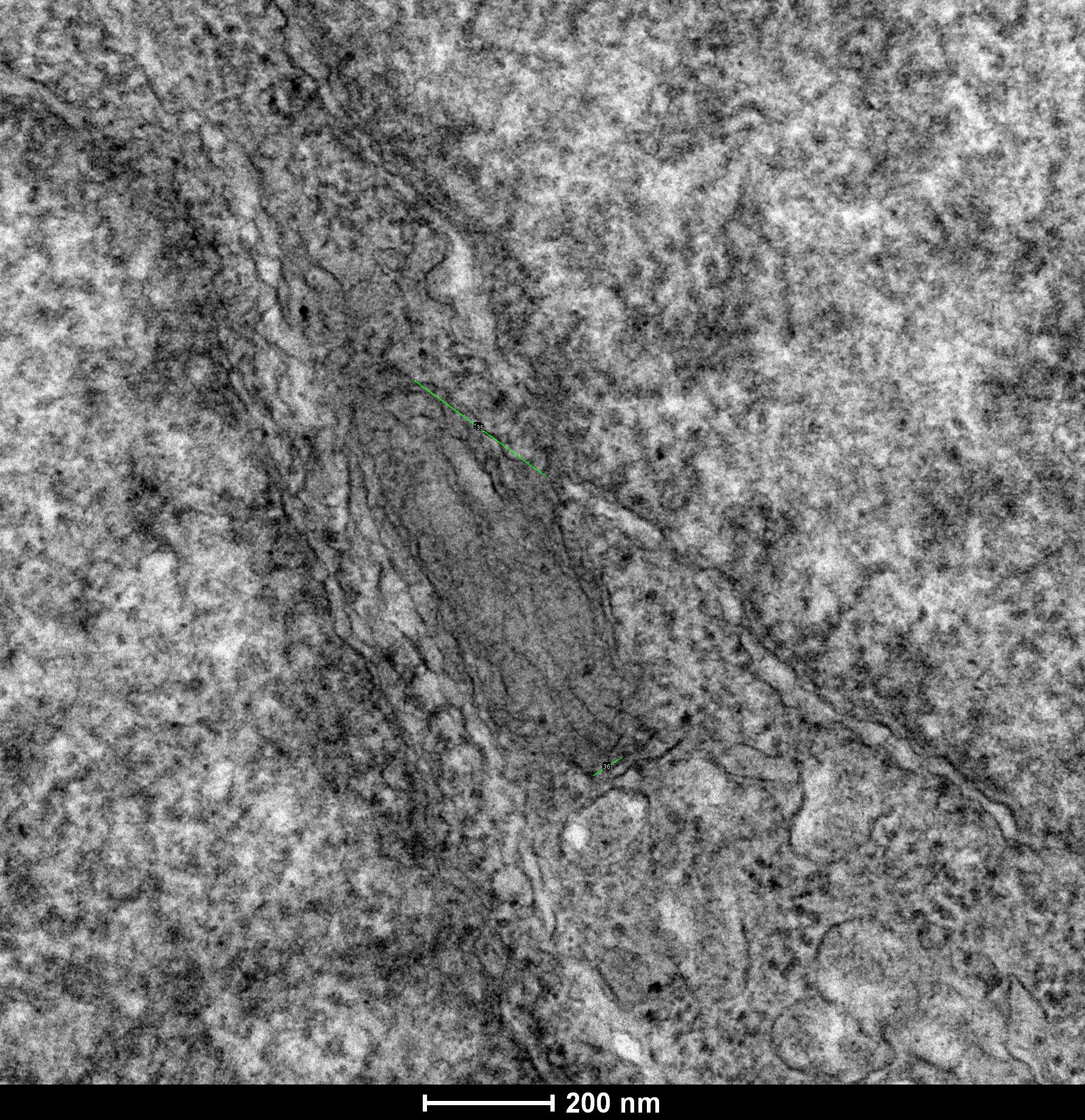

Supplement: S10 File — (ZIP) [file pone.0179859.s012.zip › Supplementary Images 4A/1e_L1_60000x_c6_m1.jpg]

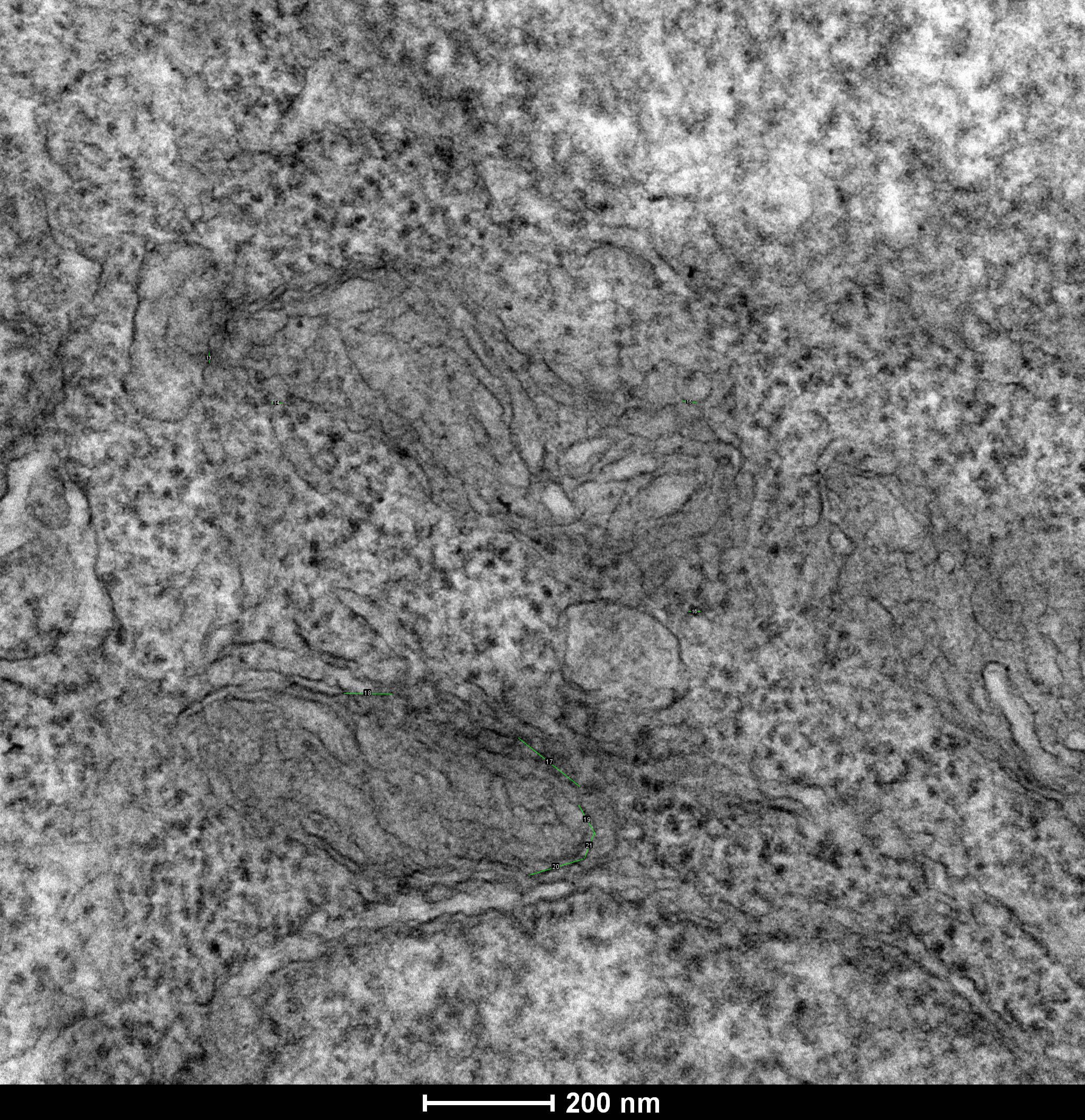

Supplement: S10 File — (ZIP) [file pone.0179859.s012.zip › Supplementary Images 4A/1e_L1_60000x_c7_m1_m2.jpg]

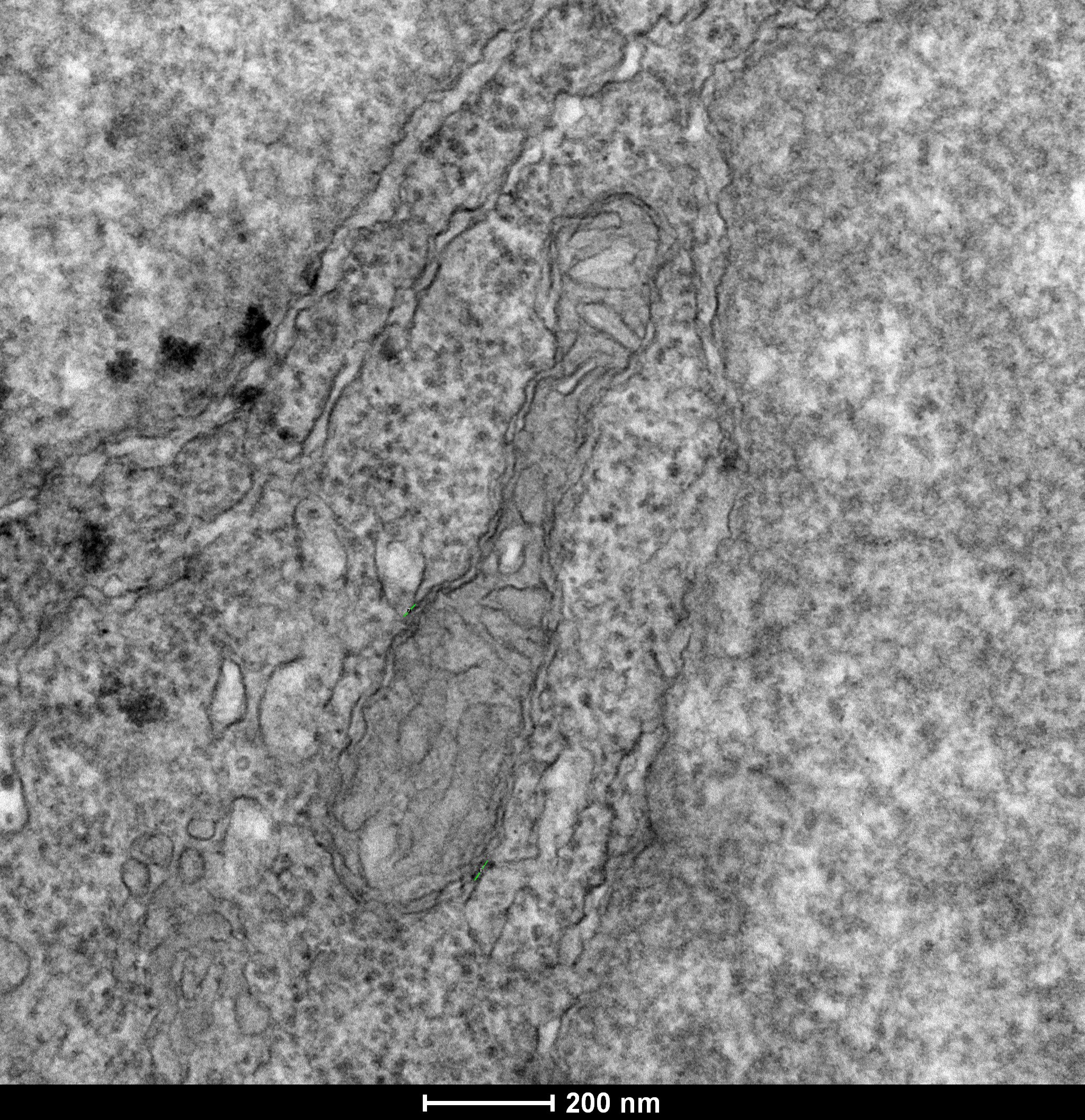

Supplement: S10 File — (ZIP) [file pone.0179859.s012.zip › Supplementary Images 4A/1d_L1_60000x_c4_m1.jpg]

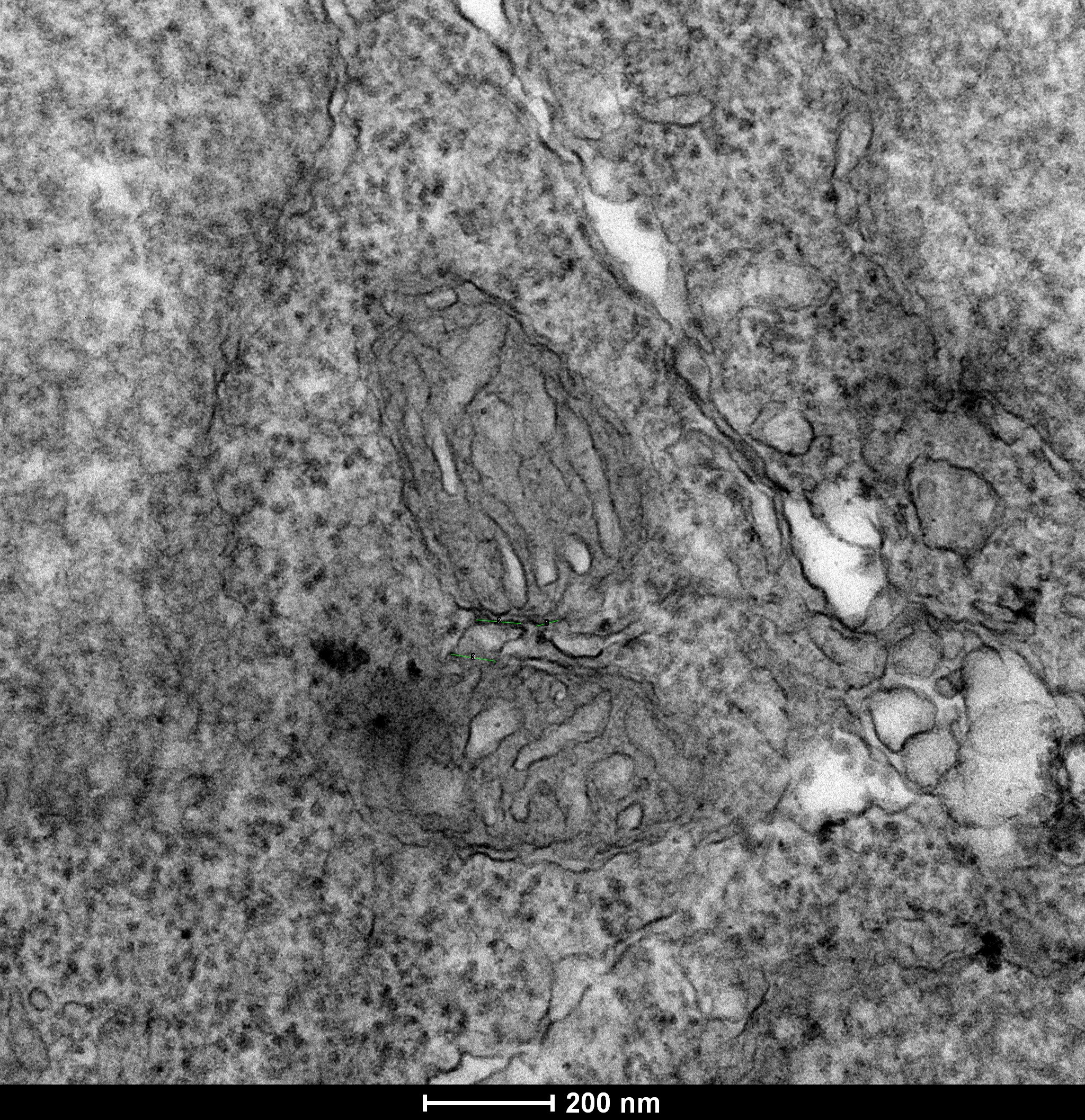

Supplement: S10 File — (ZIP) [file pone.0179859.s012.zip › Supplementary Images 4A/1d_L1_60000x_c5_m1_m2.jpg]

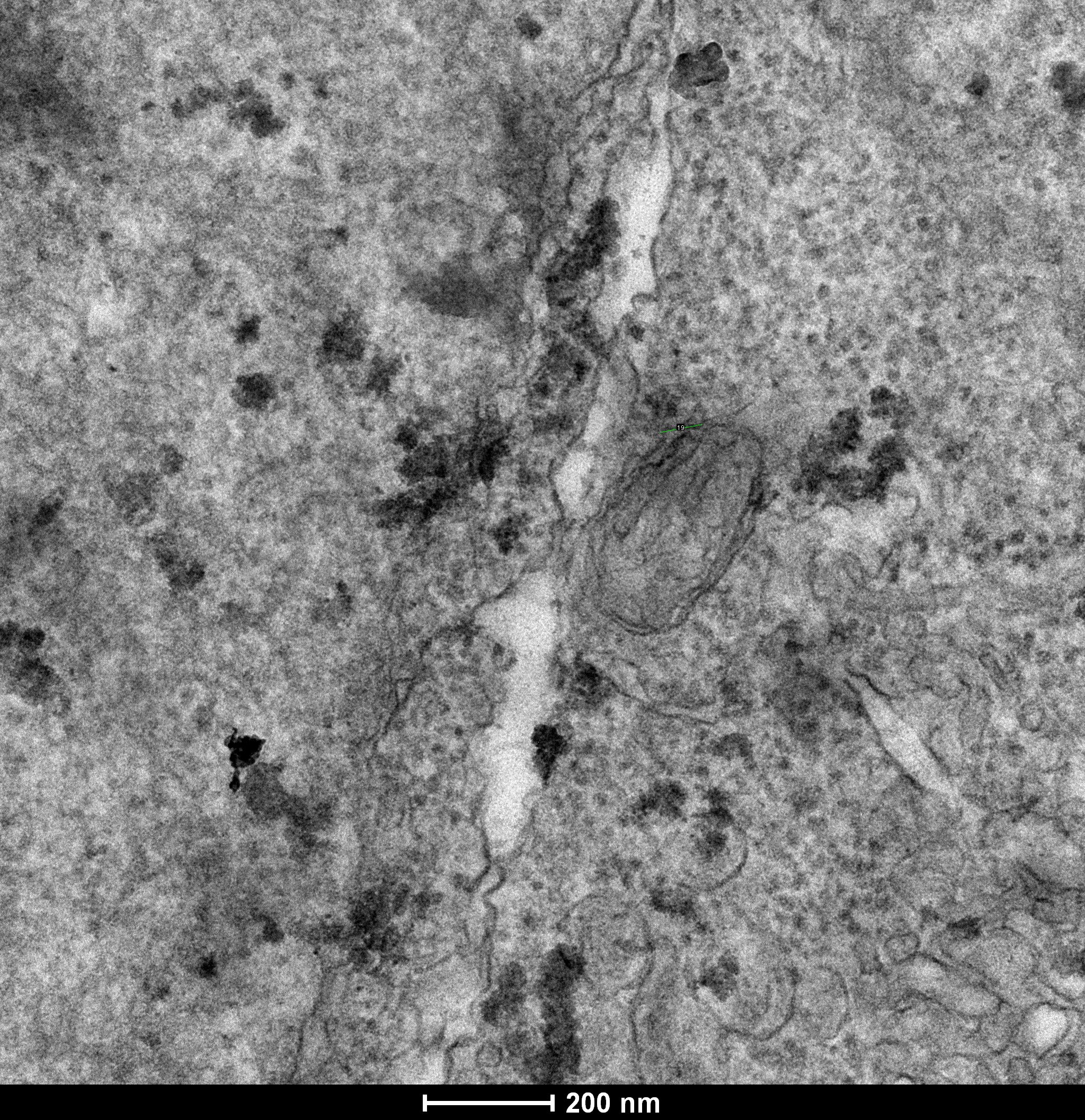

Supplement: S10 File — (ZIP) [file pone.0179859.s012.zip › Supplementary Images 4A/1d_L1_60000x_c5_m3.jpg]

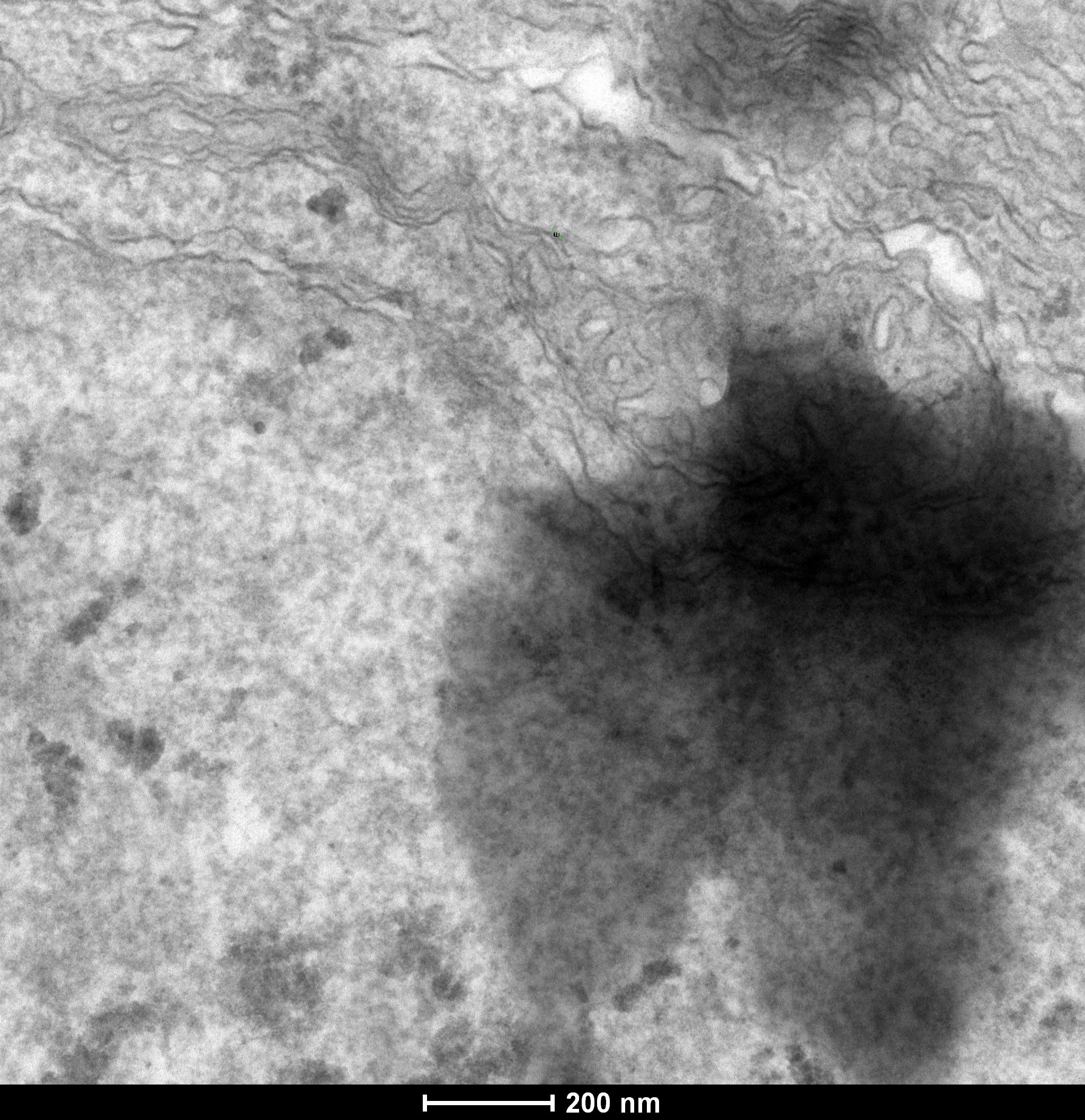

Supplement: S10 File — (ZIP) [file pone.0179859.s012.zip › Supplementary Images 4A/1d_L1_60000x_c6_m2.jpg]

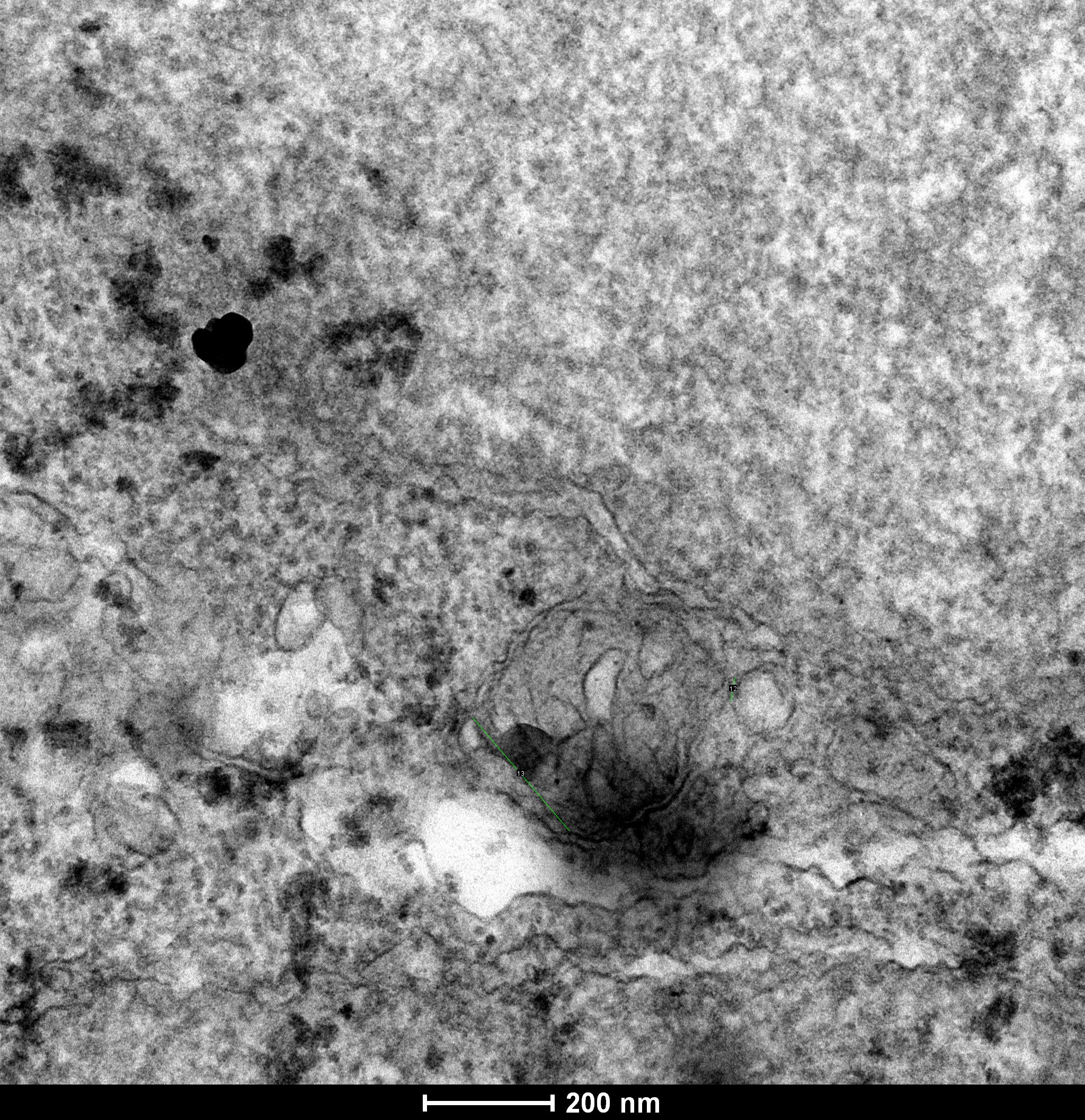

Supplement: S10 File — (ZIP) [file pone.0179859.s012.zip › Supplementary Images 4A/1d_L1_60000x_c8_m1.jpg]

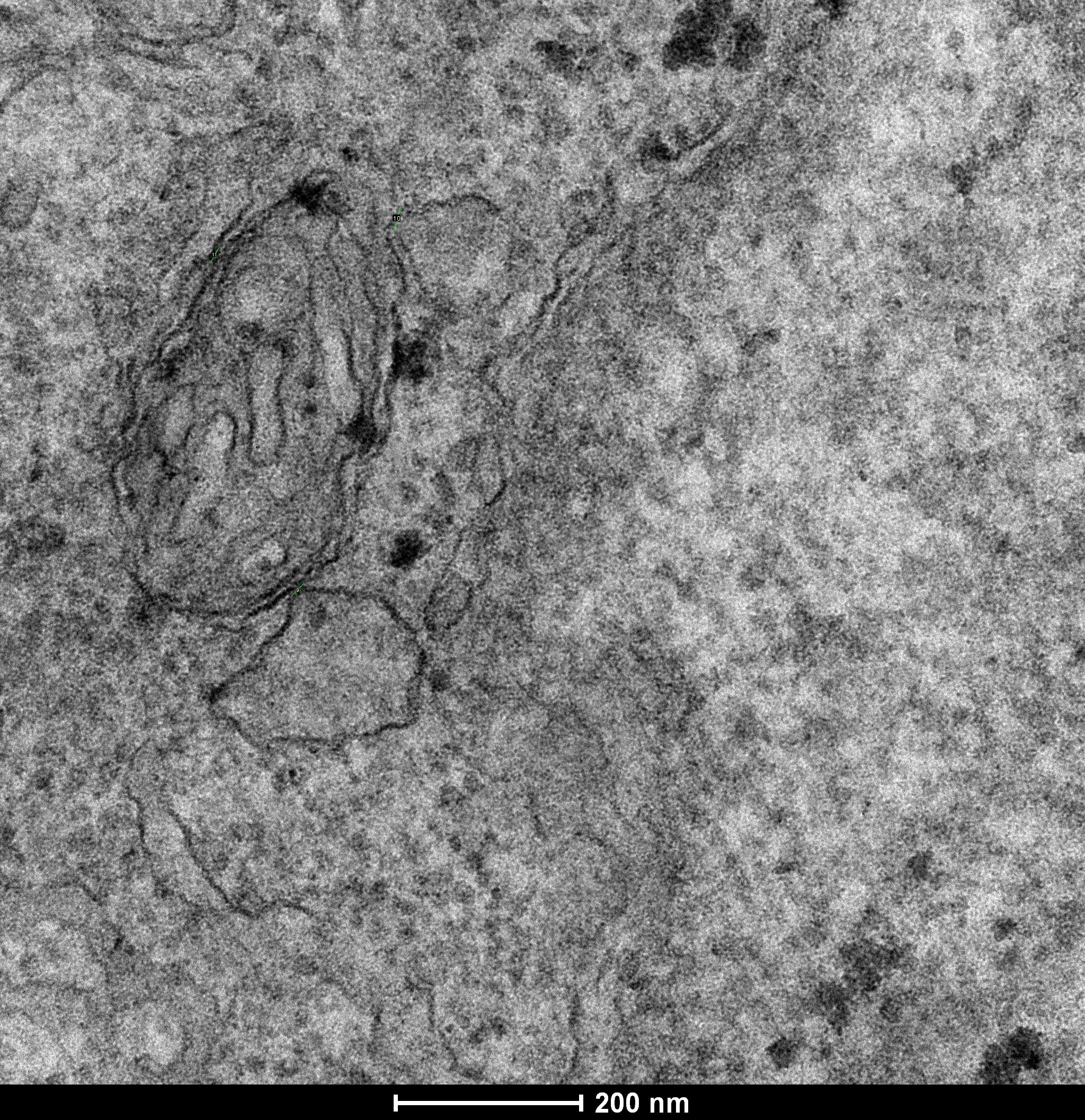

Supplement: S10 File — (ZIP) [file pone.0179859.s012.zip › Supplementary Images 4A/1d_L1_87000x_c2_m1.jpg]

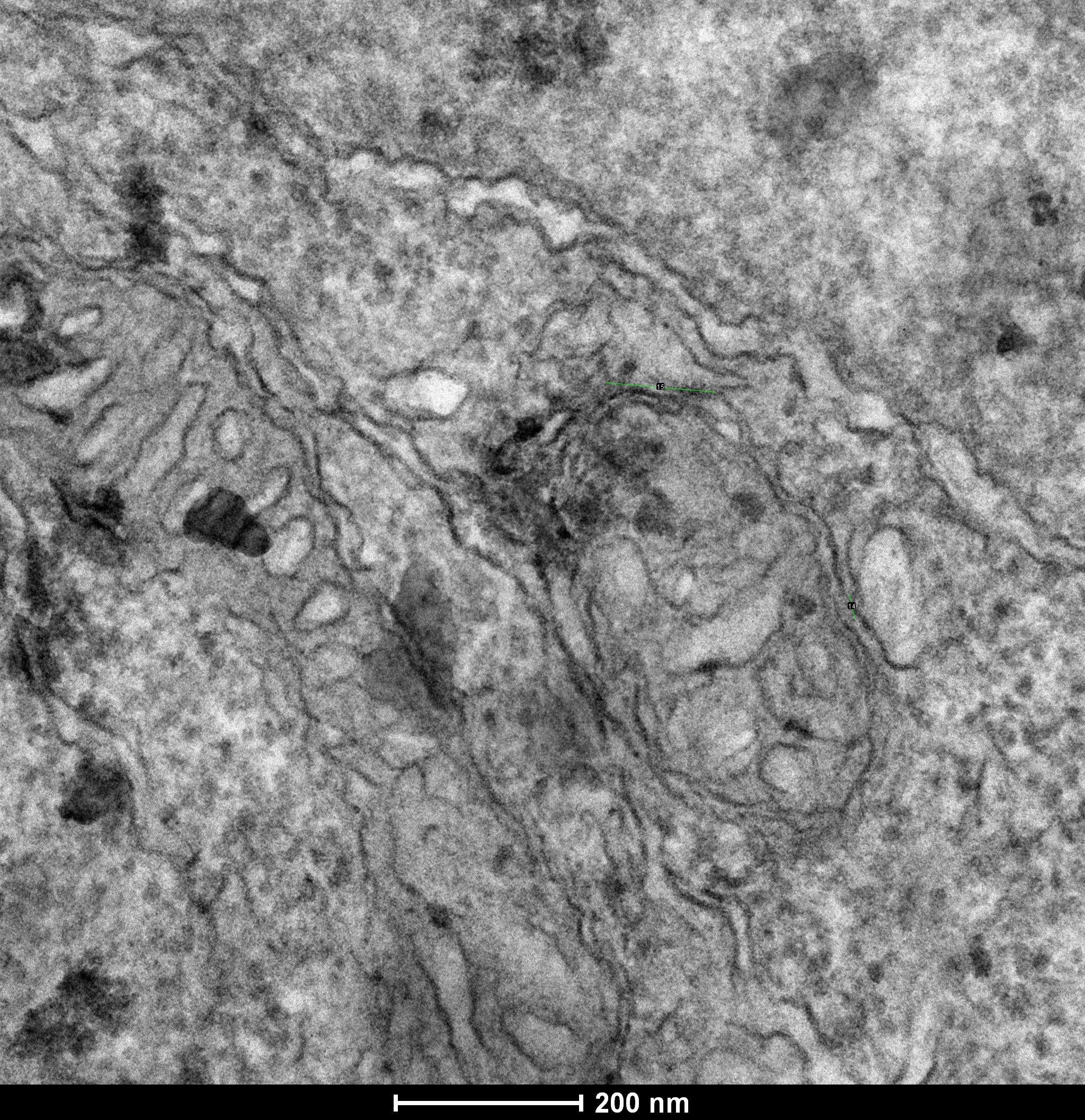

Supplement: S10 File — (ZIP) [file pone.0179859.s012.zip › Supplementary Images 4A/1d_L1_87000x_c4_m2.jpg]

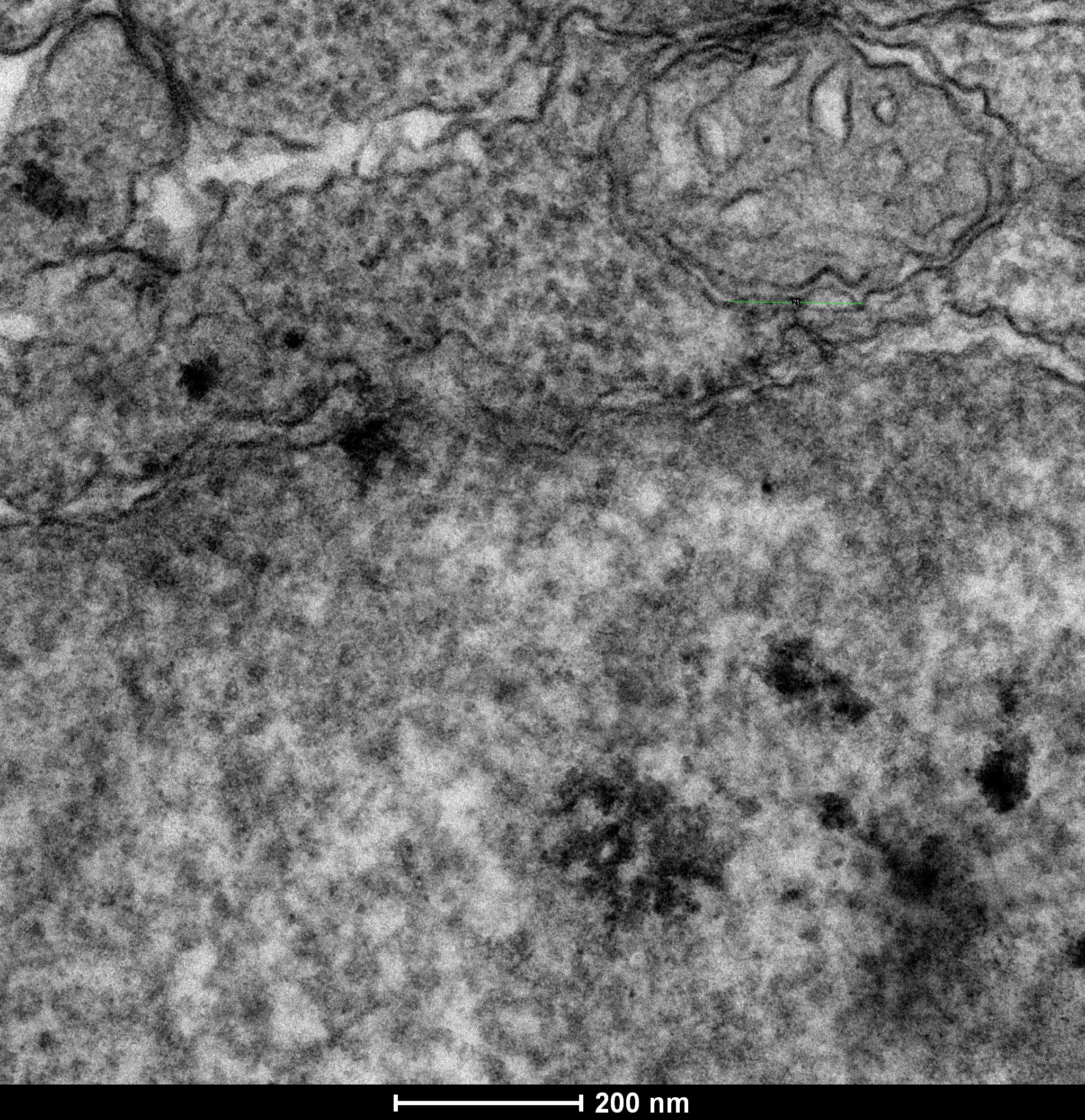

Supplement: S10 File — (ZIP) [file pone.0179859.s012.zip › Supplementary Images 4A/1d_L1_87000x_c6_m1.jpg]

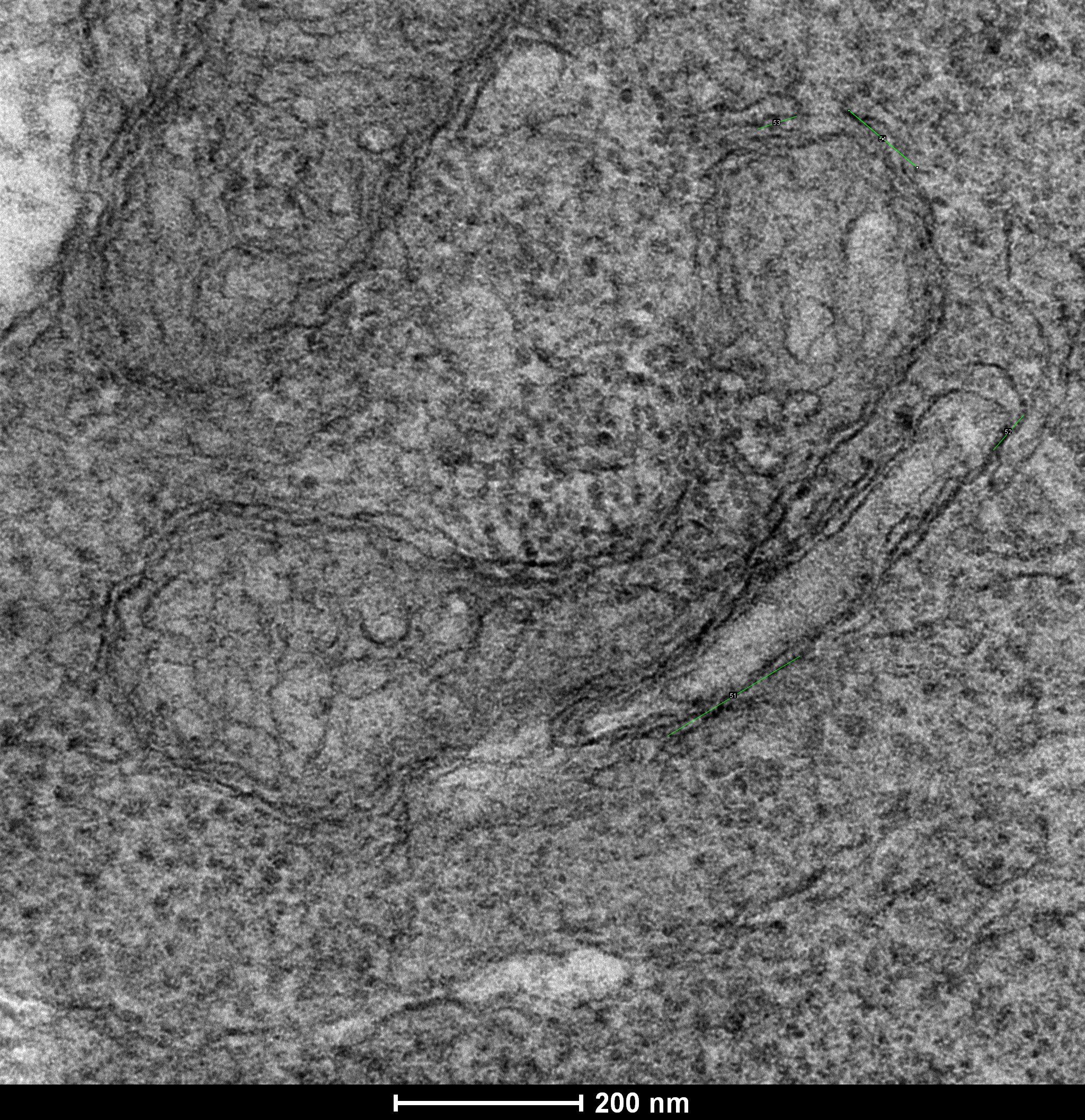

Supplement: S10 File — (ZIP) [file pone.0179859.s012.zip › Supplementary Images 4A/1b_L1_60000x_c1_M1_M2.jpg]

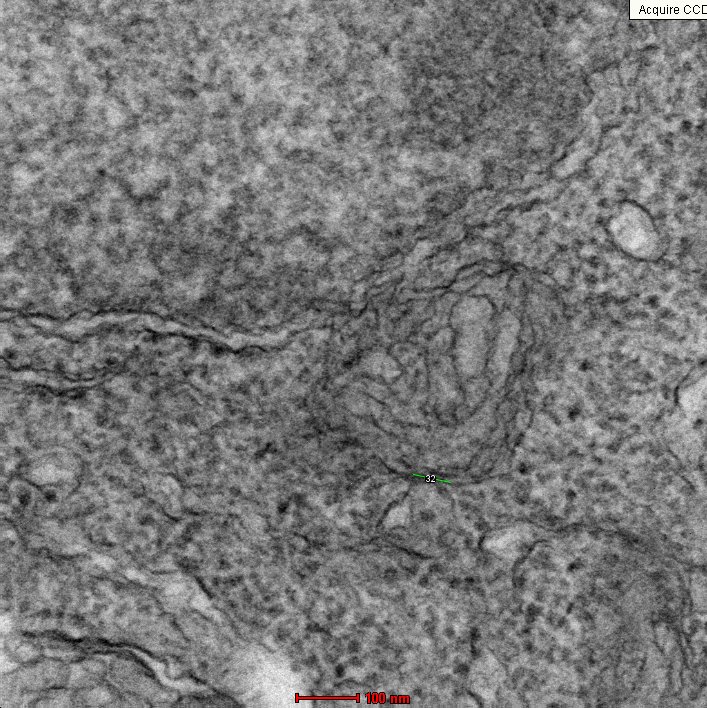

Supplement: S11 File — (ZIP) [file pone.0179859.s013.zip › Supplementary Images 4E/5a_L1_8700x_c3_m1 .jpg]

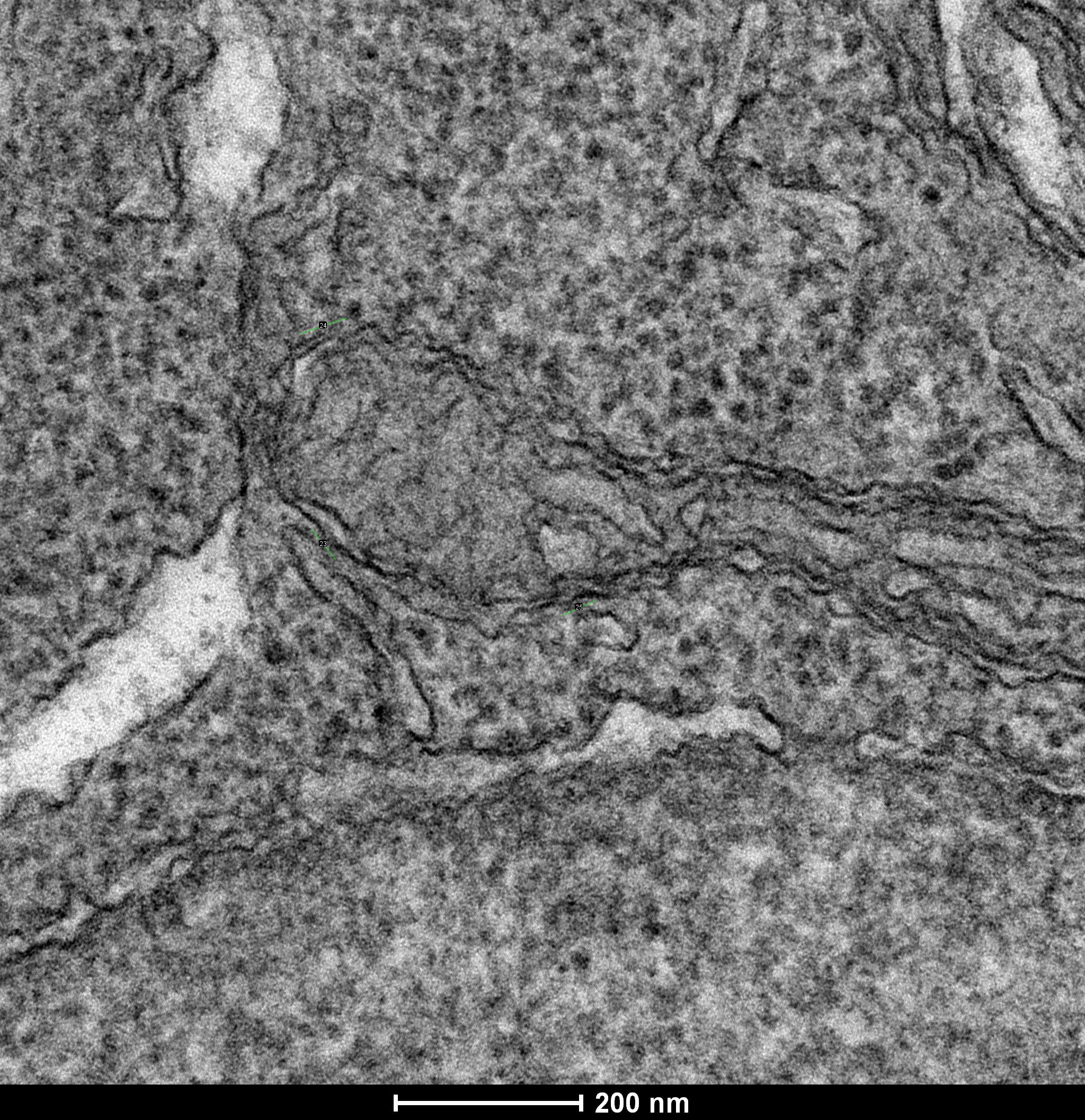

Supplement: S11 File — (ZIP) [file pone.0179859.s013.zip › Supplementary Images 4E/5a_L1_87000x_c1_m1.jpg]

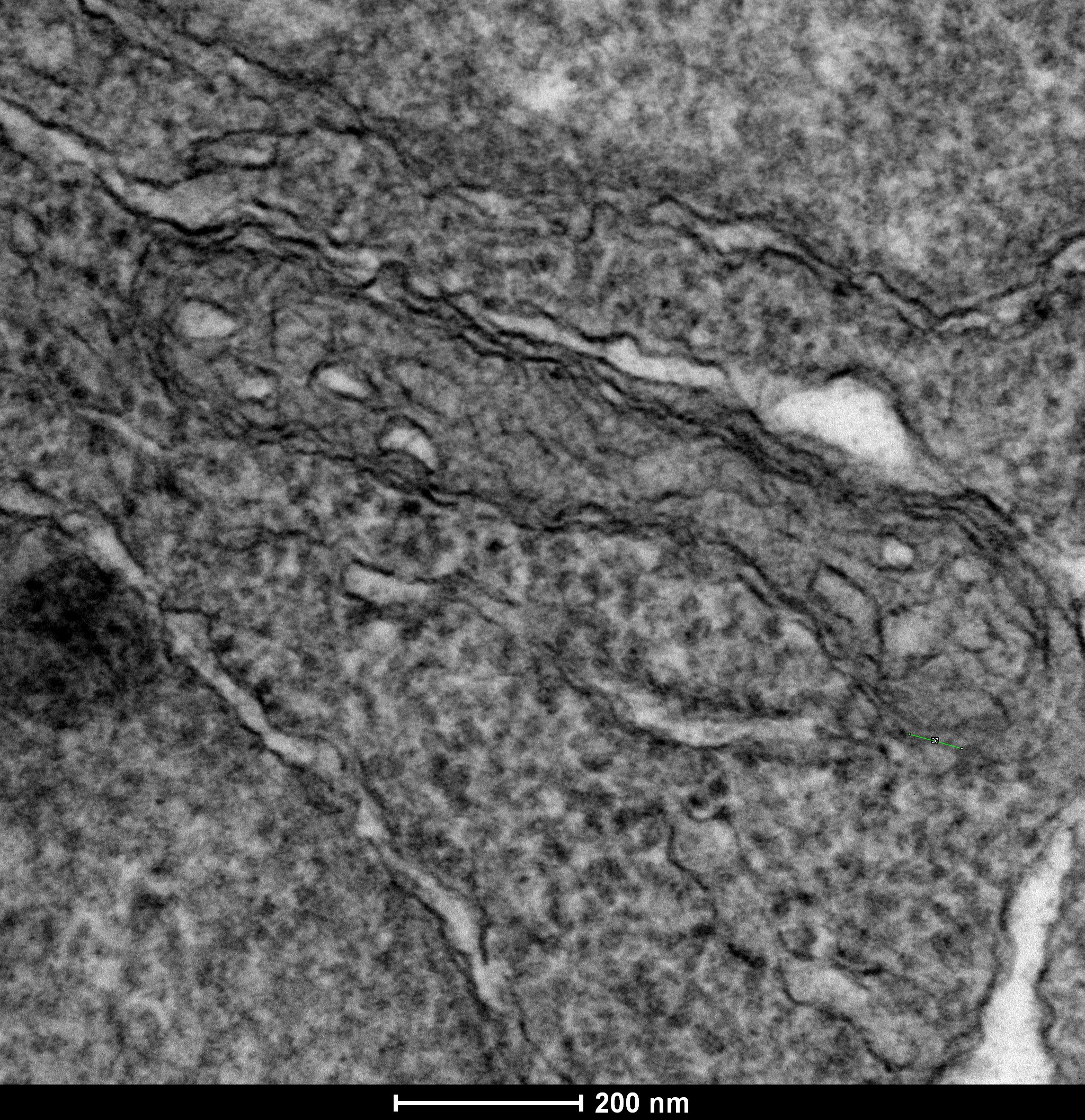

Supplement: S11 File — (ZIP) [file pone.0179859.s013.zip › Supplementary Images 4E/5a_L1_87000x_c1_m2.jpg]

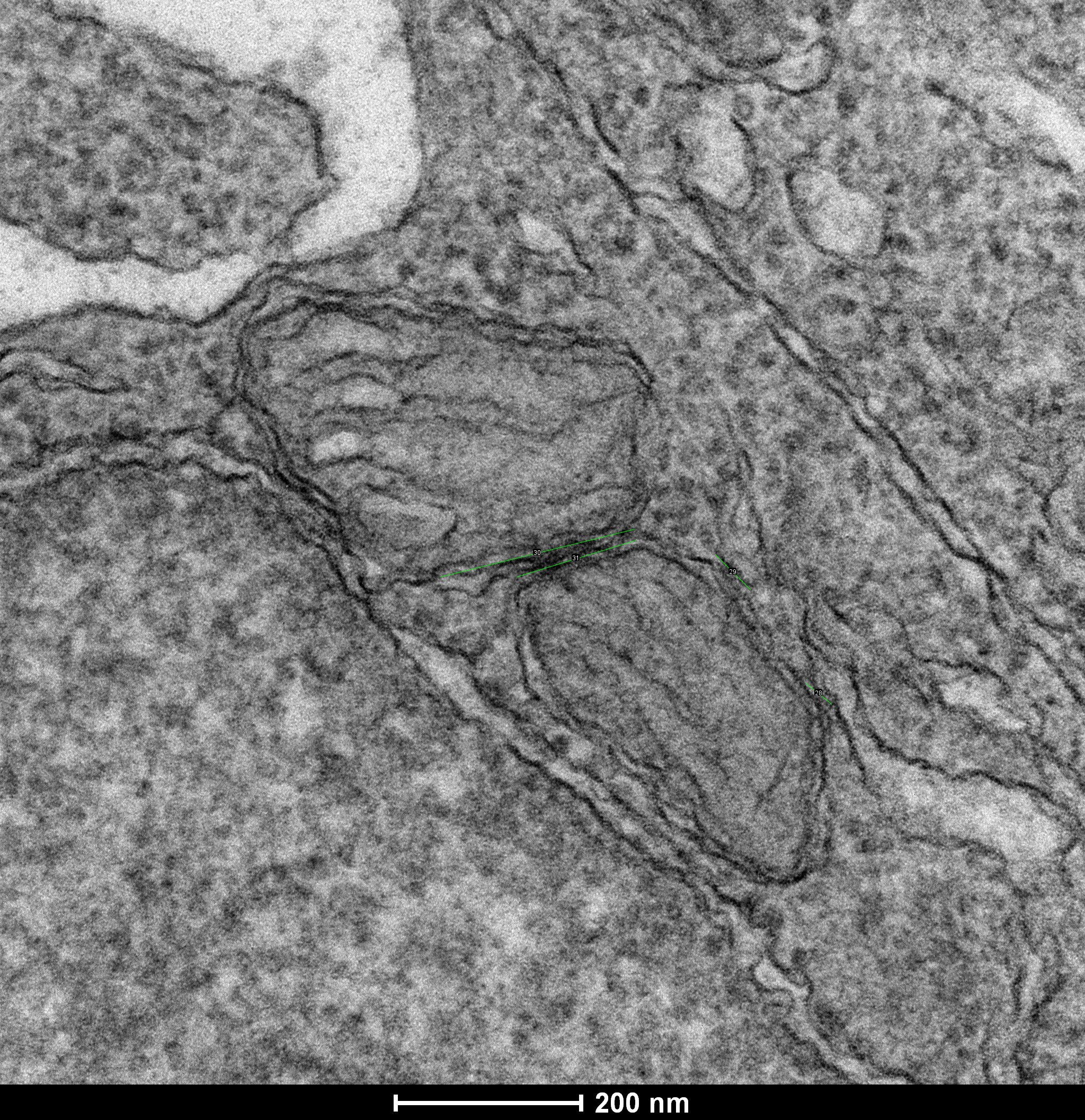

Supplement: S11 File — (ZIP) [file pone.0179859.s013.zip › Supplementary Images 4E/5a_L1_87000x_c2-m2m3.jpg]

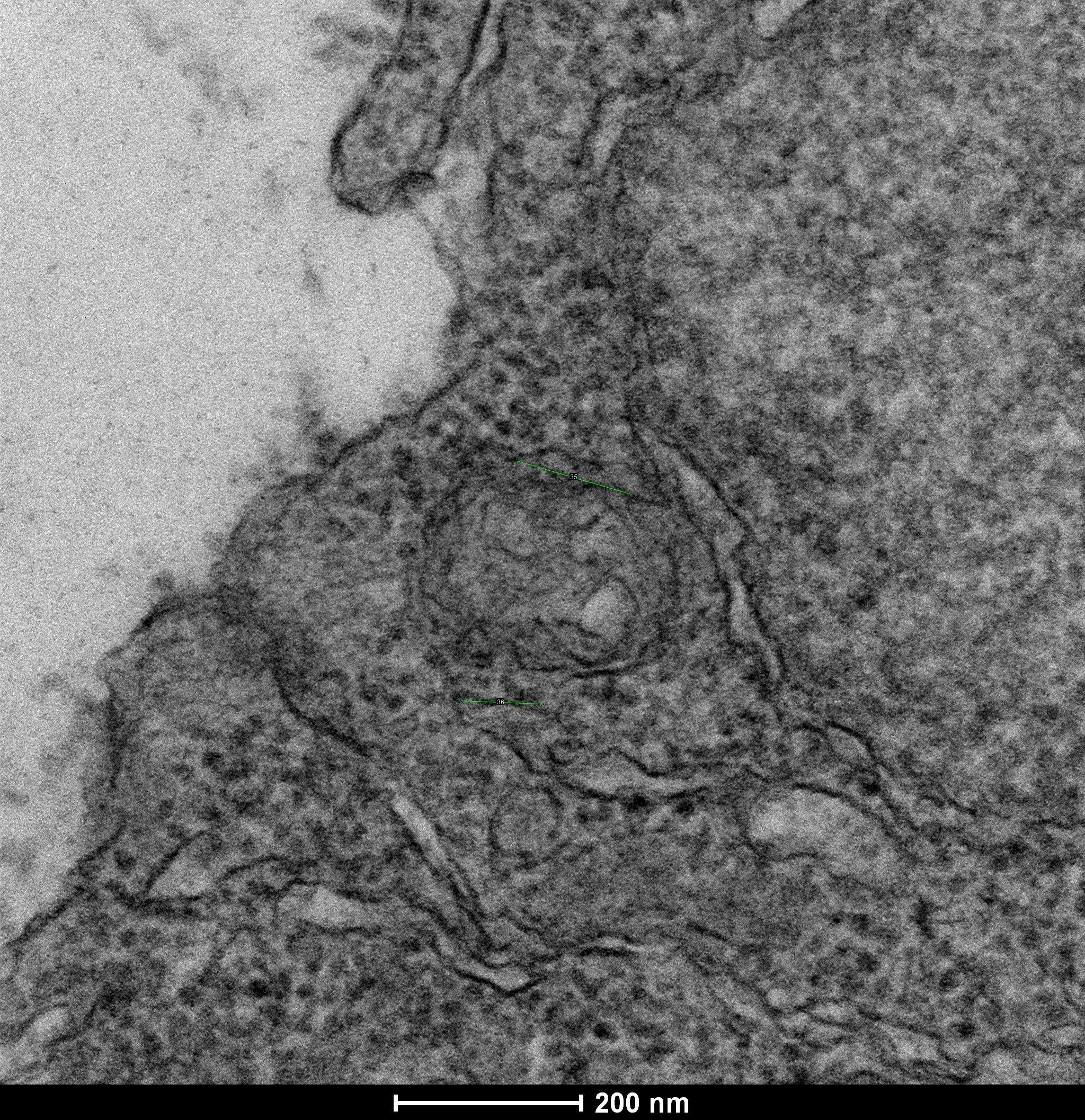

Supplement: S11 File — (ZIP) [file pone.0179859.s013.zip › Supplementary Images 4E/5a_L1_87000x_c4_m1.jpg]

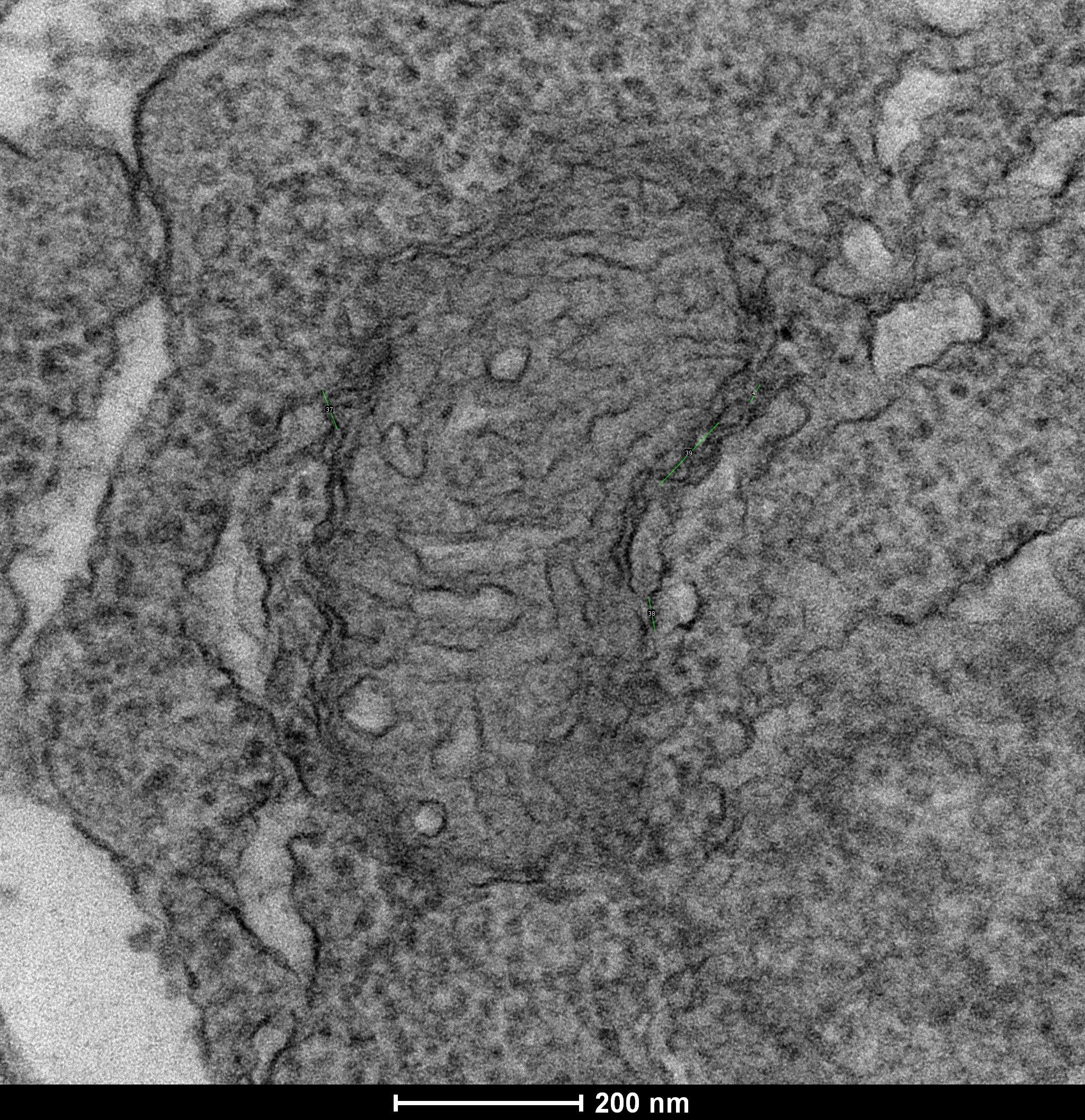

Supplement: S11 File — (ZIP) [file pone.0179859.s013.zip › Supplementary Images 4E/5a_L1_87000x_c5__m2.jpg]

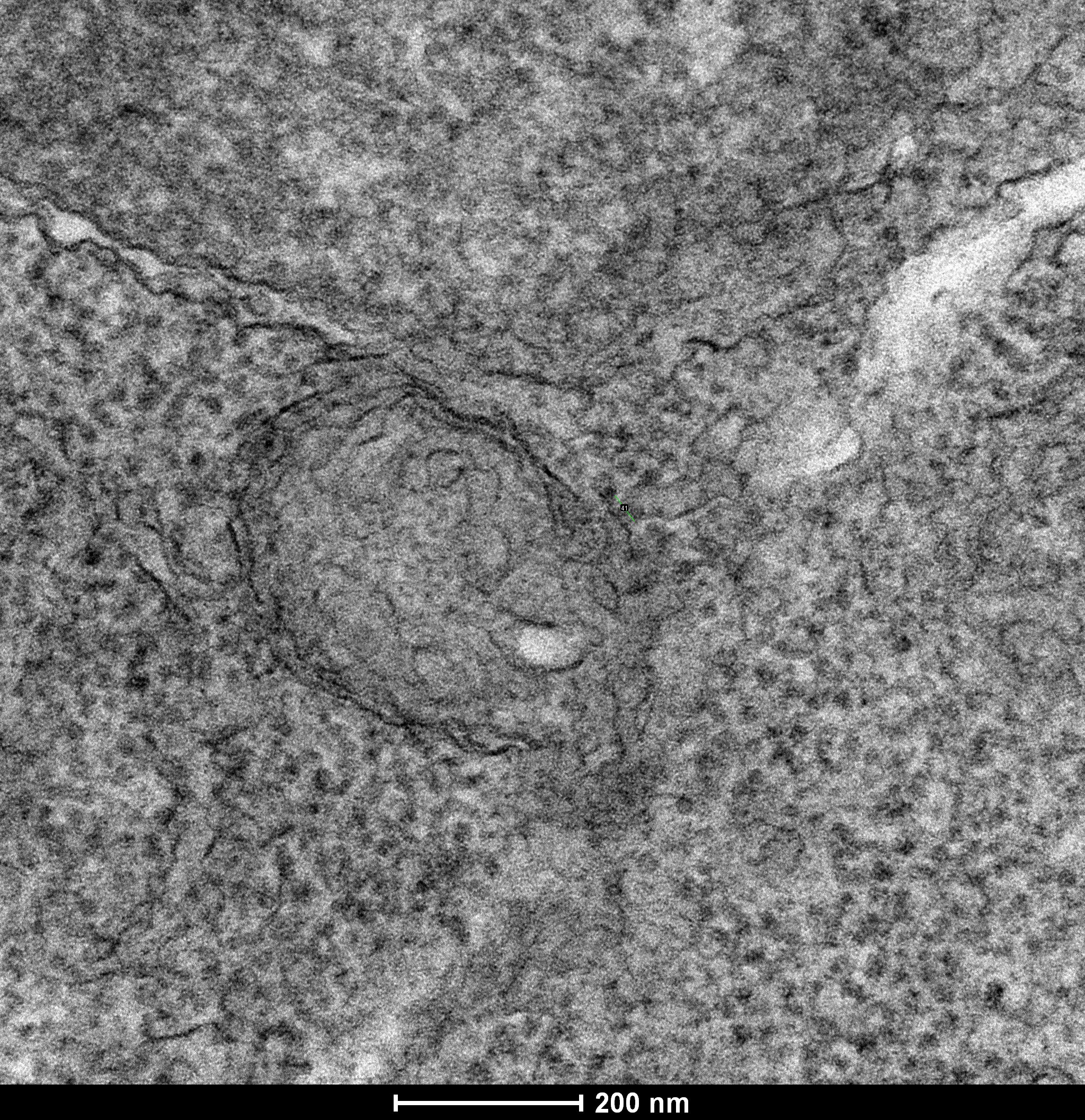

Supplement: S11 File — (ZIP) [file pone.0179859.s013.zip › Supplementary Images 4E/5a_L1_87000x_c5__m3.jpg]

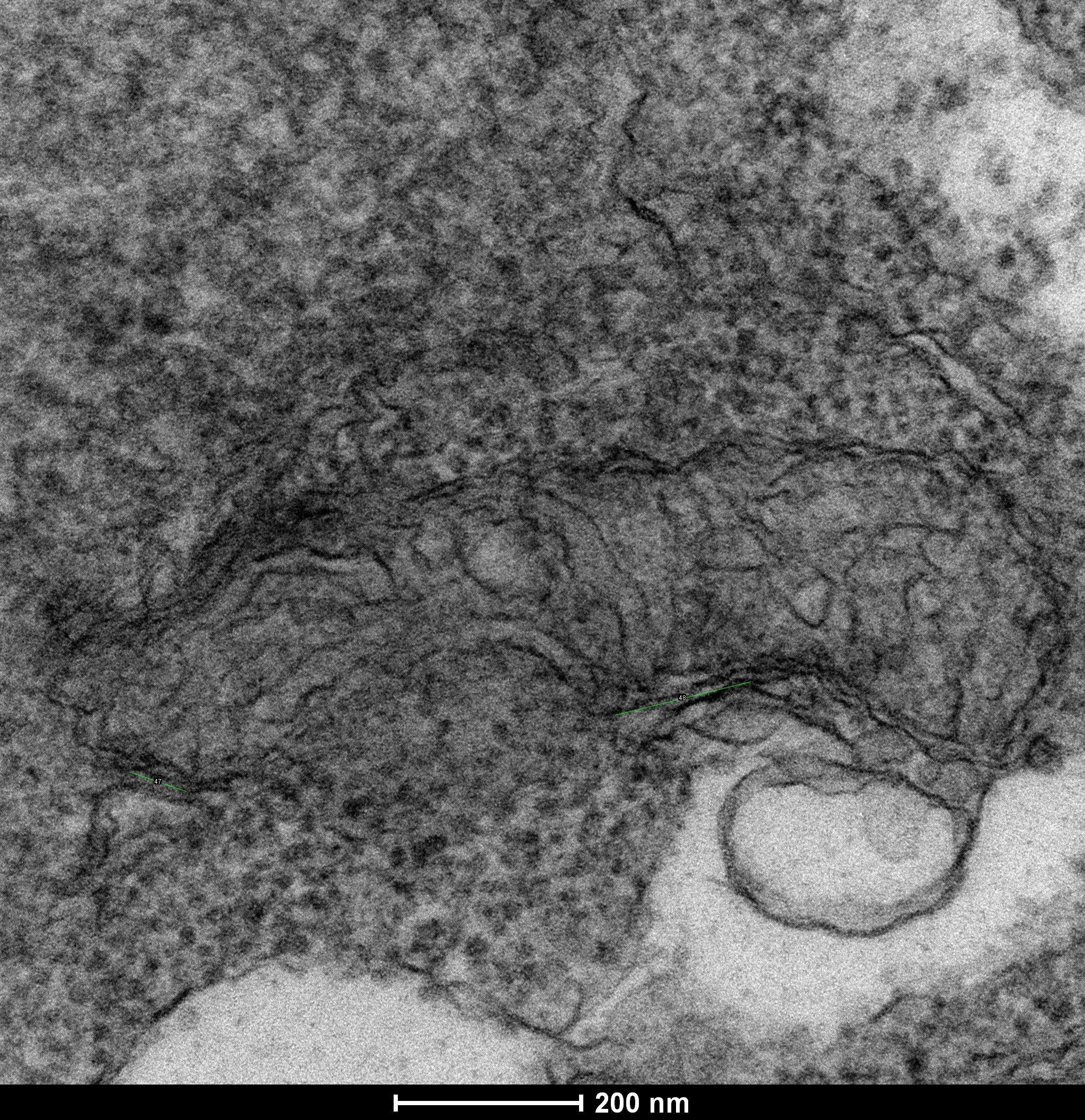

Supplement: S11 File — (ZIP) [file pone.0179859.s013.zip › Supplementary Images 4E/5a_L1_87000x_c5__m4.jpg]

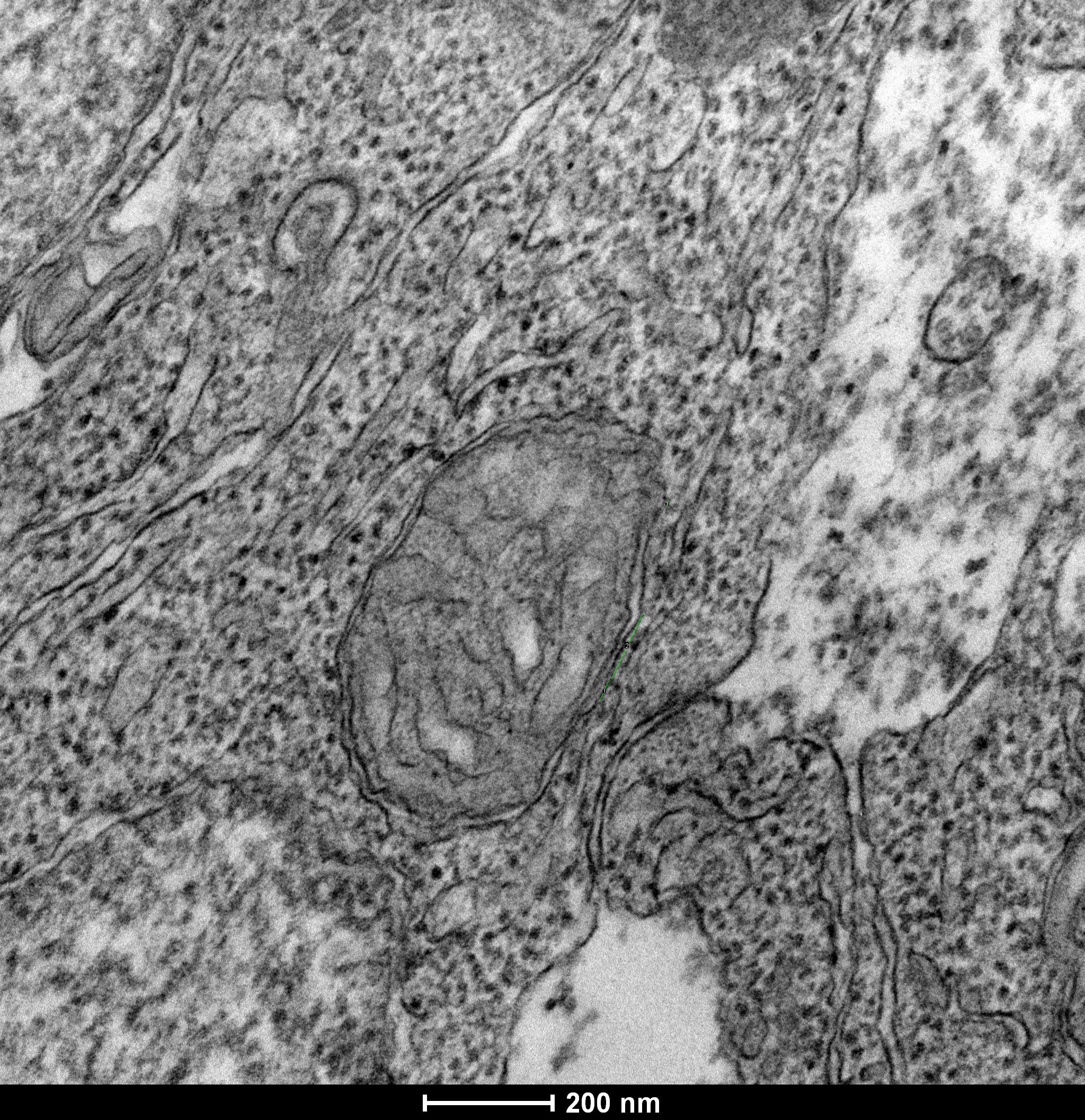

Supplement: S11 File — (ZIP) [file pone.0179859.s013.zip › Supplementary Images 4E/5b_L1_60000x_c2_m3.jpg]

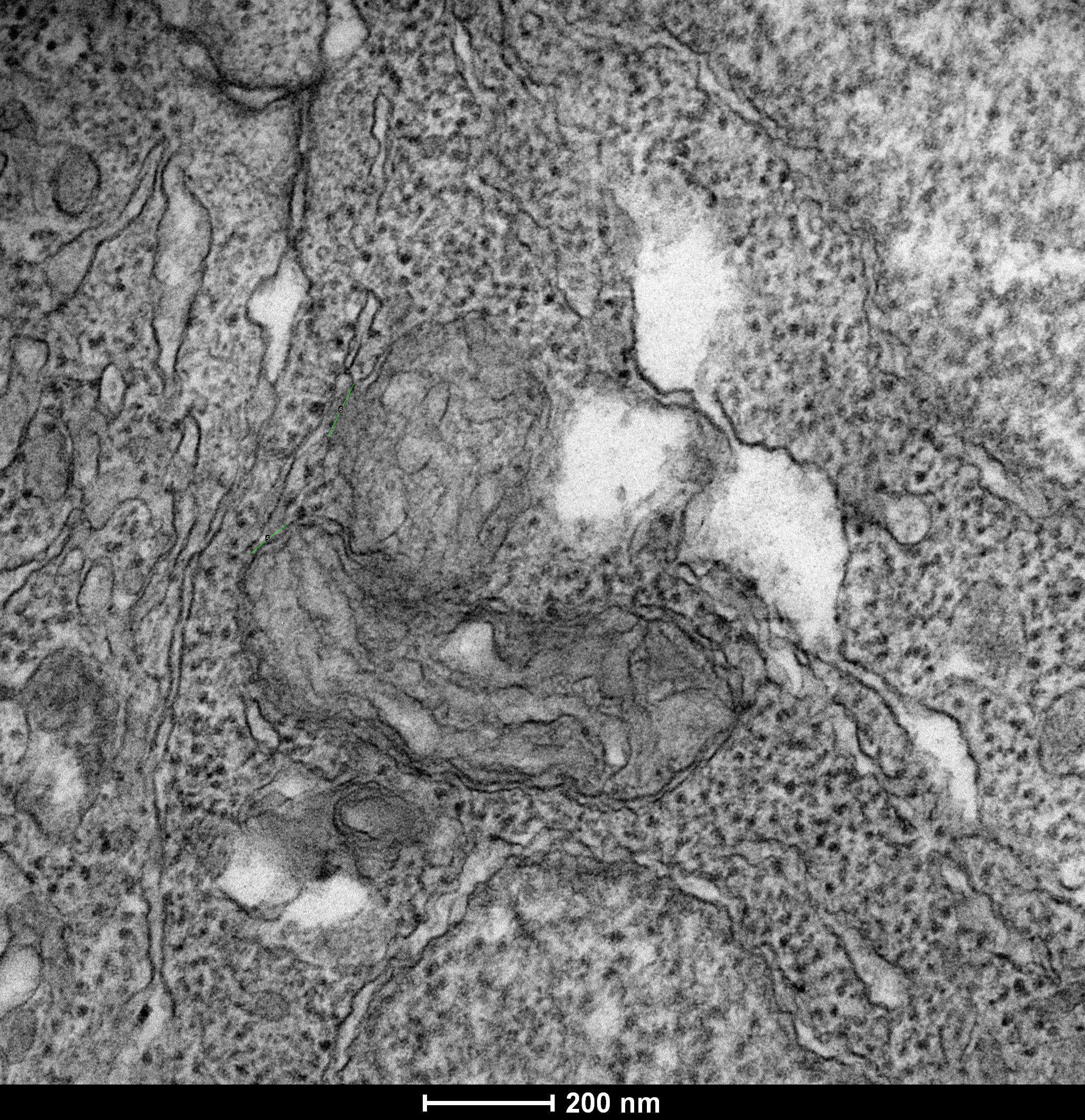

Supplement: S11 File — (ZIP) [file pone.0179859.s013.zip › Supplementary Images 4E/5b_L1_60000x_c7_m2_m3.jpg]

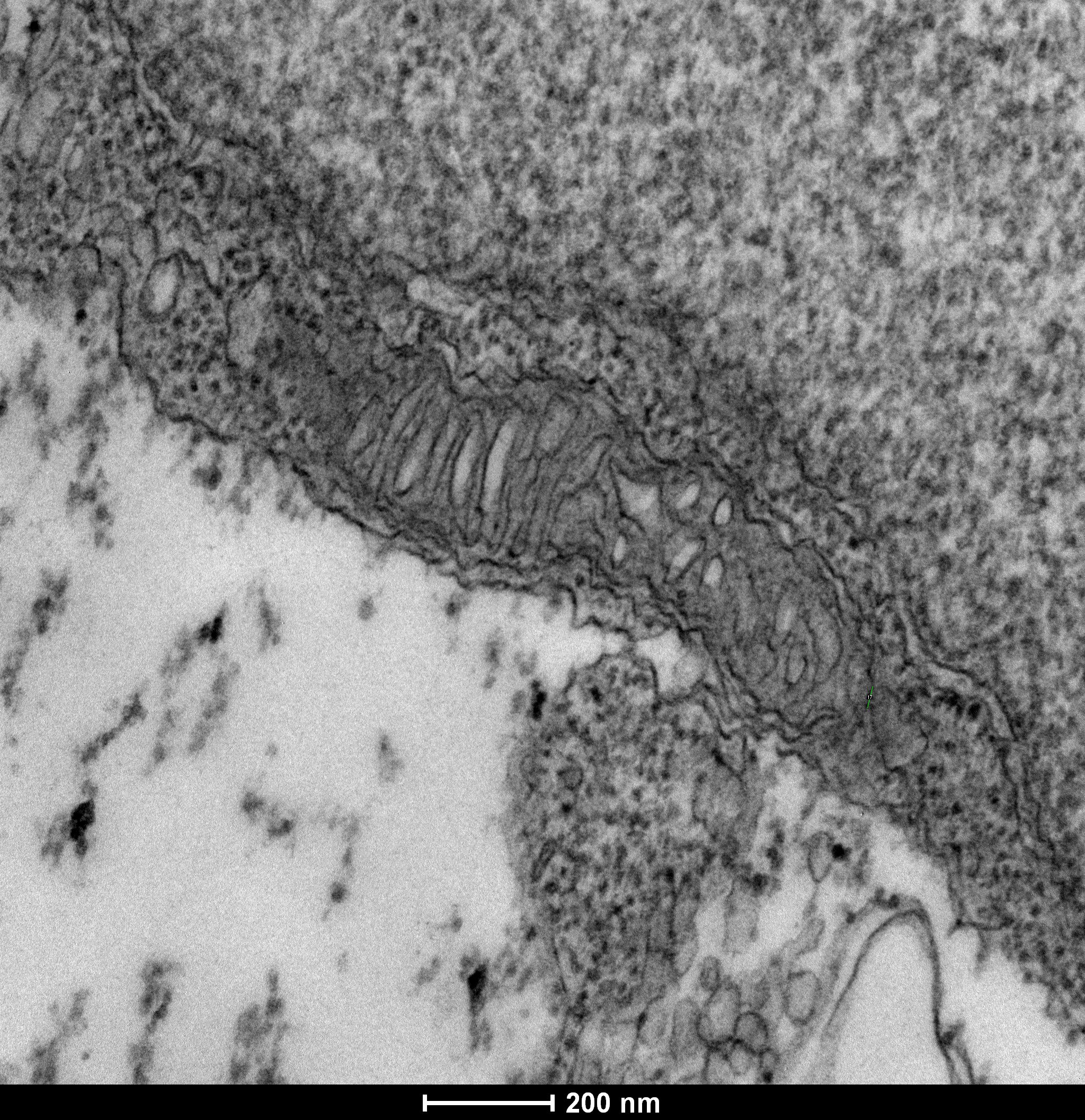

Supplement: S11 File — (ZIP) [file pone.0179859.s013.zip › Supplementary Images 4E/5b_L1_60000x_c9_m1.jpg]

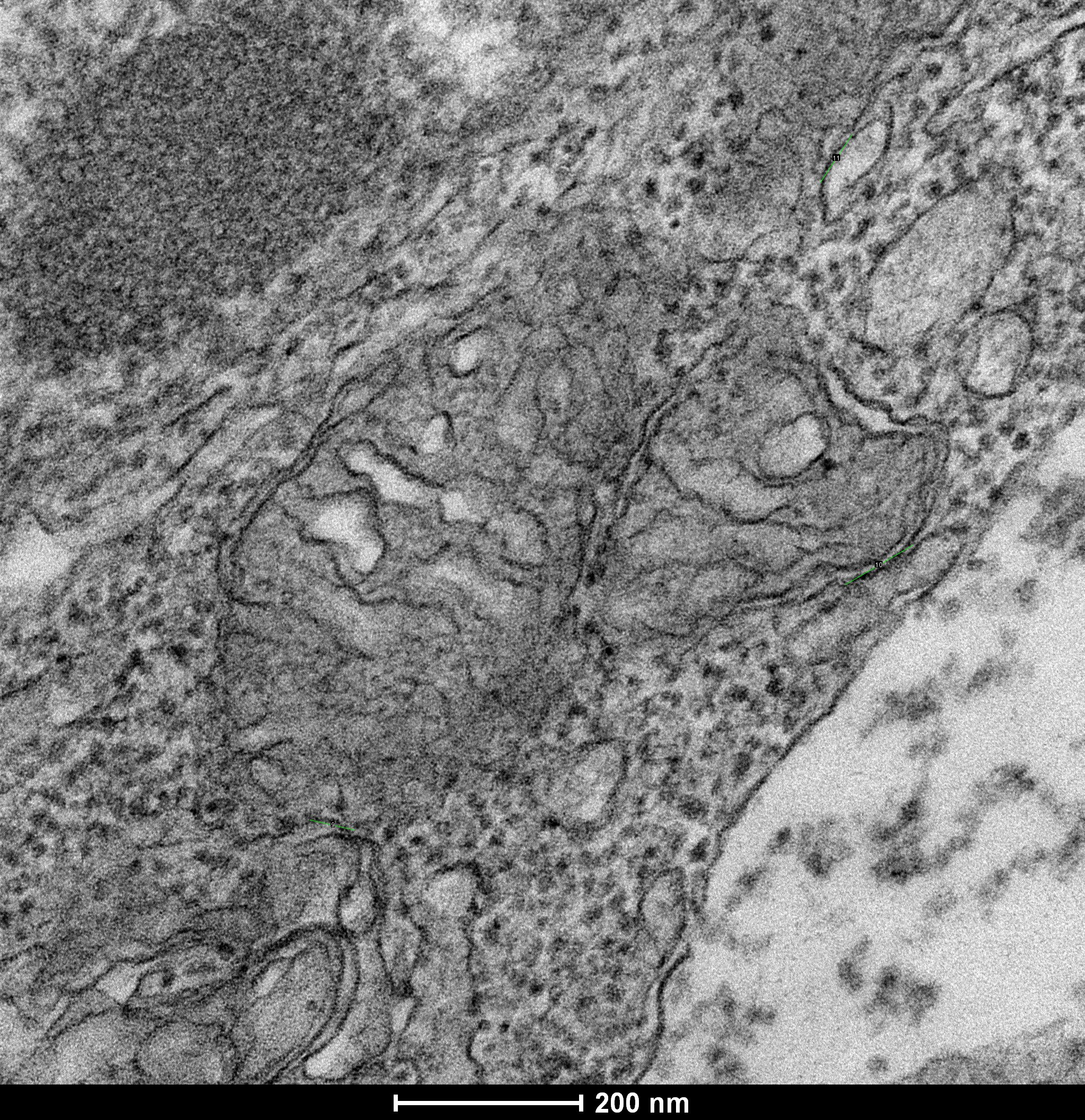

Supplement: S11 File — (ZIP) [file pone.0179859.s013.zip › Supplementary Images 4E/5b_L1_87000x_c4_m1_m2tif.jpg]

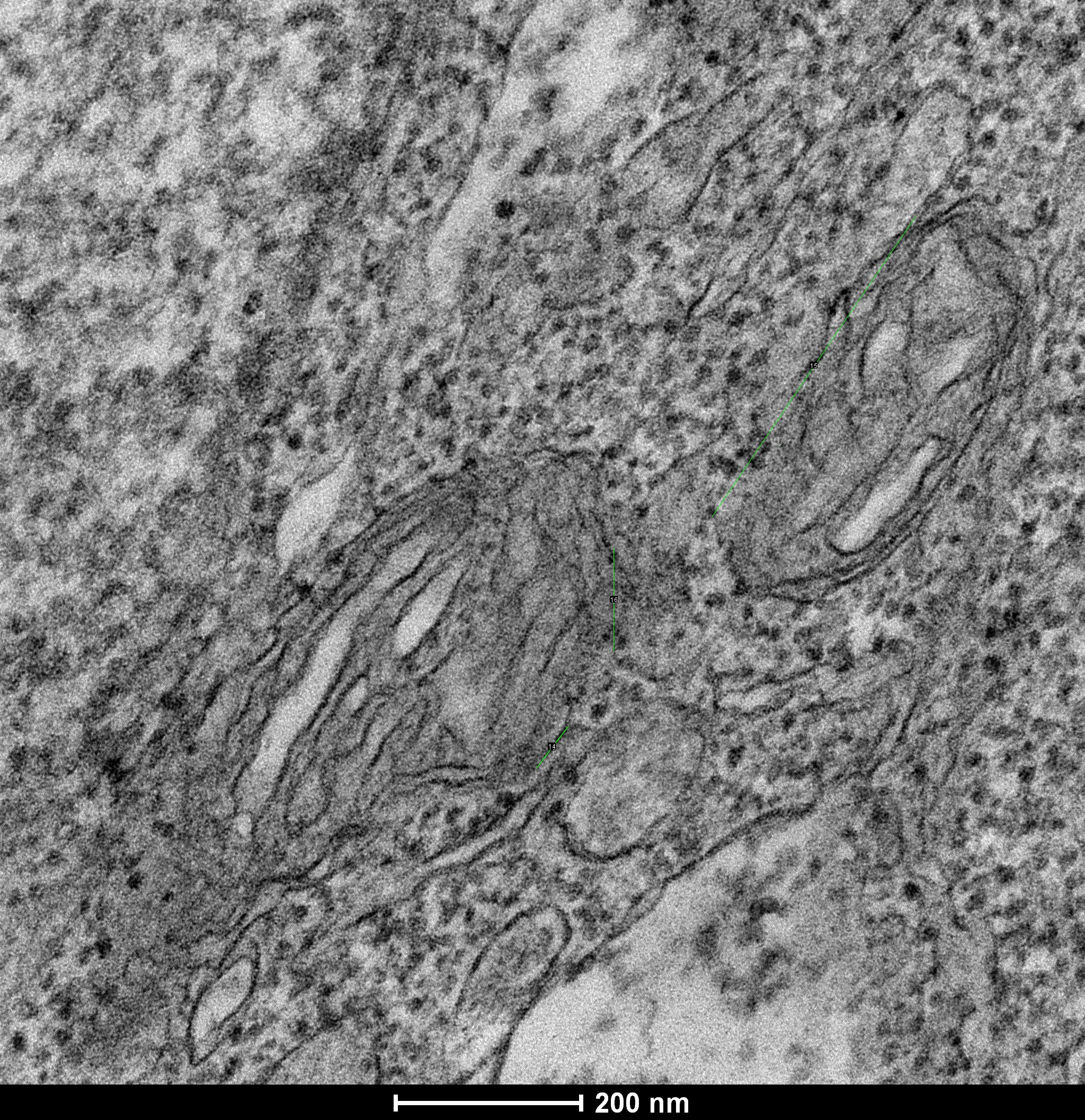

Supplement: S11 File — (ZIP) [file pone.0179859.s013.zip › Supplementary Images 4E/5b_L1_87000x_c4_m3_m4tif.jpg]

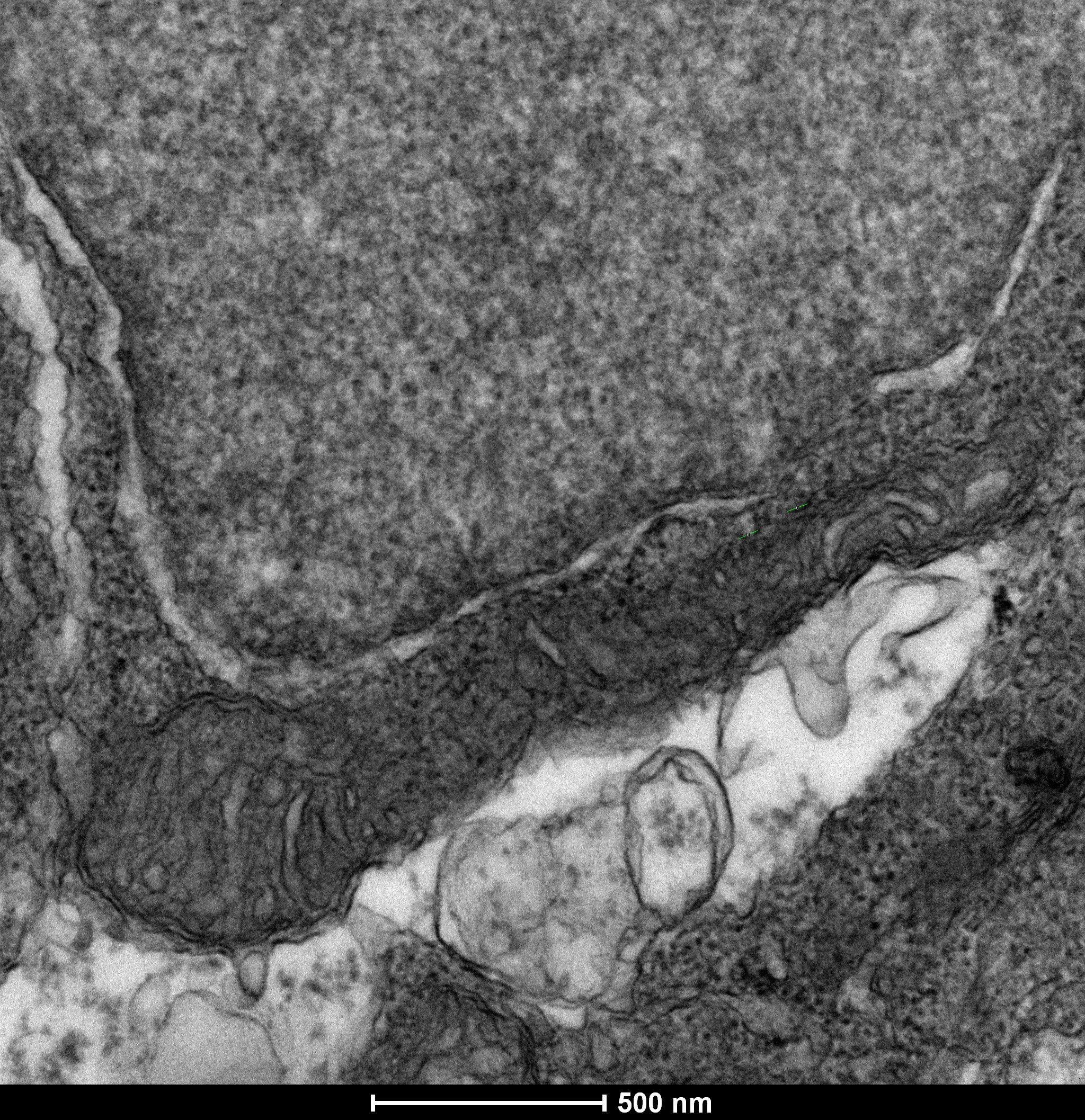

Supplement: S11 File — (ZIP) [file pone.0179859.s013.zip › Supplementary Images 4E/5c_L1_43000x_c2_m1.jpg]

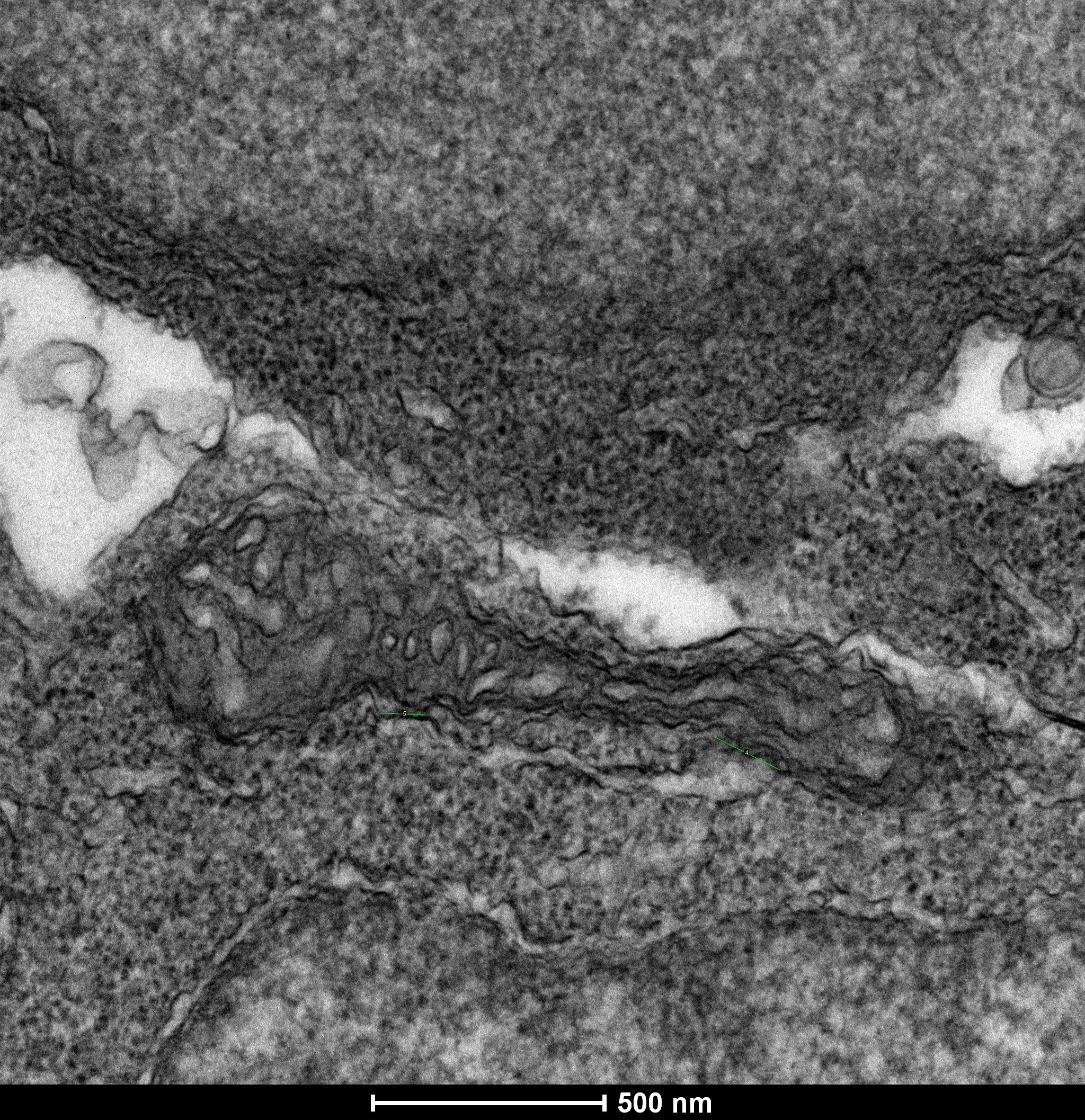

Supplement: S11 File — (ZIP) [file pone.0179859.s013.zip › Supplementary Images 4E/5c_L1_43000x_c3_m1.jpg]

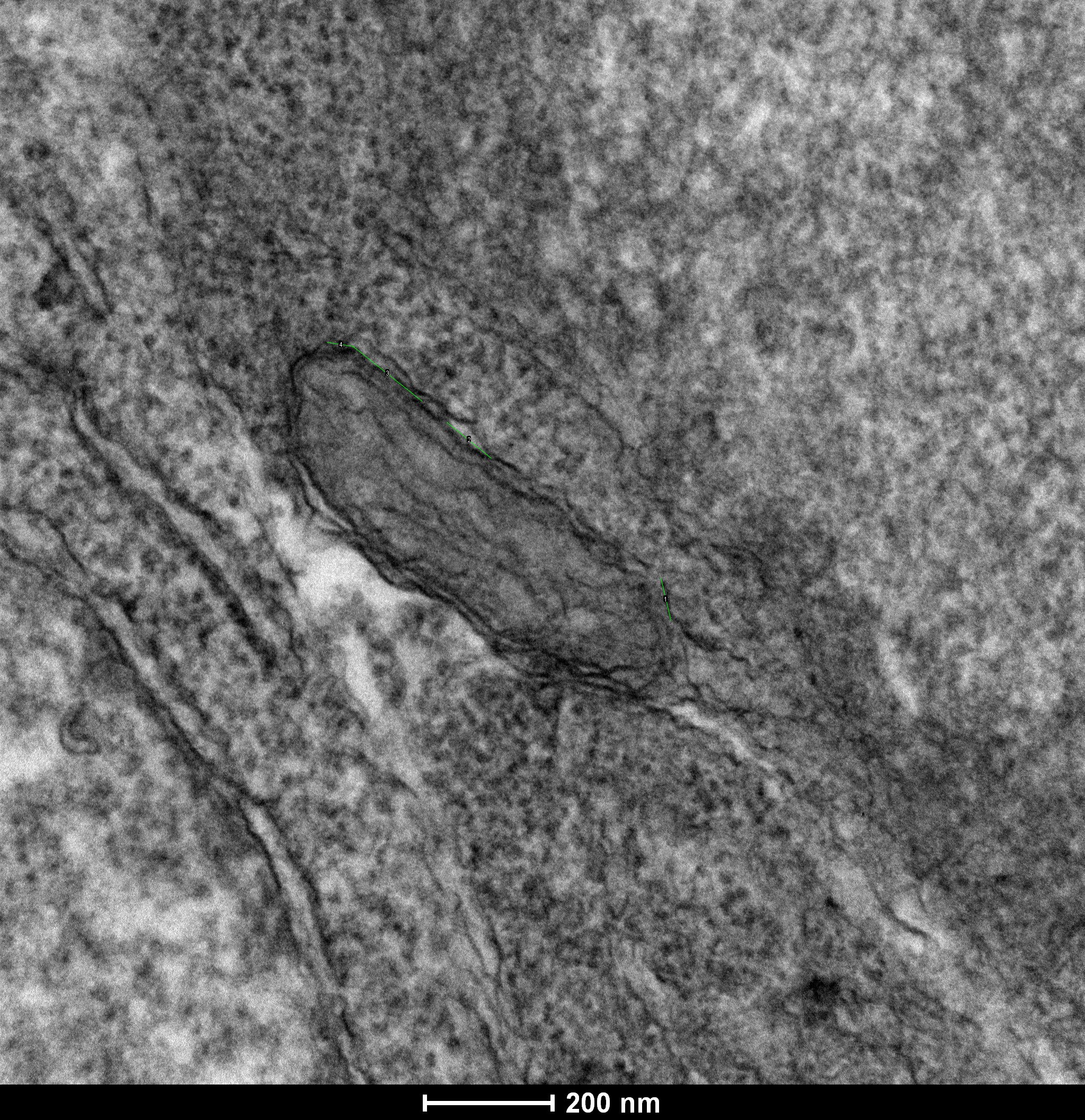

Supplement: S11 File — (ZIP) [file pone.0179859.s013.zip › Supplementary Images 4E/5c_L1_60000x_c1_m1.jpg]

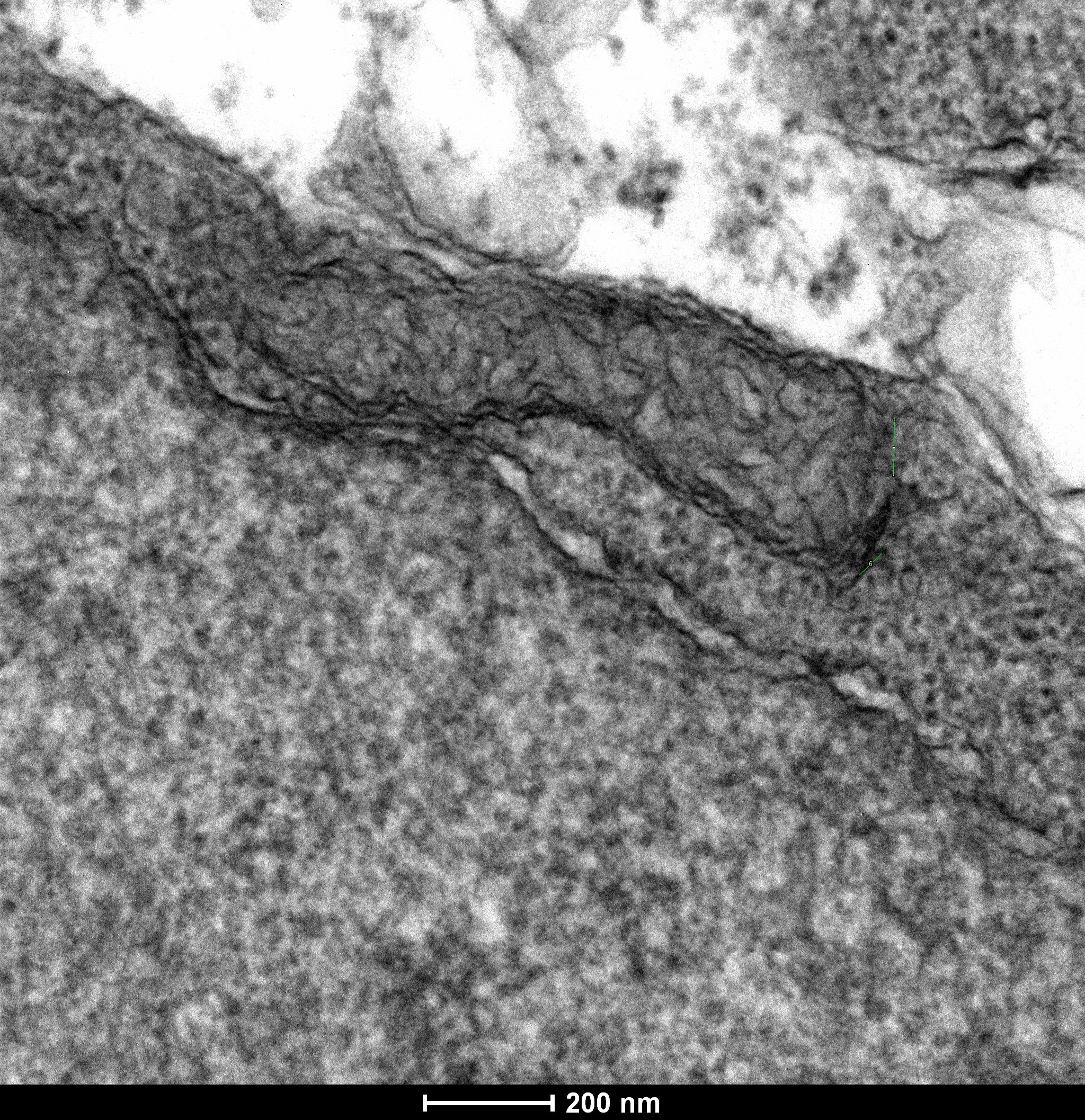

Supplement: S11 File — (ZIP) [file pone.0179859.s013.zip › Supplementary Images 4E/5c_L1_60000x_c1_m2.jpg]

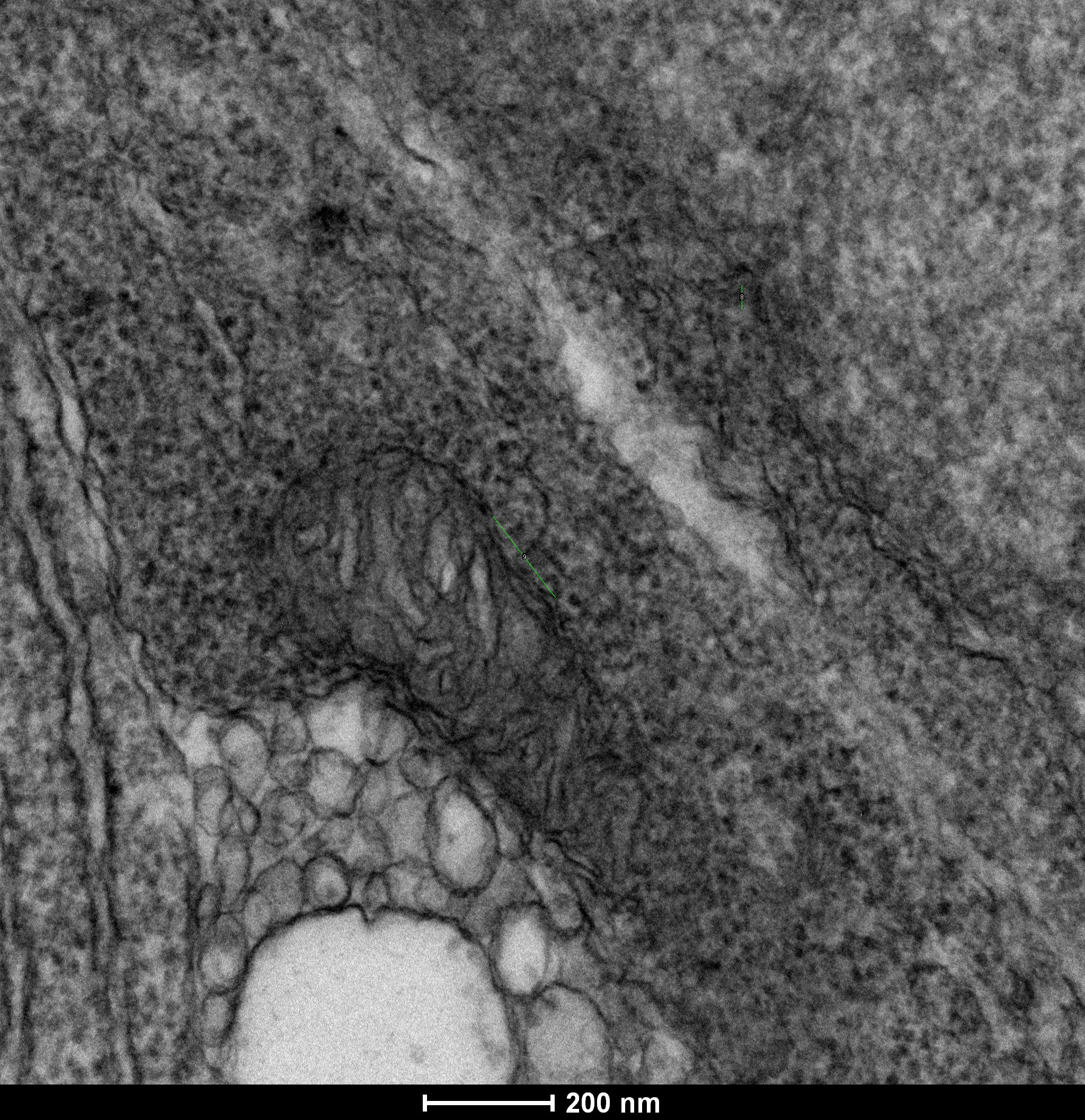

Supplement: S11 File — (ZIP) [file pone.0179859.s013.zip › Supplementary Images 4E/5c_L1_60000x_c2_m2.jpg]

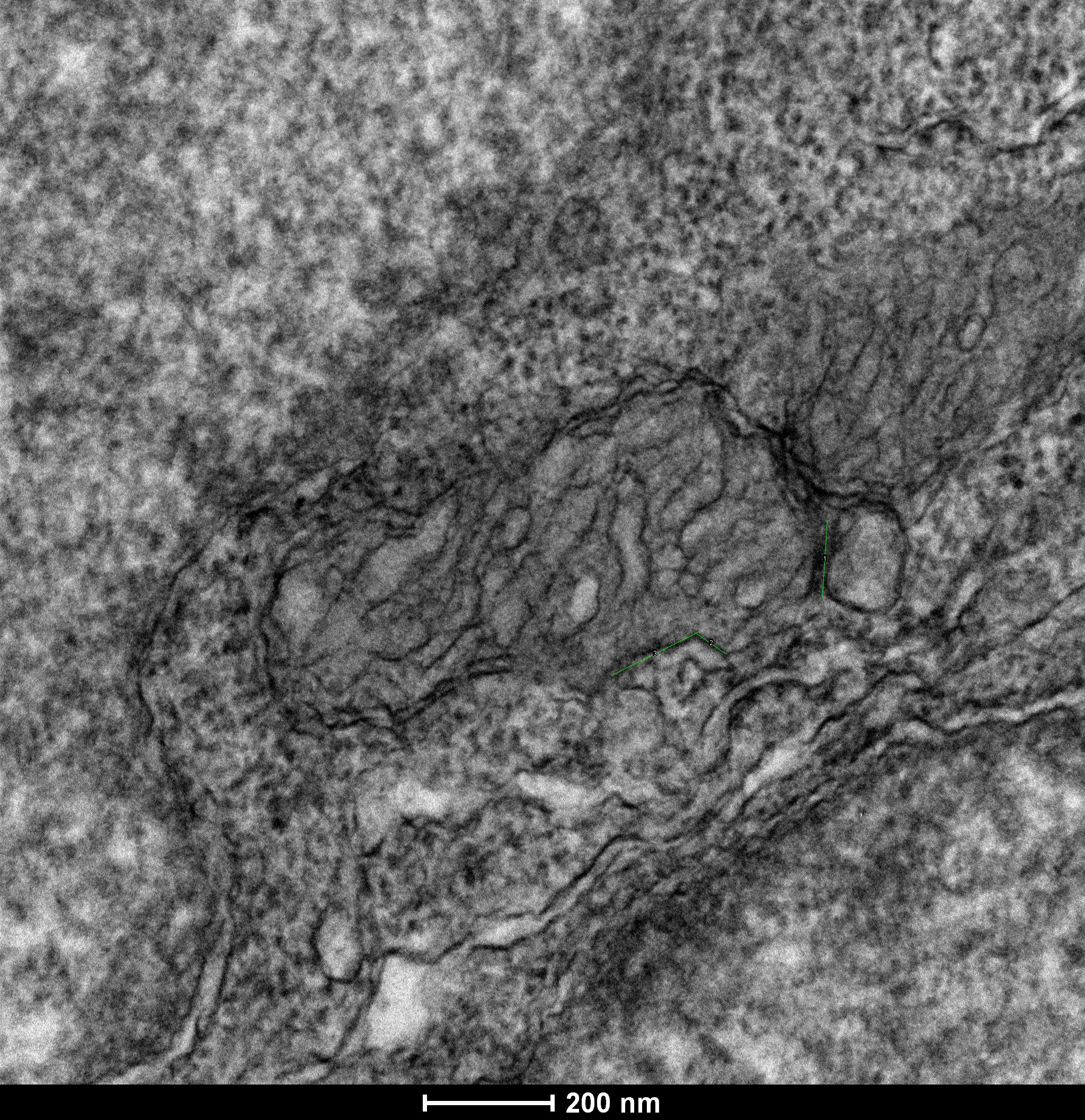

Supplement: S11 File — (ZIP) [file pone.0179859.s013.zip › Supplementary Images 4E/5c_L1_60000x_c3_m2.jpg]

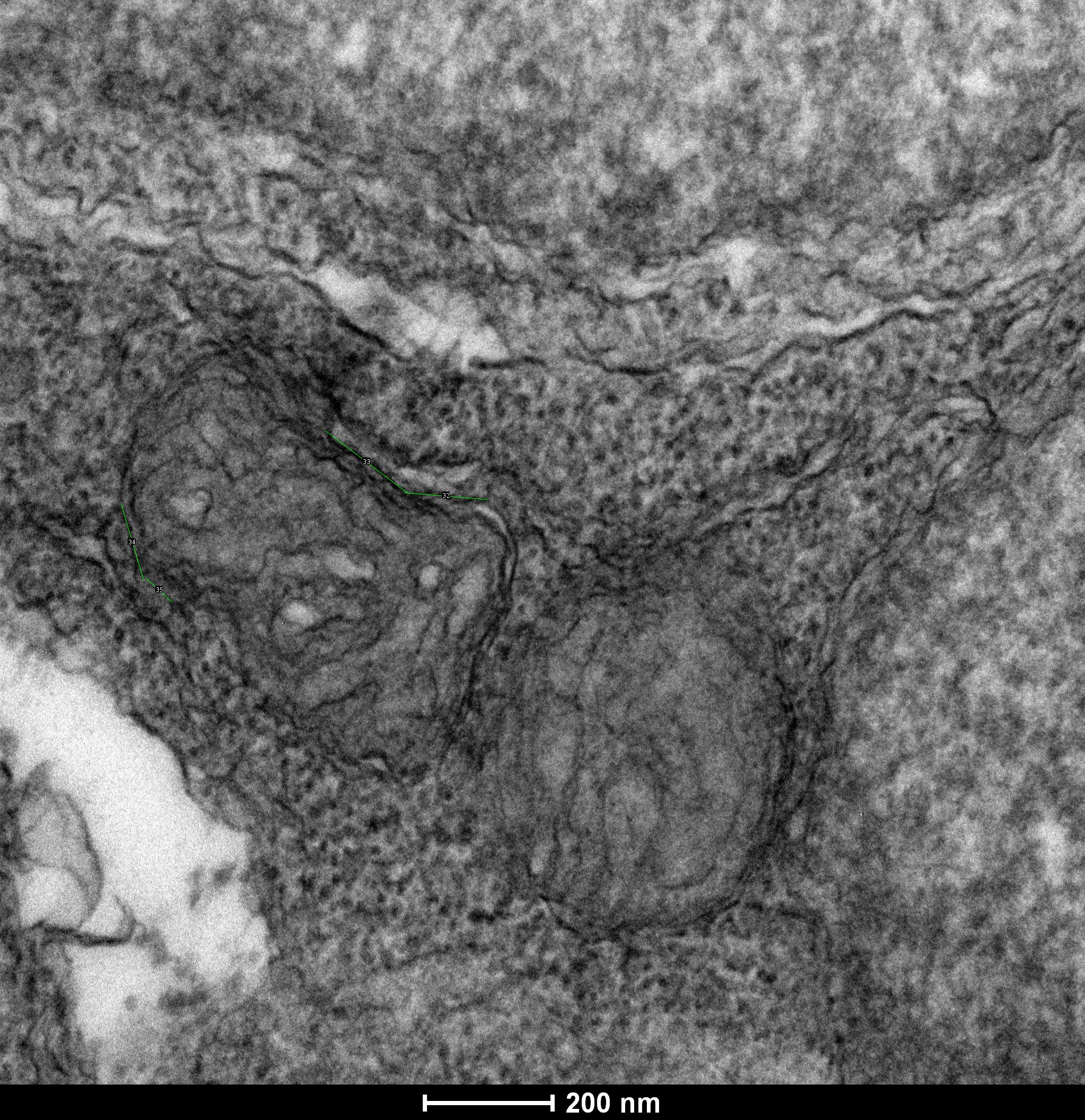

Supplement: S11 File — (ZIP) [file pone.0179859.s013.zip › Supplementary Images 4E/5c_L1_60000x_c4_m1_m2.jpg]

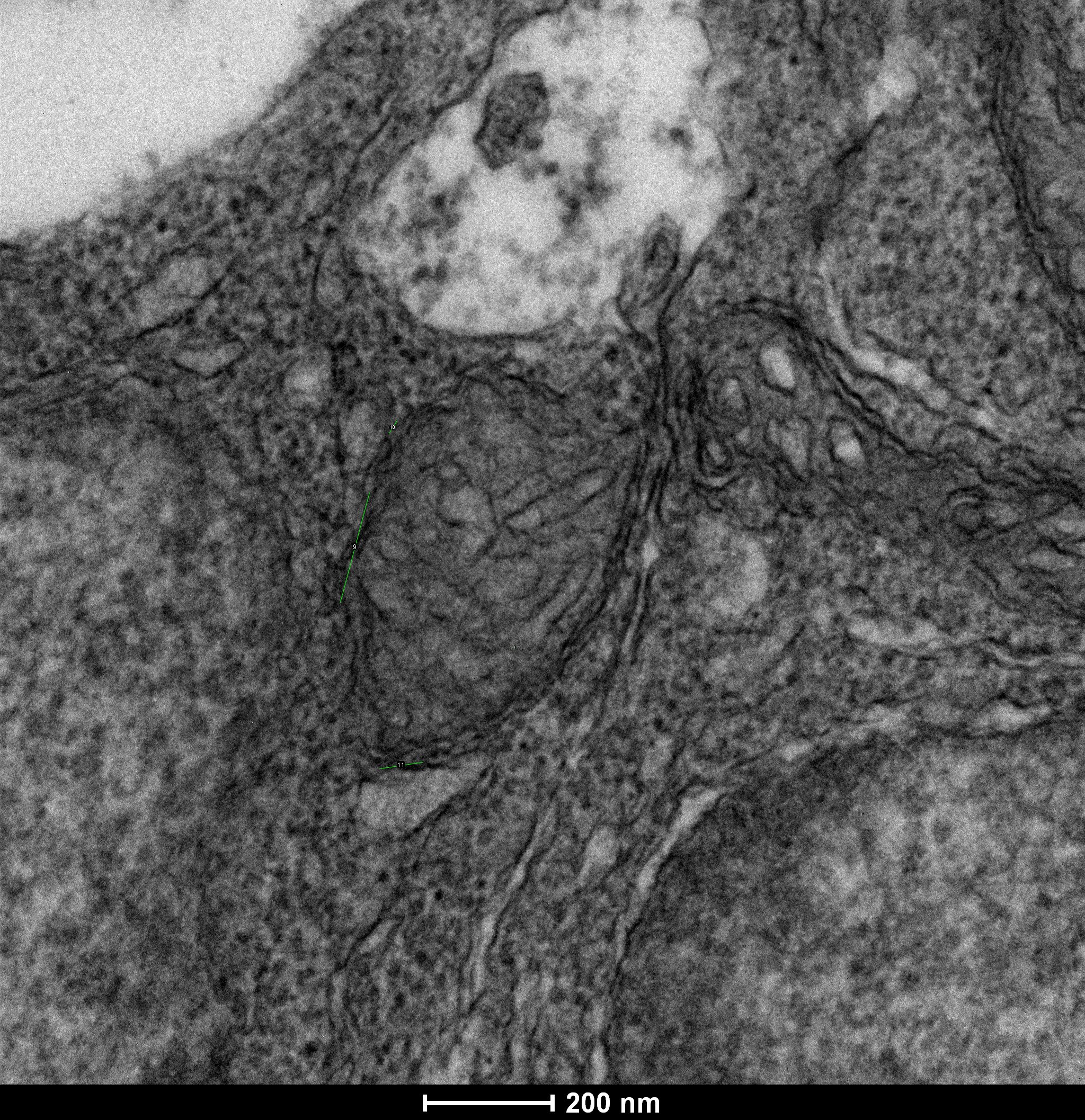

Supplement: S11 File — (ZIP) [file pone.0179859.s013.zip › Supplementary Images 4E/5c_L1_60000x_c4_m3.jpg]

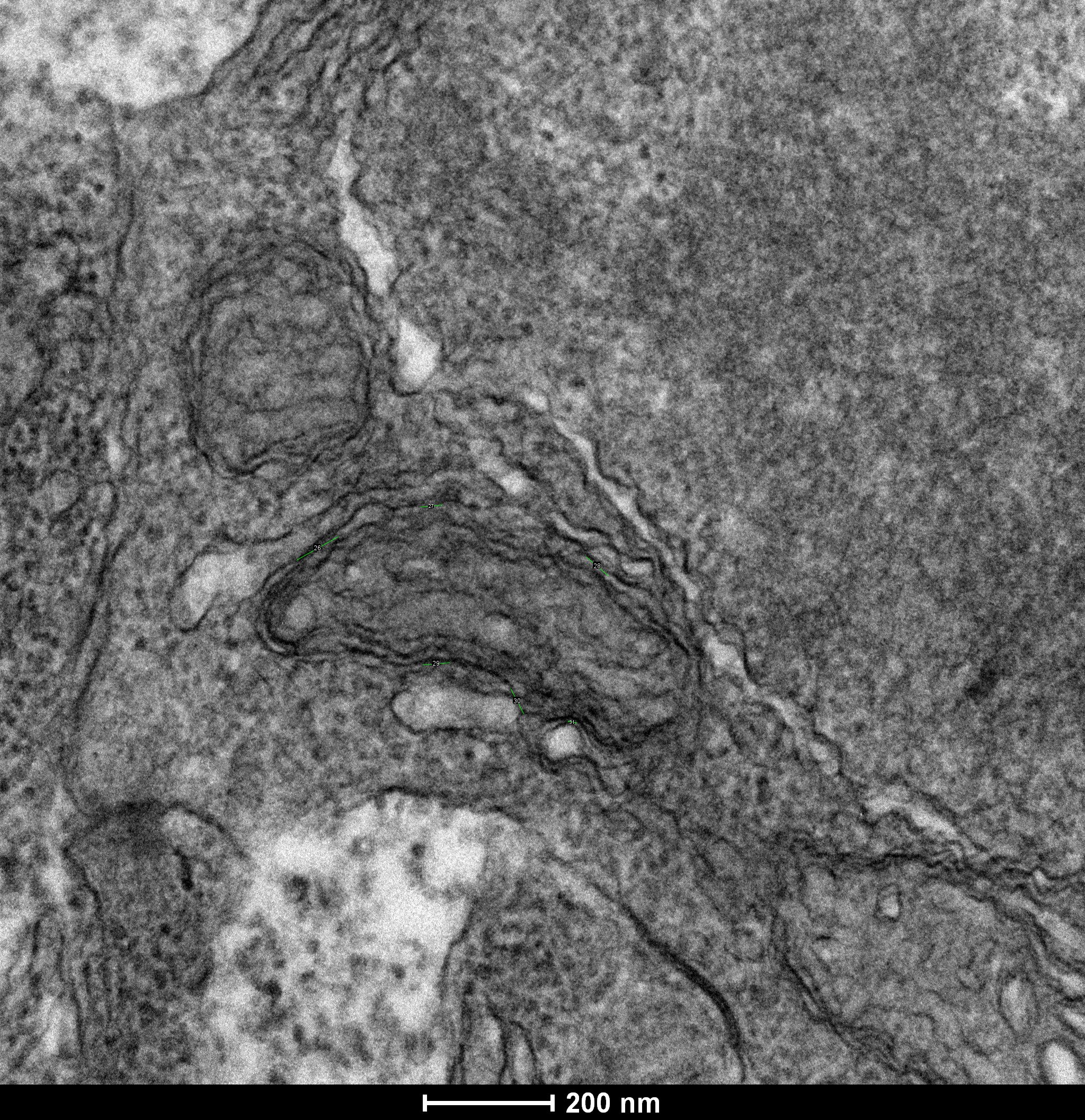

Supplement: S11 File — (ZIP) [file pone.0179859.s013.zip › Supplementary Images 4E/5c_L1_60000x_c5_m1_m2.jpg]

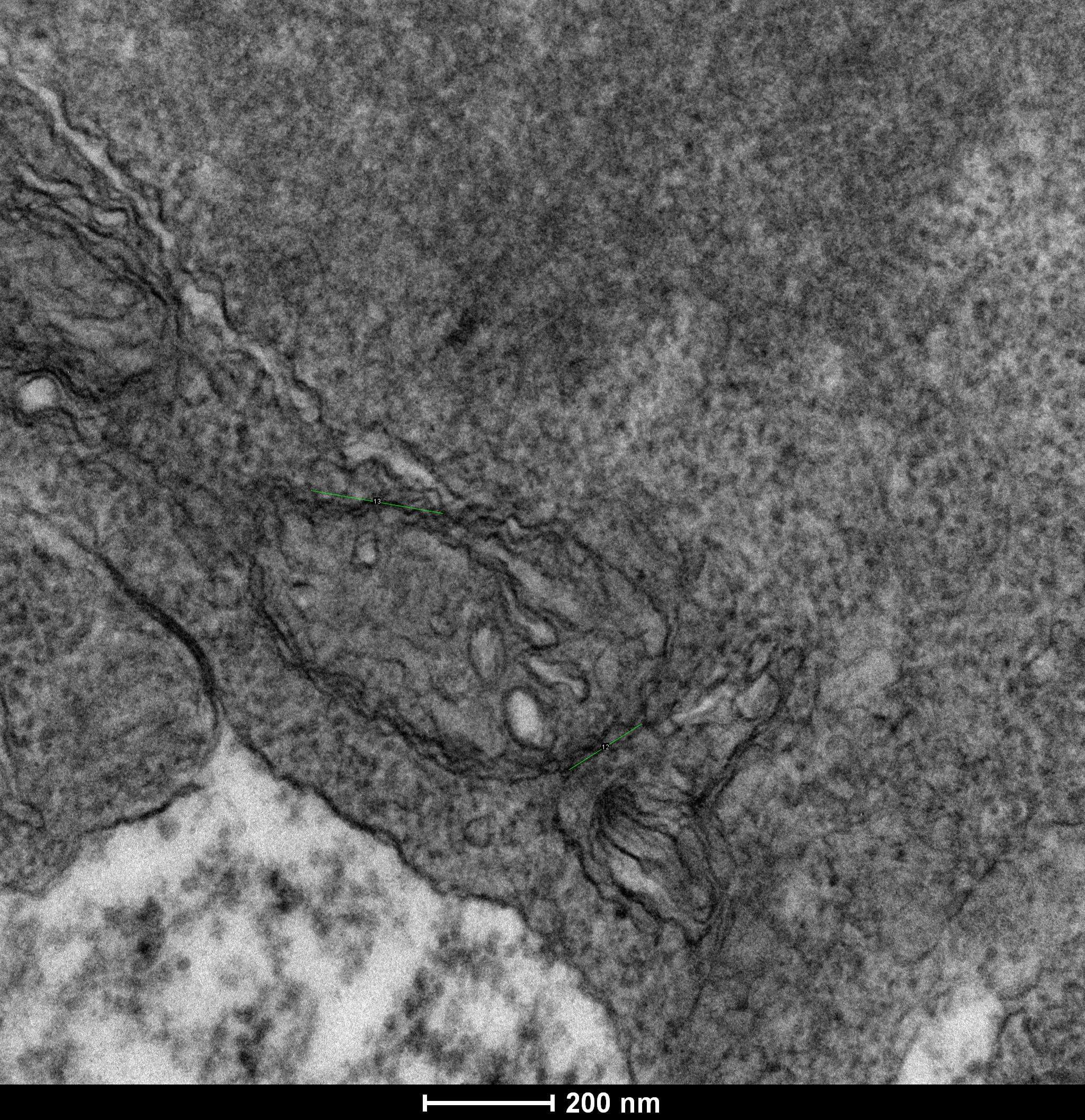

Supplement: S11 File — (ZIP) [file pone.0179859.s013.zip › Supplementary Images 4E/5c_L1_60000x_c5_m3.jpg]

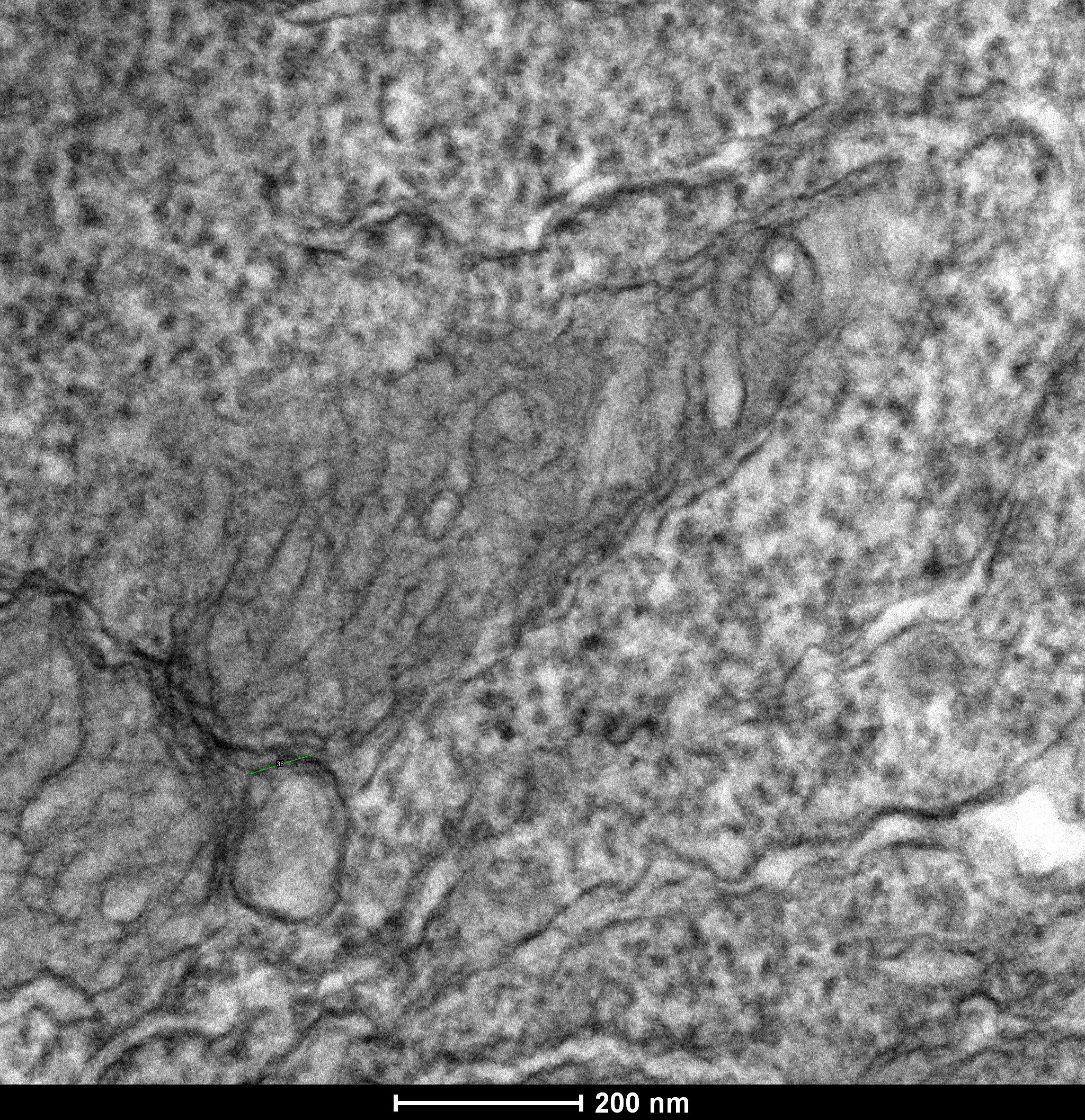

Supplement: S11 File — (ZIP) [file pone.0179859.s013.zip › Supplementary Images 4E/5c_L1_87000x_c3_m3.jpg]
